# Supplementary material for: A selective and orally bioavailable VHL-recruiting PROTAC achieves SMARCA2 degradation in vivo
Source: Nat Commun. 2022 Oct 10;13:5969. doi: 10.1038/s41467-022-33430-6 (PMC9551036; doi:10.1038/s41467-022-33430-6)
Supplement: Supplementary file 1 — Supplementary Information [file 41467_2022_33430_MOESM1_ESM.pdf]

Supplementary Information:

A selective and orally bioavailable VHL-recruiting PROTAC achieves SMARCA2 degradation *in vivo*

Kofink, Trainor, Mair et al.

Contents **Page**

Contents	Page
**Supplementary Figures 1-6**	**2**
**Crystallography data collection and refinement statistics**	
- Supplementary Tables 1-4	12
**Supplementary Methods**	
- NMR methods	
- Experiments for compound 11 and ACBI2	17
- Supplementary Figures 7-11	18
- Supplementary Tables 5 and 6	21
- Chemical Synthesis	
- Supplementary Figure 12 (final compound structures)	23
- General information	27
- Synthesis of bromodomain ligands (with Supplementary Figures 13-16)	28
- Synthesis of phenolic PROTACs (with Supplementary Figures 17-19)	42
- Synthesis of benzylic PROTACs (with Supplementary Figures 20-31)	49
- Supplementary Figures 32-63 (1H and 13C 1D NMR spectra for final compounds)	104
**Supplementary References**	**136**

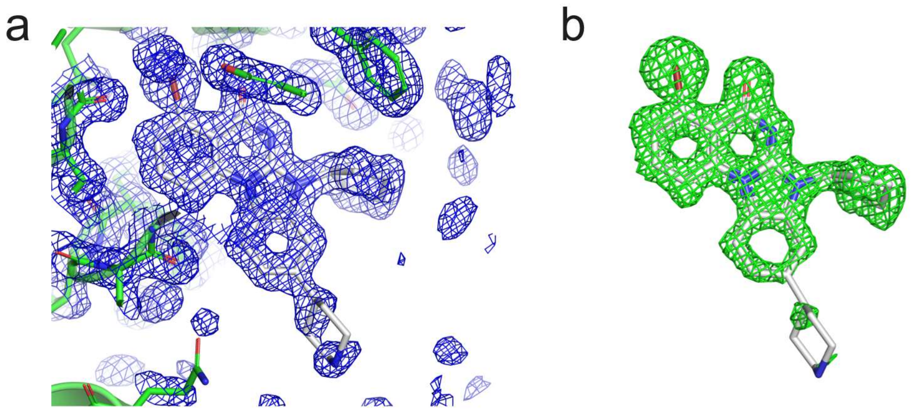

Supplementary Figure 1. Co-crystal structure of the SMARCA2^{BD} : compound 4 complex. a. Representative 2Fo-Fc electron density for compound **4** co-crystal structure at 1 σ contour level. Green: SMARCA2, Yellow: VHL. **b.** Fo-Fc omit map for compound **4** co-crystal structure at 3 σ contour level.

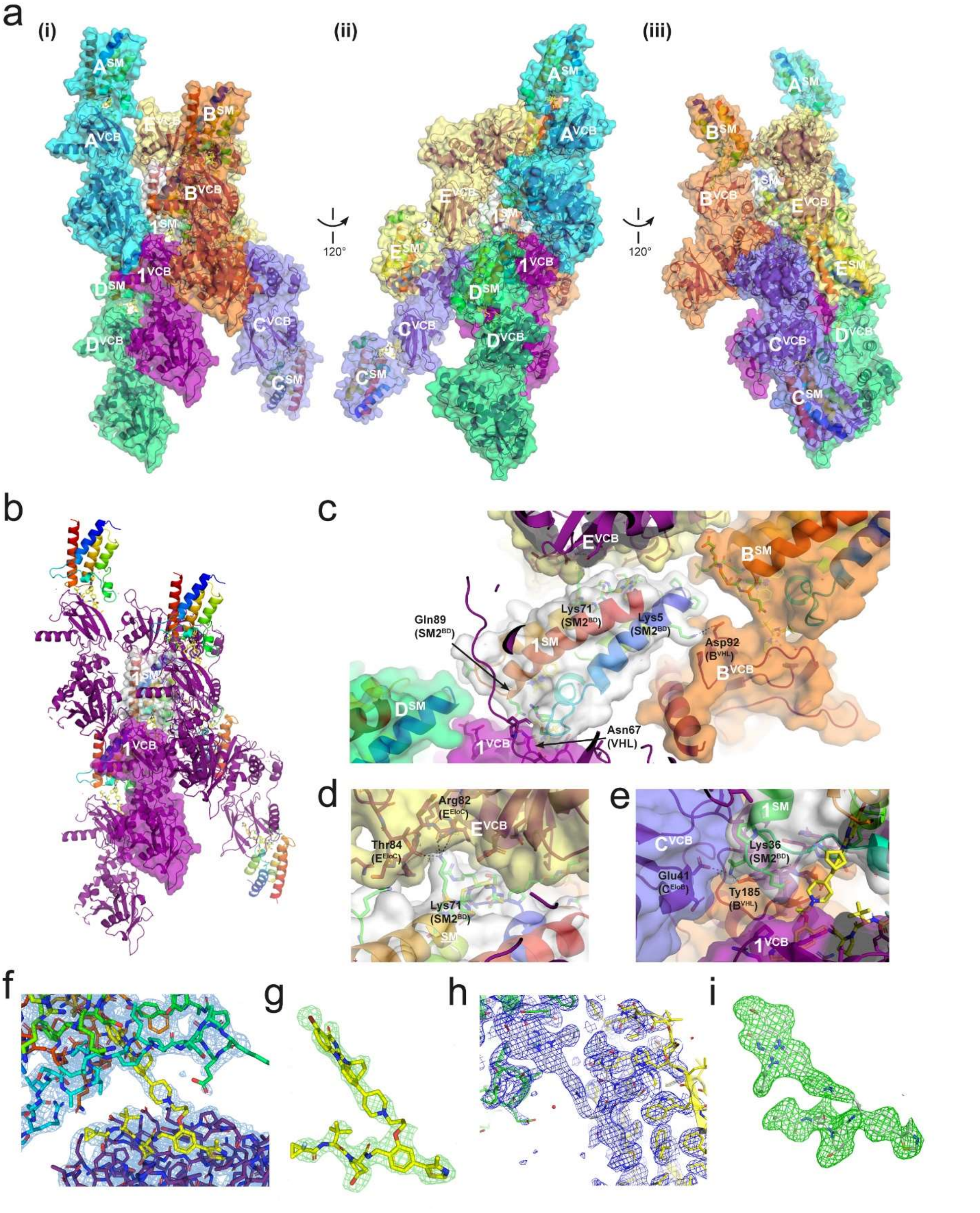

Supplementary Figure 2. Close crystal packing in the VCB : compound 5 : SMARCA2^{BD} ternary complex crystal structure. Cartoon and surface representation showing central single copy (1) of the VCB : compound **5** : SMARCA2^{BD} ternary complex comprising the asymmetric unit (asu), surrounded by closely packed symmetry-related copies (A-E, surface coloured teal, orange, blue, green, yellow respectively in

panel **a**). Panels **a(i)** and **b** show the identical orientation, however in **b** for symmetry copies A-E the surface is not shown, to more clearly identify the asu (copy 1, both cartoon and surface in both panels; VCB shown as purple cartoon and surface; compound **5** shown as yellow sticks; SMARCA2^{BD}, SM, shown as rainbow cartoon from blue N--terminus to red C-terminus and light grey surface). Panels **c-e** show in closeup some of the interactions of the SMARCA2^{BD} that mediate crystal contacts, including hydrogen bond/salt bridge interactions and van der Waals interactions, in particular residues Lys5, Lys36 and Lys71 of SMARCA2^{BD}. PDB: 7Z6L. **f.** Ternary X-ray crystal structure for VCB : compound **5** : SMARCA2^{BD} (PDB: 7Z6L), showing electron density map (2Fobs-Fc, blue mesh, contour level 1.0 σ, carve radius 2.0 Å), protein (VCB: purple sticks, SMARCA2^{BD}: rainbow sticks corresponding to Figure 2b) and modelled compound **5** (yellow sticks). **g.** Simulated anneal omit map with ligand removed (Fobs-Fc, green mesh, contour level 3.0 σ, carve radius 2.0 Å) overlaid with modelled compound **5** (yellow sticks), from the VCB : compound **5** : SMARCA2^{BD} (PDB: 7Z6L) ternary complex. **h.** Representative 2Fo-Fc electron density for compound **6** co-crystal structure at 1 σ contour level. Green: SMARCA2, Yellow: VHL. **i.** Fo-Fc omit map for compound **6** co-crystal structure at 3 σ contour level.

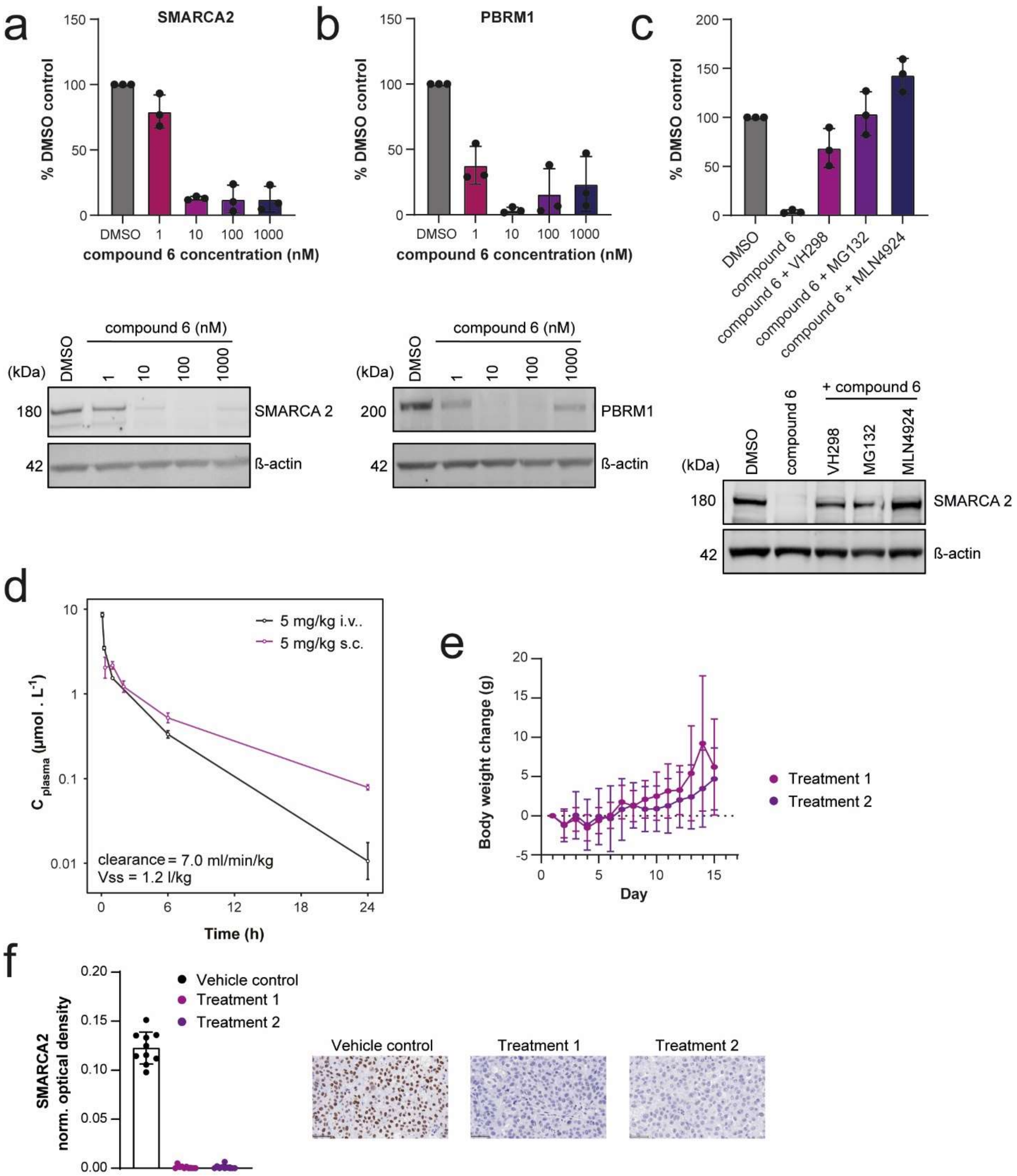

Supplementary Figure 3. Quantification of cellular and *in vivo* target protein degradation by compound 6. a. Quantified immunoblot of SMARCA2 degradation in NCI-H1568 cells treated with indicated concentrations of compound **6** for 4 h (shades of purple vs. DMSO control in gray). Data was obtained from n = 3 independent experiments, one representative blot is shown. Source data are provided as a Source Data file. **b.** As **a** for PBRM1. Source data are provided as a Source Data file. **c.** Quantified immunoblot of SMARCA2 degradation in NCI-H1568 cells treated with 100 nM compound **6** alone and in

combination with 10 µM VH298, MLN4924 and MG132 for 4 h (shades of purple vs. DMSO control in gray). Data was obtained from n = 3 independent experiments, one representative blot is shown. Source data are provided as a Source Data file. **d.** Plasma profiles of compound **6** in mouse after administration of 5 mg/kg i.v. (black) or 5 mg/kg s.c. (purple). Displayed are mean and standard deviation of n = 3 animals. Source data are provided as a Source Data file. **e**. NCI-H1568 tumour bearing mice were treated subcutaneously with 5 mg/kg compound **6** with two different treatment schedules (Treatment 1/2, see methods for details, different shades of purple). Body weight was measured daily. Displayed are mean and standard deviation of change per day for n = 10 animals. Source data are provided as a Source Data file. **f**. Tumours from **e** were collected at the end of the study and SMARCA2 levels in viable tumour tissue were determined using DAB-based IHC staining (representative images are shown, scale bar = 50 µm). Each datapoint represents the background-normalised DAB optical density (OD) within the viable tumour area of one tumour section, corresponding to an individual tumour. Mean OD levels and standard deviations are indicated in the graphs. In most cases, SMARCA2 levels in tumours from treated animals (shades of purple vs. vehicle control in black) were below the limit of detection. Source data are provided as a Source Data file.

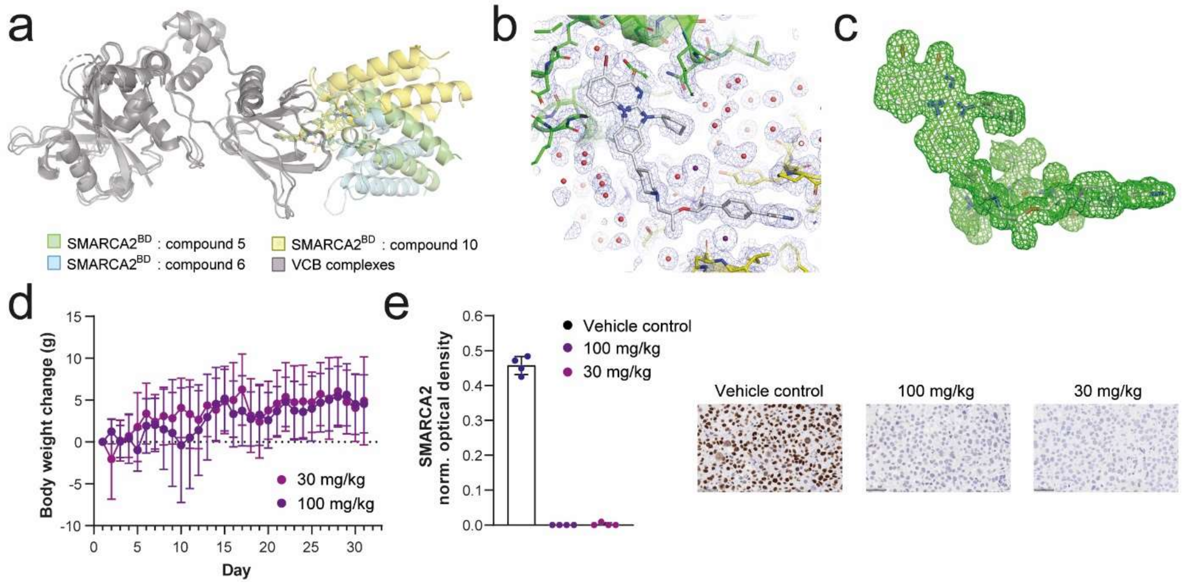

Supplementary Figure 4. Tolerability and biomarker modulation of compound 11 after oral administration. a. Superposition of ternary PROTAC complexes showing close overlap of VCB complexes (gray) and different orientations for SMARCA2^{BD} with compound **10** (yellow), compound **5** (green) and compound **6** (blue). PDB IDs: 7Z76, 7Z6L, 7Z77. **b.** Representative 2Fo-Fc electron density for compound **10** co-crystal structure at 1 σ contour level. Green: SMARCA2, Yellow: VHL. **c.** Fo-Fc omit map for compound **10** co-crystal structure at 3 σ contour level.**d.** NCI-H1568 tumour bearing mice were treated orally with compound **11** at 30 or 100 mg/kg daily (shades of purple). Average tumour volume at the beginning of treatment was ~220 mm^3. Body weight was measured daily. Displayed are mean and standard deviation of change per day for n = 10 animals. Source data are provided as a Source Data file. **e**. Tumours from **d** were collected at the end of the study and SMARCA2 levels in viable tumour tissue from four animals per group were determined using DAB-based IHC staining (representative images are shown, scale bar = 50 µm). Each datapoint represents the background-normalised DAB optical density (OD) within the viable tumour area of one tumour section, corresponding to an individual tumour. Mean OD levels and standard deviations are indicated in the graphs. In most cases, SMARCA2 levels in tumours from treated animals (shades of purple vs. vehicle control in black) were below the limit of detection. Source data are provided as a Source Data file.

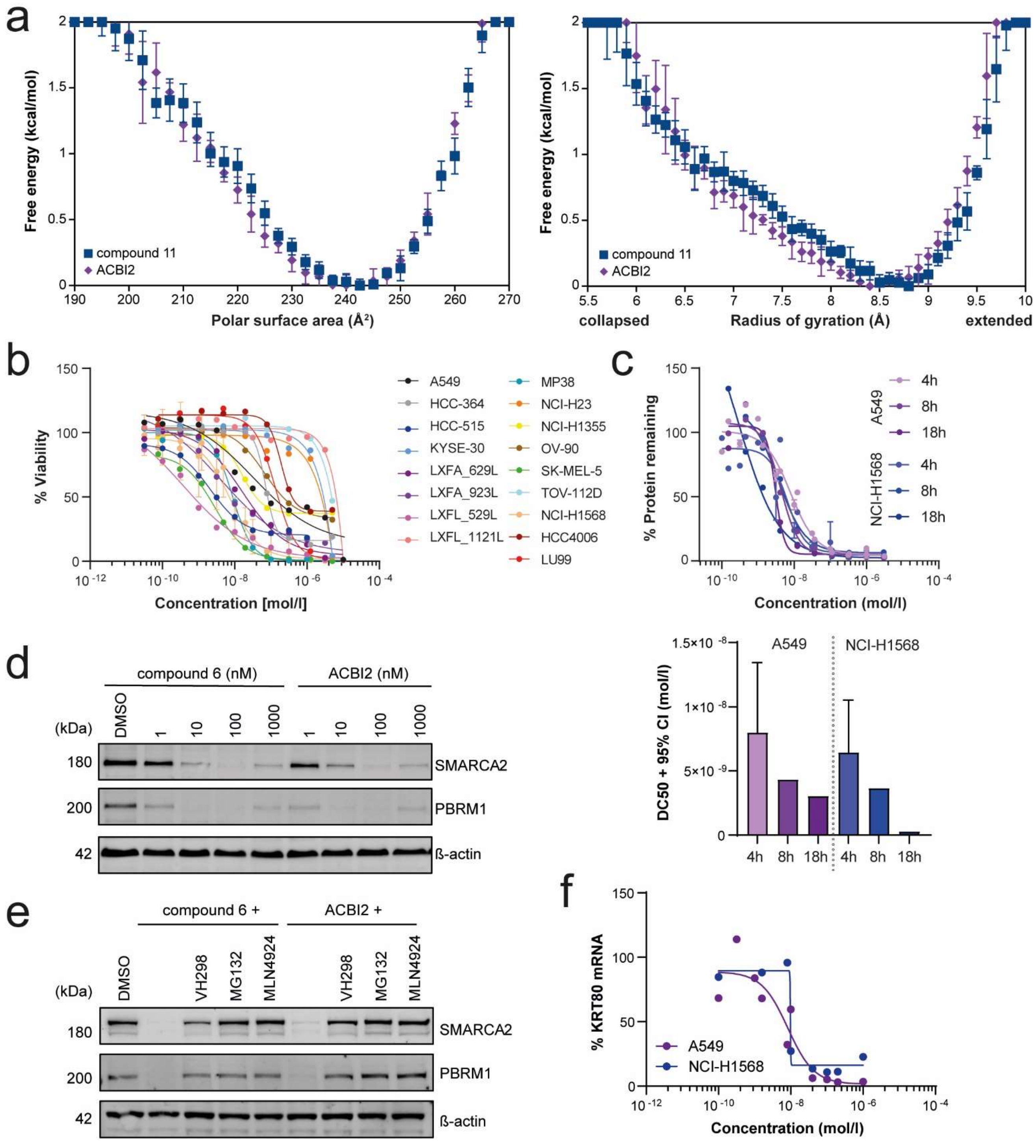

Supplementary Figure 5. Characterisation of ACBI2 by molecular dynamics, cellular degradation, qPCR assays and unbiased proteomics. a. Free energy profiles (mean with standard deviation based on n = 5 trajectory splits) from MD simulations for ACBI2 (purple diamonds) compared to compound **11** (blue squares). Source data providing first and last trajectory snapshot for compound 11 and first and last trajectory snapshot for ACBI2 are provided as a Source Data file. **b**. The indicated cell lines were treated

with ACBI2 for 144-192 h, and cell viability was measured using CellTiter Glo (n = 2-7 independent experiments: n = 2 for A549, HCC-364, HCC-515, HCC4006, KYSE-30, LU99, LXFA_629L, LXFA_923L, LXFL_529L, LXFL_1121L, MP38, NCI-H23, NCI-H1355, OV-90, SK-MEL-5, TOV-112D; n = 7 for NCI-H1568; different colours according to legend). Displayed are mean values with standard deviation for n > 2 and 4-parametric logistic curve fits. Source data are provided as a Source Data file. **c.** A549 (shades of purple) and NCI-H1568 cells (shades of blue) were treated with ACBI2 at the indicated concentrations for the indicated times, and remaining SMARCA2 protein levels were determined by capillary electrophoresis relative to DMSO control. Displayed are means of n = 1-3 independent experiments (n = 3 for A549 4h and NCI-H1568 4h, n = 2 for A549 8h, n = 1 for A549 18h, NCI-H1568 8h and NCI-H1568 18h), error bars for n > 2 indicate standard deviation. Lower panel shows summary DC_{50} values (=centre of error bar) with 95% confidence interval (error bars) from 4-parametric logistic curve fit over all biological replicates (i.e. individual data points cannot be displayed). Source data are provided as a Source Data file. **d.** Immunoblot of SMARCA2 and PBRM1 degradation in NCI-H1568 cells treated with indicated concentrations of compound **6** and ACBI2 for 4 h. Immunoblot is a representative of n = 3 independent experiments. Source data are provided as a Source Data file. **e.** Immunoblot of SMARCA2 and PBRM1 degradation in NCI-H1568 cells treated with 100 nM compound **6** and ACBI2 alone and in combination (indicated by +) with 10 µM VH298, MLN4924 and MG132 for 4 h. Immunoblot is a representative of n = 3 independent experiments. Source data are provided as a Source Data file. **f.** qPCR for *KRT80* mRNA levels (normalised to *GAPDH* housekeeping gene) after 18 h ACBI2 treatment at the indicated concentrations in A549 (purple) and NCI-H1568 cells (blue). Means of n = 2 independent experiments are displayed relative to the DMSO control. Source data are provided as a Source Data file.

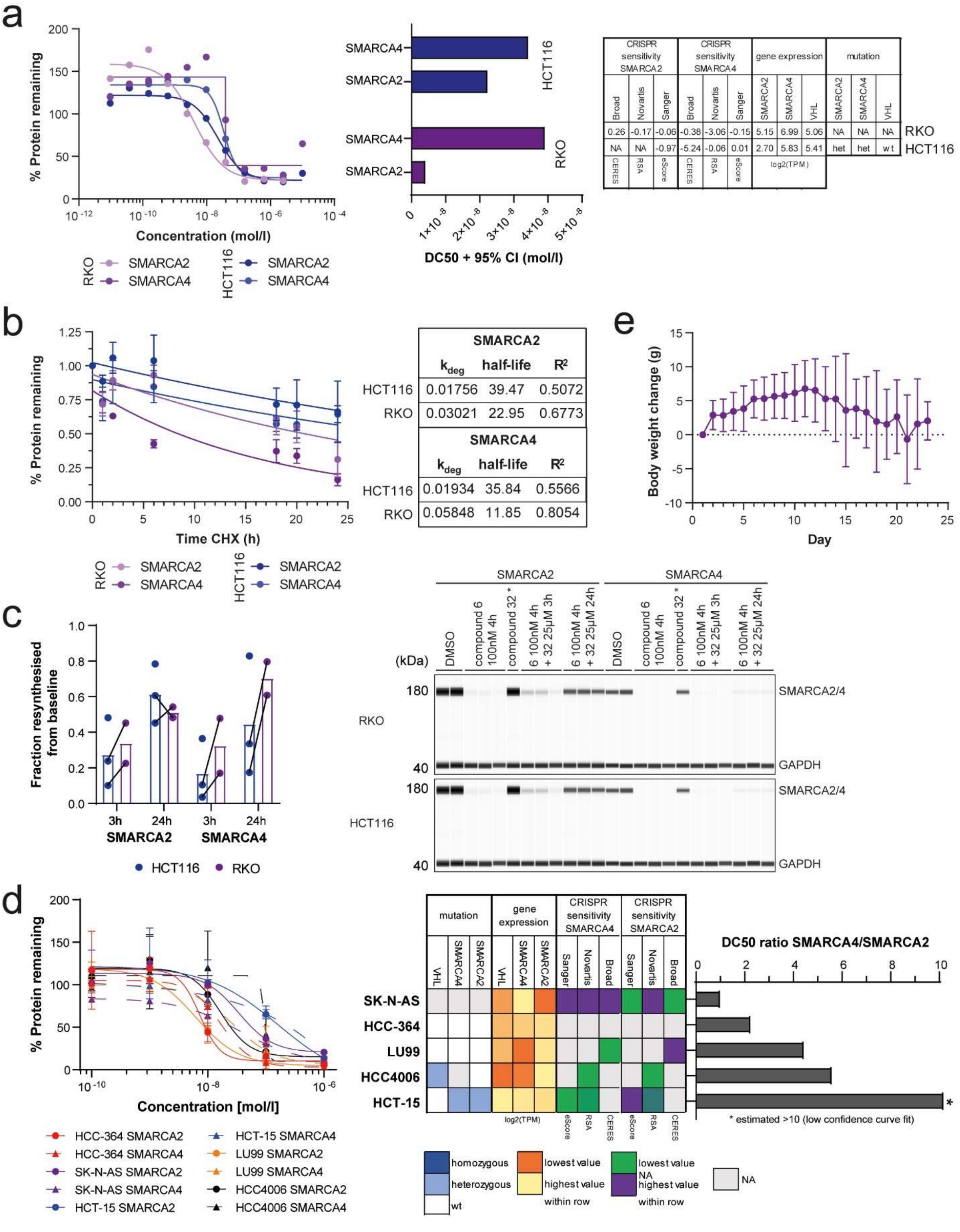

Supplementary Figure 6. Characterisation of ACBI2 in SMARCA2/4 expressing cancer cell lines and *in vivo* tolerability. a. RKO (shades of purple) and HCT116 cells (shades of blue) were treated with ACBI2 at

the indicated concentrations for 18 h, and remaining SMARCA2/4 protein levels were determined by capillary electrophoresis relative to DMSO control. Displayed are means of n = 2 independent experiments. Right panel shows DC_{50} values with 95% confidence interval from 4-parametric logistic curve fit. Table provides sensitivity to genetic depletion by CRISPR, gene expression and mutation data from DepMap/CCLE. Source data are provided as a Source Data file. **b.** SMARCA2/4 half-life after cycloheximide treatment for the indicated duration in HCT116 and RKO cells. Colours as in **a**. Protein levels were determined by capillary electrophoresis and were displayed relative to respective DMSO controls (means of n = 3 independent experiments, error bars indicate standard deviation). Table shows curve fit parameters (degradation rate, half-life and goodness of fit) from one-phase exponential decay. Source data are provided as a Source Data file. **c.** SMARCA2/4 re-synthesis after degradation with 100 nM compound **6** for 4 h (= baseline) and blocking with a VHL inhibitor (compound **32**, 25-50 µM) for 3 or 24 h. Baseline values were subtracted from DMSO control values and set to 1. 3 and 24 h time points are displayed as fractions of this. n = 2-3 independent experiments (n = 3 for HCT116, n = 2 for RKO, colours as in **a**); one representative experiment is shown. Source data are provided as a Source Data file. **d.** As in **a** for SMARCA2/4 in the indicated cell lines after ACBI2 treatment, error bars for n > 2 indicate standard deviaton. Bars (right panel) display ratios of SMARCA4 over SMARCA2 DC_{50} values obtained from 4-parametric logistic curve fits of n = 2-5 independent experiments (left panel; n = 5 for LU99, n = 3 for HCC-364, HCT-15 and HCC4006, n = 2 for SK-N-AS, colours according to legend). Heatmap (middle panel) provides sensitivity to genetic depletion by CRISPR, gene expression and mutation data from DepMap/CCLE, colours are scaled for each column according to legend, i.e., across cell lines, but separate for each parameter. Source data are provided as a Source Data file. **e.** A549 tumour bearing mice were treated orally with 80 mg/kg ACBI2 once daily. Average tumour volume at the start of treatment was ~220 mm^3. Body weight was measured daily. Displayed are mean and standard deviation of change per day for n = 10 animals. Source data are provided as a Source Data file.

Crystallography data collection and refinement statistics

Supplementary Table 1: Data collection and refinement statistics for Compound 4 : SMARCA2^{BD} co-crystal structure

	Compound 4 (PDB 7Z78)
**Data collection**	
Space group	$P3_1$
Cell dimensions	
a, *b*, *c* (Å)	61.06, 61.06, 89.28
α, β, γ (°)	90.0, 90.0, 120
Resolution (Å)	89.275-1.324 (1.347-1.324)*
R_{merge}	0.045 (0.845)
$I / \sigma I$	16.3 (1.5)
Completeness (%)	94.0 (21.6)
Redundancy	5.1 (4.8)
**Refinement**	
Resolution (Å)	1.32
No. reflections	60971
R_{work} / R_{free}	18.7 / 21.0
No. atoms	
Protein	2771
Ligand/ion	94
Water	427
B-factors	
Protein	27.85
Ligand/ion	24.43
Water	38.51
R.m.s. deviations	
Bond lengths (Å)	0.008
Bond angles (°)	0.85

*Values in parentheses are for highest-resolution shell.

Supplementary Table 2: Data collection and refinement statistics for VCB : Compound 5 : SMARCA2^{BD} ternary complex co-crystal structure

	Compound 5 (7Z6L)
**Data collection**	
Space group	$P\ 2_1$
Cell dimensions	
a, b, c (Å)	47.3, 86.8, 59.3
α, β, γ (°)	90, 98.9, 90
Resolution (Å)[a]	48.5 - 2.25 (2.33 - 2.25)
R_{merge} [a]	0.123 (0.876)
R_{meas} [a]	0.134 (0.964)
$I/\sigma(I)$ [a]	10.41 (1.98)
$CC_{1/2}$ [a]	0.996 (0.663)
Completeness (%)[a]	99.3 (95.1)
Redundancy [a]	6.5 (5.9)
**Refinement**	
Resolution (Å)	2.25
No. unique reflections [a]	22539 (2132)
R_{work} / R_{free}	0.1960 / 0.2350
Reflections used for R-free	1126 (106)
No. atoms	
Protein	3600
Ligand	70
Water	64
Wilson *B*-factors (Å^2)	41.8
Average *B*-factors (Å^2)	
Protein	52.8
Ligand	43.0
Water	47.1
R.m.s deviations from ideal	

Bond lengths (Å)	0.014
Bond angles (°)	1.68
Ramachandran plot	
Favored (%)	97.99
Allowed (%)	2.01
Outliers (%)	0

Supplementary Table 3: Data collection and refinement statistics for VCB : Compound 6 : SMARCA2^{BD} ternary complex co-crystal structure

	Compound 6 (PDB: 7Z77)
**Data collection**	
Space group	$P2_1$
Cell dimensions	
a, *b*, *c* (Å)	48.90, 63.71, 91.96
α, β, γ (°)	90.0, 101.32, 90.0
Resolution (Å)	90.167-1.974 (2.231-1.974)*
R_{sym} or R_{merge}	0.073 (0.991)
$I / \sigma I$	12.3 (1.6)
Completeness (%)	87.7 (59.1)
Redundancy	5.0 (4.4)
**Refinement**	
Resolution (Å)	1.97
No. reflections	29493
R_{work} / R_{free}	21.3 / 25.1
No. atoms	
Protein	3706
Ligand/ion	69
Water	100
B-factors	
Protein	55.39
Ligand/ion	46.98
Water	51.49
R.m.s. deviations	
Bond lengths (Å)	0.009
Bond angles (°)	0.98

*Values in parentheses are for highest-resolution shell.

Supplementary Table 4: Data collection and refinement statistics for VCB : Compound 10 : SMARCA2^{BD} ternary complex co-crystal structure.

	Compound 10 (PDB 7Z76)
**Data collection**	
Space group	$P2_1$
Cell dimensions	
a, b, c (Å)	47.47, 101.40, 69.13
α, β, γ (°)	90.0, 105.56, 90.0
Resolution (Å)	66.597-1.326 (1.450-1.326)*
R_{sym} or R_{merge}	0.052 (0.924)
I / σI	12.1 (1.5)
Completeness (%)	92.0 (50.5)
Redundancy	4.5 (4.0)
**Refinement**	
Resolution (Å)	1.32
No. reflections	104311
R_{work} / R_{free}	17.8 / 20.2
No. atoms	
Protein	3632
Ligand/ion	80
Water	672
B-factors	
Protein	25.08
Ligand/ion	16.90
Water	39.43
R.m.s. deviations	
Bond lengths (Å)	0.008
Bond angles (°)	0.95

*

*Values in parentheses are for highest-resolution shell.

Supplementary Methods

NMR experimental procedures for compound 11 and ACBI2

NOE experiments on compound **11** and **ACBI2** were performed on Bruker 400 MHz and 600 MHz NMR spectrometers equipped with cryoprobes. T1 relaxation times of **11** and **ACBI2** in CDCl3 were acquired with a d1 relaxation delay of 10 seconds, 32 scans per increment and the pulseprogram t1ir, data were analyzed with the t1guide routine within Bruker Topspin version 3.6. Relaxation rates of respective resonances of **11** and **ACBI2** were very similar indicating that no systematic errors when analyzing the NOE data are to be expected. The pulseprogram noesygpphpp was used with a mixing time of 700 ms and 1024 and between 256 and 512 datapoint acquired in the direct and indirect dimensions. We acquired spectra in both DMSO-d_6 and $CDCl_3$-*d* in order to determine if there are major conformational changes when changing the solvent from polar to apolar. Some very weak long-range NOEs of **ACBI2** were indeed only detectable in $CDCl_3$-*d,* like the one from the t-butyl group to the H27, 29 protons of the piperidine ring, indicating a further compacting when moving from DMSO to $CDCl_3$-*d.* To provide further evidence that these long-range NOEs observed in **ACBI2** are valid, we recorded NOESYs with mixing time s of 0.1 s, 0.2 s, 0.4 s, 0.6 s, 0.8 s and 1.0 s, thereby determining the build-up curves of the cross peaks as shown in Supplementary Fig. 9. Here diagonal and cross peaks were integrated with Topspin (average volume) and the regions saved and transferred to the respective NOESY spectra which were all recorded with identical parameters such as increments, number of scans and gain. Overall we observed almost identical NOE crosspeaks and very similar chemical shifts of the matched pair aside from the OH-51 and NH-59 shifts. These were the only chemical shifts of greater than 0.5 ppm upfield when moving from DMSO to $CDCl_3$-*d* on the 1H scale. This can be attributed to the loss of the strong hydrogen bond acceptor properties of DMSO for the more acidic protons when moving to $CDCl_3$-*d*. This also indicates that OH-51 and NH-59 (Supplementary Table 5) are not involved in intramolecular hydrogen bonds, because the shifts would be much smaller otherwise. To the contrary NH-37 has only small upfield shift of <0.2 ppm, indicating a weak intramolecular hydrogen bond.

Both **11** and **ACBI2** have molecular weights of roughly 1050 g/mol which puts them in a range where the NOE is close to zero depending on the solvent's viscosity, temperature, NMR spectrometer frequency an d the shape of the molecule to be studied.[1] Interestingly, **11** has mostly negative NOEs in DMSO both at 400 MHz and 600 MHz, but mostly negative and very close to zero NOEs in $CDCl_3$-*d* (Supplementary Fig. 7). On the other hand, **ACBI2** has negative NOEs in DMSO, but mostly positive NOEs in $CDCl_3$-*d*, providing further evidence that the overall shape of **ACBI2** seems to be more compact compared to **11**. The more compact shape in turn is reflected in a higher molecular tumbling rate as evidenced in the positive NOE of **ACBI2** in $CDCl_3$-*d*. Due to **11** and **ACBI2** being close to the zero crossing of the NOE, we recorded 1D selective ROESY experiments to avoid missing NOEs. Here the pulseprogram selrogp was used with a spinlock time of 200 msec. Shown in Supplementary Fig. 10 below are the 1D spectra obtained when inverting the t-butyl group, showing the very similar ROE patterns except for the very small ROE observed to the pyrimidine ring. Due to the different solubilities of **11** and **ACBI2** in $CDCl_3$-*d* we had to use 8k scans with **11** compared to only 256 scans with **ACBI2** to achieve the same signal to noise ratio, determined on proton 37 in both compounds. We had difficulty to complete the assignment of the complete linker region of **11** due to exchange line broadening and overlap in both DMSO and $CDCl_3$-*d*, as can be observed in the missing assignments in Supplementary Table 5. While this makes a direct comparison of NOE patterns difficult, it is a further indication on the higher degree of flexibility of **11** compared to **ACBI2**.

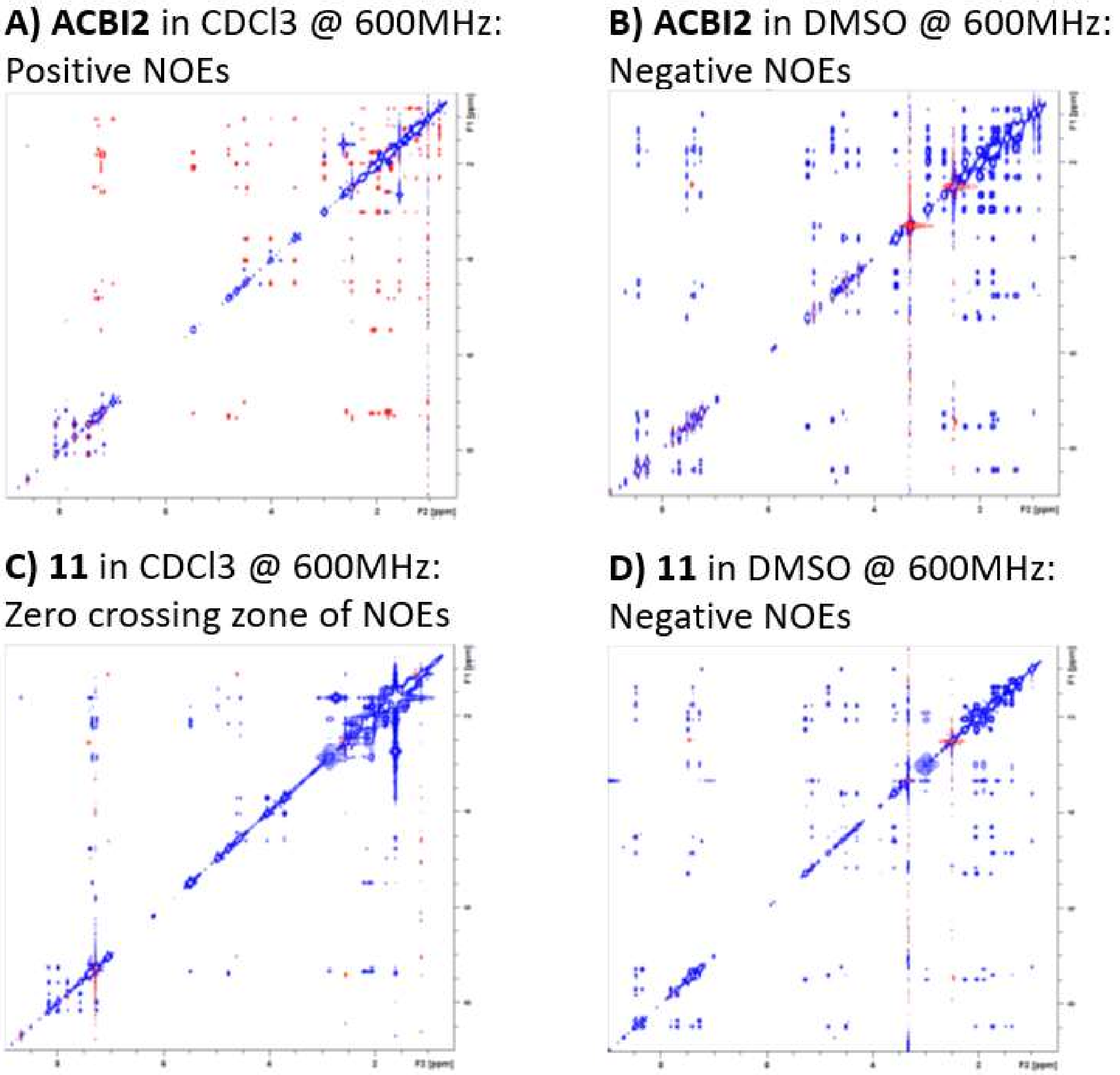

Supplementary Figure 7: NOESY spectra of **ACBI2** (A and B) and compound **11** (C and D) in $CDCl_3$-*d* and DMSO-d_6, that show that the more compact structure of ACBI2 has a marked influence on the sign of the NOE (see text)

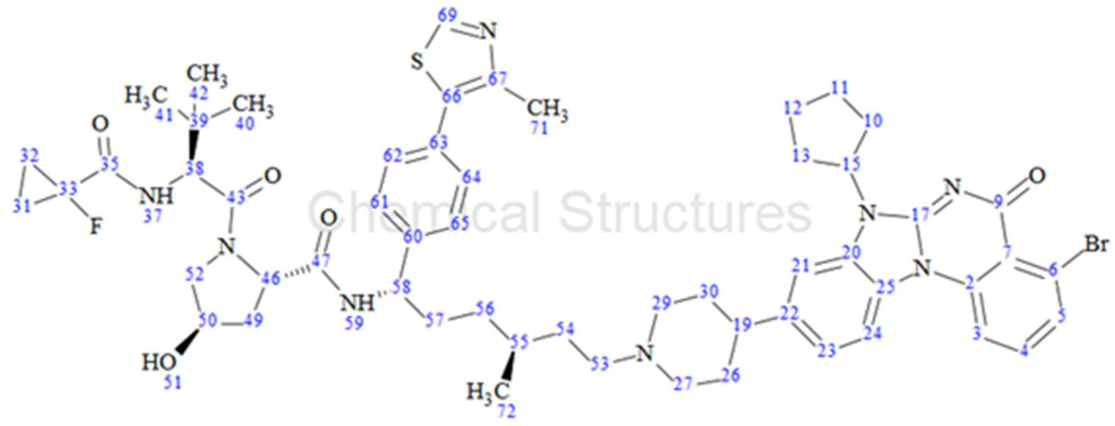

Supplementary Figure 8: Numbering of **ACBI2** (and compound **11** w/o methyl group 72)

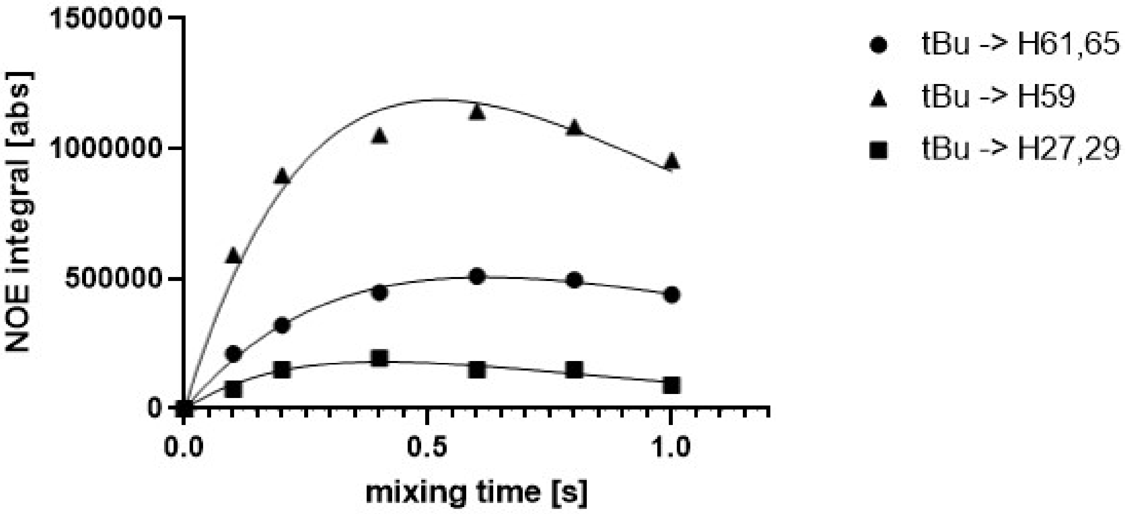

Supplementary Figure 9: NOE build-up curves for selected cross peaks of **ACBI2** in $CDCl_3$-*d*. Data shown is for the three long range NOEs displayed in Figure 4b of the main paper. Build up curves were fit according to the isolated spin pair approximation by integrating both diagonal and cross peak intensities as described by Neuhaus and Williamson[5]

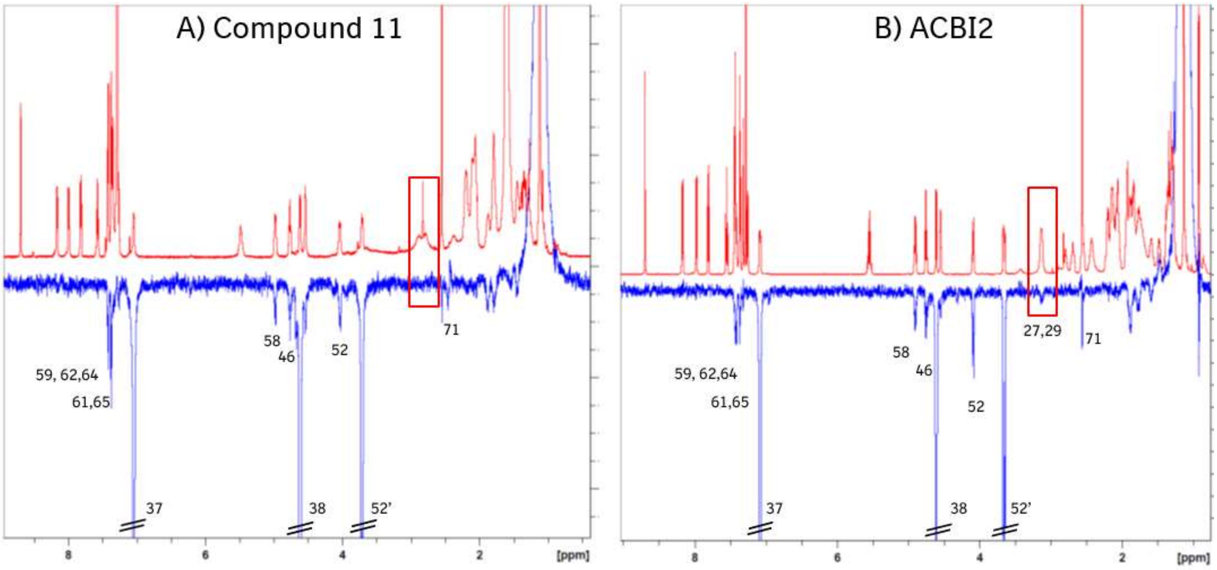

Supplementary Figure 10: ^{1}H NMR of **11** (A) and **ACBI2** (B) shown in red, and inverted in blue the selective 1D ROESY traces measured in $CDCl_3$-*d*. The t-butyl group (41, 42, 43) was selectively inverted with a gaussian pulse. Overall ROE patterns and intensities are similar. Only in ACBI2 a weak intensity ROE was detected to the piperidine 27, 29 protons. Interestingly this ROE as well as those of the t-butyl group to proton 71 on the thiazole were not detected in DMSO (not shown) as a solvent indicating that these compounds adopt slightly different conformations in $CDCl_3$-*d* and DMSO-*d6*.

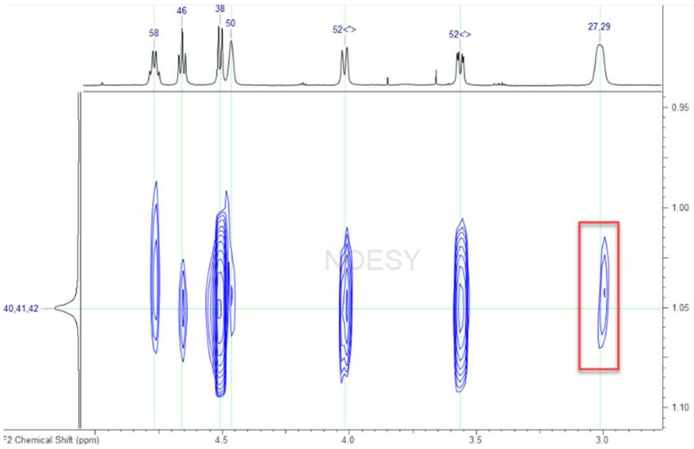

Supplementary Figure 11: Section of NOESY spectrum for compound **30** which has a geminal dimethyl group in the linker in $CDCl_3$-*d* indicating a similar trend regarding the long range NOEs as ACBI2 with the NOE from the t-butyl group to the protons 27,29 of the piperidine.

Supplementary Table 5: Chemical shifts of **ACBI2** in DMSO-d_6 and $CDCl_3$-*d* and compound **11** in DMSO-d_6, n/a means the assignment was not unequivocally possible due to overlap. For geminal protons that are left blank the chemical shift separation was too small to distinguish them. **11** was not sufficiently soluble in $CDCl_3$-*d* to be able to assign all atoms which is why it was omitted from the table.

	ACBI2 in DMSO-d_6		ACBI2 in CDCl3-*d*		Compound 11 in DMSO-d_6	
Atom Number	H Shift [ppm]	C Shift [ppm]	H Shift [ppm]	C Shift [ppm]	H Shift [ppm]	C Shift [ppm]
2		139.3		139		139.2
3	8.47	115.8	8.14	113.8	8.48	115.8
4	7.68	133.8	7.52	132.3	7.69	133.8
5	7.8	132.6	7.78	132.8	7.82	132.7
6		123.3		124.9		123.3
7		117		117.7		117.1
9		164.6		165.8		164.6
10, 13	2.29	28.4	2.17	28.4	2.05	28.5
10', 13'	2.01	28.4	2.13	28.4	2.26	28.5
11, 12	2.02	25	2.03	25	1.77	25.1
11', 12'	1.74	25	1.81	25	2.02	25.1
15	5.26	54.4	5.54	54.1	5.27	54.4
17		149.3		149.5		149.4
19	2.68	42.5	2.65	42.9	n/a	n/a
20		131		130.3		130.8
21	7.54	109.6	7.29	109.6	7.48	109.6
22		144.1		143.7		142.2
23	7.27	121.7	7.23	121.5	7.27	121.3
24	8.29	114	7.95	113.2	8.35	114.3
25		125.3		125.6		125.7
26, 30	1.74	33.9	1.9	33.9	2.08	31.1
26', 30'	1.79	33.9	1.84	33.9	1.9	31.1
27, 29	1.98	54.6	2.04	54.5	n/a	n/a
27', 29'	2.99	54.6	3.07	54.5	3.01	n/a
31, 32	1.22	13.3	1.31	13.8	1.23	13.3
31', 32'	1.36	13.3			1.36	13.3
33		78.7		78.3		78.7
35		168.4		170.6		168.4
37	7.24		7.06		7.23	
38	4.58	57	4.58	57.7	4.59	57
39		36.6		35.1		36.6
40, 41, 42	0.98	26.7	1.11	26.6	0.99	26.7
43		169.1		171.6		169.2
46	4.51	59.1	4.73	58.5	4.51	59.1
47		171.2		169.6		171.2
49	2.05	38.2	2.04	35.4	1.75	38.2
49'	1.76	38.2	2.55	35.4	2.05	38.2
50	4.3	69.3	4.53	70.1	4.3	69.3
51	5.14		2.71		5.15	
52	3.59	57.1	4.08	56.6	3.6	57.2
52'			3.63	56.6		
53	2.3	56.6	2.35	57.1	n/a	n/a
54	1.5	34.1	1.54	34	n/a	n/a
54'	1.26	34.1	1.32	34	n/a	n/a
55	1.49	30.9	1.44	31.7	n/a	n/a
56	1.32	33.4	1.25	33.5	1.37	26
56'					1.49	26
57	1.75	34.4	1.85	34.1	1.73	36.6
57'	1.66	34.4	1.76	34.1	n/a	n/a
58	4.79	52.9	4.88	54.1	4.84	52.3
59	8.45		7.39		8.48	
60		144.4		142.1		144.3
61, 65	7.39	127.3	7.34	126.9	7.4	127.3
62, 64	7.45	129.3	7.41	129.6	7.46	129.3
63		130.2		131		130.3
66		131.6		131.6		131.5
67		148.2		148.5		148.3
69	8.98	151.9	8.67	150.3	9	152.3
71	2.46	16.5	2.54	16.2	2.47	16.5
72	0.89	20	0.9	19.6	Not present in molecule	

Supplementary Table 6: T1 relaxation times of selected well resolved signals of **11** and **ACBI2** in $CDCl_3$-*d*

	In CDCl3 at 25°C	
Atom Number	**ACBI2 T1 [s]**	**11 T1 [s]**
69	>5	>5
3	1.25	1.09
24	1.28	1.08
5	3.63	2.84
4	2.21	1.88
62, 64	1.62	2.04
23	1.57	1.44
37	0.88	0.74
15	1.33	1.26
58	1.19	1.07
46	1.42	1.18
38	0.81	0.77
50	1.33	1.16
52''	0.53	0.52
52'	0.64	0.55
27/29	0.56	0.64
40,41,42	0.42	0.40

Chemical Synthesis

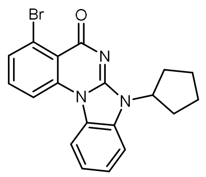

Compound 1

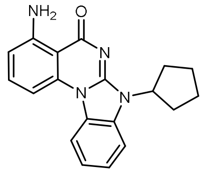

Compound 2

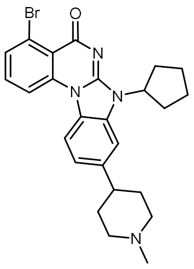

Compound 3

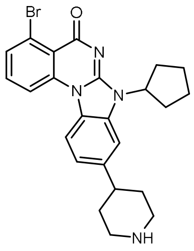

Compound 4

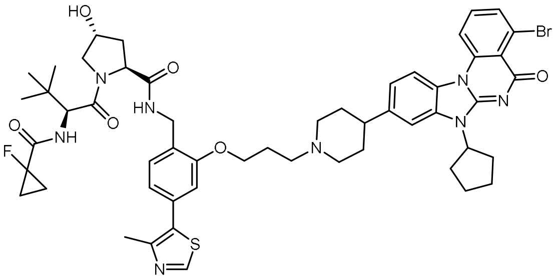

Compound 5

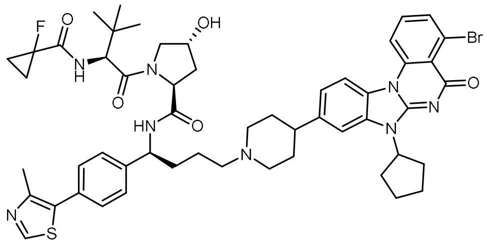

Compound 6

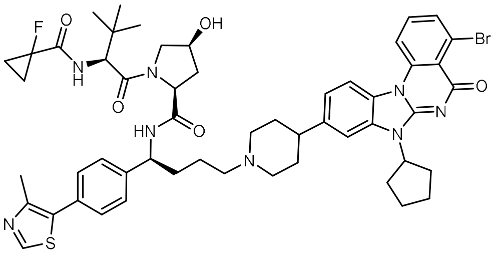

Compound 7
(cis-6)

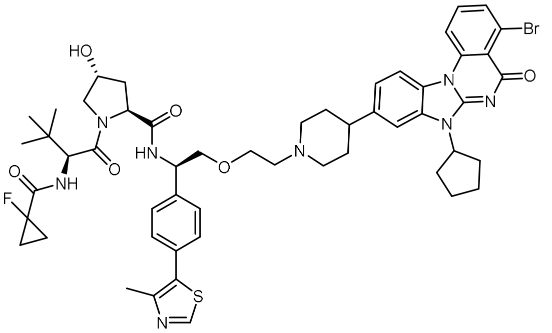

Compound 8

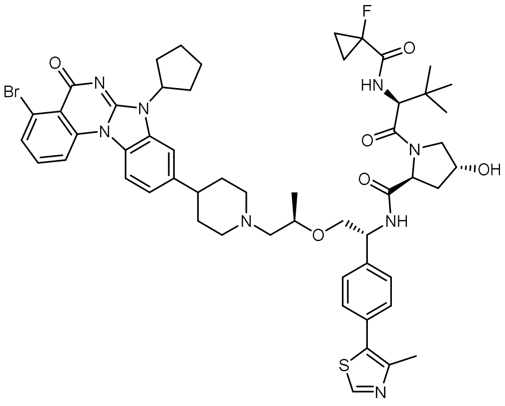

Compound 9

Supplementary Figure 12: List of final compound structures

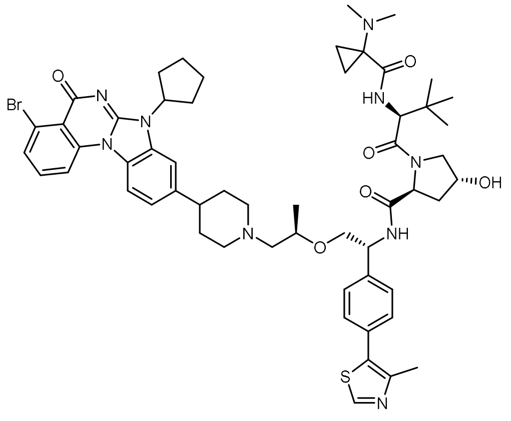

Compound 10

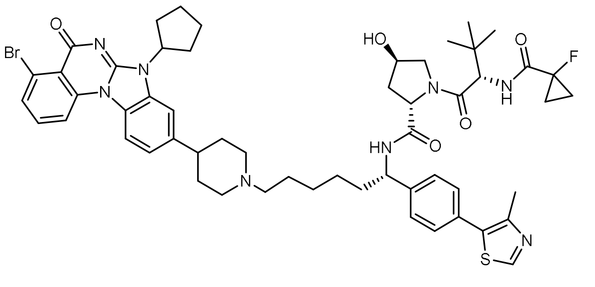

Compound 11

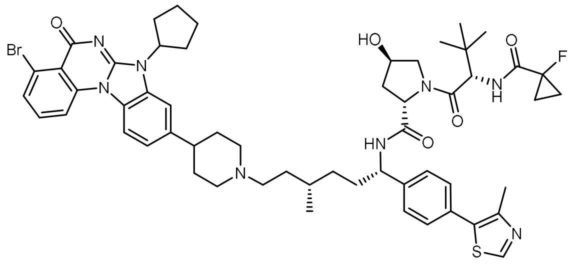

ACBI2

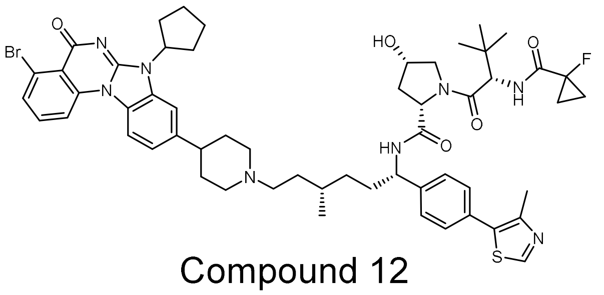

Compound 12

(cis-ACBI2)

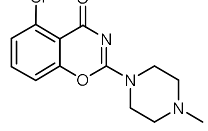

Compound 13

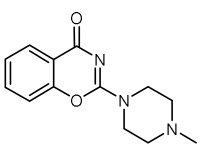

Compound 14

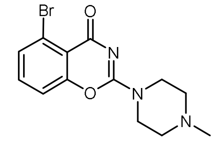

Compound 15

Supplementary Figure 12: List of final compound structures (continued)

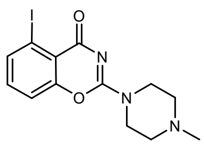

Compound 16

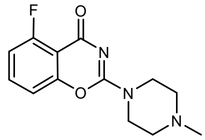

Compound 17

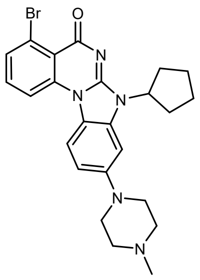

Compound 18

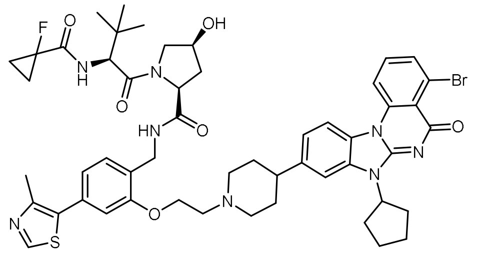

Compound 19 (cis-20)

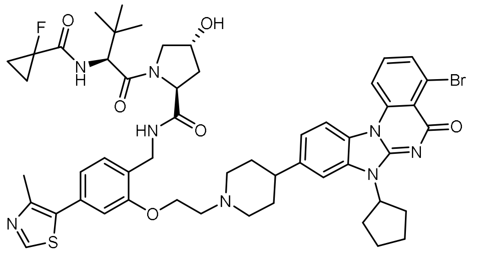

Compound 20

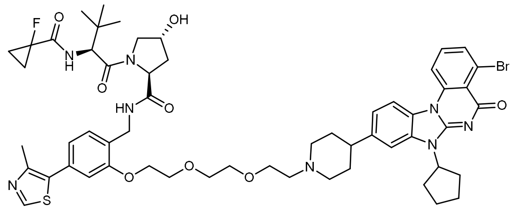

Compound 21

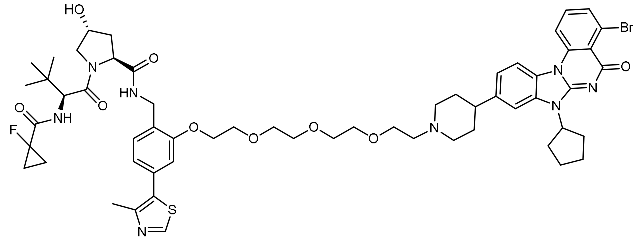

Compound 22

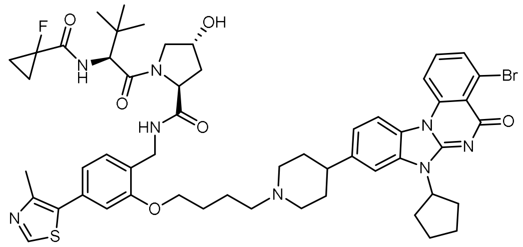

Compound 23

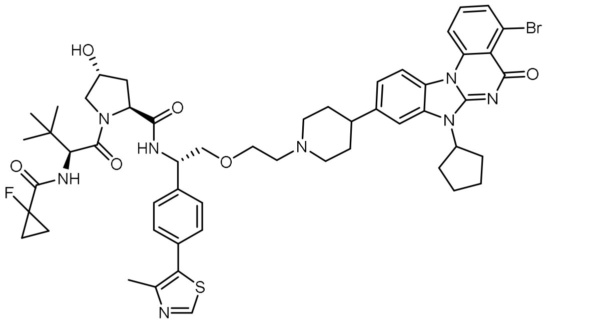

Compound 24

Supplementary Figure 12: List of final compound structures (continued)

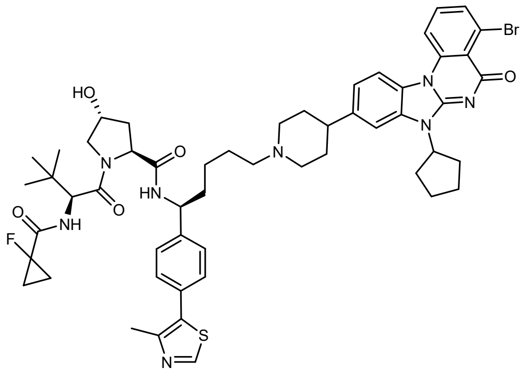

Compound 25

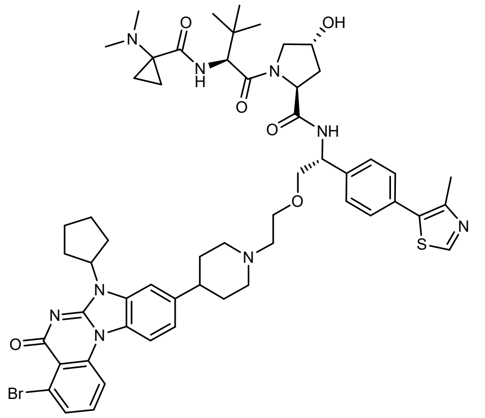

Compound 26

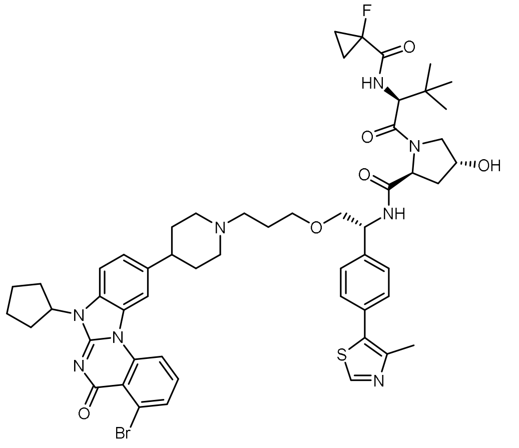

Compound 27

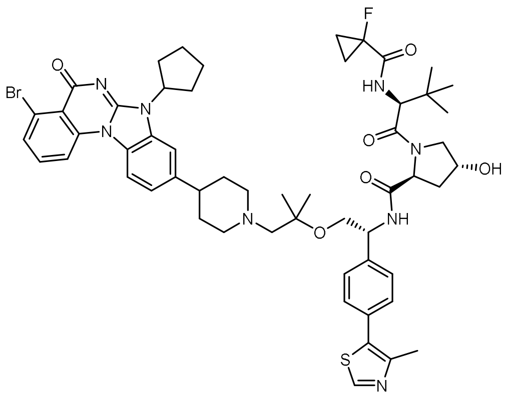

Compound 28

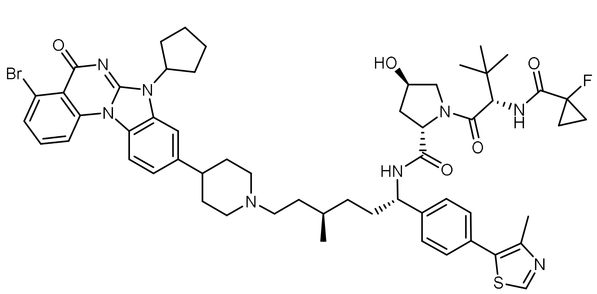

Compound 29

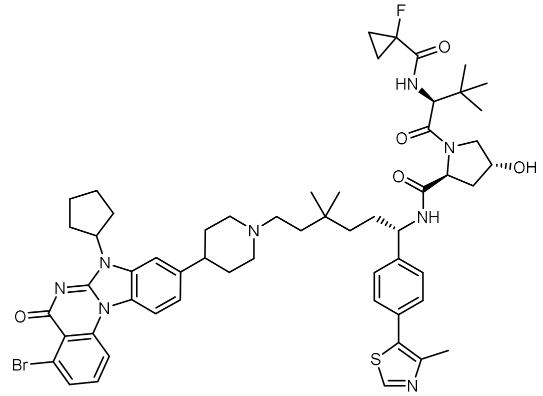

Compound 30

Supplementary Figure 12: List of final compound structures (continued)

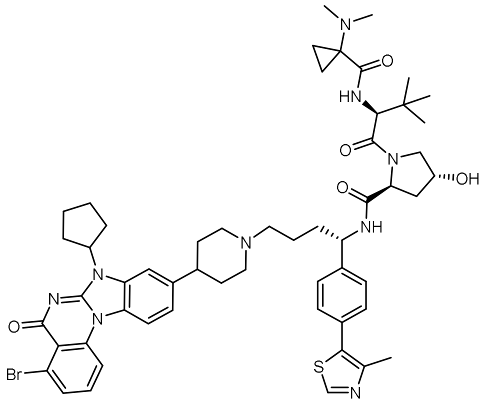

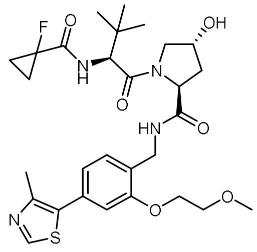

Compound 31 Compound 32

Supplementary Figure 12: List of final compound structures (continued)

General information

Commercially available dry solvents were used from Sigma Aldrich. All reagents unless otherwise noted were commercially available and purchased from Sigma Aldrich, Combi Blocks, ABCR, Fluorochem, Activate or Enamine, at least 95% pure and used without further purification. All reactions were carried out in oven- or flame dried glassware under nitrogen atmosphere. Normal phase TLC was carried out on pre-coated silica plates (Kieselgel 60 F254, BDH) with visualization via UV light (UV 254 and/or 365 nm) and/or basic potassium permanganate solution. Isolute® phase separator columns from Biotage were used. Flash column chromatography was performed using either a Teledyne Isco Combiflash Rf or Rf200i or a Biotage Isolera One with prepacked Redisep RF normal phase disposable columns. Reverse phase chromatography was carried out using Biotage SNAP-C18 columns. Strong cation exchange (SCX) chromatography was carried out using Biotage Isolute SCX-2 columns. NMR Spectra were recorded on Bruker 400 MHz, 500 MHz or 600MHz spectrometers as specified. Chemical shifts are quoted in ppm and referenced to the residual solvent signals: ^{1}H NMR δ (ppm) = 7.26 ($CDCl_3$-*d*), ^{13}C NMR δ (ppm) = 77.2 ($CDCl_3$-*d*), ^{1}H NMR δ (ppm) = 2.50 (DMSO-d_6), ^{13}C NMR δ (ppm) = 39.5 (DMSO-d_6). Signal splitting patterns are described as singlet (s), doublet (d), triplet (t), quartet (q), quintet (quin.), multiplet (m), broad (br) or a combination thereof. Coupling constants (*J*) are measured in Hertz (Hz). Diastereomeric ratios (dr) were calculated using the ratios of NMR integrals.

Analytical MS Methods and instrumentation:
Method 1: HRMS data was recorded using a LTQ Orbitrap XL (Thermo Scientific) coupled with a Triversa Nanomate Nanospray ion source (ADVION Bioscience Inc.) The mass calibration was performed using the Pierce LTQ Velos ESI positive ion calibration solution from Thermo Scientific (Product Nr. 88323).
MS parameters: The scan window was set to 50–400 amu with a maximum injection time of 500 ms and 1 microscan. Resolution of the Orbitrap was 60000 with a mass accuracy ≤ 5 ppm. The ion mode set to positive with a capillary temperature 200 °C and voltage of 60 eV. The tube lens potential was set to 110 eV. 12 NanoESI voltage was 1.45 kV and the N_2 gas pressure set to 0.45 psi. Total sample volume was 5 µL and the acquisition time was 0.4 sec, with 10 scans of averaging per spectrum Sample dilution: 10 mM DMSO stock solution was diluted 1:200 in 50% MeOH +0.01% formic acid.

Method 2: Agilent Technologies 1200 series HPLC connected to an Agilent Technologies 6130 quadrupole LC/MS with an Agilent diode array detector. **Method 2a** was run under the following conditions: Waters Xbridge C18 column, 2.5 µm particle size, 2.1 x 20 mm. Run time 2.1 minutes, flow 1 mL/min, column temperature 60 °C and 5 µL injections. Solvent A (20mM NH_4HCO_3/NH_4OH pH 9), solvent B (MS grade acetonitrile). Start 10% B, gradient 10% - 95% B from 0.0 - 1.5 min, 95% B from 1.5 - 2.0 min, gradient 95% - 10% B from 2.0 – 2.1 min. Purity was determined via UV detection with a bandwidth of 170 nm in the range from 230-400 nm. **Method 2b** used a Waters XBridge column (50 mm × 2.1 mm, 3.5 µm particle size) and the compounds were eluted with a gradient 5–95% acetonitrile/water + 0.1% formic acid ("acidic method").

Preparative purification methods and instrumentation:
Method 3: Preparative HPLC was performed on a Gilson system with Waters C18 columns (50 mm x 150 mm; 10 µm particle size) and a gradient of 10% to 95% acetonitrile in water over 8 minutes and a flow rate of 150 mL/min, with a SunFire column and 0.1% formic acid (**Method 3a**) or a XBridge column and 0.5% ammonium bicarbonate/ammonia buffer pH 9 (**Method 3b**) in the aqueous phase.
Method 4: Preparative HPLC was performed on a Waters Prep 150 LC system with a Waters XBridge C18 column (100 mm x 19 mm; 5 µm particle size) and a gradient of 5% to 95% acetonitrile in water over 10 minutes and a flow rate of 25 mL/min, with 0.1% formic acid (**Method 4a**) or ammonia (**Method 4b**) in the aqueous phase.
Method 5: Supercritical Fluid Chromatography (SFC) was performed on a Sepiatec Prep SFC 100 instrument using the indicated conditions.

Abbreviations used: aq. for aqueous, BH_3-DMS for borane-dimethylsulfide complex, Boc for *N-tert*-butyloxycarbonyl, Boc_2O for di-*tert*-butyl dicarbonate, DCE for 1,2-dichloroethane, DCM for dichloromethane, DEA for diethylamine, DMA for *N*,*N*-dimethylacetamide, DMF for *N*,*N*-dimethylformamide, DIPEA for *N*,*N*-diisopropylethylamine, DMSO for dimethylsulfoxide, Et_3N for triethylamine, EtOAc for ethyl acetate, HATU for 1-[bis(dimethylamino)methylene]-1*H*-1,2,3-triazolo[4,5-*b*]pyridinium 3-oxideHexafluorophosphate, KOAc for potassium acetate, MeOH for methanol, MsCl for methanesulfonyl chloride, PTSA for *p*-toluene sulfonic acid monohydrate, sat. for saturated, $SOCl_2$ for thionyl chloride, THF for tetrahydrofuran.

Synthesis of bromodomain ligands

Supplementary Figure 13: Synthesis of tetracyclic quinazolinones 1 and 2

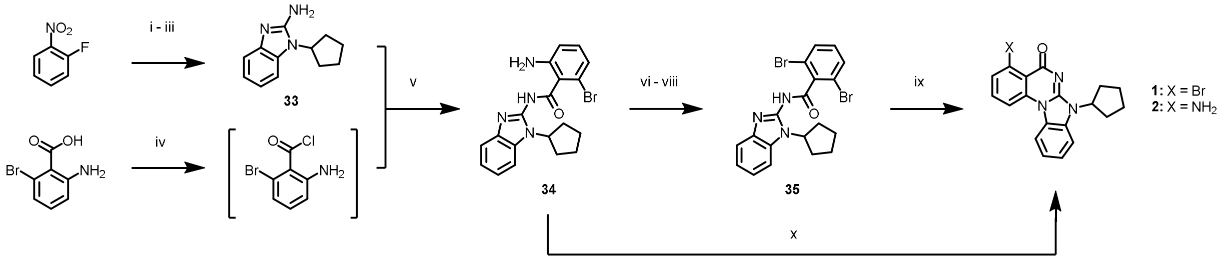

Synthesis compound 1: i) cyclopentylamine, K_2CO_3, DMF, RT ; ii) Fe, NH_4Cl, EtOH/H_2O, 80 °C ; iii) cyanogen bromide, MeCN/H_2O, 80 °C ; iv) $SOCl_2$, 60 °C ; v) Et_3N, DCM, RT ; vi) HBr, 0 °C ; vii) $NaNO_2$,H_2O ; viii) CuBr or CuI ; ix) CuI, 1,10-phenanthroline, Cs_2CO_3, DMF, 120 °C.
Synthesis compound 2: i) cyclopentylamine, K_2CO_3, DMF, RT ; ii) Fe, NH_4Cl, EtOH/H_2O, 80 °C ; iii) cyanogen bromide, MeCN/H_2O, 80 °C ; iv) $SOCl_2$, 60 °C ; v) Et_3N, DCM, RT; x) CuI, 1,10-phenanthroline, Cs_2CO_3, DMF, 120 °C.

1-cyclopentyl-1*H*-benzo[*d*]imidazol-2-amine 33

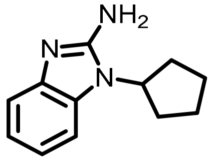

To a stirred solution of 1-fluoro-2-nitrobenzene (15.0 g, 0.11 mol) and cyclopentylamine (12.6 mL, 128 mmol) in DMF (100 mL) was added K_2CO_3 (29.4 g, 213 mmol) at RT. The reaction mixture was stirred for 16 hours at RT. Upon completion of the reaction, the reaction mixture was diluted with water and extracted with diethyl ether. The organic layer was dried over Na_2SO_4, filtered and concentrated. The crude *N*-cyclopentyl-2-nitroaniline (20.0 g) was taken to the next step without purification.

To a stirred solution of crude *N*-cyclopentyl-2-nitroaniline (10.0 g, 0.048 mol) in ethanol (100 mL) and water (50 mL), was added iron powder (13.5 g, 0.24 mol) and ammonium chloride (13.4 g, 0.24 mol) and the reaction was stirred for 16 hours at 80 °C. Upon completion of the reaction, the mixture was cooled to RT and filtered through celite. The filtrate was concentrated under reduced pressure. The remaining residue was dissolved in EtOAc and washed with water and brine solution. The organic layer was dried over Na_2SO_4, filtered and concentrated. The crude *N*-cyclopentyl-benzene-1,2-diamine (7.0 g) was taken forward to the next step without purification.

To a stirred solution of crude *N*-cyclopentyl-benzene-1,2-diamine (5.0 g, 0.028 mol) in water (100 mL) at 0 °C was added dropwise a solution of cyanogen bromide (6.0 g, 56.7 mol) in MeCN (25 mL) and the reaction mixture was stirred for 2 hours at RT. Upon completion of the reaction, the mixture was diluted with water and basified with sodium bicarbonate solution. The resultant precipitate was filtered, washed with water and dried under reduced pressure to give **33** (5.0 g, 64% yield over three steps).

^{1}H NMR (400 MHz, DMSO-*d*$_6$) δ 7.09 - 7.19 (m, 2H), 6.92 (td, J = 7.5, 1.2 Hz, 1H), 6.81 - 6.87 (m, 1H), 6.29 (s, 2H), 4.72 (quin, J = 8.7 Hz, 1H), 1.86 - 2.18 (m, 6H), 1.59 - 1.76 (m, 2H)

MS (ESI) for $C_{12}H_{15}N_3$ $[M+H]^+$ 202

2-amino-6-bromo-*N*-(1-cyclopentyl-1*H*-benzo[*d*]imidazol-2-yl)benzamide 33

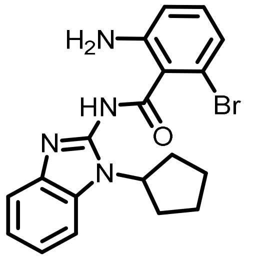

2-amino-6-bromobenzoic acid (1.2 g, 5.4 mmol) was dissolved in $SOCl_2$ (5 mL). The reaction mixture was stirred for 1 hour at 60 °C. Upon completion of the reaction, the mixture was concentrated under reduced pressure to obtain crude 2-amino-6-bromobenzoyl chloride, which was taken forward without further purification. To a solution of **33** (944 mg, 4.7 mmol) in DCM (2 mL) was added a solution of Et_3N (1.2 mL, 8.5 mmol) and 2-amino-6-bromobenzoyl chloride (1.0 g, 4.3 mmol) in DCM (2 mL). The reaction mixture was stirred for 1 hour at RT. Upon completion, the mixture was concentrated under reduced pressure and

purified by acidic reverse phase chromatography (20-98% gradient of MeCN in water) to give **34** (608 mg, 36% yield).

MS (ESI) for $C_{19}H_{19}BrN_4O$ $[M+H]^+$ 399

2,6-dibromo-*N*-(1-cyclopentyl-1*H*-benzo[*d*]imidazol-2-yl)benzamide 35

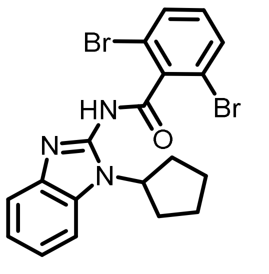

34 (392 mg, 0.98 mmol) was dissolved in concentrated HBr (48% aq., 1 mL) and cooled to 0 °C. A solution of sodium nitrite (68 mg, 0.98 mmol) in water (0.5 mL) was added dropwise. The mixture was stirred for 45 minutes at RT. Copper(I) bromide (177 mg, 1.2 mmol) was added and the reaction mixture was stirred at RT for 5 minutes. Upon completion, water (4 mL) was added and the mixture was sonicated. The precipitate was collected by filtration and dried overnight under vacuum. It was used without purification for the next step: **35** (429 mg, 92% yield).

MS (ESI) for $C_{19}H_{17}Br_2N_3O$ $[M+H]^+$ 462

4-bromo-7-cyclopentylbenzo[4,5]imidazo[1,2-*a*]quinazolin-5(7*H*)-one 1

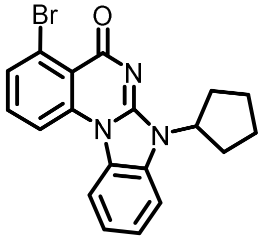

35 (300 mg, 0.65 mmol), copper(I) iodide (62 mg, 0.32 mmol), 1,10-phenanthroline (23 mg, 0.13 mmol) and Cs_2CO_3 (317 mg, 0.97 mmol) were dissolved in DMF (2 mL). The mixture was degassed with argon and stirred at 110°C overnight. Upon completion, the reaction mixture was filtered and purified by acidic reverse phase chromatography (20-98% gradient of MeCN in water) to give **1** (20 mg, 8.1% yield).

^{1}H NMR (500 MHz, DMSO-d_6) δ 8.52 (d, J = 8.5 Hz, 1H), 8.41 (d, J = 8.2 Hz, 1H), 7.82 (d, J = 7.6 Hz, 1H), 7.66 - 7.73 (m, 2H), 7.48 (t, J = 7.7 Hz, 1H), 7.39 - 7.45 (m, 1H), 5.29 (quin, J = 8.8 Hz, 1H), 2.19 - 2.32 (m, 2H), 2.04 (br d, J = 2.8 Hz, 4H), 1.70 - 1.83 (m, 2H)

^{13}C NMR (125 MHz, DMSO-d_6) δ 164.6, 149.3, 139.3, 133.8, 132.7, 130.4, 127.1, 125.2, 123.3, 123.1, 117.2, 115.9, 114.3, 111.8, 54.3, 28.5, 25.0

HRMS (m/z) for $C_{19}H_{16}BrN_3O$ $[M+H]^+$ calculated 382.05495; obtained 382.05439

4-amino-7-cyclopentylbenzo[4,5]imidazo[1,2-*a*]quinazolin-5(7*H*)-one 2

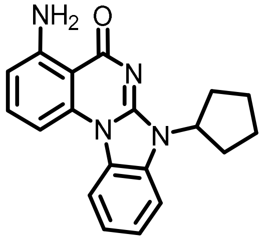

34 (125 mg, 0.31 mmol), copper(I) iodide (30 mg, 0.16 mmol), 1,10-phenanthroline (11 mg, 0.063 mmol) and Cs_2CO_3 (153 mg, 0.47 mmol) were dissolved in DMF (2 mL). The mixture was degassed with argon and stirred at 120 °C overnight. Upon completion, the reaction mixture was filtered and purified by acidic reverse phase chromatography (20-98% gradient of MeCN in water) to give **2** (28 mg, 28% yield).

^{1}H NMR (500 MHz, DMSO-*d*$_6$) δ 8.32 (d, J = 8.2 Hz, 1H), 7.67 (d, J = 8.2 Hz, 1H), 7.36 - 7.47 (m, 4H), 6.69 (dd, J = 7.6, 1.6 Hz, 1H), 5.29 (quin, J = 8.8 Hz, 1H), 2.17 - 2.30 (m, 2H), 1.97 - 2.09 (m, 4H), 1.68 - 1.80 (m, 2H)

^{13}C NMR (125 MHz, DMSO-*d*$_6$) δ 170.5, 152.6, 149.4, 138.8, 133.9, 130.3, 127.2, 124.9, 122.9, 114.1, 111.9, 111.5, 102.3, 100.5, 54.2, 28.4, 25.0

HRMS (m/z) for $C_{19}H_{18}N_4O$ $[M+H]^+$ calculated 319.15534; obtained 319.15449

Supplementary Figure 14: synthesis of Compound 3

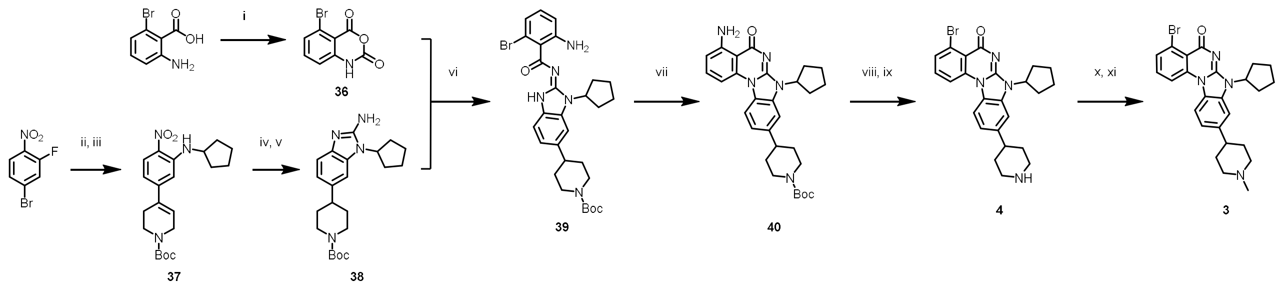

i) triphosgene, THF, -10 °C - RT; ii) cyclopentylamine, K_2CO_3, DMF, RT ; iii) *N*-Boc-1,2,3,6-tetrahydropyridine-4-boronic acid pinacol ester, Cs_2CO_3, $Pd(PPh_3)_4$, 1,4-dioxane, 100 °C ; iv) Pd/C,H_2, THF, RT ; v) cyanogen bromide, MeOH, 0 °C ; vi) 1,4-dioxane, 120 °C ; vii) CuI, 1,10-phenanthroline, Cs_2CO_3, NMP, 120 °C ; viii) PTSA, CuBr, MeCN ; ix) $NaNO_2$,H_2O ; x) CH_2O, AcOH, DMF, RT ; xi) $NaBH(OAc)_3$

5-Bromo-1*H*-benzo[*d*][1,3]oxazine-2,4-dione 36

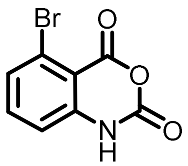

2-Amino-6-fluoro-benzoic acid (150 g, 0.69 mol) was dissolved in THF (1.5 L) and cooled to -10 °C. Triphosgene (206 g, 0.69 mol) was added. The reaction mixture was stirred overnight at RT. The reaction mixture was quenched with ice cold water. The resultant precipitate was filtered and washed with water then dried under high vacuum to obtain crude **36** (150 g, 89% yield).

^{1}H NMR (400 MHz, DMSO-d_6) δ 11.83 (s, 1H), 7.54 - 7.59 (m, 1H), 7.50 - 7.54 (m, 1H), 7.15 (dd, J = 7.9, 1.3 Hz, 1H)

MS (ESI) for $C_8H_4BrNO_3$ $[M+H]^+$ 242

***tert*-butyl 4-(3-(cyclopentylamino)-4-nitrophenyl)-5,6-dihydropyridine-1(2*H*)-carboxylate 37**

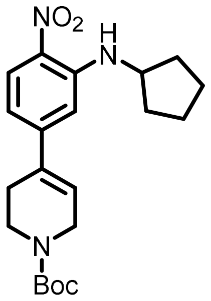

To a stirred solution of 4-Bromo-2-fluoro-1-nitrobenzene (300 g, 1.36 mol) and cyclopentylamine (128 g, 1.5 mol) in DMF (2.5 L), was added K_2CO_3 (471 g, 3.4 mol) at RT. The solution was stirred for 16 hours at RT. The reaction mixture was diluted with ice cold water. The resultant precipitate was filtered and washed with water. The residue was dried under reduced pressure to give 5-Bromo-*N*-cyclopentyl-2-nitroaniline (300 g) which was taken directly to the next step.

To a stirred solution of 5-Bromo-*N*-cyclopentyl-2-nitroaniline (200 g, 0.7 mol) in 1,4-dioxane (1.4 L) and water (600 mL), was added *N*-Boc-1,2,3,6-tetrahydropyridine-4-boronic acid pinacol ester (238 g, 0.78 mol) and Cs_2CO_3 (570 g, 1.75 mol). The mixture was degassed with argon for 15 minutes before the addition of $Pd(PPh_3)_4$ (8.1 g, 0.007 mol) at RT. The mixture was stirred over 16 hours at 100 °C. After complete conversion to the desired product the solvents were removed under reduced pressure and the residue was diluted with EtOAc. The mixture was filtered through a Celite bed which was washed twice with EtOAc. The organic layer was washed with brine, dried over Na_2SO_4, filtered and concentrated under reduced pressure. The crude residue was purified by column chromatography (30% EtOAc in petroleum ether) to give **37** (190 g, 54% yield over two steps).

^{1}H NMR (400 MHz, $CDCl_3$-*d*) δ 8.19 (br d, J = 5.4 Hz, 1H), 8.11 (d, J = 9.0 Hz, 1H), 6.74 - 6.80 (m, 1H), 6.65 (d, J = 9.0 Hz, 1H), 6.19 (br s, 1H), 4.07 - 4.18 (m, 2H), 3.99 (dq, J = 11.8, 6.0 Hz, 1H), 3.65 (t, J = 5.6 Hz, 2H), 2.51 (br s, 2H), 2.07 - 2.18 (m, 2H), 1.96 (s, 1H), 1.79 - 1.88 (m, 2H), 1.63 - 1.74 (m, 3H), 1.50 (s, 9H)

MS (ESI) for $C_{21}H_{29}N_3O_4$ $[M+H]^+$ 388

***tert*-butyl 4-(2-amino-1-cyclopentyl-1*H*-benzo[*d*]imidazol-6-yl)piperidine-1-carboxylate 38**

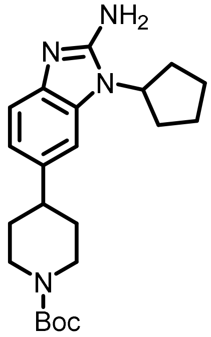

Palladium (10% on carbon, 14 g) was added to a solution of **37** (65 g, 0.17 mol) in THF (600 mL). The reaction mixture was stirred for 14 hours with a hydrogen pressure of 80 PSI at RT. The reaction was filtered through a Celite bed followed by washing twice with EtOAc. The filtrate was concentrated under reduced pressure to give *tert*-butyl 4-(4-amino-3-(cyclopentylamino)phenyl)piperidine-1-carboxylate (50 g) which was taken forward as a crude product.

To a stirred solution of *tert*-butyl 4-(4-amino-3-(cyclopentylamino)phenyl)piperidine-1-carboxylate (60 g, 0.17 mol) in MeOH (600 mL) at 0 °C was added portion-wise cyanogen bromide (26.3 g, 0.25 mol) and the reaction mixture was stirred vigorously for 2 hours at RT. The mixture was concentrated and the residue was dissolved in DCM. The solution was washed with sat. aq. $NaHCO_3$ then water, then dried over Na_2SO_4, filtered and concentrated under reduced pressure. The crude product was washed with diethyl ether to obtain **38** (50 g, 65% yield over two steps).

^1H NMR (400 MHz, DMSO-d_6) δ 7.03 (d, J = 8.3 Hz, 1H), 6.97 (s, 1H), 6.80 (dd, J = 8.1, 1.2 Hz, 1H), 4.69 (quin, J = 8.7 Hz, 1H), 4.06 (br d, J = 13.2 Hz, 2H), 2.79 (br s, 2H), 2.60 - 2.73 (m, 1H), 2.00 - 2.11 (m, 2H), 1.93 (br d, J = 4.4 Hz, 4H), 1.62 - 1.80 (m, 4H), 1.43 - 1.55 (m, 2H), 1.41 (s, 9H)

MS (ESI) for $C_{22}H_{32}N_4O_2$ $[M+H]^+$ 385

***tert*-butyl 4-(2-((2-amino-6-bromobenzoyl)imino)-3-cyclopentyl-2,3-dihydro-1*H*-benzo[*d*]imidazol-5-yl)piperidine-1-carboxylate 39**

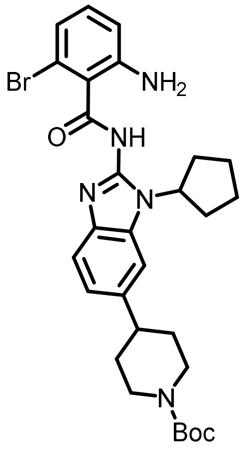

To a stirred solution of **38** (100 g, 0.26 mol) in 1,4-dioxane (1 L) was added **35** (63 g, 0.26 mol) at RT and the reaction mixture was stirred for 14 hours at 120 °C. The mixture was cooled to RT and concentrated under reduced pressure. The residue was purified by column chromatography (10-80% EtOAc in petroleum ether) to yield **39** (82 g, 54% yield).

^1H NMR (400 MHz, DMSO-d_6) δ 12.71 (br s, 1H), 7.48 (d, J = 7.8 Hz, 1H), 7.32 (s, 1H), 7.12 (br d, J = 8.3 Hz, 1H), 6.85 - 6.93 (m, 1H), 6.74 (d, J = 7.3 Hz, 1H), 6.66 (d, J = 8.3 Hz, 1H), 5.46 (s, 2H), 5.15 - 5.32 (m, 1H), 4.08 (br d, J = 11.7 Hz, 2H), 2.73 - 2.86 (m, 3H), 2.14 (br d, J = 6.8 Hz, 2H), 1.87 - 1.96 (m, 4H), 1.78 (br d, J = 12.2 Hz, 2H), 1.65 (br s, 2H), 1.49 - 1.60 (m, 2H), 1.40 (s, 9H)

MS (ESI) for $C_{29}H_{36}BrN_5O_3$ $[M+H]^+$ 582

tert-butyl 4-(4-amino-7-cyclopentyl-5-oxo-5,7-dihydrobenzo[4,5]imidazo[1,2-*a*]quinazolin-9-yl)piperidine-1-carboxylate 40

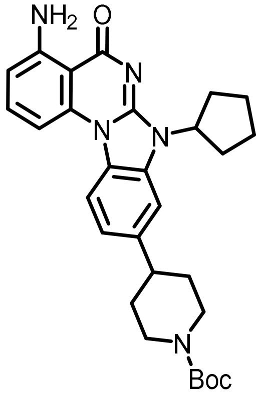

To a stirred solution of **39** (27.5 g, 0.047 mol) in *N*-methylpyrrolidine (270 mL), was added 1,10-phenanthroline (1.7 g, 9.4 mmol), copper(I) iodide (4.5 g, 23.6 mmol) and Cs_2CO_3 (23 g, 71 mmol) and the reaction mixture was degassed with nitrogen for 5 minutes then stirred at 120 °C for 1 hour. The mixture was cooled to RT and diluted with ice cold water. The mixture was extracted with EtOAc and the organic layer was dried over Na_2SO_4, filtered and concentrated under reduced pressure. The product was recrystallized from EtOAc to yield **40** (6.6 g, 28% yield).

^{1}H NMR (400 MHz, DMSO-d_6) δ 8.18 (br d, J = 8.3 Hz, 1H), 7.50 (s, 1H), 7.36 - 7.42 (m, 1H), 7.31 - 7.36 (m, 1H), 7.23 (br d, J = 7.8 Hz, 1H), 6.65 (br d, J = 7.8 Hz, 1H), 5.16 - 5.29 (m, 1H), 4.10 (br d, J = 9.8 Hz, 2H), 2.85 (br d, J = 11.7 Hz, 2H), 2.26 (br d, J = 3.9 Hz, 2H), 1.90 - 2.07 (m, 4H), 1.67 - 1.86 (m, 5H), 1.53 - 1.66 (m, 2H), 1.41 (s, 9H)

MS (ESI) for $C_{29}H_{35}N_5O_3$ $[M+H]^+$ 502

4-bromo-7-cyclopentyl-9-(piperidin-4-yl)benzo[4,5]imidazo[1,2-*a*]quinazolin-5(7*H*)-one 4

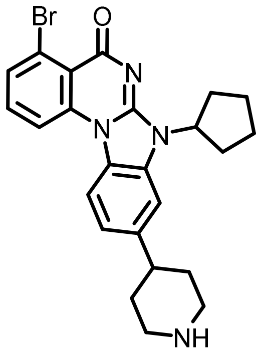

To a stirred solution of **40** (10.0 g, 19.9 mmol) in MeCN (70 mL) and acetic acid (105 mL) was added sodium nitrite (1.38 g, 19.9 mmol), dissolved in water (35 mL) dropwise add 0 °C. It was stirred at 0 °C for 10 minutes. Then copper(I) bromide (3.43 g, 23.9 mmol) was added portion wise, followed by dropwise addition of HBr (47% in water) (35.7 mL, 283 mmol) at the same temperature. The mixture was allowed to reach RT and stirred at 50 °C for 15 minutes. It was filtered, the filtrate was diluted with water and basified with sodium bicarbonate solution to pH 8 before it was extracted with DCM/MeOH 9:1 three times. The combined organic layers were dried over Na_2SO_4 and concentrated *in vacuo*. The residue was purified by acidic HPLC (Method 3a) to yield **4** (6.5 g, 70% yield).

^{1}H NMR (600 MHz, DMSO-d_6) δ 8.47 (br d, J = 8.4 Hz, 1 H), 8.31 (br d, J = 8.4 Hz, 1 H), 7.80 (br d, J = 7.9 Hz, 1 H), 7.65 - 7.72 (m, 1 H), 7.51 (s, 1 H), 7.27 (br d, J = 8.4 Hz, 1 H), 5.27 (quin, J = 8.5 Hz, 1 H), 3.11 (br d, J = 11.7 Hz, 2 H), 2.82 (br t, J = 11.9 Hz, 1 H), 2.68 (br t, J = 11.8 Hz, 2 H), 2.27 (br d, J = 3.3 Hz, 2 H), 2.04 (br s, 4 H), 1.72 - 1.84 (m, 4 H), 1.59 - 1.70 (m, 2 H)

^{13}C NMR (DMSO-d_6, 150 MHz) δ 164.6, 149.3, 144.4, 139.3, 133.8, 132.6, 130.8, 125.3, 123.3, 121.5, 117.0, 115.8, 114.1, 109.6, 54.4, 46.7, 42.5, 34.3, 28.5, 25.1

HRMS (m/z) for $C_{24}H_{25}BrN_4O$ $[M+H]^+$ calculated 465.12845; obtained 465.12704

4-bromo-7-cyclopentyl-9-(1-methylpiperidin-4-yl)benzo[4,5]imidazo[1,2-*a*]quinazolin-5(7*H*)-one 3

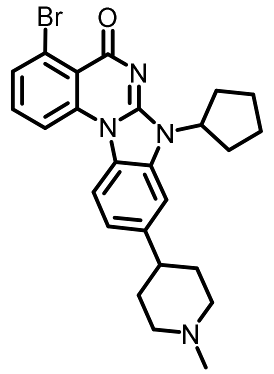

4 (50 mg, 0.092 mmol) was suspended in DMF (1 mL). Formaldehyde (80 µL, 1.1 mmol) and acetic acid (0.5 mL) were added and the reaction mixture was stirred at RT for 1 hour. $NaBH(OAc)_3$ (247 mg, 1.1 mmol) was added and the reaction mixture was stirred at RT for 40 minutes. Upon completion, the mixture was quenched with water and basified with 4M NaOH. The resultant precipitate was collected by filtration and washed with water. The solid was dissolved in MeCN/water and freeze dried to yield **3** (48 mg, quantitative yield).

^{1}H NMR (500 MHz, DMSO-d_6) δ 8.48 (d, J = 8.5 Hz, 1H), 8.30 (d, J = 8.5 Hz, 1H), 7.80 (d, J = 7.6 Hz, 1H), 7.68 (t, J = 8.2 Hz, 1H), 7.55 (s, 1H), 7.29 (d, J = 8.5 Hz, 1H), 5.26 (quin, J = 8.7 Hz, 1H), 2.91 (br d, J = 11.0 Hz, 2H), 2.63 - 2.73 (m, 1H), 2.24 - 2.33 (m, 2H), 2.22 (s, 3H), 2.02 (br dd, J = 6.5, 2.7 Hz, 6H), 1.70 - 1.84 (m, 6H)

^{13}C NMR (125 MHz, DMSO-d_6) δ 164.6, 149.3, 144.0, 139.3, 133.8, 132.6, 130.9, 125.3, 123.3, 121.6, 117.0, 115.8, 114.1, 109.7, 56.3, 54.4, 46.7, 41.7, 33.7, 28.4, 25.0

HRMS (m/z) for $C_{25}H_{27}BrN_4O$ $[M+H]^+$ calculated 479.14410; obtained 479.14317

Supplementary Figure 15: synthesis of substituted benzoxazinones 13-17

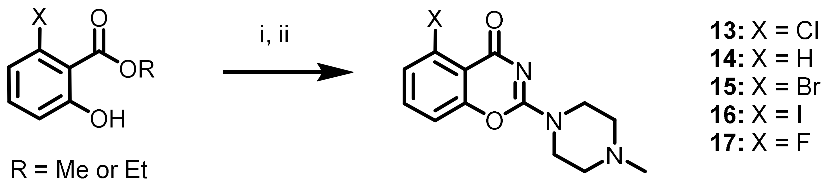

i) cyanogen bromide, Et_3N, acetone, 0 °C ; ii) *N*-methylpiperazine, RT

5-chloro-2-(4-methylpiperazin-1-yl)-4*H*-benzo[*e*][1,3]oxazin-4-one 13

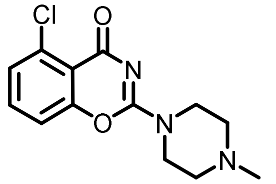

To a solution of ethyl 2-chloro-6-hydroxybenzoate (50 mg, 0.24 mmol) and Et_3N (33 µL, 0.24 mmol) in acetone (1 mL) at 0 °C was added cyanogen bromide (3.0 M solution in THF, 79 µL, 0.24 mmol). The reaction mixture was stirred at RT overnight. To this solution was added *N*-methylpiperazine (53 µL, 0.47 mmol) and the reaction mixture was stirred at RT for 1 hour. Upon completion, water was added and the solvents were removed *in vacuo*. Purification by basic HPLC (Method 3b) gave the product **13** (33 mg, 50% yield).

^{1}H NMR (500 MHz, DMSO-d_6) δ 7.64 (t, J = 7.9 Hz, 1H), 7.41 - 7.45 (m, 2H), 3.71 (br s, 4H), 2.42 (br s, 4H), 2.23 (s, 3H)

^{13}C NMR (125 MHz, DMSO-d_6) δ 163.5, 155.6, 155.5, 134.3, 133.0, 128.6, 116.1, 114.9, 54.3, 54.1, 46.0, 44.4, 43.8

HRMS (m/z) for $_{13}H_{14}ClN_3O_2$ $[M+H]^+$ calculated 280.08473; obtained 280.08432

2-(4-methylpiperazin-1-yl)-4*H*-benzo[*e*][1,3]oxazin-4-one 14

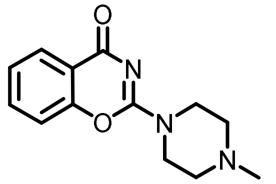

To a solution of methyl 2-hydroxybenzoate (50 µL, 0.39 mmol) and Et_3N (54 µL, 0.39 mmol) in acetone (1 mL) at 0 °C was added cyanogen bromide (3.0 M solution in THF, 129 µL, 0.39 mmol). The reaction mixture was stirred at RT overnight. To this solution was added *N*-methylpiperazine (86 µL, 0.78 mmol) and the reaction mixture was stirred at RT for 1 hour. Upon completion, water was added and the solvents were removed *in vacuo*. Purification by basic HPLC (Method 3b) gave the product **14** (61 mg, 64% yield).

^{1}H NMR (500 MHz, DMSO-d_6) δ 7.88 (dd, J = 7.6, 1.6 Hz, 1H), 7.72 (ddd, J = 8.4, 7.2, 1.7 Hz, 1H), 7.45 (d, J = 8.5 Hz, 1H), 7.38 - 7.43 (m, 1H), 3.74 (br s, 4H), 2.43 (br s, 4H), 2.24 (s, 3H)

^{13}C NMR (125 MHz, DMSO-d_6) δ 165.9, 156.8, 153.8, 134.7, 127.1, 126.0, 117.5, 116.5, 54.4, 54.2, 46.0, 44.4, 43.9

HRMS (m/z) for $C_{13}H_{15}N_3O_2$ $[M+H]^+$ calculated 246.12370; obtained 246.12305

5-bromo-2-(4-methylpiperazin-1-yl)-4*H*-benzo[*e*][1,3]oxazin-4-one 15

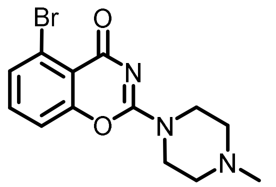

To a solution of methyl 2-bromo-6-hydroxybenzoate (77 mg, 0.33 mmol) and Et_3N (47 µL, 0.33 mmol) in acetone (1 mL) at 0 °C was added cyanogen bromide (3.0 M solution in THF, 111 µL, 0.33 mmol). The reaction mixture was stirred at RT overnight. To this solution was added *N*-methylpiperazine (74 µL, 0.67 mmol) and the reaction mixture was stirred at RT for 1 hour. Upon completion, water was added and the solvents were removed *in vacuo*. Purification by basic HPLC (Method 3b) gave the product **15** (37 mg, 34% yield).

^1H NMR (500 MHz, DMSO-d_6) δ 7.56 (dd, J = 7.9, 0.9 Hz, 1H), 7.48 (t, J = 8.0 Hz, 1H), 7.40 (dd, J = 8.4, 1.1 Hz, 1H), 3.64 (br s, 4H), 2.34 (br s, 4H), 2.16 (s, 3H)

^{13}C NMR (125 MHz, DMSO-d_6) δ 163.7, 155.5, 155.3, 134.6, 132.2, 121.0, 116.8, 115.9, 54.3, 54.1, 46.0, 44.4, 43.8

HRMS (m/z) for $C_{13}H_{14}BrN_3O_2$ $[M+H]^+$ calculated 324.03421; obtained 324.03375

5-iodo-2-(4-methylpiperazin-1-yl)-4*H*-benzo[*e*][1,3]oxazin-4-one 16

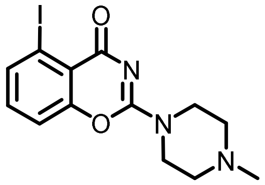

To a solution of methyl 2-hydroxy-6-iodobenzoate (44 mg, 0.16 mmol) and Et_3N (22 µL, 0.16 mmol) in acetone (1 mL) at 0 °C was added cyanogen bromide (3.0 M solution in THF, 53 µL, 0.16 mmol). The reaction mixture was stirred at RT overnight. To this solution was added *N*-methylpiperazine (35 µL, 0.32 mmol) and the reaction mixture was stirred at RT for 1 hour. Upon completion, water was added and the solvents were removed *in vacuo*. Purification by basic HPLC (Method 3b) gave the product **16** (34 mg, 58% yield).

^1H NMR (500 MHz, DMSO-d_6) δ 7.96 (dd, J = 7.9, 0.9 Hz, 1H), 7.48 (dd, J = 8.2, 0.9 Hz, 1H), 7.35 (t, J = 8.0 Hz, 1H), 3.71 (br s, 4H), 2.42 (br s, 4H), 2.23 (s, 3H)

^{13}C NMR (125 MHz, DMSO-d_6) δ 163.9, 155.5, 154.3, 139.3, 135.1, 117.4, 117.3, 54.3, 54.1, 46.0, 44.4, 43.7

HRMS (m/z) for $C_{13}H_{14}IN_3O_2$ $[M+H]^+$ calculated 372.02035; obtained 372.01919

5-fluoro-2-(4-methylpiperazin-1-yl)-4*H*-benzo[*e*][1,3]oxazin-4-one 17

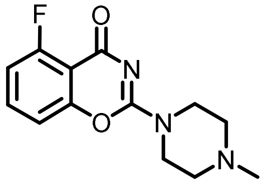

To a solution of methyl 2-fluoro-6-hydroxybenzoate (50 mg, 0.29 mmol) and Et_3N (60 µL, 0.43 mmol) in acetone (1 mL) at 0 °C was added cyanogen bromide (3.0 M solution in THF, 144 µL, 0.43 mmol). The reaction mixture was stirred at RT overnight. To this solution was added *N*-methylpiperazine (64 µL, 0.58 mmol) and the reaction mixture was stirred at RT for 1 hour. Upon completion, water was added and the solvents were removed *in vacuo*. Purification by basic HPLC (Method 3b) gave the product **17** (45 mg, 59% yield).

^{1}H NMR (500 MHz, DMSO-d_6) δ 7.70 (td, J = 8.4, 5.8 Hz, 1H), 7.30 (d, J = 8.5 Hz, 1H), 7.18 (dd, J = 9.6, 8.7 Hz, 1H), 3.72 (br s, 4H), 2.42 (br s, 4H), 2.23 (s, 3H)

^{13}C NMR (125 MHz, DMSO-d_6) δ 162.91 (d, CF = 3.6 Hz), 160.18 (d, CF = 259.8 Hz), 155.99, 154.84 (d, CF = 3.6 Hz), 135.12 (d, CF = 10.9 Hz), 113.32 (d, CF = 20.9 Hz), 112.75 (d, CF = 4.5 Hz), 107.30 (d, CF = 11.8 Hz), 54.28, 54.07, 45.97, 44.41, 43.84

HRMS (m/z) for $C_{13}H_{14}FN_3O_2$ $[M+H]^+$ calculated 264.11428; obtained 264.11353

Supplementary Figure 16: synthesis of compound 18

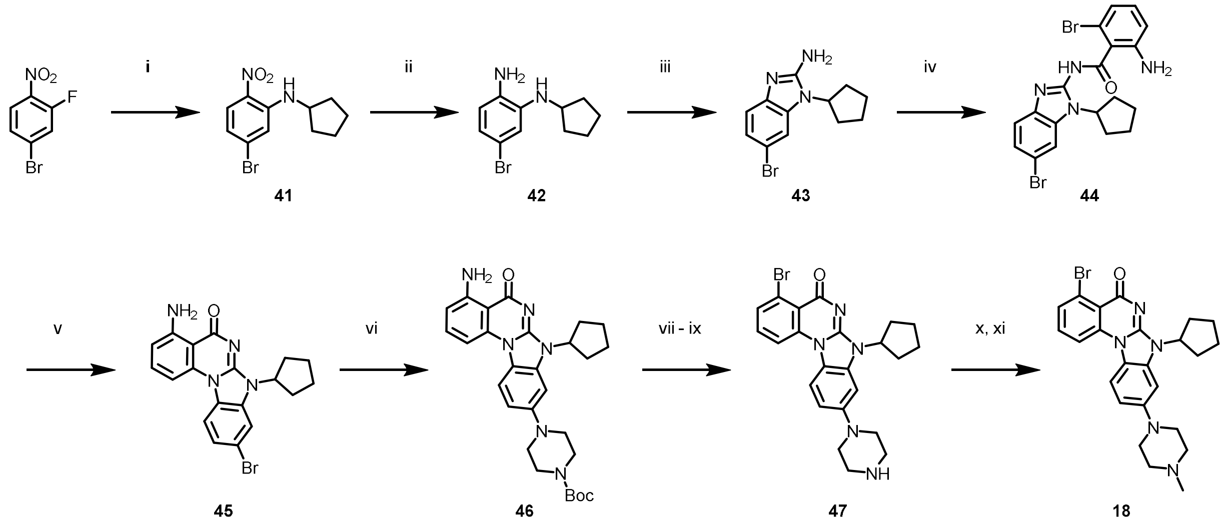

i) cyclopentylamine, K_2CO_3, DMF, 50 °C ; ii) Zn, NH_4COOH, MeOH, 50 °C ; iii) cyanogen bromide, MeOH, RT ; iv) **35**, 1,4-dioxane, 120 °C ; v) CuI, 1,10-phenanthroline, Cs_2CO_3, DMF, 120 °C ; vi) *N*-Boc piperazine, RuPhos Pd G4, LiHMDS, THF, 85 °C ; vii) AcOH/MeCN, 0 °C ; viii) $NaNO_2$,H_2O, 0 °C ; ix) CuBr, HBr, RT ; x) CH_2O, AcOH, DMF, RT ; xi) $NaBH(OAc)_3$

5-bromo-*N*-cyclopentyl-2-nitroaniline 41

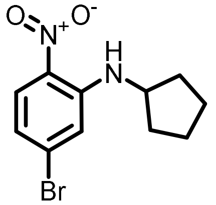

4-Bromo-2-fluoro-1-nitrobenzene (2.00 g, 9.09 mmol) and K_2CO_3 (2.51 g, 18.2 mmol) were suspended in DMF (20 mL). Then cyclopentylamine (1.0 mL, 10.1 mmol) was added and the mixture was stirred at 50 °C for 1 hour. It was diluted with water and stirred at RT for 10 minutes. The solids were collected by filtration, rinsed with water and dried at 50 °C under vacuum to obtain **41** (2.51 g, 97% yield).

^{1}H NMR (400 MHz, DMSO-d_6) δ 7.99 (d, J = 9.1 Hz, 2H), 7.28 (d, J = 1.8 Hz, 1H), 6.85 (dd, J = 9.1, 2.0 Hz, 1H), 4.09 (sxt, J = 6.4 Hz, 1H), 2.06 (dq, J = 12.1, 6.0 Hz, 2H), 1.44 - 1.77 (m, 6H)

MS (ESI) for $C_{11}H_{13}BrN_2O_2$ $[M+H]^+$ 285

5-bromo-*N*[1]-cyclopentylbenzene-1,2-diamine 42

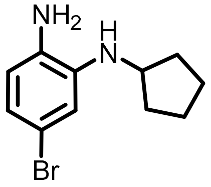

41 (1.00 g, 3.51 mmol) and NH_4COOH (1.33 g, 21.0 mmol) were suspended in MeOH (10 mL) and zinc dust (500 mg, 7.49 mmol) was added. The mixture was stirred at 50 °C for 30 minutes. Another portion of zinc dust (500 mg, 7.49 mmol) was added and stirring was continued at 50 °C for 15 minutes. After complete conversion, the mixture was filtered, rinsed with MeOH and the filtrate was concentrated *in vacuo*. The crude was purified by silica gel column chromatography (0-5% MeOH in DCM) to yield **42** (767 mg, 86% yield).

^{1}H NMR (400 MHz, DMSO-d_6) δ 6.47 - 6.51 (m, 1H), 6.45 (d, J = 2.0 Hz, 1H), 6.42 - 6.44 (m, 1H), 4.71 (br s, 2H), 4.54 (br d, J = 6.1 Hz, 1H), 3.66 (sxt, J = 6.0 Hz, 1H), 1.93 (dq, J = 12.0, 6.1 Hz, 2H), 1.62 - 1.77 (m, 2H), 1.51 - 1.60 (m, 2H), 1.40 - 1.51 (m, 2H)

MS (ESI) for $C_{11}H_{15}BrN_2$ $[M+H]^+$ 255

6-bromo-1-cyclopentyl-1*H*-benzo[*d*]imidazol-2-amine 43

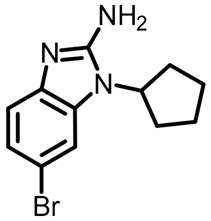

To a stirred solution of **42** (1.00 g, 3.92 mmol) in MeOH (20 mL) was added cyanogen bromide (1.66 g, 15.7 mmol) in one portion. The mixture was stirred at RT for 4 hours. After complete conversion, the mixture was poured into sat. aq. $NaHCO_3$ solution. The solids were collected by filtration, rinsed with water and diethyl ether and dried *in vacuo* to yield **43** (700 mg, 64% yield).

^{1}H NMR (400 MHz, DMSO-d_6) δ 7.24 (s, 1H), 7.04 (s, 2H), 6.46 (s, 2H), 4.69 (quin, J = 8.7 Hz, 1H), 1.83 - 2.04 (m, 6H), 1.60 - 1.70 (m, 2H)

MS (ESI) for $C_{12}H_{14}BrN_3$ $[M+H]^+$ 280

2-amino-6-bromo-*N*-(6-bromo-1-cyclopentyl-1*H*-benzo[*d*]imidazol-2-yl)benzamide 44

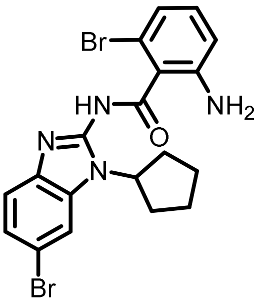

43 (1.30 g, 4.64 mmol) and **36** (1.40 g, 5.80 mmol) were suspended in 1,4-dioxane (40 mL). The mixture was stirred at 120 °C for 1 hour and the solvent was removed *in vacuo*. The residue was suspended in 30 mL of MeCN/water (3:1), sonicated for 2 minutes and stored in the fridge for 10 minutes. The solids were collected by filtration, rinsed with MeCN/water (3:1) and dried *in vacuo* to yield **44** (1.98 g, 89% yield).

^{1}H NMR (400 MHz, DMSO-d_6) δ 12.93 (br s, 1H), 7.70 (d, J = 1.5 Hz, 1H), 7.49 - 7.54 (m, 1H), 7.42 (dd, J = 8.5, 1.6 Hz, 1H), 6.88 - 6.97 (m, 1H), 6.76 (d, J = 7.1 Hz, 1H), 6.68 (d, J = 8.1 Hz, 1H), 5.49 (s, 2H), 5.11 - 5.26 (m, 1H), 2.07 - 2.23 (m, 2H), 1.87 - 2.03 (m, 4H), 1.60 - 1.73 (m, 2H)

MS (ESI) for $C_{19}H_{18}Br_2N_4O$ $[M+H]^+$ 477

4-amino-9-bromo-7-cyclopentylbenzo[4,5]imidazo[1,2-*a*]quinazolin-5(7*H*)-one 45

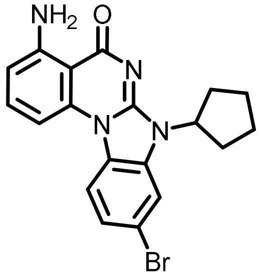

To a stirred solution of **44** (1.98 g, 4.14 mmol) in dry DMF (270 mL), was added 1,10-phenanthroline (149 mg, 0.83 mmol), copper(I) iodide (394 mg, 2.07 mmol) and Cs_2CO_3 (2.02 g, 6.21 mmol) and the reaction mixture was degassed with argon for 5 minutes then stirred at 120 °C for 20 minutes. The mixture was cooled to RT, filtered and concentrated *in vacuo*. The crude was purified by silica gel column chromatography (0-10% MeOH in DCM) to yield **45** (1.11 g, 67% yield).

^{1}H NMR (400 MHz, DMSO-d_6) δ 8.25 (d, J = 8.9 Hz, 1H), 7.86 (d, J = 1.8 Hz, 1H), 7.51 (dd, J = 8.7, 1.6 Hz, 1H), 7.40 - 7.47 (m, 1H), 7.33 (d, J = 8.1 Hz, 1H), 6.69 (d, J = 8.4 Hz, 1H), 5.19 (quin, J = 8.7 Hz, 1H), 2.21 - 2.31 (m, 2H), 2.00 (br s, 4H), 1.71 (br d, J = 5.3 Hz, 2H)

MS (ESI) for $C_{19}H_{17}BrN_4O$ $[M+H]^+$ 397

tert-butyl 4-(4-amino-7-cyclopentyl-5-oxo-5,7-dihydrobenzo[4,5]imidazo[1,2-_a_]quinazolin-9-yl)piperazine-1-carboxylate 46

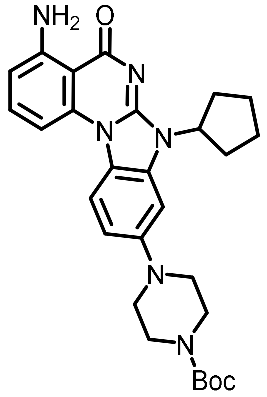

45 (300 mg, 0.68 mmol), _N_-Boc piperazine (333 mg, 1.7 mmol) and RuPhos Pd G4 (59 mg, 0.068 mmol) were suspended in THF (6 mL). To the reaction mixture was added dropwise LiHMDS (1 M in THF, 4.8 mL, 4.8 mmol). The mixture was flushed with argon for 5 minutes and stirred for 20 minutes at 85 °C. The mixture was cooled to RT and diluted with DCM and sat. aq. NH_4Cl. The organic layer was separated and concentrated under reduced pressure. The crude residue was purified by silica gel column chromatography (0-5% MeOH (+NH_3) in DCM) to obtain **46** (227 mg, 60% yield).

^1H NMR (400 MHz, $CDCl_3$-_d_) δ 7.92 (br d, J = 9.8 Hz, 1H), 7.41 (t, J = 8.1 Hz, 1H), 6.85 - 6.96 (m, 3H), 6.58 (br d, J = 7.8 Hz, 1H), 5.45 - 5.69 (m, 1H), 4.07 - 4.08 (m, 1H), 3.59 - 3.71 (m, 4H), 3.12 - 3.25 (m, 4H), 2.17 (br s, 3H), 1.97 - 2.06 (m, 2H), 1.82 (br s, 2H), 1.50 (s, 9H)

MS (ESI) for $C_{28}H_{34}N_6O_3$ $[M+H]^+$ 503

4-bromo-7-cyclopentyl-9-(piperazin-1-yl)benzo[4,5]imidazo[1,2-_a_]quinazolin-5(7_H_)-one 47

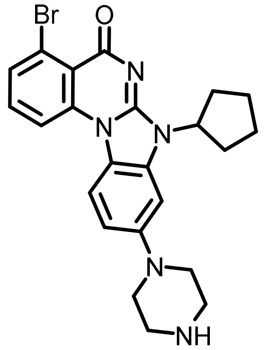

46 (88 mg, 0.18 mmol) was dissolved in a mixture of MeCN (1 mL) and acetic acid (2 mL) and cooled to 0 °C. A solution of sodium nitrite (12 mg, 0.18 mmol) in water (1 mL) was added and the mixture was stirred for 5 minutes. Copper(I) bromide (30 mg, 0.21 mmol) and HBr (48% aq., 2 mL) were added and the reaction mixture was stirred for 30 minutes at RT. Upon completion of the reaction, the mixture was filtered and purified by acidic HPLC (Method 3a) to furnish **47** (50 mg, 61% yield).

^1H NMR (400 MHz, DMSO-d_6) δ 8.41 (d, J = 8.1 Hz, 1H), 8.24 (d, J = 9.1 Hz, 1H), 7.79 (d, J = 7.1 Hz, 1H), 7.68 (t, J = 8.1 Hz, 1H), 7.16 (d, J = 2.0 Hz, 1H), 7.01 (dd, J = 9.1, 2.0 Hz, 1H), 5.10 - 5.30 (m, 1H), 3.40 (br s, 4H), 3.20 (br s, 4H), 2.22 - 2.32 (m, 2H), 1.95 - 2.07 (m, 4H), 1.74 (br dd, J = 6.1, 5.3 Hz, 2H)

MS (ESI) for $C_{23}H_{24}BrN_5O$ [M+H]$^+$ 466

4-bromo-7-cyclopentyl-9-(4-methylpiperazin-1-yl)benzo[4,5]imidazo[1,2-*a*]quinazolin-5(7*H*)-one 18

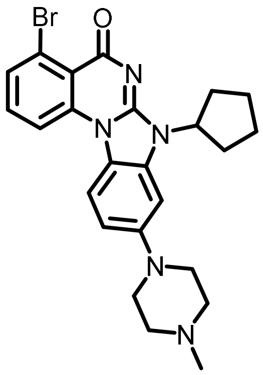

47 (78 mg, 0.16 mmol) was suspended in DMF (1 mL). Formaldehyde (120 µL, 1.6 mmol) and acetic acid (0.5 mL) were added and the reaction mixture was stirred at RT for 1 hour. NaBH(OAc)$_3$ (400 mg, 1.8 mmol) was added and the reaction mixture was stirred at RT for 1 hour. Upon completion, the mixture was quenched with water and basified with 4M NaOH. The resultant precipitate was collected by filtration and washed with water. The solid was dissolved in MeCN/water and freeze dried to yield **18** (76 mg, quantitative yield).

^{1}H NMR (500 MHz, DMSO-*d*$_6$) δ 8.41 (d, J = 8.5 Hz, 1H), 8.19 (d, J = 9.1 Hz, 1H), 7.78 (d, J = 7.6 Hz, 1H), 7.67 (t, J = 8.0 Hz, 1H), 7.13 (d, J = 2.2 Hz, 1H), 6.97 (dd, J = 9.1, 2.2 Hz, 1H), 5.22 (quin, J = 8.7 Hz, 1H), 3.22 - 3.29 (m, 4H), 2.27 - 2.35 (m, 2H), 2.26 (s, 3H), 1.96 - 2.05 (m, 4H), 1.69 - 1.78 (m, 2H)

^{13}C NMR (125 MHz, DMSO-*d*$_6$) δ 164.4, 149.4, 149.1, 139.2, 133.7, 132.4, 132.2, 123.3, 119.6, 116.8, 115.6, 114.6, 110.7, 98.1, 55.0, 54.3, 49.0, 46.3, 28.4, 25.2

HRMS (m/z) for $C_{24}H_{26}BrN_5O$ [M+H]$^+$ calculated 480.13935; obtained 480.13813

Synthesis of phenolic PROTACs

Compounds 48, *cis*-48, 50 and **51** were prepared according to literature procedures[2,3]

Supplementary Figure 17: synthesis of phenolic PROTAC 5

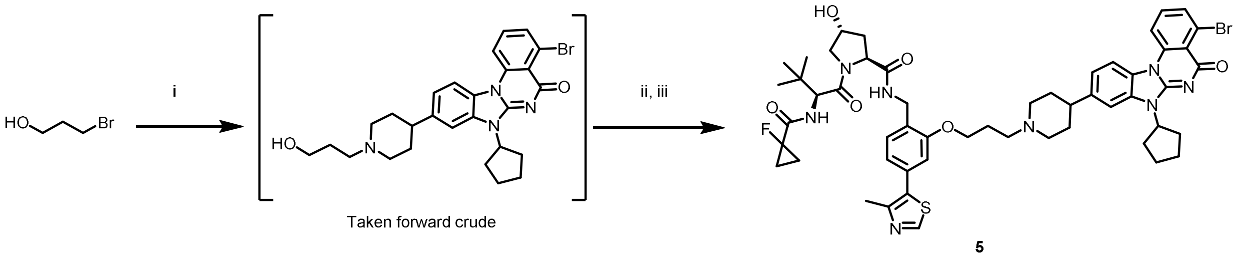

i) **4**, NaI, acetone, 50 °C ; ii) MsCl, Et$_3$N, DCM/THF/DMSO, 0 °C – RT ; iii) **48**, K_2CO_3, DMF, 75 °C

(2*S*,4*R*)-*N*-(2-(3-(4-(4-bromo-7-cyclopentyl-5-oxo-5,7-dihydrobenzo[4,5]imidazo[1,2-*a*]quinazolin-9-yl)piperidin-1-yl)propoxy)-4-(4-methylthiazol-5-yl)benzyl)-1-((*S*)-2-(1-fluorocyclopropane-1-carboxamido)-3,3-dimethylbutanoyl)-4-hydroxypyrrolidine-2-carboxamide 5

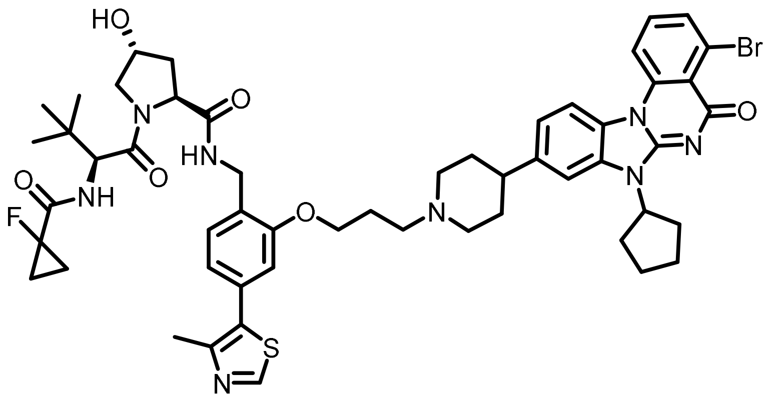

A mixture of **4** (50 mg, 0.11 mmol), K_2CO_3 (40 mg, 0.29 mmol), sodium iodide (20 mg, 0.13 mmol) and 3-bromopropan-1-ol (15 µL, 0.17 mmol) in acetone (1.0 mL) was stirred at 50 °C overnight. The reaction mixture was concentrated and purified by SCX chromatography: the column was washed with MeOH and the crude residue was applied as a solution in MeOH. The product was eluted using 3 M ammonia in MeOH. The ammonia phase was concentrated *in vacuo*. The crude intermediate was dissolved in a mixture of DCM (1.0 mL), THF (2.0 mL) and DMSO (0.5 mL) and cooled to 0 °C. Et_3N (42 µL, 0.30 mmol) was added followed by dropwise addition of MsCl (15 µL, 0.19 mmol) and the mixture was allowed to warm to RT and stirred overnight. The mixture was concentrated under reduced pressure and dissolved in DMF (3.0 mL). K_2CO_3 (99 mg, 0.72 mmol) and **48** (217 mg, 0.41 mmol) were added and the mixture was stirred at 75 °C overnight. The mixture was filtered over a fritted column and concentrated under reduced pressure. The residue was purified by basic HPLC (Method 4b) to yield **5** (13 mg, 9% yield).

^{1}H NMR (600 MHz, DMSO-*d₆*) δ 9.05 (s, 1H), 8.58 (br t, J = 6.0 Hz, 1H), 8.53 (d, J = 8.4 Hz, 1H), 8.35 (d, J = 8.6 Hz, 1H), 7.85 (d, J = 7.7 Hz, 1H), 7.73 (t, J = 8.2 Hz, 1H), 7.60 (s, 1H), 7.48 (d, J = 7.9 Hz, 1H), 7.30 - 7.39 (m, 3H), 7.10 (d, J = 1.3 Hz, 1H), 7.03 (dd, J = 7.9, 1.3 Hz, 1H), 5.24 - 5.36 (m, 2H), 4.66 (d, J = 9.2 Hz, 1H), 4.59 (t, J = 8.3 Hz, 1H), 4.42 (br s, 1H), 4.27 - 4.40 (m, 3H), 4.19 (br t, J = 6.0 Hz, 2H), 3.64 - 3.75 (m, 2H), 3.11 (br d, J = 11.0 Hz, 2H), 2.53 (s, 3H), 2.35 (br dd, J = 8.5, 3.9 Hz, 3H), 1.93 - 2.20 (m, 7H), 1.74 - 1.91 (m, 7H), 1.36 - 1.49 (m, 3H), 1.28 (br dd, J = 8.8, 2.4 Hz, 3H), 1.01 - 1.04 (m, 9H)

^{13}C NMR (150 MHz, DMSO-d_6) δ 172.3, 169.4, 168.5 (d, CF=19.1 Hz), 164.6, 156.4, 149.3, 148.3, 144.1, 139.3, 133.8, 132.6, 131.8, 131.4, 131.0, 128.2, 127.5, 125.3, 123.3, 121.7, 121.2, 117.0, 115.8, 114.0, 112.2, 109.7, 78.6 (d, CF=232.7 Hz), 69.4, 66.6, 59.3, 57.2, 57.0, 55.2, 54.4, 54.4, 54.3, 42.4, 38.7, 38.4, 37.7, 36.5, 36.2, 33.8, 28.4, 26.9, 26.7, 26.6, 25.0, 16.5, 13.3 (m, CF)

HRMS (m/z) for $C_{53}H_{62}BrFN_8O_6S$ $[M+H]^+$ calculated 1037.37532; obtained 1037.37217

Supplementary Figure 18: synthesis of PEG-based phenolic PROTACs 19-21

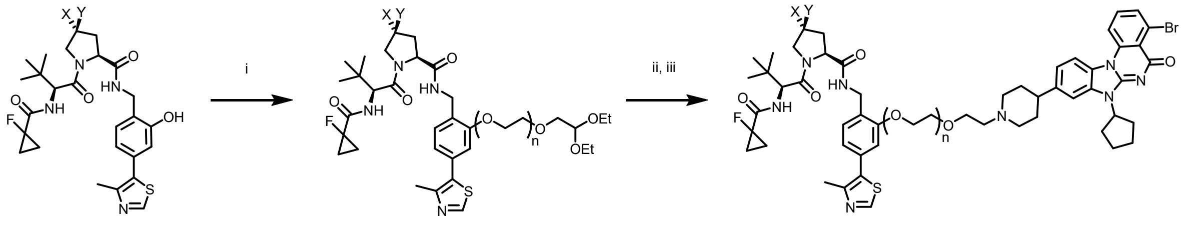

48: X = OH, Y = H
***cis*-48:** X = H, Y = OH

49: n = 0
50: n = 2
51: n = 3
} X = OH, Y = H

19: n = 0, X = H, Y = OH
20: n = 0
21: n = 2
22: n = 3
} X = OH, Y = H

i) Alkyl bromide or tosylate, K_2CO_3, DMF, 80 °C ; ii) HCl, THF/H_2O, 75 °C ; iii) **4**, Et_3N, DMF, RT, then $NaBH(OAc)_3$

(2*S*,4*R*)-*N*-(2-(2,2-diethoxyethoxy)-4-(4-methylthiazol-5-yl)benzyl)-1-((*S*)-2-(1-fluorocyclopropane-1-carboxamido)-3,3-dimethylbutanoyl)-4-hydroxypyrrolidine-2-carboxamide 49

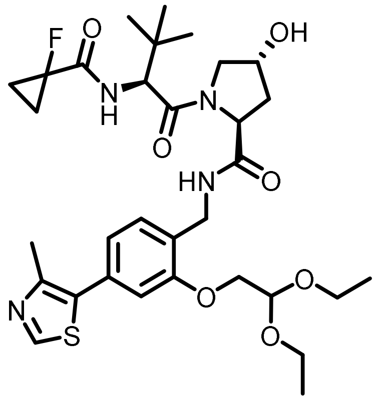

2-bromo-1,1-diethoxyethane (20 µL, 26 mg, 0.13 mmol) was added to a solution of **48** (50 mg, 0.094 mmol) and K_2CO_3 (50 mg, 0.36 mmol) in DMF (1.0 mL). The reaction mixture was stirred at 80 °C overnight. It was cooled to room temperature, diluted with H_2O and DCM and passed through a phase separator and the organic layer was concentrated. The remaining residue was purified by column chromatography on silica gel (0-10% MeOH in DCM) to yield **49** (47 mg, 77% yield).

^{1}H NMR (400 MHz, $CDCl_3$-*d*) δ 8.60 (s, 1H), 7.26 (m, 2H), 6.98 (dd, J = 9.0, 3.6 Hz, 1H), 6.90 (dd, J = 7.7, 1.6 Hz, 1H), 6.83 (d, J = 1.6 Hz, 1H), 4.86 (t, J = 5.2 Hz, 1H), 4.63 (t, J = 7.7 Hz, 1H), 4.51 – 4.38 (m, 3H), 4.35 (dd, J = 14.9, 5.4 Hz, 1H), 4.06 – 3.93 (m, 2H), 3.88 (dt, J = 11.4, 1.9 Hz, 1H), 3.72 (m, 2H), 3.59 (m, 3H), 3.41 (d, J = 4.3 Hz, 1H), 2.44 (s, 4H), 2.07 – 1.96 (m, 1H), 1.31 – 1.23 (m, 1H), 1.27 – 1.11 (m, 8H), 0.86 (s, 9H)

MS (ESI) for $C_{32}H_{45}FN_4O_7S$ $[M+H]^+$ 649

(2*S*,4*R*)-*N*-(2-(2-(4-(4-bromo-7-cyclopentyl-5-oxo-5,7-dihydrobenzo[4,5]imidazo[1,2-*a*]quinazolin-9-yl)piperidin-1-yl)ethoxy)-4-(4-methylthiazol-5-yl)benzyl)-1-((*S*)-2-(1-fluorocyclopropane-1-carboxamido)-3,3-dimethylbutanoyl)-4-hydroxypyrrolidine-2-carboxamide 20

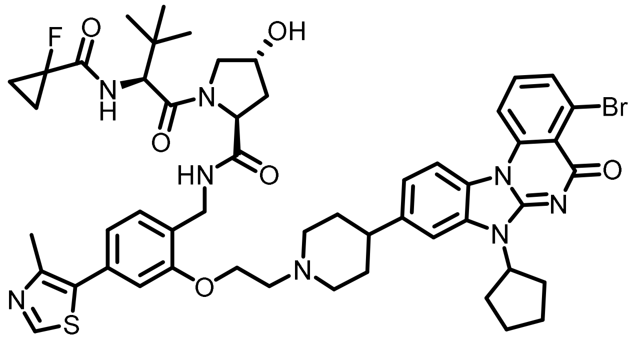

49 (47 mg, 0.072 mmol) was dissolved in THF (1.0 mL) and 0.5 M aq. HCl (1.0 mL). The reaction mixture was stirred at 75 °C for 1 hour. The reaction mixture was then concentrated *in vacuo*. To the resultant residue was added **4** (24 mg, 0.052 mmol) in DCE (2.0 mL) and DMSO was added dropwise until all components were in solution. Et_3N (150 µL, 1.04 mmol) was then added followed by $MgSO_4$ and $NaBH(OAc)_3$ (40 mg, 0.19 mmol). The reaction mixture was stirred at RT overnight. The reaction was diluted with DCM, filtered and concentrated under reduced pressure. The residue was purified by acidic HPLC (Method 4a) to yield **20** (15 mg, 20% yield).

^{1}H NMR (600 MHz, DMSO-*d$_6$*) δ 9.00 (m, 1H), 8.53 (m, 1H), 8.47 (d, J = 8.4 Hz, 1H), 8.31 (br d, J = 8.4 Hz, 1H), 7.80 (d, J = 7.7 Hz, 1H), 7.68 (m, 1H), 7.54 (m, 1H), 7.42 (d, J = 7.7 Hz, 1H), 7.24 - 7.33 (m, 2H), 7.08 (m, 1H), 6.99 (br d, J = 7.5 Hz, 1H), 5.26 (m, 1H), 5.18 (d, J = 3.5 Hz, 1H), 4.59 (d, J = 9.2 Hz, 1H), 4.53 (m, 1H), 4.20 - 4.38 (m, 5H), 3.58 - 3.68 (m, 2H), 2.65 - 3.24 (m, 5H), 2.27 (br d, J = 4.8 Hz, 4H), 1.97 - 2.15 (m, 7H), 1.69 - 1.97 (m, 8H), 1.29 - 1.43 (m, 2H), 1.18 - 1.26 (m, 2H), 0.94 (m, 9H)

^{13}C NMR (150 MHz, DMSO-*d$_6$*) δ 171.2, 168.3, 167.4 (d, CF=20.3 Hz), 163.5, 148.3, 147.3, 138.2, 132.7, 131.6, 130.7, 130.4, 129.8, 127.2, 126.4, 124.3, 122.2, 120.5, 116.0, 114.8, 113.0, 111.3, 108.6, 77.5 (d, CF=232.7 Hz), 68.3, 58.2, 56.1, 55.9, 53.3, 37.3, 36.7, 35.4, 27.4, 25.5, 24.0, 15.4, 12.2 (m, CF)

HRMS (m/z) for $C_{52}H_{60}BrFN_8O_6S$ $[M+H]^+$ calculated 1023.35967; obtained 1023.35761

(2*S*,4*S*)-*N*-(2-(2-(4-(4-bromo-7-cyclopentyl-5-oxo-5,7-dihydrobenzo[4,5]imidazo[1,2-*a*]quinazolin-9-yl)piperidin-1-yl)ethoxy)-4-(4-methylthiazol-5-yl)benzyl)-1-((*S*)-2-(1-fluorocyclopropane-1-carboxamido)-3,3-dimethylbutanoyl)-4-hydroxypyrrolidine-2-carboxamide 19

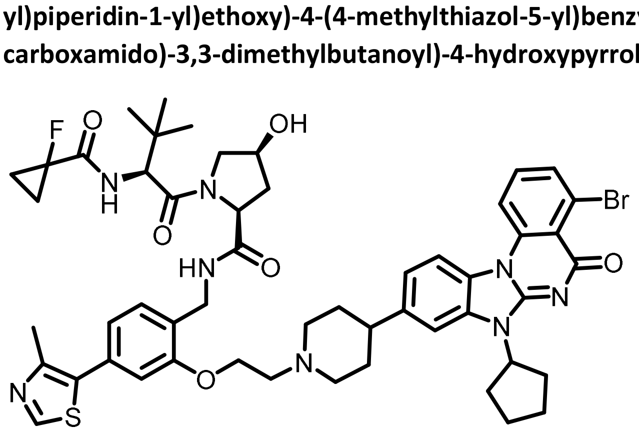

19 was prepared according to same procedures as for **20** using ***cis*-48**

^{1}H NMR (600 MHz, DMSO-d_6) δ 9.00 (s, 1H), 8.57 (t, J = 5.9 Hz, 1H), 8.47 (d, J = 8.4 Hz, 1H), 8.29 (d, J = 8.6 Hz, 1H), 7.79 (d, J = 7.7 Hz, 1H), 7.67 (t, J = 8.2 Hz, 1H), 7.55 (s, 1H), 7.41 (d, J = 7.7 Hz, 1H), 7.25 - 7.32 (m, 2H), 7.09 (d, J = 0.9 Hz, 1H), 6.97 (dd, J = 7.7, 1.1 Hz, 1H), 5.45 (d, J = 7.3 Hz, 1H), 5.25 (quin, J = 8.8 Hz, 1H), 5.16 (d, J = 4.8 Hz, 1H), 4.55 (d, J = 8.8 Hz, 1H), 4.45 (dd, J = 8.4, 6.2 Hz, 1H), 4.31 - 4.38 (m, 1H), 4.18 - 4.29 (m, 4H), 3.86 (dd, J = 10.2, 5.6 Hz, 1H), 3.46 (dd, J = 9.9, 5.3 Hz, 1H), 3.13 (br d, J = 10.5 Hz, 2H), 2.83 (br t, J = 5.5 Hz, 2H), 2.68 - 2.77 (m, 1H), 2.48 (s, 3H), 2.34 - 2.38 (m, 1H), 2.16 - 2.32 (m, 5H), 2.02 (br d, J = 5.5 Hz, 3H), 1.68 - 1.87 (m, 6H), 1.28 - 1.42 (m, 2H), 1.14 - 1.26 (m, 2H), 0.97 (s, 9H)

^{13}C NMR (150 MHz, DMSO-d_6) δ 171.8, 168.5, 167.8 (d, CF=20.3 Hz), 163.5, 155.3, 150.9, 148.2, 147.3, 143.0, 138.2, 132.7, 131.5, 130.7, 130.4, 129.9, 127.1, 126.2, 124.2, 122.2, 120.5, 120.2, 116.0, 114.8, 113.0, 111.4, 108.6, 77.4 (d, CF=231.4 Hz), 68.4, 65.9, 58.1, 56.4, 56.1, 55.1, 53.6, 53.4, 41.0, 36.9, 36.3, 34.9, 32.8, 27.4, 25.6, 23.9, 15.4, 12.3 (m, CF)

HRMS (m/z) for $C_{52}H_{60}BrFN_8O_6S$ $[M+H]^+$ calculated 1023.35967; obtained 1023.35734

(2*S*,4*R*)-*N*-(2-(2-(2-(2-(4-(4-bromo-7-cyclopentyl-5-oxo-5,7-dihydrobenzo[4,5]imidazo[1,2-*a*]quinazolin-9-yl)piperidin-1-yl)ethoxy)ethoxy)ethoxy)-4-(4-methylthiazol-5-yl)benzyl)-1-((*S*)-2-(1-fluorocyclopropane-1-carboxamido)-3,3-dimethylbutanoyl)-4-hydroxypyrrolidine-2-carboxamide 21

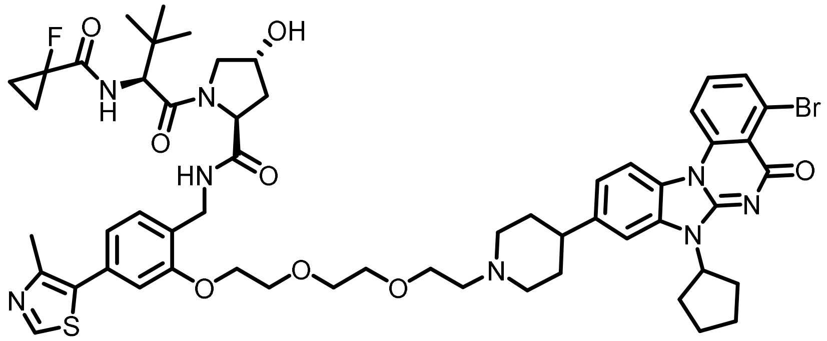

50 (47 mg, 0.064 mmol) was dissolved in THF (1.0 mL) and 0.5 M aq. HCl (1.0 mL). The reaction was stirred at 75 °C for 45 minutes. The mixture was concentrated under reduced pressure. To the residue was added **4** (24 mg, 0.052 mmol) in DCE (2.0 mL) and DMSO was added dropwise until all components were in solution. Et_3N (150 µL, 1.04 mmol) was then added followed by $MgSO_4$ and $NaBH(OAc)_3$ (40 mg, 0.19 mmol). The reaction mixture was stirred at RT overnight. The reaction was diluted with DCM, filtered and concentrated under reduced pressure. The residue was purified by acidic HPLC (Method 4a) to yield **21** (15 mg, 21% yield).

^{1}H NMR (500 MHz, DMSO-d_6) δ 8.88 (s, 1H), 8.44 (br t, J = 6.0 Hz, 1H), 8.39 (d, J = 8.2 Hz, 1H), 8.20 (d, J = 8.5 Hz, 1H), 7.73 (d, J = 7.9 Hz, 1H), 7.61 (t, J = 8.2 Hz, 1H), 7.45 (s, 1H), 7.34 (d, J = 7.9 Hz, 1H), 7.22 (br dd, J = 9.1, 2.5 Hz, 1H), 7.19 (dd, J = 8.7, 0.8 Hz, 1H), 6.97 (d, J = 1.3 Hz, 1H), 6.89 (dd, J = 7.7, 1.4 Hz, 1H), 5.17 (br t, J = 8.8 Hz, 1H), 5.12 (br s, 1H), 4.52 (d, J = 9.1 Hz, 1H), 4.45 (t, J = 8.2 Hz, 1H), 4.21 - 4.32 (m, 3H), 4.09 - 4.20 (m, 4H), 3.70 - 3.78 (m, 3H), 3.46 - 3.60 (m, 4H), 2.94 (br d, J = 11.0 Hz, 2H), 2.58 - 2.63 (m, 1H), 2.37 (s, 3H), 2.11 - 2.26 (m, 3H), 1.98 - 2.09 (m, 3H), 1.94 (br d, J = 5.0 Hz, 5H), 1.84 (ddd, J = 12.9, 8.8, 4.4 Hz, 1H), 1.62 - 1.75 (m, 7H), 1.24 - 1.36 (m, 2H), 1.14 (br dd, J = 8.2, 2.2 Hz, 2H), 0.88 (s, 9H)

HRMS (m/z) for $C_{56}H_{68}BrFN_8O_8S$ $[M+H]^+$ calculated 1111.41210; obtained 1111.40973

(2*S*,4*R*)-*N*-(2-(2-(2-(2-(2-(4-(4-bromo-7-cyclopentyl-5-oxo-5,7-dihydrobenzo[4,5]imidazo[1,2-*a*]quinazolin-9-yl)piperidin-1-yl)ethoxy)ethoxy)ethoxy)ethoxy)-4-(4-methylthiazol-5-yl)benzyl)-1-((*S*)-2-(1-fluorocyclopropane-1-carboxamido)-3,3-dimethylbutanoyl)-4-hydroxypyrrolidine-2-carboxamide 22

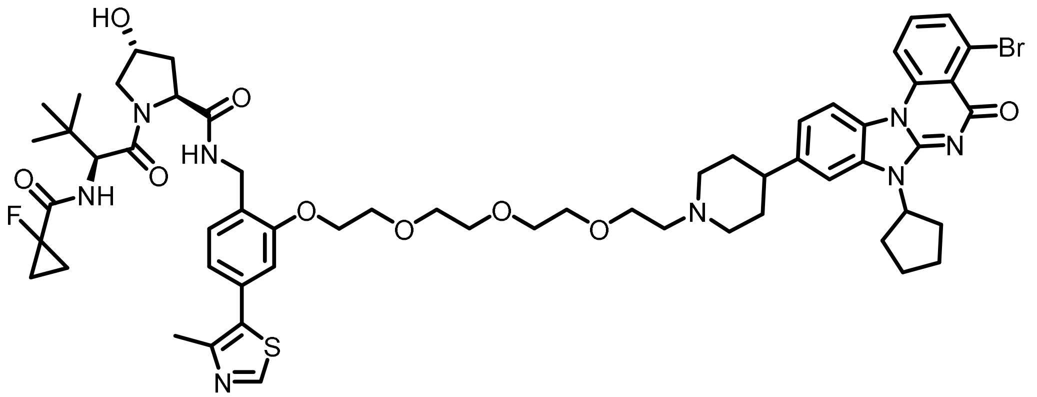

51 (55 mg, 0.07 mmol) was dissolved in THF (1.0 mL) and 0.5 M aq. HCl (1.0 mL). The reaction was stirred at 75 °C for 45 minutes. The mixture was concentrated under reduced pressure. To the residue was added **4** (37 mg, 0.080 mmol) in DCE (2.0 mL) and DMSO was added dropwise until all components were in solution. The reaction mixture was stirred until all species were in solution. Et_3N 150 µL, 1.04 mmol) was then added followed by $MgSO_4$ and $NaBH(OAc)_3$ (40 mg, 0.19 mmol). The reaction mixture was stirred at RT overnight. The reaction was diluted with DCM, filtered and concentrated under reduced pressure. The residue was purified by acidic HPLC (Method 4a) to yield **22** (12 mg, 15% yield).

^{1}H NMR (400 MHz, DMSO-d_6) δ 8.95 (s, 1H), 8.50 (t, J = 6.0 Hz, 1H), 8.45 (d, J = 8.6 Hz, 1H), 8.24 - 8.29 (m, 1H), 7.80 (d, J = 7.6 Hz, 1H), 7.68 (t, J = 8.1 Hz, 1H), 7.52 (s, 1H), 7.41 (d, J = 7.9 Hz, 1H), 7.30 (dd, J = 9.3, 2.7 Hz, 1H), 7.26 (d, J = 7.9 Hz, 1H), 7.03 (d, J = 1.3 Hz, 1H), 6.96 (dd, J = 7.9, 1.3 Hz, 1H), 5.15 - 5.32 (m, 2H), 4.59 (d, J = 9.1 Hz, 1H), 4.51 (t, J = 8.2 Hz, 1H), 4.27 - 4.40 (m, 2H), 4.15 - 4.26 (m, 3H), 3.77 - 3.86 (m, 2H), 3.61 - 3.69 (m, 4H), 3.49 - 3.60 (m, 9H), 3.01 (br d, J = 11.2 Hz, 2H), 2.61 - 2.72 (m, 1H), 2.44 (s, 3H), 2.20 - 2.31 (m, 2H), 1.87 - 2.16 (m, 9H), 1.67 - 1.82 (m, 6H), 1.29 - 1.44 (m, 2H), 1.21 (dd, J = 8.4, 3.3 Hz, 2H), 0.95 (s, 9H)

13C NMR (150 MHz, DMSO-d_6) δ 172.4, 169.4, 168.7 (d, CF=20.3 Hz), 164.7, 156.3, 151.9, 149.2, 148.3, 144.0, 139.2, 133.9, 132.7, 131.7, 131.3, 130.8, 128.2, 127.6, 125.3, 123.3, 121.7, 121.5, 116.9, 115.8, 114.0, 112.5, 109.6, 78.6 (d, CF=231.4 Hz), 70.6, 70.3, 70.3, 70.1, 69.5, 69.4, 68.8, 68.4, 59.3, 57.9, 57.1, 57.0, 54.5, 54.4, 42.1, 38.3, 37.8, 36.5, 33.6, 28.4, 26.6, 25.0, 16.4, 13.3 (m, CF)

HRMS (m/z) for $C_{58}H_{72}BrFN_8O_9S$ $[M+H]^+$ calculated 1155.43831; obtained 1155.43543

Supplementary Figure 19: Synthesis of phenolic PROTAC 5

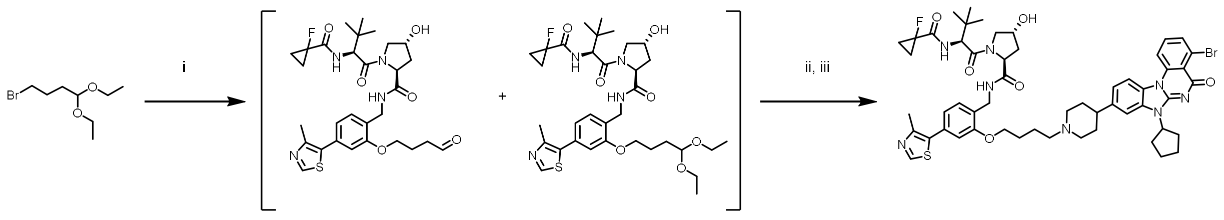

i) **48**, K_2CO_3, DMF, 75 °C ; ii) HCl, THF/H_2O, 75 °C ; iii) **4**, Et_3N, DCE/DMSO, RT, then $NaBH(OAc)_3$

(2*S*,4*R*)-*N*-(2-(4-(4-(4-bromo-7-cyclopentyl-5-oxo-5,7-dihydrobenzo[4,5]imidazo[1,2-*a*]quinazolin-9-yl)piperidin-1-yl)butoxy)-4-(4-methylthiazol-5-yl)benzyl)-1-((*S*)-2-(1-fluorocyclopropane-1-carboxamido)-3,3-dimethylbutanoyl)-4-hydroxypyrrolidine-2-carboxamide 23

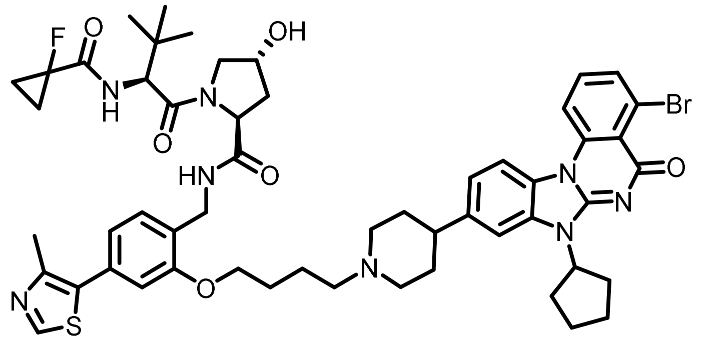

A mixture of **48** (82 mg, 0.15 mmol), 4-bromo-1,1-diethoxybutane (80 mg, 0.36 mmol) and K_2CO_3 (48 mg, 0.35 mmol) in DMF (2.0 mL) was stirred at 75 °C overnight. The reaction was allowed to cool to RT then diluted with water and DCM and filtered through a phase separator. The filtrate was then concentrated under reduced pressure. The residue was purified by column chromatography on silica gel (0-5% MeOH in DCM), which enabled isolation of a 1:1 mixture of aldehyde and acetal. The mixture was dissolved in THF (1.0 mL) and 0.5 M aq. HCl (1.0 mL). The mixture was heated at 75 °C for 45 minutes. The mixture was allowed to cool to RT and concentrated under reduced pressure. To the residue was added **4** in DCE (2.0 mL) and DMSO (0.6 mL), followed by Et_3N (150 µL, 1.04 mmol), $MgSO_4$ and $NaBH(OAc)_3$ (40 mg, 0.19 mmol). The mixture was stirred at room temperature overnight. The mixture was filtered on a fritted column and concentrated under reduced pressure. The residue was purified by acidic HPLC (Method 4a) to yield **23** (21 mg, 37% yield).

^{1}H NMR (600 MHz, DMSO-d_6) δ 8.98 (s, 1H), 8.51 (br t, J = 5.9 Hz, 1H), 8.47 (br d, J = 8.4 Hz, 1H), 8.28 (br d, J = 8.4 Hz, 1H), 8.20 (s, 1H), 7.80 (d, J = 7.9 Hz, 1H), 7.65 - 7.71 (m, 1H), 7.55 (s, 1H), 7.41 (br d, J = 7.7 Hz, 1H), 7.25 - 7.31 (m, 2H), 7.03 (s, 1H), 6.97 (br d, J = 7.9 Hz, 1H), 5.25 (quin, J = 8.8 Hz, 1H), 5.07 - 5.20 (m, 1H), 4.60 (br d, J = 9.2 Hz, 1H), 4.53 (br t, J = 8.3 Hz, 1H), 4.36 (br s, 1H), 4.21 - 4.34 (m, 2H), 4.11 (br t, J = 5.9 Hz, 2H), 3.59 - 3.72 (m, 2H), 3.06 (br d, J = 10.6 Hz, 2H), 2.72 (br t, J = 12.2 Hz, 1H), 2.47 (s, 3H), 2.41 - 2.45 (m, 2H), 2.23 - 2.34 (m, 2H), 1.97 - 2.15 (m, 7H), 1.90 - 1.97 (m, 1H), 1.64 - 1.87 (m, 7H), 1.31 - 1.44 (m, 3H), 1.22 (br dd, J = 8.5, 2.7 Hz, 3H), 0.95 (s, 9H)

^{13}C NMR (150 MHz, DMSO-d_6) δ 172.3, 169.4, 168.5 (d, CF=20.3 Hz), 164.6, 156.3, 149.3, 148.3, 144.0, 139.3, 133.8, 132.6, 131.8, 131.3, 131.0, 128.1, 127.4, 125.3, 123.3, 121.6, 121.2, 117.0, 115.8, 114.0,

112.1, 109.7, 78.6 (d, CF=231.4 Hz), 69.4, 68.1, 59.3, 58.2, 57.1, 57.0, 54.5, 54.2, 42.3, 40.9, 38.4, 37.7, 36.5, 33.7, 29.5, 28.4, 27.2, 26.7, 26.6, 25.0, 23.4, 16.5, 13.3 (m, CF)

HRMS (m/z) for $C_{54}H_{64}BrFN_8O_6S$ $[M+H]^+$ calculated 1051.39097; obtained 1051.38758

Synthesis of benzylic PROTACs

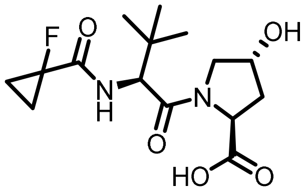

52

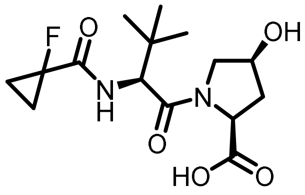

***cis*-52**

Carboxylic acids **52** and ***cis*-52** were synthesised according to literature procedures[4]

Supplementary Figure 20: synthesis of benzylic PROTAC 6

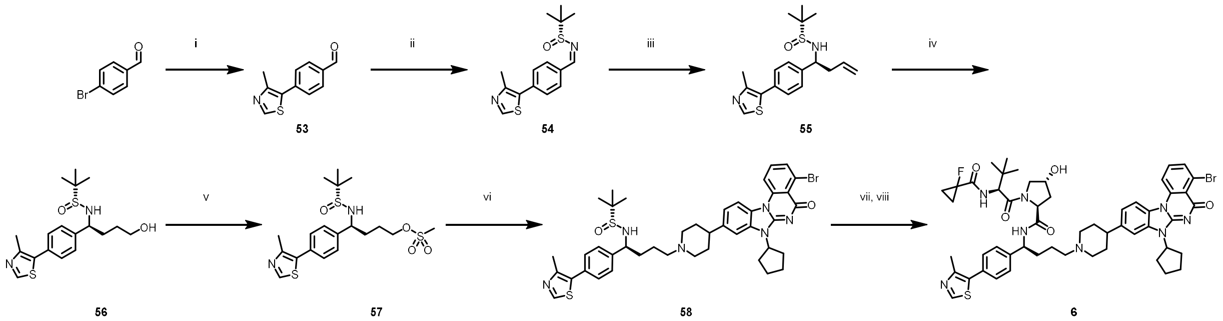

i) 4-methylthiazole, Pd(OAc)$_2$, KOAc, DMA, 150 °C ; ii) (*R*)-2-methylpropane-2-sulfinamide, Ti(O^{i}Pr)$_4$, THF, RT ; iii) allylmagnesium bromide, THF, 0 °C – RT ; iv) 9-BBN, THF, 0 °C – RT, then 30% aq.H_2O_2, 4 M NaOH, 0 °C – RT ; v) MsCl, DIPEA, DCM, 0 °C – RT ; vi) **4**, DIPEA, DMF, 80 °C ; vii) HCl, DCM, MeOH, 0 °C – RT ; viii) **52**, HATU, DIPEA, DMF, RT

4-(4-methylthiazol-5-yl)benzaldehyde 53

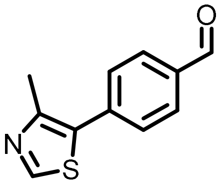

A suspension of 4-methylthiazole (5.9 mL, 64.9 mmol), 4-bromobenzaldehyde (6.0 g, 32.4 mmol), Pd(OAc)$_2$ (76 mg, 0.34 mmol) and KOAc (6.4 g, 64.9 mmol) in DMA (30 mL) was heated to 150 °C and stirred at this temperature overnight. The mixture was cooled and diluted in EtOAc and partitioned with brine. The aqueous phase was extracted three times and the combined organics were dried over $MgSO_4$ and concentrated. The residue was purified by column chromatography on silica gel (0-50% EtOAc in heptane) to yield **53** (4.9 g, 75% yield).

^{1}H NMR (400 MHz, $CDCl_3$-*d*) δ 10.08 (s, 1H), 8.78 (s, 1H), 8.03 – 7.93 (m, 2H), 7.69 – 7.60 (m, 2H), 2.62 (s, 3H)

MS (ESI) for $C_{11}H_9NOS$ $[M+H]^+$ 204

(*R*)-2-methyl-*N*-(4-(4-methylthiazol-5-yl)benzylidene)propane-2-sulfinamide 54

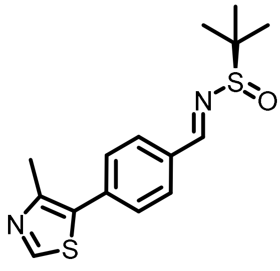

To a solution of **53** (4.9 g, 24.1 mmol) and (*R*)-2-methylpropane-2-sulfinamide (2.92 g, 24.1 mmol) in THF (50 mL) was slowly added $Ti(O^iPr)_4$ (8.5 mL, 28.9 mmol) and the reaction was stirred at RT overnight. The mixture was concentrated *in vacuo* and then diluted with EtOAc and partitioned with brine. The mixture was filtered over Celite and the organic layer was separated with water. The aqueous layer was extracted three times and the combined organics were dried over $MgSO_4$ and concentrated. The residue was purified by column chromatography on silica gel (0-50% EtOAc in heptane) to yield **54** (7.18 g, 97% yield).

^{1}H NMR (400 MHz, $CDCl_3$-*d*) δ 8.76 (s, 1H), 8.64 (s, 1H), 7.98 – 7.90 (m, 2H), 7.63 – 7.56 (m, 2H), 2.61 (s, 3H), 1.31 (s, 9H)

MS (ESI) for $C_{15}H_{18}N_2OS_2$ $[M+H]^+$ 307

(*R*)-2-methyl-*N*-((*S*)-1-(4-(4-methylthiazol-5-yl)phenyl)but-3-en-1-yl)propane-2-sulfinamide 55

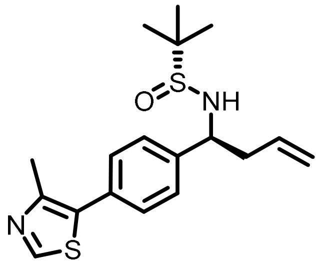

To a solution of **54** (7.18 g, 23.4 mmol) in THF (100 mL) at 0 °C was slowly added allylmagnesium bromide (1.0 M in diethyl ether) (25.8 mL, 25.8 mmol). The mixture was allowed to reach RT and stirred for 1 hour. It was quenched with sat. aq. NH_4Cl (40 mL) and extracted three times with EtOAc. The combined organics were washed with brine, dried over $MgSO_4$ and concentrated. The residue was purified by column chromatography on silica gel (0-100% EtOAc in heptane) to yield **55** (3.75 g, 46% yield, dr 68:32).

^{1}H NMR (500 MHz, $CDCl_3$-*d*) δ 8.70 (s, 1H), 7.48 – 7.38 (m, 4H), 5.78 (m, 1H), 5.28 – 5.22 (m, 1H), 4.55 (ddd, J = 8.0, 5.3, 2.3 Hz, 1H), 3.74 – 3.68 (m, 1H), 2.71 – 2.45 (m, 2H), 2.58 (s, 3H), 1.25 (s, 9H)

MS (ESI) for $C_{18}H_{24}N_2OS_2$ $[M+H]^+$ 349

(*R*)-*N*-((*S*)-4-hydroxy-1-(4-(4-methylthiazol-5-yl)phenyl)butyl)-2-methylpropane-2-sulfinamide 56

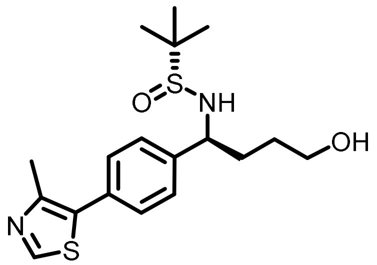

To a solution of **55** (3.75 g, 10.8 mmol) in THF (50 mL, 12.5 mmol) at 0 °C was added dropwise 9-BBN (0.5 M in THF) (64.6 mL, 32.3 mmol). The reaction was allowed to reach RT and stirred for two hours. It was cooled to 0 °C and 30% aq.H_2O_2 (12.2 mL, 107.6 mmol) was slowly added, followed by 4 M aq. NaOH (26.9 mL, 107.6 mmol). After 1 hour at RT, the mixture was adjusted to pH 4 with careful addition of 1 M aq. HCl and then extracted three times with DCM. The combined organics were washed with brine, dried over $MgSO_4$ and concentrated. The residue was purified by acidic reverse phase chromatography (Method 3a) to yield **56** (2.3 g, 58% yield).

^1H NMR (400 MHz, $CDCl_3$-*d*) δ 8.71 (s, 1H), 7.47 – 7.35 (m, 4H), 4.50 (t, J = 6.9 Hz, 1H), 3.77 – 3.61 (m, 2H), 2.57 (s, 3H), 1.78 – 1.45 (m, 5H), 1.24 (s, 9H)

MS (ESI) for $C_{18}H_{26}N_2O_2S_2$ $[M+H]^+$ 367

(*S*)-4-(((*R*)-*tert*-butylsulfinyl)amino)-4-(4-(4-methylthiazol-5-yl)phenyl)butyl methanesulfonate 57

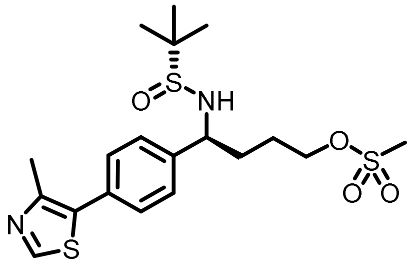

To a solution of **56** (164 mg, 0.45 mmol) in DCM (4 mL) at 0 °C was added DIPEA (218 µL, 1.34 mmol) followed by MsCl (38 µL, 0.49 mmol). The reaction was allowed to warm to RT and stirred overnight. The mixture was partitioned between DCM and sat. aq. $NaHCO_3$ and passed through a phase separator. The organic layer was dried over $MgSO_4$ and concentrated. The residue was purified by column chromatography on silica gel (0-10% MeOH in DCM) to yield **57** (140 mg, 42% yield).

^1H NMR (400 MHz, $CDCl_3$-*d*) δ 8.76 (s, 1H), 7.49 – 7.35 (m, 4H), 4.48 (t, J = 8.2 Hz, 1H), 4.24 (t, J = 6.1 Hz, 2H), 3.54 – 3.45 (m, 1H), 3.03 (s, 3H), 2.58 (s, 3H), 2.05 – 1.92 (m, 1H), 1.90 – 1.78 (m, 1H), 1.77 – 1.64 (m, 1H), 1.24 (s, 9H)

MS (ESI) for $C_{19}H_{28}N_2O_4S_3$ $[M+H]^+$ 445

(*R*)-*N*-((*S*)-4-(4-(4-bromo-7-cyclopentyl-5-oxo-5,7-dihydrobenzo[4,5]imidazo[1,2-*a*]quinazolin-9-yl)piperidin-1-yl)-1-(4-(4-methylthiazol-5-yl)phenyl)butyl)-2-methylpropane-2-sulfinamide 58

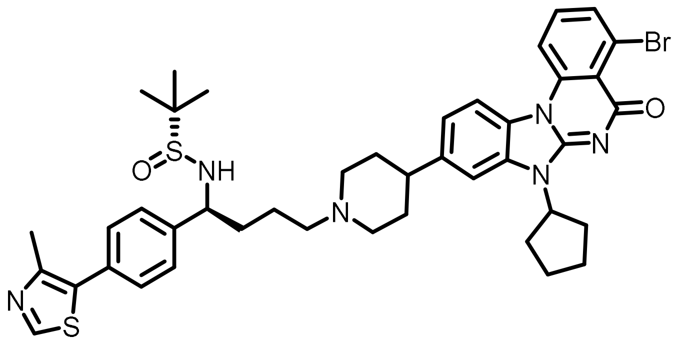

To a solution of **57** (140 mg, 0.19 mmol) and **4** (88 mg, 0.19 mmol) in DMF (2.0 mL) was added DIPEA (184 µL, 1.13 mmol) and the reaction was heated to 80 °C overnight. The mixture was concentrated in vacuo. The residue was purified by acidic reverse phase chromatography (Method 3a) followed by column chromatography on silica gel (0-10% MeOH in DCM) to yield **58** (19 mg, 12% yield).

^{1}H NMR (400 MHz, $CDCl_3$-*d*) δ 8.70 (s, 1H), 8.44 (s, 1H), 8.13 (d, J = 8.4 Hz, 1H), 7.97 (dd, J = 8.6, 2.7 Hz, 1H), 7.79 (d, J = 7.9 Hz, 1H), 7.54 (m, 1H), 7.50 – 7.36 (m, 4H), 7.36 – 7.23 (m, 2H), 5.52 (quin., J = 9.4 Hz, 1H), 4.51 – 4.41 (m, 1H), 3.70 – 3.30 (m, 2H), 3.03 – 2.68 (m, 4H), 2.63 – 2.43 (m, 1H), 2.57 (s, 3H), 2.38 – 1.90 (m, 12H), 1.86 – 1.80 (m, 4H), 1.25 (s, 9H)

MS (ESI) for $C_{42}H_{49}BrN_6O_2S_2$ $[M+H]^+$ 813

(2*S*,4*R*)-*N*-((*S*)-4-(4-(4-bromo-7-cyclopentyl-5-oxo-5,7-dihydrobenzo[4,5]imidazo[1,2-*a*]quinazolin-9-yl)piperidin-1-yl)-1-(4-(4-methylthiazol-5-yl)phenyl)butyl)-1-((*S*)-2-(1-fluorocyclopropane-1-carboxamido)-3,3-dimethylbutanoyl)-4-hydroxypyrrolidine-2-carboxamide 6

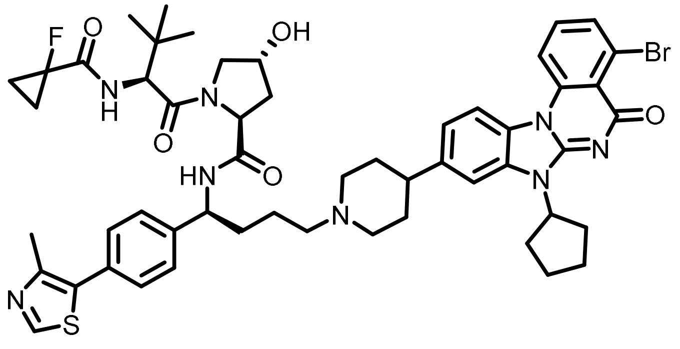

To a solution of **58** (19 mg, 0.021 mmol) in DCM (2.0 mL) and MeOH (0.2 mL) at 0 °C was added 4 M HCl in 1,4-dioxane (53 µL, 0.21 mmol) and the mixture was left to stir overnight at RT. The solvent was removed *in vacuo* and the residue was azeotroped with toluene to give the desired intermediate. To a solution of the intermediate, **52** (9.1 mg, 0.03 mmol) and HATU (11.6 mg, 0.03 mmol) in DMF (1.0 mL) was added DIPEA (15.7 µL, 0.09 mmol). The mixture was stirred at RT overnight. The mixture was diluted in DCM, partitioned with 1 M aq. HCl and passed through a phase separator. The organic layer was dried over $MgSO_4$ and concentrated. The residue was purified by column chromatography on silica gel (0-15% MeOH in DCM) followed by acidic HPLC to yield **6** (11 mg, 40% yield).

^{1}H NMR (600 MHz, DMSO-d_6) δ 9.00 (s, 1H), 8.56 (br s, 1H), 8.47 (br d, J = 8.6 Hz, 1H), 8.33 (br d, J = 7.2 Hz, 1H), 7.80 (d, J = 7.9 Hz, 1H), 7.68 (t, J = 8.1 Hz, 1H), 7.47 (br d, J = 8.3 Hz, 3H), 7.42 (br d, J = 7.9 Hz, 2H), 7.27 (br d, J = 8.1 Hz, 1H), 7.20 (br d, J = 7.7 Hz, 1H), 5.27 (quin, J = 8.7 Hz, 1H), 5.17 (br d, J = 2.6 Hz, 1H), 4.91 (br d, J = 4.0 Hz, 1H), 4.59 (br d, J = 9.2 Hz, 1H), 4.51 (t, J = 8.3 Hz, 1H), 4.31 (br s, 1H), 3.54 - 3.67 (m, 2H), 2.72 - 3.18 (m, 3H), 2.47 (s, 3H), 2.25 (br s, 3H), 1.68 - 2.10 (m, 18H), 1.29 - 1.37 (m, 2H), 1.21 (br dd, J = 8.2, 2.8 Hz, 3H), 0.93 - 1.05 (m, 9H)

^{13}C NMR (150 MHz, DMSO-d_6) δ 171.2, 169.2, 168.4 (d, CF=19.1 Hz), 164.6, 152.0, 149.4, 148.3, 139.2, 133.8, 132.7, 131.5, 130.8, 130.3, 129.3, 127.3, 123.3, 121.5, 117.1, 115.8, 114.2, 109.6, 78.7 (d, CF=232.7 Hz), 69.3, 59.3, 57.2, 57.0, 54.3, 38.3, 36.6, 28.4, 26.7, 25.1, 16.5, 13.3 (m, CF)

HRMS (m/z) for $C_{53}H_{62}BrFN_8O_5S$ $[M+H]^+$ calculated 1021.38040; obtained 1021.37861

(2*S*,4*S*)-*N*-((*S*)-4-(4-(4-bromo-7-cyclopentyl-5-oxo-5,7-dihydrobenzo[4,5]imidazo[1,2-*a*]quinazolin-9-yl)piperidin-1-yl)-1-(4-(4-methylthiazol-5-yl)phenyl)butyl)-1-((*S*)-2-(1-fluorocyclopropane-1-carboxamido)-3,3-dimethylbutanoyl)-4-hydroxypyrrolidine-2-carboxamide 7

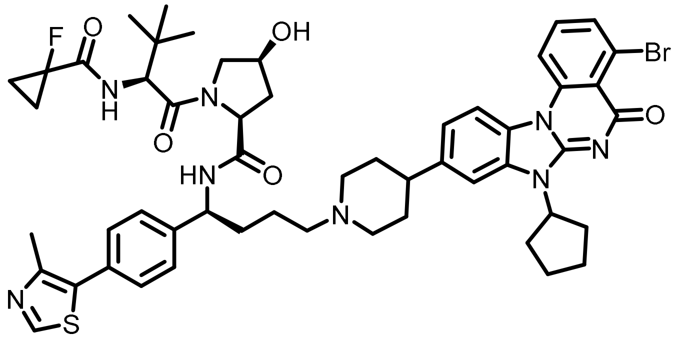

7 was prepared from ***cis*-52** according to the same procedures as **6**

^{1}H NMR (600 MHz, DMSO-d_6) δ 8.99 (s, 1H), 8.56 (d, J = 8.3 Hz, 1H), 8.47 (d, J = 8.4 Hz, 1H), 8.29 (d, J = 8.6 Hz, 1H), 7.79 (d, J = 7.7 Hz, 1H), 7.67 (t, J = 8.2 Hz, 1H), 7.52 (s, 1H), 7.45 - 7.48 (m, 2H), 7.39 - 7.43 (m, 2H), 7.27 (br d, J = 8.1 Hz, 1H), 7.24 (dd, J = 8.9, 2.3 Hz, 1H), 5.41 (d, J = 7.5 Hz, 1H), 5.25 (quin, J = 8.8 Hz, 1H), 4.89 (q, J = 7.3 Hz, 1H), 4.55 (d, J = 9.0 Hz, 1H), 4.42 (dd, J = 8.5, 6.3 Hz, 1H), 4.16 - 4.27 (m, 1H), 3.83 (dd, J = 10.2, 5.6 Hz, 1H), 3.43 (dd, J = 10.1, 5.3 Hz, 1H), 3.02 (br dd, J = 14.9, 12.1 Hz, 2H), 2.65 - 2.75 (m, 1H), 2.47 (s, 3H), 2.22 - 2.37 (m, 5H), 1.94 - 2.06 (m, 6H), 1.66 - 1.88 (m, 9H), 1.60 (dt, J = 12.7, 6.1 Hz, 1H), 1.43 - 1.55 (m, 1H), 1.28 - 1.40 (m, 2H), 1.16 - 1.27 (m, 2H), 1.00 (s, 9H)

^{13}C NMR (150 MHz, DMSO-d_6) δ 170.7, 168.3, 167.6 (d, CF=20.3 Hz), 163.5, 150.9, 148.2, 147.2, 143.1, 143.0, 138.2, 132.7, 131.5, 130.5, 129.8, 129.2, 128.3, 126.2, 124.2, 122.2, 120.6, 116.0, 114.8, 113.0, 108.5, 77.5 (d, CF=232.7 Hz), 68.4, 58.0, 57.3, 56.1, 55.0, 53.3, 53.2, 51.5, 41.4, 36.1, 35.0, 33.7, 32.8, 32.8, 27.4, 25.6, 24.0, 22.6, 15.4, 12.2 (m, CF)

HRMS (m/z) for $C_{53}H_{62}BrFN_8O_5S$ $[M+H]^+$ calculated 1021.38040; obtained 1021.37770

Supplementary Figure 21: synthesis of benzylic PROTACs 8 and 24

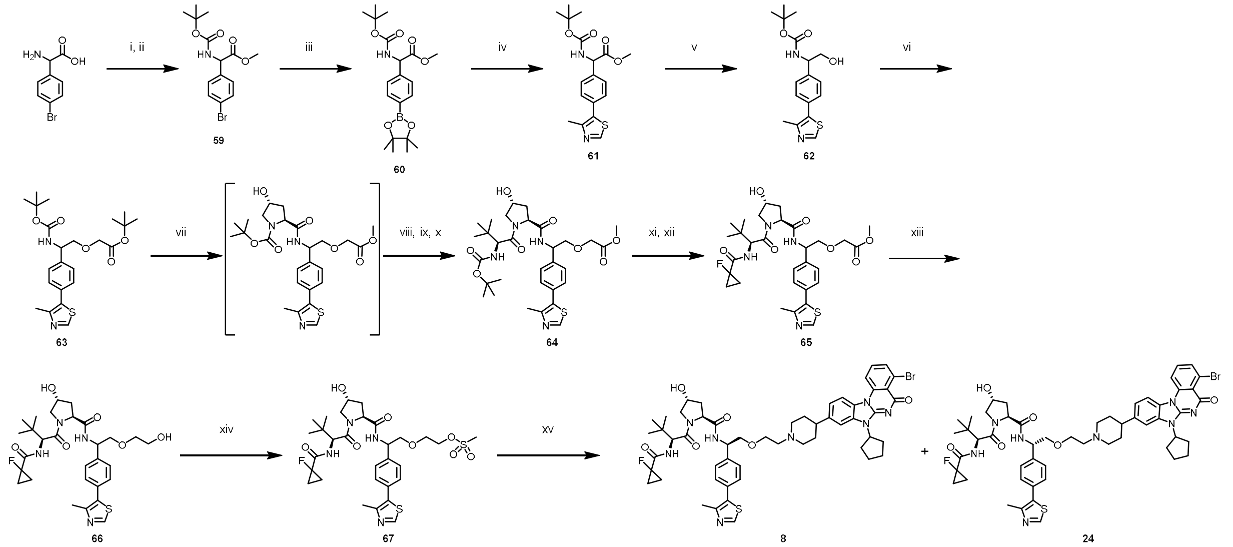

i) $SOCl_2$, MeOH, 0 °C – RT ; ii) Boc_2O, Et_3N, 1,4-dioxane/H_2O ; iii) bis(pinacolato)diboron, KOAc, $PdCl_2(PPh_3)_2$, 1,4-dioxane, 80 °C ; iv) 5-bromo-4-methylthiazole, Cs_2CO_3, Pd(dppf)Cl_2 · CH_2Cl_2, 1,4-dioxane,H_2O, 80 °C, µW ; v) $NaBH_4$, MeOH, 50 °C ; vi) *tert*-butyl bromoacetate, tetrabutylammonium hydrogen sulfate, DCM, NaOH, RT ; vii) HCl, DCM, MeOH, 0 °C – RT ; viii) $SOCl_2$, MeOH, 0 °C – RT ; ix) Boc-Hyp-OH, HATU, DIPEA, DMF, RT ; x) HCl, DCM, MeOH, 0 °C – RT ; xi) Boc-Tle-OH, HATU, DIPEA, DMF, RT ; xii) HCl, DCM, MeOH, 0 °C – RT ; xiii) 1-fluorocyclopropane-1-carboxylic acid, HATU, DIPEA, DMF, RT ; xiv) $NaBH_4$, MeOH, 50 °C ; xv) MsCl, DIPEA, DCM, 0 °C – RT ; xvi) **4**, DIPEA, DMF, 80 °C

methyl 2-(4-bromophenyl)-2-((*tert*-butoxycarbonyl)amino)acetate 59

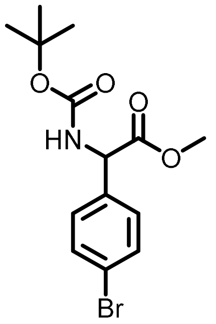

To a suspension of 2-amino-2-(4-bromophenyl)acetic acid (5.0 g, 21.73 mmol) in MeOH (75 mL) at 0 °C was slowly added $SOCl_2$ (9.51 mL, 130.40 mmol). The reaction was allowed to reach RT and stirred overnight. The solvent was removed *in vacuo* and azeotroped with toluene to yield the desired intermediate as a yellow solid. To a solution of the intermediate and Boc_2O (5.21 g, 23.91 mmol) in 1,4-dioxane (18 mL) and water (18 mL) at 0 °C was added Et_3N (12.6 mL, 86.93 mmol). The reaction was allowed to reach RT and stirred overnight. The dioxane was removed in vacuo and the aqueous mixture was partitioned with EtOAc. The organic layer was washed with sat. aq. NH_4Cl, then sat. aq. $NaHCO_3$ and then brine. It was dried over $MgSO_4$ and concentrated *in vacuo* to yield crude **59** (7.55 g, quantitative yield).

^1H NMR (400 MHz, $CDCl_3$-*d*) δ 7.45 – 7.37 (m, 2H), 7.20 – 7.13 (m, 2H), 5.54 – 5.49 (m, 1H), 5.21 (d, J = 7.1 Hz, 1H), 3.65 (s, 3H), 1.35 (s, 9H)

MS (ESI) for $C_{14}H_{18}BrNO_4$ $[M+H]^+$ 344

methyl 2-((*tert*-butoxycarbonyl)amino)-2-(4-(4,4,5,5-tetramethyl-1,3,2-dioxaborolan-2-yl)phenyl)acetate 60

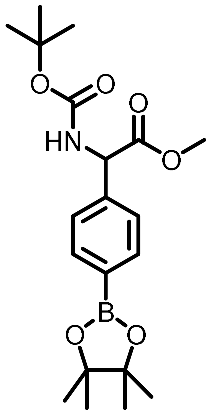

A solution of **59** (3.05 g, 8.86 mmol), bis(pinacolato)diboron (2.7 g, 10.63 mmol) and potassium acetate (2.6 g, 26.58 mmol) in 1,4-dioxane (50 mL) was degassed at RT. $PdCl_2(PPh_3)_2$ (389 mg, 0.53 mmol) was added and the mixture was degassed again, then heated to 80 °C for two hours. It was cooled to RT, diluted with EtOAc and partitioned with brine. The aqueous portion was extracted three times and the combined organics were dried over $MgSO_4$ and concentrated *in vacuo*. The residue was purified by column chromatography on silica gel (0-50% EtOAc in heptane) to yield **60** (1.56 g, 45% yield).

^1H NMR (500 MHz, $CDCl_3$-*d*) δ 7.85 – 7.80 (m, 2H), 7.41 – 7.36 (m, 2H), 5.58 (m, 1H), 5.35 (d, J = 7.5 Hz, 1H), 3.72 (s, 3H), 1.45 (s, 7H), 1.36 (s, 12H), 1.29 (s, 2H)

MS (ESI) for $C_{20}H_{30}BNO_6$ $[M+H]^+$ 292 (fragmentation of Boc group)

methyl 2-((*tert*-butoxycarbonyl)amino)-2-(4-(4-methylthiazol-5-yl)phenyl)acetate 61

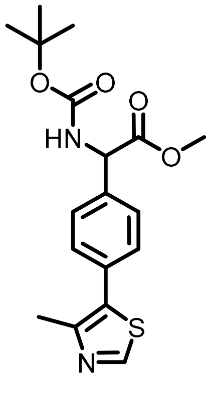

To a degassed suspension of **60** (2.26 g, 5.60 mmol), 5-bromo-4-methylthiazole (997 mg, 5.60 mmol) and Cs_2CO_3 (2.74 g, 8.40 mmol) in 1,4-dioxane (40 mL) and water (4 mL) was added $Pd(dppf)Cl_2 \cdot CH_2Cl_2$ (458 mg, 0.56 mmol). The reaction was degassed again, then heated to 80 °C for 30 minutes under microwave

irradiation. It was cooled to RT and diluted in EtOAc, then separated with brine and the aqueous layer was extracted three times. The combined organics were dried over $MgSO_4$ then concentrated *in vacuo*. The residue was purified by column chromatography on silica gel (0-80% EtOAc in heptane) to yield **61** (1.64 g, 81% yield).

^{1}H NMR (500 MHz, $CDCl_3$-*d*) δ 8.71 (s, 1H), 7.46 (s, 4H), 5.65 (m, 1H), 5.40 (d, J = 7.2 Hz, 1H), 3.78 (s, 3H), 2.56 (s, 3H), 1.47 (s, 9H)

MS (ESI) for $C_{18}H_{22}N_2O_4S$ $[M+H]^+$ 363

***tert*-butyl (2-hydroxy-1-(4-(4-methylthiazol-5-yl)phenyl)ethyl)carbamate 62**

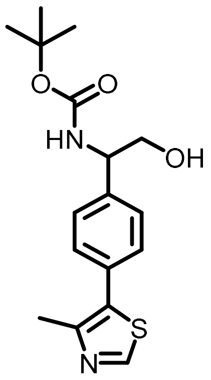

To a solution of **61** (1.89 g, 5.20 mmol) in MeOH (24 mL) was added $NaBH_4$ (787 mg, 20.81 mmol). The mixture was heated to 50 °C and stirred overnight. The reaction was cooled to RT and quenched with 2 M NaOH (24 mL). After 20 min stirring at RT, it was neutralised with careful dropwise addition of 0.5 M aq. HCl. MeOH was removed in vacuo and the remaining aqueous fraction was extracted three times with DCM. The combined organics were dried over $MgSO_4$ and concentrated *in vacuo*. The residue was purified by column chromatography on silica gel (0-100% EtOAc in heptane) to yield **62** (1.44 g, 83% yield).

^{1}H NMR (500 MHz, $CDCl_3$-*d*) δ 8.69 (s, 1H), 7.50 – 7.43 (m, 2H), 7.43 – 7.37 (m, 2H), 5.33 (d, J = 7.2 Hz, 1H), 4.84 (m, 1H), 3.98 – 3.87 (m, 2H), 2.55 (s, 3H), 2.32 (s, 1H), 1.47 (s, 9H)

MS (ESI) for $C_{17}H_{22}N_2O_3S$ $[M+H]^+$ 335

***tert*-butyl 2-(2-((tert-butoxycarbonyl)amino)-2-(4-(4-methylthiazol-5-yl)phenyl)ethoxy)acetate 63**

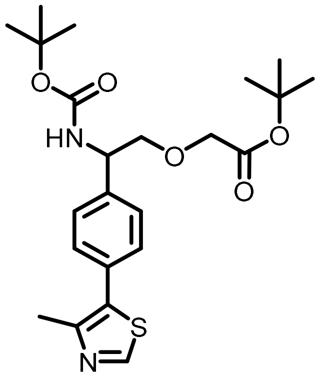

To a suspension of **62** (1.58 g, 4.73 mmol) and tetrabutylammonium hydrogen sulfate (643 mg, 1.89 mmol) in DCM (24 mL) was added 4 M aq. NaOH (24 mL). A solution of *tert*-butyl bromoacetate (839 µL, 5.68 mmol) in DCM (1 mL) was added dropwise. The reaction was stirred for 3 hours at RT. Reaction progress was monitored by LC-MS and successive solutions of *tert*-butyl bromoacetate (839 µL, 5.68 mmol) in DCM (1 mL) were added dropwise until near complete conversion of the starting material. The mixture was diluted with water (10 mL) and extracted three times with DCM. The combined organics were washed with brine, dried over $MgSO_4$ and concentrated *in vacuo*. The residue was purified by column chromatography on silica gel (0-5% MeOH in DCM) to yield **63** (2.03 g, 96% yield).

^{1}H NMR (500 MHz, $CDCl_3$-*d*) δ 8.69 (s, 1H), 7.44 (s, 4H), 5.84 (s, 1H), 4.87 (s, 1H), 3.99 (d, J = 1.8 Hz, 2H), 3.82 (dd, J = 9.5, 4.1 Hz, 1H), 3.76 (s, 1H), 2.56 (s, 3H), 1.52 (s, 9H), 1.41 (s, 9H)

MS (ESI) for $C_{23}H_{32}N_2O_5S$ $[M+H]^+$ 449

methyl 2-(2-((2*S*,4*R*)-1-((*S*)-2-((*tert*-butoxycarbonyl)amino)-3,3-dimethylbutanoyl)-4-hydroxypyrrolidine-2-carboxamido)-2-(4-(4-methylthiazol-5-yl)phenyl)ethoxy)acetate 64

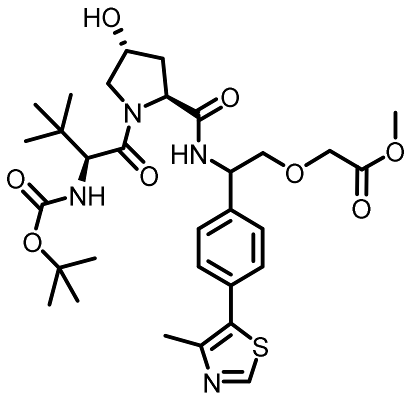

To a solution of **63** (974 mg, 2.17 mmol) in DCM (14 mL) and MeOH (2 mL) at 0 °C was added 4 M HCl in 1,4-dioxane (5.43 mL, 21.71 mmol) and the mixture was left to stir overnight at RT. The solvent was removed *in vacuo* and azeotroped with toluene to give the desired intermediate. The intermediate was suspended in MeOH (14 mL) at 0 °C and $SOCl_2$ (0.95 mL, 13.03 mmol) was added dropwise. The reaction was allowed to warm to RT and stirred for two hours. It was dried in vacuo and azeotroped with toluene to yield the desired intermediate. To a solution of the intermediate, Boc-Hyp-OH (505 mg, 2.18 mmol) and HATU (995 mg, 2.62 mmol) in DMF (5 mL) was added DIPEA (1.13 mL, 6.51 mmol). The mixture was stirred at RT overnight. It was diluted in EtOAc and partitioned with sat. aq. $NaHCO_3$ and extracted three times. The combined organics were washed with brine, dried over $MgSO_4$ and concentrated. The residue was purified by column chromatography on silica gel (0-10% MeOH in DCM) which afforded *tert*-butyl (2S,4R)-4-hydroxy-2-((2-(2-methoxy-2-oxoethoxy)-1-(4-(4-methylthiazol-5-yl)phenyl)ethyl)carbamoyl)pyrrolidine-1-carboxylate in sufficient purity for the next step. It was dissolved in DCM (7 mL) and MeOH (1 mL) at 0 °C and 4 M HCl in 1,4-dioxane (3.27 mL, 13.09 mmol) was added. The mixture was left to stir overnight at RT. The solvent was removed in vacuo and azeotroped with toluene to give the desired amine intermediate. To a solution of the intermediate amine, Boc-Tle-OH (299 mg, 1.29 mmol) and HATU (590 mg, 1.55 mmol) in DMF (6 mL) was added DIPEA (674 µL, 3.87 mmol). The mixture was stirred at RT overnight. It was diluted in EtOAc and partitioned with sat. aq. $NaHCO_3$ and extracted three times. The combined organics were washed with brine, dried over $MgSO_4$ and concentrated. The residue was purified by acidic HPLC (Method 4a) to yield **64** (511 mg, 37% yield).

^{1}H NMR (500 MHz, $CDCl_3$-*d*) δ 8.69 (s, 1H), 8.03 – 7.90 (m, 1H), 7.51 – 7.34 (m, 4H), 5.34 – 5.21 (m, 1H), 5.21 – 5.06 (m, 1H), 4.89 – 4.76 (m, 1H), 4.59 – 4.47 (m, 1H), 4.29 – 4.00 (m, 4H), 3.94 – 3.80 (m, 2H), 3.78 (s, 1.5H), 3.76 (s, 1.5H), 3.73 – 3.56 (m, 1H), 2.60 – 2.34 (m, 1H), 2.55 (s, 1.5H), 2.53 (s, 1.5H), 2.30 – 2.11 (m, 1H), 1.44 (s, 4.5H), 1.43 (s, 4.5H), 1.12 – 0.86 (m, 9H)
MS (ESI) for $C_{31}H_{44}N_4O_8S$ $[M+H]^+$ 633

methyl 2-(2-((2*S*,4*R*)-1-((*S*)-2-(1-fluorocyclopropane-1-carboxamido)-3,3-dimethylbutanoyl)-4-hydroxypyrrolidine-2-carboxamido)-2-(4-(4-methylthiazol-5-yl)phenyl)ethoxy)acetate 65

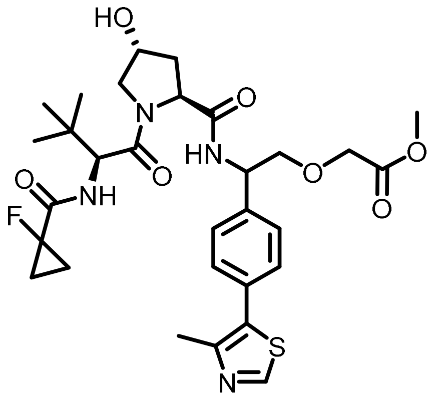

To a solution of **64** (50 mg, 0.79 mmol) in DCM (7 mL) and MeOH (1 mL) at 0 °C was added 4 M HCl in 1,4-dioxane (1.98 mL, 7.90 mmol) and the mixture was left to stir overnight at RT. The solvent was removed in vacuo and azeotroped with toluene to give the desired intermediate. To a solution of the intermediate, 1-fluorocyclopropane-1-carboxylic acid (82 mg, 0.79 mmol) and HATU (360 mg, 0.95 mmol) in DMF (6 mL) was added DIPEA (674 µL, 3.87 mmol). The mixture was stirred at RT for two hours. It was diluted in EtOAc and partitioned with sat. aq. $NaHCO_3$ and extracted three times. The combined organics were washed with brine, dried over $MgSO_4$ and concentrated. The residue was purified by column chromatography on silica gel (0-10% MeOH in DCM) to yield **65** (363 mg, 74% yield)

^{1}H NMR (500 MHz, $CDCl_3$-*d*) δ 8.68 (s, 1H), 7.98 – 7.88 (m, 1H), 7.55 – 7.32 (m, 4H), 7.20 – 7.08 (m, 1H), 5.24 – 5.06 (m, 1H), 4.86 – 4.71 (m, 1H), 4.66 – 4.48 (m, 2H), 4.14 (s, 1H), 4.12 (s, 1H), 4.07 – 3.95 (m, 1H), 3.92 – 3.81 (m, 2H), 3.78 (s, 1.5H), 3.77 (s, 1.5H), 3.58 – 3.41 (m, 1H), 2.54 (s, 1.5H), 2.53 (s, 1.5H), 2.50 – 2.29 (m, 1H), 2.29 – 2.11 (m, 1H), 1.44 – 1.22 (m, 4H), 1.09 (s, 4.5H), 0.95 (s, 4.5H).

MS (ESI) for $C_{30}H_{39}FN_4O_7S$ $[M+H]^+$ 619

(2*S*,4*R*)-1-((*S*)-2-(1-fluorocyclopropane-1-carboxamido)-3,3-dimethylbutanoyl)-4-hydroxy-*N*-(2-(2-hydroxyethoxy)-1-(4-(4-methylthiazol-5-yl)phenyl)ethyl)pyrrolidine-2-carboxamide 66

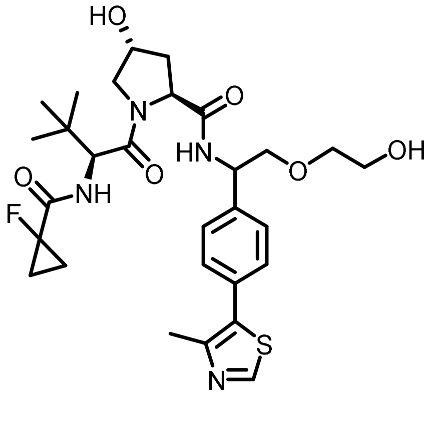

To a solution of **65** (363 mg, 0.59 mmol) in MeOH (10 mL) was added $NaBH_4$ (89 mg, 2.35 mmol) The mixture was heated to 50 °C and stirred overnight. The reaction was cooled and quenched with 2 M aq. NaOH (10 mL). After 20 min stirring at rt, it was neutralised with careful dropwise addition of 1 M aq. HCl. MeOH was removed *in vacuo* and the remaining aqueous mixture was extracted three times with DCM. The combined organics were dried over $MgSO_4$ and concentrated. The residue was purified by acidic reverse phase chromatography (Method 4a) to yield **66** (185 mg, 53% yield).

^{1}H NMR (500 MHz, $CDCl_3$-*d*) δ 8.70 (s, 0.5H), 8.69 (0.5H), 8.18 – 8.11 (m, 1H), 7.89 – 7.81 (m, 1H), 7.49 – 7.34 (m, 4H), 7.22 – 7.07 (m, 1H), 5.19 (t, J = 7.6, 4.8 Hz, 0.5H), 4.87 (t, J = 7.9 Hz, 0.5H), 4.69 – 4.56 (m, 1H), 4.54 – 4.46 (m, 1H), 4.08 – 3.95 (m, 1H), 3.92 – 3.54 (m, 8H), 3.49 (s, 3H), 2.54 (s, 1.5H), 2.53 – 2.47 (m, 1H), 2.51 (s, 1.5H), 2.33 – 2.22 (m, 1H), 2.18 – 2.07 (m, 1H), 1.47 – 1.22 (m, 4H), 1.11 (s, 4.5H), 0.99 (s, 4.5H)

MS (ESI) for $C_{29}H_{39}FN_4O_6S$ $[M+H]^+$ 591

2-(2-((2*S*,4*R*)-1-((*S*)-2-(1-fluorocyclopropane-1-carboxamido)-3,3-dimethylbutanoyl)-4-hydroxypyrrolidine-2-carboxamido)-2-(4-(4-methylthiazol-5-yl)phenyl)ethoxy)ethyl methanesulfonate 67

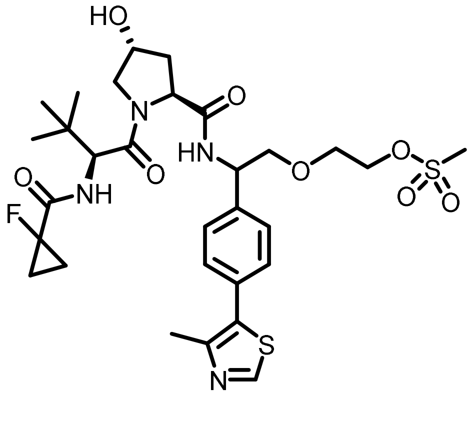

To a solution of **66** (185 mg, 0.31 mmol) in DCM (3 mL) at 0 °C was added DIPEA (153 µL, 0.94 mmol) and followed by MsCl (27 µL, 0.34 mmol). The reaction was allowed to warm to RT and stirred overnight. The

mixture was partitioned between DCM and sat. aq. $NaHCO_3$ and passed over a phase separator. The organic layer was dried over $MgSO_4$ and concentrated. The residue was purified column chromatography on silica gel (0-10% MeOH in DCM) to yield **67** (80 mg, 38% yield).

^{1}H NMR (500 MHz, $CDCl_3$-*d*) δ 8.70 (s, 0.5H), 8.69 (0.5H), 8.18 – 8.11 (m, 1H), 7.89 – 7.81 (m, 1H), 7.49 – 7.34 (m, 4H), 7.22 – 7.07 (m, 1H), 5.19 (t, J = 7.6, 4.8 Hz, 0.5H), 4.87 (t, J = 7.9 Hz, 0.5H), 4.69 – 4.56 (m, 1H), 4.54 – 4.46 (m, 1H), 4.08 – 3.95 (m, 1H), 3.92 – 3.54 (m, 8H), 3.49 (s, 3H), 2.54 (s, 1.5H), 2.53 – 2.47 (m, 1H), 2.51 (s, 1.5H), 2.33 – 2.22 (m, 1H), 2.18 – 2.07 (m, 1H), 1.47 – 1.22 (m, 4H), 1.11 (s, 4.5H), 0.99 (s, 4.5H)

MS (ESI) for $C_{30}H_{41}FN_4O_8S_2$ $[M+H]^+$ 669

(2*S*,4*R*)-*N*-((*R*)-2-(2-(4-(4-bromo-7-cyclopentyl-5-oxo-5,7-dihydrobenzo[4,5]imidazo[1,2-*a*]quinazolin-9-yl)piperidin-1-yl)ethoxy)-1-(4-(4-methylthiazol-5-yl)phenyl)ethyl)-1-((*S*)-2-(1-fluorocyclopropane-1-carboxamido)-3,3-dimethylbutanoyl)-4-hydroxypyrrolidine-2-carboxamide 8

(2*S*,4*R*)-*N*-((*S*)-2-(2-(4-(4-bromo-7-cyclopentyl-5-oxo-5,7-dihydrobenzo[4,5]imidazo[1,2-*a*]quinazolin-9-yl)piperidin-1-yl)ethoxy)-1-(4-(4-methylthiazol-5-yl)phenyl)ethyl)-1-((*S*)-2-(1-fluorocyclopropane-1-carboxamido)-3,3-dimethylbutanoyl)-4-hydroxypyrrolidine-2-carboxamide 24

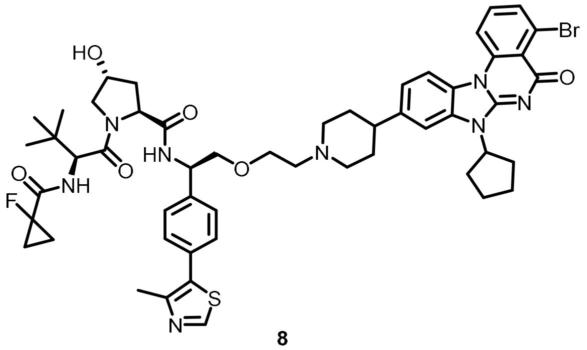

8

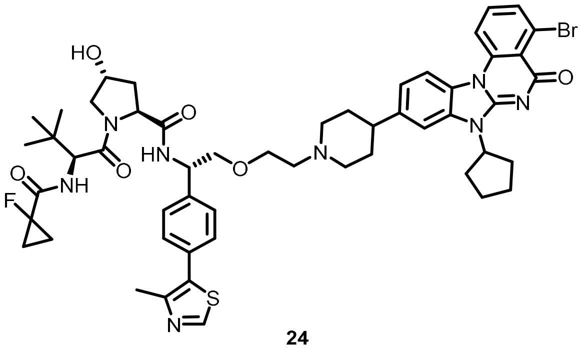

24

To a solution of **67** (80 mg, 0.12 mmol) and **4** (67 mg, 0.14 mmol) in DMF (1.5 mL) was added DIPEA (117 µL, 0.72 mmol) and the reaction was stirred at 80 °C overnight. The mixture was concentrated *in vacuo* and the residue was purified via acidic (Method 4a) then basic reverse phase chromatography (Method 4b) to yield the desired products as a mixture (35 mg, 28% yield). The diastereomers were resolved using SFC purification (Chiralcel OD column, 40% MeOH + 0.1% DEA in water).

8: ^{1}H NMR (600 MHz, DMSO-d_6) δ 8.96 (s, 1H), 8.53 (br d, J = 7.9 Hz, 1H), 8.46 (d, J = 8.4 Hz, 1H), 8.27 (d, J = 8.6 Hz, 1H), 7.80 (d, J = 7.7 Hz, 1H), 7.68 (t, J = 8.2 Hz, 1H), 7.50 (s, 1H), 7.41 - 7.47 (m, 4H), 7.24 (br d, J = 7.5 Hz, 2H), 5.25 (quin, J = 8.7 Hz, 1H), 5.15 (br d, J = 3.3 Hz, 1H), 4.99 - 5.07 (m, 1H), 4.59 (br d, J = 9.2 Hz, 1H), 4.55 (t, J = 8.3 Hz, 1H), 4.29 (br s, 1H), 3.54 - 3.70 (m, 6H), 3.01 (br s, 1H), 2.66 (br d, J = 10.5 Hz, 1H), 2.58 (br s, 1H), 2.44 (s, 3H), 2.21 - 2.32 (m, 3H), 1.85 - 2.19 (m, 8H), 1.67 - 1.81 (m, 7H), 1.27 - 1.46 (m, 2H), 1.11 - 1.26 (m, 2H), 0.95 - 1.02 (m, 9H)

^{13}C NMR (150 MHz, DMSO-d_6) δ 170.3, 168.2, 167.4 (d, CF=20.4 Hz), 163.5, 148.2, 147.2, 142.8, 140.1, 138.2, 132.7, 131.5, 130.4, 129.8, 129.5, 128.1, 126.9, 124.2, 122.2, 120.5, 116.0, 114.8, 113.0, 108.5, 77.5

(d, CF=231.4 Hz), 72.3, 68.2, 58.1, 56.6, 56.0, 55.9, 53.4, 53.3, 51.5, 37.1, 35.5, 27.4, 25.6, 24.0, 15.4, 12.2 (m, CF)

HRMS (m/z) for $C_{53}H_{62}BrFN_8O_6S$ $[M+H]^+$ calculated 1037.37532; obtained 1037.37255

24: ^{1}H NMR (400 MHz, DMSO-*d6*) δ 8.97 (s, 1H), 8.55 (d, J = 8.1 Hz, 1H), 8.48 (d, J = 8.6 Hz, 1H), 8.29 (d, J = 8.6 Hz, 1H), 7.81 (d, J = 7.9 Hz, 1H), 7.65 - 7.75 (m, 1H), 7.50 - 7.59 (m, 3H), 7.43 (d, J = 8.4 Hz, 2H), 7.23 - 7.31 (m, 2H), 5.25 (quin, J = 8.7 Hz, 1H), 5.18 (d, J = 3.8 Hz, 1H), 4.95 - 5.08 (m, 1H), 4.53 - 4.66 (m, 2H), 4.36 (br s, 1H), 3.54 - 3.74 (m, 7H), 2.99 (br d, J = 11.4 Hz, 2H), 2.44 (s, 3H), 2.22 - 2.30 (m, 2H), 1.89 - 2.16 (m, 9H), 1.67 - 1.85 (m, 7H), 1.30 - 1.45 (m, 2H), 1.18 - 1.27 (m, 2H), 0.92 (s, 9H)

HRMS (m/z) for $C_{53}H_{62}BrFN_8O_6S$ $[M+H]^+$ calculated 1037.37532; obtained 1037.37227

Supplementary Figure 22: synthesis of PROTAC 26 (via key intermediates 70 and 74)

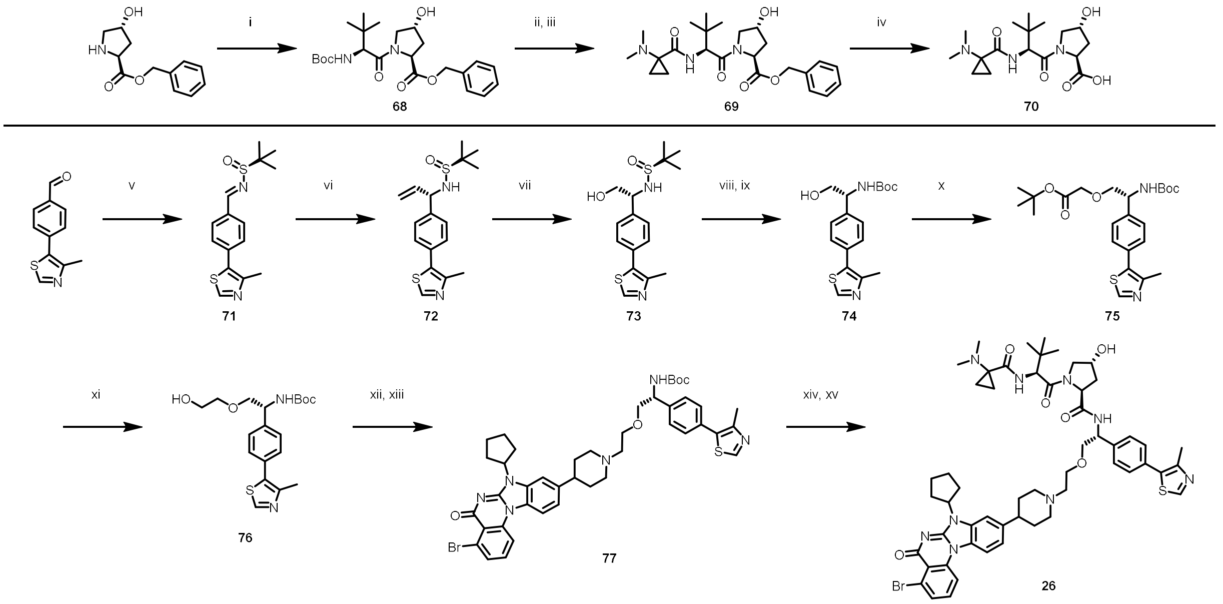

i) *N*-Boc-*L*-*tert*-Leucine, HATU, Et_3N, THF, RT ; ii) HCl, MeOH, RT ; iii) 1-(dimethylamino)cyclopropanecarboxylic acid, HATU, Et_3N, THF, RT ; iv)H_2, Pd/C, MeOH, RT ; v) (*S*)-2-methylpropane-2-sulfinamide, $Ti(O^iPr)_4$, THF, RT ; vi) vinylmagnesium bromide, Me_2Zn, THF, 0 °C – RT then **71**, THF, -78 °C ; vii) O_3, MeOH, -78 °C then $NaBH_4$, RT ; viii) HCl, DCM, 0 °C ; ix) Boc_2O, Et_3N, 1,4-dioxane, 0 °C ; x) *tert*-butyl bromoacetate, tetrabutylammonium hydrogen sulfate, DCM/NaOH, RT ; xi) $LiAlH_4$, THF, 0 °C ; xii) MsCl, Et_3N, DCM, 0 °C ; xiii) **4**, K_2CO_3, KI, NMP/MeCN, 80 °C ; xiv) HCl, MeOH, RT ; xv) **70**, HATU, Et_3N, DMF, RT

(2*S*,4*R*)-benzyl 1-((*S*)-2-((*tert*-butoxycarbonyl)amino)-3,3-dimethylbutanoyl)-4-hydroxypyrrolidine-2-carboxylate 68

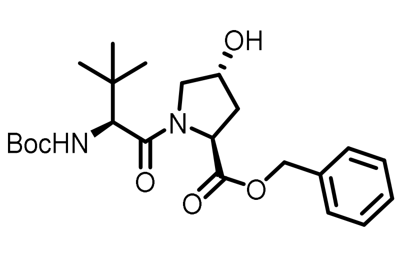

N-Boc-*L*-*tert*-Leucine (145 mg, 0.63 mmol) was dissolved in THF (1.5 mL), then Et_3N (0.33 mL, 2.28 mmol) and HATU (325 mg, 0.86 mmol) were added. The mixture was stirred at RT for 15 minutes. Then (2*S*,4*R*)-benzyl 4-hydroxypyrrolidine-2-carboxylate hydrochloride (150 mg, 0.57 mmol) was added and the mixture was stirred at RT for 2 hours. The reaction mixture was diluted with MeCN/H_2O, filtered through a syringe filter and purified by basic HPLC (Method 3b) to give the desired product **68** (215 mg, 87% yield).

1H NMR (400 MHz, DMSO-*d₆*) δ 7.27 - 7.45 (m, 5H), 6.55 (br d, J = 9.4 Hz, 1H), 5.24 (br s, 1H), 5.07 - 5.17 (m, 2H), 4.41 (t, J = 8.4 Hz, 1H), 4.34 (br s, 1H), 4.15 (br d, J = 9.4 Hz, 1H), 3.59 - 3.70 (m, 2H), 2.09 - 2.18 (m, 1H), 1.91 (ddd, J = 13.1, 8.8, 4.4 Hz, 1H), 1.38 (s, 9H), 0.89 (s, 9H)

MS (ESI) for $C_{23}H_{34}N_2O_6$ $[M+Na]^+$ 435

(2*S*,4*R*)-benzyl 1-((*S*)-2-(1-(dimethylamino)cyclopropanecarboxamido)-3,3-dimethylbutanoyl)-4-hydroxypyrrolidine-2-carboxylate 69

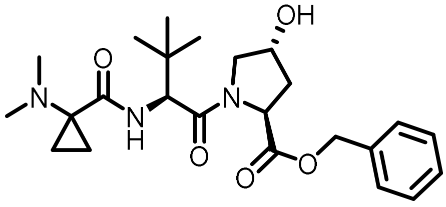

68 (215 mg, 0.50 mmol) was taken up in MeOH (2.0 mL) and concentrated HCl (1.0 mL, 11.65 mmol) was added. The mixture was stirred at RT for 2 hours. The solvents were removed *in vacuo* and the crude (2*S*,4*R*)-benzyl 1-((*S*)-2-amino-3,3-dimethylbutanoyl)-4-hydroxypyrrolidine-2-carboxylate hydrochloride was taken to the next step without further purification with an assumed quantitative yield.

1-(dimethylamino)cyclopropanecarboxylic acid (50 mg, 0.39 mmol) was dissolved in THF (1.5 mL) and Et_3N (0.29 mL, 1.97 mmol) and HATU (375 mg, 0.99 mmol) were added. The mixture was stirred at RT for 15 minutes. Then the crude amine (137 mg, 0.31 mmol) was added and it was stirred at RT overnight. The reaction mixture was diluted with MeCN/H_2O, filtered through a syringe filter and purified by basic prep HPLC (Method 3b) to give the desired product **69** (137 mg, 62% yield over two steps).

^{1}H NMR (400 MHz, DMSO-*d₆*) δ 8.11 (br d, J = 9.6 Hz, 1H), 7.30 - 7.43 (m, 5H), 5.26 (d, J = 3.5 Hz, 1H), 5.12 (s, 2H), 4.48 (d, J = 9.9 Hz, 1H), 4.42 (t, J = 8.5 Hz, 1H), 4.34 (br s, 1H), 3.64 - 3.70 (m, 1H), 3.45 - 3.50 (m, 1H), 3.34 (br d, J = 6.1 Hz, 1H), 2.17 (s, 6H), 1.92 (ddd, J = 13.0, 9.2, 4.2 Hz, 1H), 0.86 - 0.97 (m, 13H)

MS (ESI) for $C_{24}H_{35}N_3O_5$ $[M+H]^+$ 446

(2*S*,4*R*)-1-((*S*)-2-(1-(dimethylamino)cyclopropanecarboxamido)-3,3-dimethylbutanoyl)-4-hydroxypyrrolidine-2-carboxylic acid 70

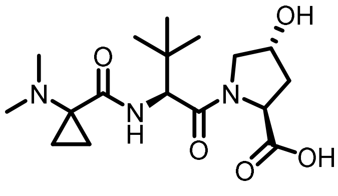

69 (137 mg, 0.31 mmol) was dissolved in MeOH (2.5 mL) and hydrogenated through a cartridge of Pd/C 10% in a Thales Nano H-Cube flow reactor. It was circled for 1 hour (flow rate: 1 mL/min; temperature: RT; pressure: full H_2). The solvent was removed *in vacuo* to give the desired crude product which was taken to the next step without further purification: **70** (103 mg, 94% yield).

MS (ESI) for $C_{17}H_{29}N_3O_5$ $[M+H]^+$ 356

(*S*)-2-methyl-*N*-(4-(4-methylthiazol-5-yl)benzylidene)propane-2-sulfinamide 71

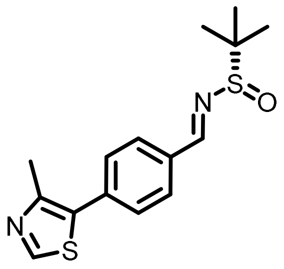

To a solution of **53** (4.46 g, 21.9 mmol) and (*S*)-2-methylpropane-2-sulfinamide (2.66 g, 21.9 mmol) in THF (50 mL) was slowly added $Ti(O^iPr)_4$ (8.0 mL, 26.3 mmol) and the reaction was stirred at RT overnight. The mixture was concentrated *in vacuo* and then diluted with EtOAc and partitioned with brine. The mixture was filtered over Celite and the organic layer was separated with water. The aqueous layer was extracted three times and the combined organics were dried over $MgSO_4$ and concentrated. The residue was purified by column chromatography on silica gel (0-50% EtOAc in heptane) to yield **71** (4.64 g, 83% yield).

^{1}H NMR (400 MHz, $CDCl_3$) δ 8.76 (s, 1H), 8.64 (s, 1H), 7.98 – 7.90 (m, 2H), 7.63 – 7.55 (m, 2H), 2.61 (s, 3H), 1.31 (s, 9H)

MS (ESI) for $C_{15}H_{18}N_2OS_2$ $[M+H]^+$ 307

(*S*)-2-methyl-*N*-((*S*)-1-(4-(4-methylthiazol-5-yl)phenyl)allyl)propane-2-sulfinamide 72

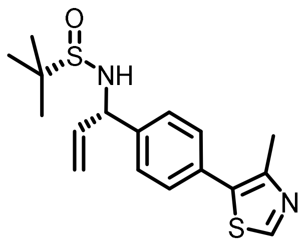

Vinylmagnesium bromide (1 M in THF, 48.9 mL, 48.9 mmol) was added to Me_2Zn (1 M in toluene, 55.5 mL, 55.5 mmol) at 0 °C. The mixture was stirred at RT for 15 minutes. The organozincate solution was then added dropwise to a solution of **71** (5.00 g, 16.3 mmol) in THF (100 mL) at -78 °C. The mixture was stirred at -78 °C for 1 hour. It was reverse quenched in cold sat. aq. NH_4Cl (100 mL) and extracted with EtOAc twice. The combined organics were washed with brine, dried over $MgSO_4$ and concentrated *in vacuo*. The residue was triturated with diethyl ether to yield **72** (6.0 g, 55% yield, dr 9:1).

^{1}H NMR (400 MHz, DMSO-d_6) δ 9.00 (s, 1H), 7.41 - 7.52 (m, 4H), 5.94 (ddd, J = 17.2, 10.1, 7.3 Hz, 1H), 5.79 (d, J = 6.1 Hz, 1H), 5.28 (dt, J = 17.0, 1.3 Hz, 1H), 5.15 (d, J = 10.1 Hz, 1H), 4.91 (t, J = 6.6 Hz, 1H), 2.46 (s, 3H), 1.16 (s, 9H)

MS (ESI) for $C_{17}H_{22}N_2OS_2$ [M+H]$^+$ 335

(*S*)-*N*-((*R*)-2-hydroxy-1-(4-(4-methylthiazol-5-yl)phenyl)ethyl)-2-methylpropane-2-sulfinamide 73

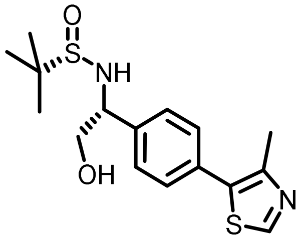

A stirred solution of **72** (2.00 g, 5.98 mmol) in MeOH (40 mL) at 70 °C was purged with ozone gas for 40 minutes. Then $NaBH_4$ (678 mg, 17.9 mmol) was added portionwise and the mixture was slowly allowed to reach RT. The solvent was removed *in vacuo* and the residue was partitioned between water and EtOAc. The aqueous layer was extracted with EtOAc twice. The combined organic layers were dried over Na_2SO_4 and concentrated *in vacuo*. The crude was purified by silica gel column chromatography (50-100% EtOAc in petroleum ether) to yield **73** (900 mg, 45% yield) as a single diastereoisomer.

^{1}H NMR (400 MHz, $CDCl_3$-*d*) δ 8.68 (s, 1H), 7.45 (s, 4H), 4.54 - 4.63 (m, 1H), 4.02 (br d, J = 5.9 Hz, 1H), 3.96 (td, J = 6.0, 2.7 Hz, 2H), 2.74 (br t, J = 6.6 Hz, 1H), 2.54 (s, 3H), 1.26 (s, 9H)

MS (ESI) for $C_{16}H_{22}N_2O_2S_2$ [M+H]$^+$ 339

***tert*-butyl (*R*)-(2-hydroxy-1-(4-(4-methylthiazol-5-yl)phenyl)ethyl)carbamate 74**

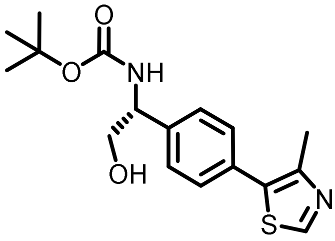

To a stirred solution of **73** (900 mg, 2.66 mmol) in DCM (10 mL) was added HCl (4 M in 1,4-dioxane, 2.0 mL, 8.00 mmol) at 0 °C and the mixture was stirred at RT for 2H. The solvents were removed in vacuo and the residue was triturated with n-pentane to give the crude amine, which was used without further purification. The crude amine (600 mg, 2.22 mmol) was taken up in 1,4-dioxane (8 mL) and water (8 mL) and cooled to 0 °C. Boc_2O (1.45 g, 6.65 mmol) and Et_3N (1.60 mL, 11.1 mmol) were added and the mixture was stirred at RT for 16H. It was poured into ice water and extracted with EtOAc twice. The combined organic layers were washed with brine, dried over Na_2SO_4 and concentrated *in vacuo*. The residue was triturated with pentane to give the **74** (700 mg, 95% yield).

^{1}H NMR (400 MHz, DMSO-*d*$_6$) δ 8.98 (s, 1H), 7.41 - 7.47 (m, 2H), 7.36 - 7.41 (m, 2H), 7.28 (br d, J = 7.6 Hz, 1H), 4.82 (t, J = 5.8 Hz, 1H), 4.46 - 4.67 (m, 1H), 3.52 (t, J = 6.2 Hz, 2H), 2.46 (s, 3H), 1.38 (s, 9H)

MS (ESI) for $C_{17}H_{22}N_2O_3S$ [M+H]$^+$ 335

tert-butyl (_R_)-2-(2-((_tert_-butoxycarbonyl)amino)-2-(4-(4-methylthiazol-5-yl)phenyl)ethoxy)acetate 75

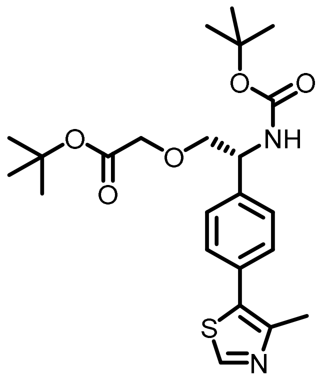

74 (200 mg, 0.60 mmol) and tetrabutylammonium hydrogen sulfate (81 mg, 0.24 mmol) were suspended in DCM (4.0 mL) and 4 M NaOH (3.0 mL) was added. Then *tert*-butyl bromoacetate (194 µL, 1.32 mmol), dissolved in 0.5 mL DCM, was added. The mixture was stirred at RT vigorously for 2 hours. The reaction mixture was diluted with water and extracted with DCM three times. The combined organic layers were passed through a phase separator cartridge and concentrated *in vacuo*. The crude was purified by silica gel column chromatography (0-5% MeOH in DCM) to give **75** (242 mg, 90% yield).

^{1}H NMR (500 MHz, DMSO-d_6) δ 8.99 (s, 1H), 7.35 - 7.51 (m, 5H), 4.70 - 4.81 (m, 1H), 3.95 - 4.09 (m, 2H), 3.55 - 3.69 (m, 2H), 2.46 (s, 3H), 1.42 (s, 9H), 1.38 (s, 9H)

MS (ESI) for $C_{23}H_{32}N_2O_5S$ $[M+H]^+$ 449

(_R_)-_tert_-butyl (2-(2-hydroxyethoxy)-1-(4-(4-methylthiazol-5-yl)phenyl)ethyl)carbamate 76

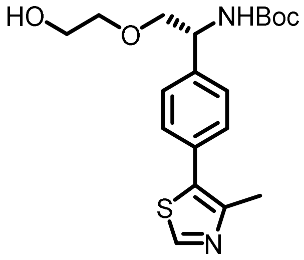

75 (403 mg, 0.90 mmol) was dissolved in dry THF (8.0 mL) and cooled to 0 °C in an ice bath. Then $LiAlH_4$ (2 M in THF, 0.67 mL, 1.35 mmol) was added slowly. The reaction mixture was stirred at 0 °C for 2 hours and cautiously quenched with water at 0 °C. It was diluted with DCM and water and filtered through a Celite pad. The layers were separated. The aqueous layer was extracted with DCM. The combined organics were dried over $MgSO_4$ and concentrated *in vacuo*. The residue was taken to the next step without further purification: **76** (334 mg, 98% yield).

MS (ESI) for $C_{19}H_{26}N_2O_4S$ $[M+H^+]$ 379

(*R*)-*tert*-butyl (2-(2-(4-(4-bromo-7-cyclopentyl-5-oxo-5,7-dihydrobenzo[4,5]imidazo[1,2-*a*]quinazolin-9-yl)piperidin-1-yl)ethoxy)-1-(4-(4-methylthiazol-5-yl)phenyl)ethyl)carbamate 77

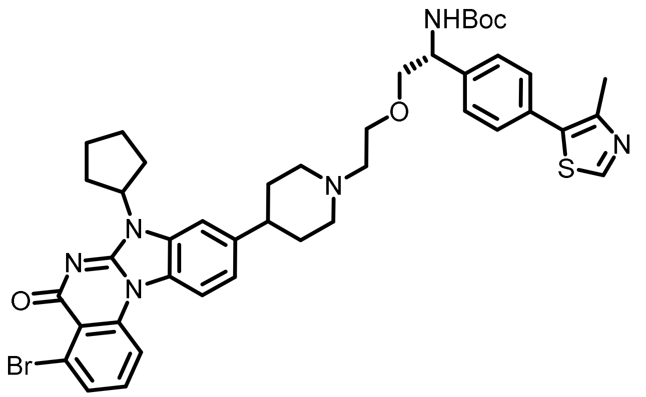

76 (173 mg, 0.46 mmol) and Et_3N (76 µL, 0.55 mmol) were dissolved in DCM (2.0 mL) and cooled to 0 °C in an ice bath. Then MsCl (38 µL, 0.48 mmol) was added slowly. The reaction was allowed to reach RT and stirred for 1 hour. The reaction was quenched with several drops of sat. aq. $NaHCO_3$ solution and diluted with water. It was extracted with DCM twice. The combined organics were washed with water, dried over $MgSO_4$ and concentrated *in vacuo*. The crude product was taken to the next step without further purification (203 mg, 97% yield).

The crude mesylate (100 mg, 0.18 mmol), **4** (112 mg, 0.20 mmol), K_2CO_3 (49 mg, 0.36 mmol) and KI (57 mg, 0.34 mmol) were suspended in NMP (2.0 mL). The mixture was degassed with argon for 2 minutes, then stirred at 80 °C. The mixture became a gel, therefore MeCN (1.0 mL) was added after 5 minutes and stirring was continued at 80 °C for 17 hours. The reaction mixture was diluted with DCM and half sat. aq. NH_4Cl solution. The layers were separated and the aqueous layer was extracted with DCM. The organic layer was dried over $MgSO_4$ and concentrated *in vacuo*. The crude was purified by basic prep HPLC (Method 3b) to give the desired product, **77** (134 mg, 91% yield).

^1H NMR (400 MHz, DMSO-d_6) δ 8.97 (s, 1H), 8.48 (d, J = 8.6 Hz, 1H), 8.29 (d, J = 8.6 Hz, 1H), 7.80 (d, J = 7.9 Hz, 1H), 7.68 (t, J = 8.1 Hz, 1H), 7.47 - 7.53 (m, 2H), 7.45 (s, 4H), 7.27 (d, J = 8.4 Hz, 1H), 5.25 (quin, J = 8.9 Hz, 1H), 4.69 - 4.86 (m, 1H), 3.49 - 3.62 (m, 4H), 2.99 (br d, J = 9.1 Hz, 2H), 2.45 (s, 3H), 2.22 - 2.30 (m, 2H), 1.95 - 2.13 (m, 6H), 1.69 - 1.81 (m, 6H), 1.38 (s, 9H)

MS (ESI) for $C_{43}H_{49}BrN_6O_4S$ $[M+H]^+$ 825

(2*S*,4*R*)-*N*-((*R*)-2-(2-(4-(4-bromo-7-cyclopentyl-5-oxo-5,7-dihydrobenzo[4,5]imidazo[1,2-*a*]quinazolin-9-yl)piperidin-1-yl)ethoxy)-1-(4-(4-methylthiazol-5-yl)phenyl)ethyl)-1-((*S*)-2-(1-(dimethylamino)cyclopropanecarboxamido)-3,3-dimethylbutanoyl)-4-hydroxypyrrolidine-2-carboxamide 26

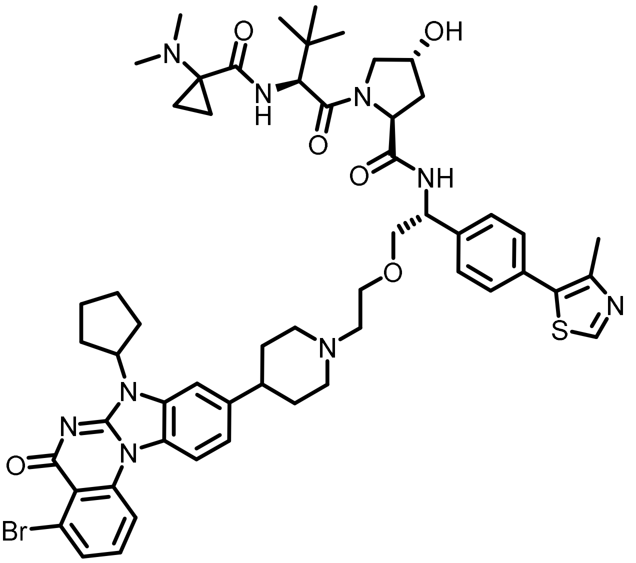

77 (86 mg, 0.10 mmol) was dissolved in MeOH (1.5 mL) and conc. HCl (0.75 mL) was added. The mixture was stirred at RT for 2 hours. The solvents were removed *in vacuo* and the crude amine was used in the next step without further purification. Acid **70** (18 mg, 0.052 mmol) was dissolved in DMF (0.5 mL) and Et_3N (20 µL, 0.14 mmol) and HATU (39 mg, 0.10 mmol) were added. The mixture was stirred at RT for 15 minutes. Then the crude amine (25 mg, 0.034 mmol) was added and the mixture stirred at RT for 12 hours.

The reaction mixture was diluted with MeCN/H2O, filtered through a syringe filter and purified by basic prep HPLC (Method 3b) to give the desired product, **26** (17 mg, 46% yield).

^{1}H NMR (500 MHz, DMSO-d_6) δ 8.97 (s, 1H), 8.52 (d, J = 8.2 Hz, 1H), 8.47 (d, J = 8.5 Hz, 1H), 8.28 (d, J = 8.5 Hz, 1H), 8.12 (d, J = 9.8 Hz, 1H), 7.80 (d, J = 7.9 Hz, 1H), 7.68 (t, J = 8.2 Hz, 1H), 7.52 (s, 1H), 7.41 - 7.49 (m, 4H), 7.26 (d, J = 7.9 Hz, 1H), 5.25 (quin, J = 8.7 Hz, 1H), 5.15 (d, J = 3.5 Hz, 1H), 5.00 - 5.08 (m, 1H), 4.52 (t, J = 8.2 Hz, 1H), 4.49 (d, J = 9.8 Hz, 1H), 4.29 (br s, 1H), 3.52 - 3.70 (m, 6H), 2.97 (br d, J = 9.5 Hz, 2H), 2.61 - 2.71 (m, 2H), 2.45 (s, 3H), 2.27 (br dd, J = 12.3, 8.2 Hz, 3H), 2.16 (s, 6H), 1.97 - 2.12 (m, 7H), 1.69 - 1.82 (m, 7H), 0.88 - 1.05 (m, 13H)

^{13}C NMR (150 MHz, DMSO-d_6) δ 172.7, 171.5, 169.8, 164.7, 152.0, 149.3, 148.2, 144.0, 141.2, 139.2, 133.9, 132.7, 131.6, 130.8, 130.5, 129.1, 127.9, 125.3, 123.3, 121.7, 117.0, 115.8, 114.1, 109.6, 73.3, 69.3, 68.8, 59.1, 57.7, 57.0, 56.8, 54.5, 54.4, 52.6, 47.9, 42.1, 38.1, 36.6, 33.7, 28.4, 26.8, 26.7, 25.0, 16.4, 12.8, 10.8

HRMS (m/z) for $C_{55}H_{68}BrN_9O_6S$ $[M+H]^+$ calculated 1062.42694; obtained 1062.42576

Supplementary Figure 23: synthesis of PROTACs 9 and 10

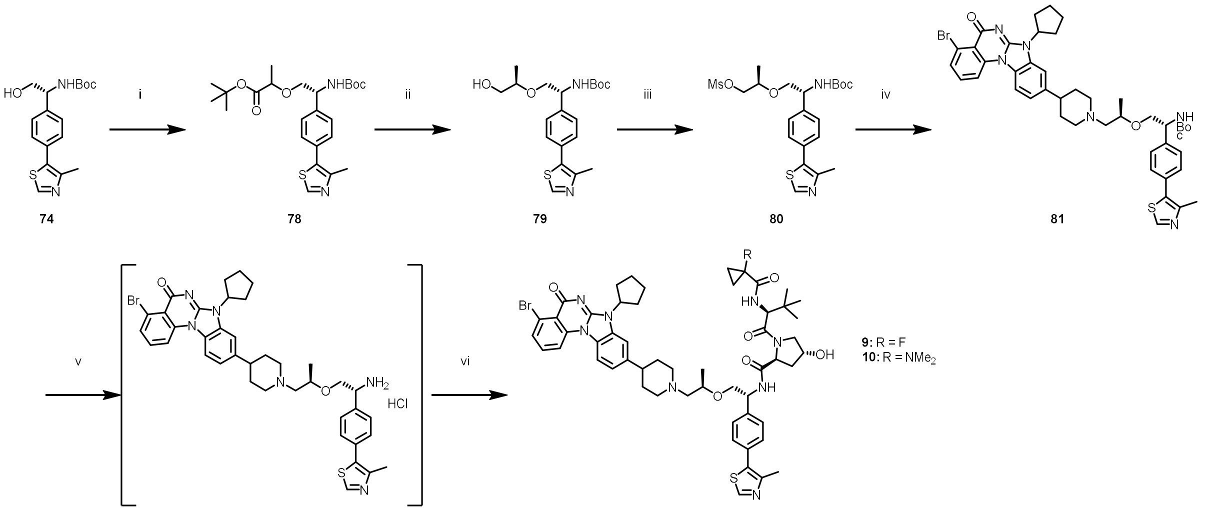

i) *tert*-butyl 2-bromopropanoate, tetrabutylammonium hydrogen sulfate, DCM, NaOH, RT ; ii) $LiAlH_4$, THF, 0 °C ; iii) MsCl, Et_3N, DCM, 0 °C ; iv) **4**, KI, DIPEA, NMP, 85 °C ; v) HCl, MeOH, 40 °C ; vi) **acid 52** or **70**, HATU, Et_3N, DMF, RT.

tert-butyl 2-((_R_)-2-((_tert_-butoxycarbonyl)amino)-2-(4-(4-methylthiazol-5-yl)phenyl)ethoxy)propanoate 78

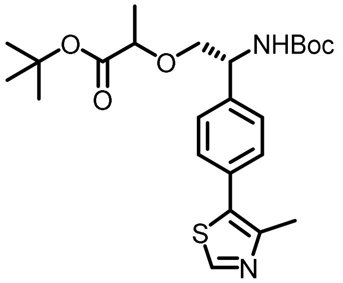

74 (1.00 g, 2.40 mmol) and tetrabutylammonium hydrogen sulfate (0.33 g, 0.96 mmol) were dissolved in DCM (10.0 mL) and 4 M NaOH (15.0 mL) was added. Then *tert*-butyl 2-bromopropanoate (2.05 g, 9.61 mmol) dissolved in DCM (5.0 mL) was added and the reaction mixture was stirred vigorously at RT for 16 hours. Additional *tert*-butyl 2-bromopropanoate (0.50 g, 2.34 mmol) was added and the RM was stirred vigorously at RT for another 27 hours. The reaction mixture was diluted with water. The layers were separated and the aq. layer was extracted with DCM twice. The combined organic layers were concentrated *in vacuo*. The crude residue was purified by column chromatography on silica gel (0-3% MeOH in DCM) to give the desired product, **78** (1.23 g, quant. yield).

^{1}H NMR (400 MHz, DMSO-d_6) δ 8.99 (s, 1H), 7.37 - 7.59 (m, 5H), 4.67 - 4.82 (m, 1H), 3.94 (qd, J = 6.8, 2.0 Hz, 1H), 3.58 - 3.69 (m, 1H), 3.44 - 3.52 (m, 1H), 2.46 (s, 3H), 1.35 - 1.44 (m, 18H), 1.20 - 1.25 (m, 3H)

MS (ESI) for $C_{24}H_{34}N_2O_5S$ $[M+H]^+$ 463

tert-butyl ((R)-2-(((R)-1-hydroxypropan-2-yl)oxy)-1-(4-(4-methylthiazol-5-yl)phenyl)ethyl)carbamate 79

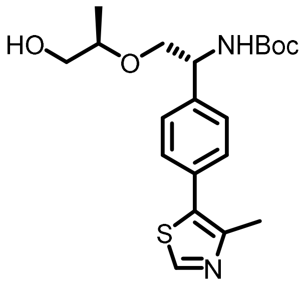

78 (0.80 g, 1.73 mmol) was dissolved in THF (10 mL) and cooled to 0 °C. Then $LiAlH_4$ (2 M in THF, 2.59 mL, 5.19 mmol) was added dropwise over a period of 5 minutes. The reaction mixture was stirred at 0 - 5 °C. After 2 hours, additional $LiAlH_4$ (2 M in THF, 2.59 ml, 5.19 mmol) was added dropwise at 0 °C and the reaction mixture was stirred at 0 °C for 14 hours. The reaction mixture was cooled to -65 °C and carefully quenched by addition of EtOAc and allowed to warm to 0 °C. Then water and sat. Na-K-Tartrate solution were added and the mixture stirred for 20 minutes at RT. The mixture was diluted with EtOAc, filtered over a pad of Celite and rinsed with EtOAc. The layers were separated, the organic layer was dried over $MgSO_4$ and concentrated *in vacuo*. The residue was purified by basic prep.HPLC (Method 3b) to give the desired product as diastereomeric mixture (387 mg, 57% yield). The diastereoisomers were separated using SFC (Chiralpak AD, 30% MeOH) to give the desired diastereoisomer, **79** (184 mg, 48% recovery).

^{1}H NMR (500 MHz, DMSO-d_6) δ 8.99 (s, 1H), 7.44 - 7.46 (m, 2H), 7.40 - 7.43 (m, 3H), 4.70 (br d, J = 5.7 Hz, 1H), 4.60 (br t, J = 5.7 Hz, 1H), 3.55 - 3.64 (m, 1H), 3.43 - 3.49 (m, 1H), 3.33 (br d, J = 5.7 Hz, 1H), 3.23 - 3.30 (m, 1H), 2.46 (s, 4H), 1.39 (s, 9H), 1.01 (d, J = 6.3 Hz, 3H)

MS (ESI) for $C_{20}H_{28}N_2O_4S$ $[M+H]^+$ 393

(R)-2-((R)-2-((tert-butoxycarbonyl)amino)-2-(4-(4-methylthiazol-5-yl)phenyl)ethoxy)propyl methanesulfonate 80

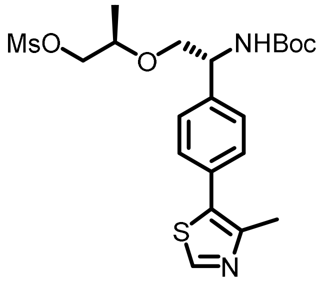

79 (387 mg, 0.99 mmol) was dissolved in DCM (10 mL) and Et_3N (0.41 mL, 2.99 mmol) was added. The mixture was cooled to 0 °C in an ice bath. MsCl (0.15 mL, 1.97 mmol) was added slowly and the reaction mixture was stirred at 0 °C for 10 minutes. The reaction was quenched with sat. $NaHCO_3$ solution and diluted with DCM. The mixture was stirred for 20 minutes. The layers were separated and the aqueous layer was extracted with DCM twice. The combined organic layers were dried over $MgSO_4$ and

concentrated *in vacuo*. The residue was purified by column chromatography on silica gel (0-5% MeOH in DCM) to give the desired product, **80** (441 mg, 95% yield).

^{1}H NMR (400 MHz, DMSO-d_6) δ 8.99 (s, 1H), 7.34 - 7.55 (m, 5H), 4.68 - 4.78 (m, 1H), 4.15 - 4.23 (m, 1H), 4.04 - 4.14 (m, 1H), 3.70 - 3.84 (m, 1H), 3.59 (d, J = 6.6 Hz, 2H), 3.13 (s, 3H), 2.45 (s, 3H), 1.38 (s, 9H), 1.12 (d, J = 6.3 Hz, 3H)

MS (ESI) for $C_{21}H_{30}N_2O_6S_2$ $[M+H]^+$ 471

***tert*-butyl ((*R*)-2-(((*R*)-1-(4-(4-bromo-7-cyclopentyl-5-oxo-5,7-dihydrobenzo[4,5]imidazo[1,2-*a*]quinazolin-9-yl)piperidin-1-yl)propan-2-yl)oxy)-1-(4-(4-methylthiazol-5-yl)phenyl)ethyl)carbamate 81**

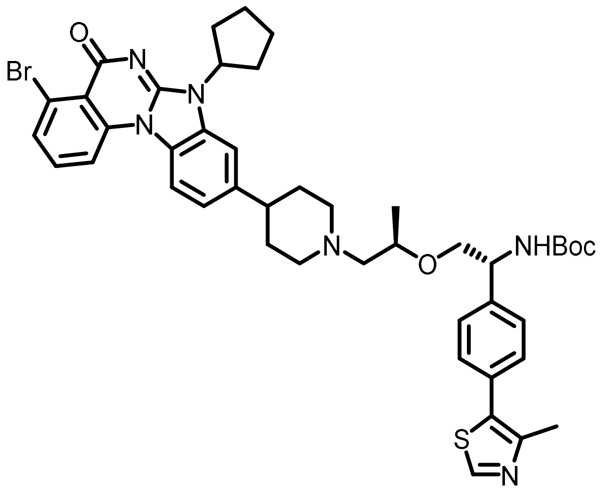

80 (213 mg, 0.45 mmol), **4** (221 mg, 0.45 mmol), KI (150 mg, 0.91 mmol) and DIPEA (237 µL, 1.36 mmol) were suspended in NMP (4.0 mL). The reaction mixture was degassed with argon for 5 minutes, then stirred at 85 °C for 18 hours. The reaction mixture was diluted with DCM and half sat. NH_4Cl solution. The layers were separated via a phase separating cartridge. The aqueous layer was extracted with DCM twice. The combined organic layers were concentrated *in vacuo* and the crude was purified by basic prep HPLC (Method 3b) to give the desired product, **81** (201 mg, 53% yield).

^{1}H NMR (500 MHz, DMSO-d_6) δ 8.96 (s, 1H), 8.47 (d, J = 8.5 Hz, 1H), 8.27 (d, J = 8.5 Hz, 1H), 7.80 (d, J = 7.6 Hz, 1H), 7.66 - 7.71 (m, 1H), 7.51 (br s, 1H), 7.45 (s, 5H), 7.24 (br d, J = 8.5 Hz, 1H), 5.18 - 5.28 (m, 1H), 4.67 - 4.78 (m, 1H), 3.62 - 3.69 (m, 2H), 3.56 (br dd, J = 9.9, 6.1 Hz, 1H), 2.94 (br dd, J = 26.6, 10.2 Hz, 2H), 2.63 - 2.69 (m, 2H), 2.43 (s, 3H), 2.25 (br d, J = 7.9 Hz, 3H), 1.95 - 2.11 (m, 7H), 1.73 (br s, 5H), 1.38 (s, 9H), 1.10 (d, J = 6.0 Hz, 3H)

MS (ESI) for $C_{44}H_{51}BrN_6O_4S$ $[M+H]^+$ 839

(2*S*,4*R*)-*N*-((*R*)-2-(((*R*)-1-(4-(4-bromo-7-cyclopentyl-5-oxo-5,7-dihydrobenzo[4,5]imidazo[1,2-*a*]quinazolin-9-yl)piperidin-1-yl)propan-2-yl)oxy)-1-(4-(4-methylthiazol-5-yl)phenyl)ethyl)-1-((*S*)-2-(1-fluorocyclopropanecarboxamido)-3,3-dimethylbutanoyl)-4-hydroxypyrrolidine-2-carboxamide 9

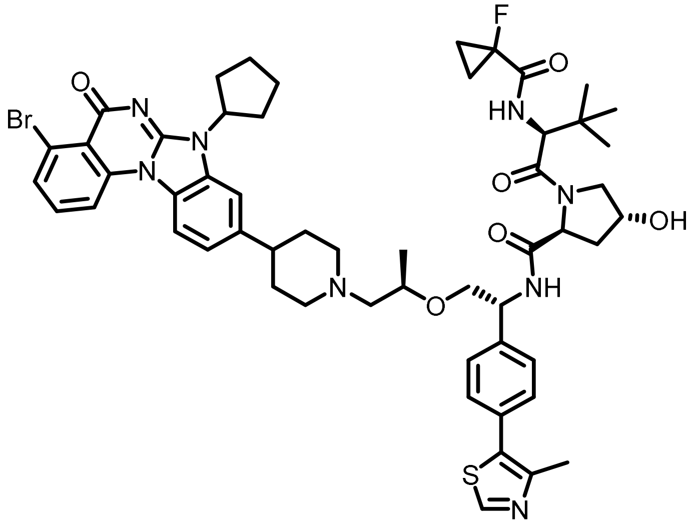

80 195 mg, 0.23 mmol) was dissolved in MeOH (4.0 mL) and 4 M HCl in dioxane (1.0 mL, 4.00 mmol) was added. The mixture was stirred at 40 °C for 1 hour. The reaction mixture was dried in vacuo and the crude amine was taken to the next step without further purification. **52** (99 mg, 0.30 mmol) and HATU (132 mg, 0.35 mmol) were dissolved in DMF (3.0 mL) and Et_3N (0.67 mL, 4.64 mmol) was added. The mixture was stirred at RT for 20 minutes. Then the activated acid was added to the crude amine (180 mg, 0.23 mmol) and the mixture was stirred at RT for 20 minutes. It was diluted with DCM and half sat. NH_4Cl solution. The layers were separated and the aqueous layer was extracted with DCM twice. The combined organic layers were concentrated *in vacuo* and the residue was purified by basic HPLC (Method 3b) to give the desired product, **9** (196 mg, 80% yield).

^{1}H NMR (600 MHz, DMSO-d_6) δ 8.95 (s, 1H), 8.45 (dd, J = 8.2, 3.9 Hz, 2H), 8.25 (d, J = 8.6 Hz, 1H), 7.80 (d, J = 7.7 Hz, 1H), 7.68 (t, J = 8.2 Hz, 1H), 7.49 (s, 1H), 7.42 - 7.48 (m, 4H), 7.18 - 7.28 (m, 2H), 5.24 (quin, J = 8.8 Hz, 1H), 5.15 (d, J = 3.7 Hz, 1H), 4.96 (q, J = 6.8 Hz, 1H), 4.59 (d, J = 9.2 Hz, 1H), 4.53 (t, J = 8.3 Hz, 1H), 4.29 (br s, 1H), 3.74 (dd, J = 9.8, 6.1 Hz, 1H), 3.54 - 3.67 (m, 4H), 2.92 (br dd, J = 28.6, 10.8 Hz, 2H), 2.59 - 2.68 (m, 1H), 2.43 (s, 3H), 2.22 - 2.30 (m, 4H), 1.95 - 2.14 (m, 7H), 1.64 - 1.81 (m, 7H), 1.26 - 1.42 (m, 2H), 1.16 - 1.24 (m, 2H), 1.11 (d, J = 6.2 Hz, 3H), 0.98 (s, 9H)

^{13}C NMR (150 MHz, DMSO-d_6) δ 170.3, 168.2, 167.4 (d, CF=19.1 Hz), 163.5, 150.9, 148.2, 147.1, 142.9, 140.3, 138.2, 132.7, 131.5, 130.5, 129.9, 129.5, 128.0, 127.2, 127.0, 124.2, 122.2, 120.5, 116.0, 114.7, 112.9, 108.5, 77.5 (d, CF=232.7 Hz), 72.9, 70.3, 68.2, 66.3, 63.1, 58.1, 56.0, 55.9, 54.1, 53.5, 53.3, 52.1, 41.1, 37.1, 35.5, 32.8, 27.4, 25.7, 25.6, 24.0, 18.0, 15.4, 12.2 (m, CF)

HRMS (m/z) for $C_{54}H_{64}BrFN_8O_6S$ $[M+H]^+$ calculated 1051.39097; obtained 1051.38766

(2*S*,4*R*)-*N*-((*R*)-2-(((*R*)-1-(4-(4-bromo-7-cyclopentyl-5-oxo-5,7-dihydrobenzo[4,5]imidazo[1,2-*a*]quinazolin-9-yl)piperidin-1-yl)propan-2-yl)oxy)-1-(4-(4-methylthiazol-5-yl)phenyl)ethyl)-1-((*S*)-2-(1-(dimethylamino)cyclopropanecarboxamido)-3,3-dimethylbutanoyl)-4-hydroxypyrrolidine-2-carboxamide 10

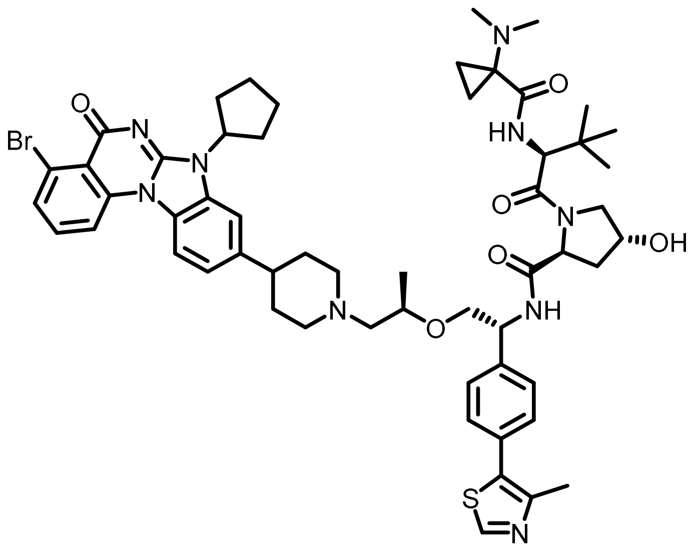

70 (59 mg, 0.14 mmol) and HATU (60 mg, 0.16 mmol) were dissolved in DMF (2.0 mL) and Et_3N (0.31 mL, 2.14 mmol) was added. The mixture was stirred at RT for 20 minutes. Then the activated acid was added to **81** (83 mg, 0.11 mmol) and the mixture was stirred at RT for 20 minutes. The reaction mixture was diluted with MeCN/water, filtered and purified by basic HPLC (Method 3b) to give the desired product, **10** (71 mg, 62% yield).

^{1}H NMR (600 MHz, DMSO-*d$_6$*) δ 8.95 (s, 1H), 8.46 (d, J = 8.4 Hz, 1H), 8.43 (d, J = 7.9 Hz, 1H), 8.24 (d, J = 8.6 Hz, 1H), 8.11 (d, J = 9.9 Hz, 1H), 7.80 (d, J = 7.9 Hz, 1H), 7.68 (t, J = 8.2 Hz, 1H), 7.50 (s, 1H), 7.45 (s, 4H), 7.22 (d, J = 8.4 Hz, 1H), 5.24 (quin, J = 8.8 Hz, 1H), 5.13 (d, J = 3.3 Hz, 1H), 4.91 - 5.04 (m, 1H), 4.44 - 4.56 (m, 2H), 4.29 (br s, 1H), 3.75 (br dd, J = 9.6, 6.1 Hz, 1H), 3.58 - 3.71 (m, 4H), 3.52 - 3.57 (m, 1H), 2.92 (br dd, J = 31.8, 9.4 Hz, 2H), 2.59 - 2.68 (m, 1H), 2.41 - 2.44 (m, 4H), 2.26 (br d, J = 3.1 Hz, 4H), 2.12 - 2.19 (m, 6H), 1.95 - 2.09 (m, 8H), 1.64 - 1.83 (m, 8H), 1.12 (d, J = 6.1 Hz, 3H), 0.94 - 0.98 (m, 9H)

^{13}C NMR (150 MHz, DMSO-*d$_6$*) δ 171.4, 170.3, 168.7, 163.5, 150.9, 148.2, 147.1, 142.9, 140.4, 138.2, 132.7, 131.5, 130.5, 129.9, 129.5, 128.0, 127.0, 124.2, 122.2, 120.5, 116.0, 114.7, 112.9, 108.6, 72.9, 70.3, 68.2, 63.1, 58.0, 56.0, 55.7, 54.0, 53.5, 53.3, 52.1, 46.8, 41.1, 37.1, 35.5, 32.8, 27.4, 25.8, 25.7, 24.0, 18.0, 15.4, 11.7, 9.7

HRMS (m/z) for $C_{56}H_{70}BrN_9O_6S$ $[M+H]^+$ calculated 1076.44259; obtained 1076.43954

Supplementary Figure 24: synthesis of PROTAC 25

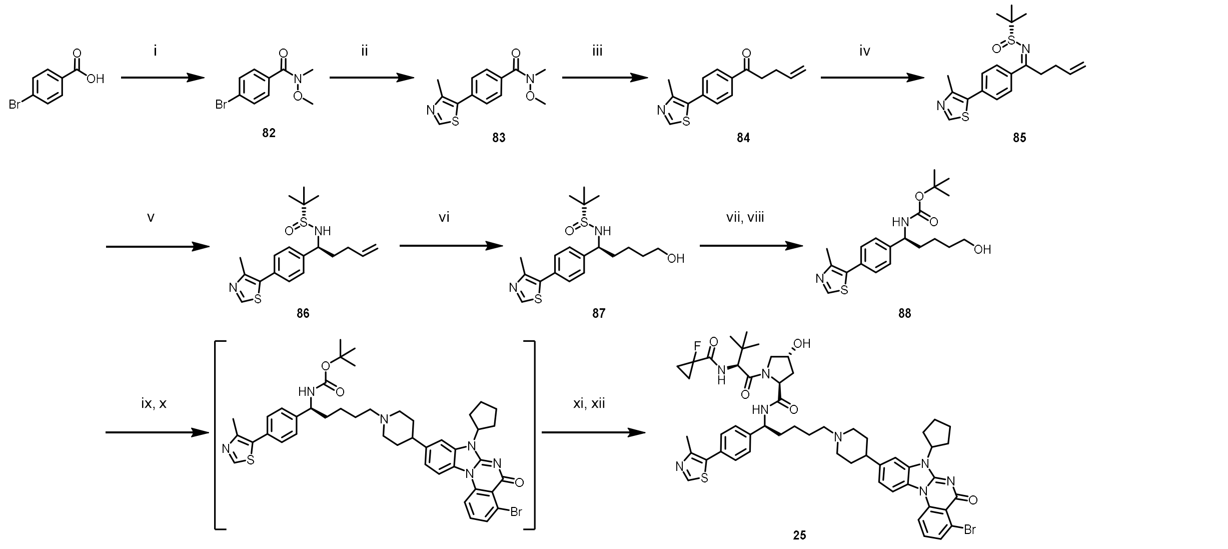

i) *N*,*O*-dimethylhydroxylamine hydrochloride, HATU, DIPEA, DMF, RT ; ii) 4-methylthiazole, $Pd(OAc)_2$, KOAc, DMA, 130 °C ; iii) butenylmagnesium bromide, THF, 0 °C – RT ; iv) (*R*)-2-methylpropane-2-sulfinamide, $Ti(O^iPr)_4$, THF, RT ; v) *L*-selectride, THF, -78 °C – RT ; vi) 9-BBN, THF, 0 °C – RT, then 30% aq. H_2O_2, 4 M NaOH, 0 °C – RT ; vii) HCl, DCM, MeOH, 0 °C – RT ; viii) Boc_2O, Et_3N, 1,4-dioxane/H_2O, 0 °C – RT ; ix) MsCl, Et_3N, DCM, 0 °C – RT ; x) **4**, DIPEA, DMF, 80 °C ; xi) 37% aq. HCl, MeOH, 0 °C – RT ; xii) 1-fluorocyclopropane-1-carboxylic acid, HATU, Et_3N, DMF, RT.

4-bromo-*N*-methoxy-*N*-methylbenzamide 82

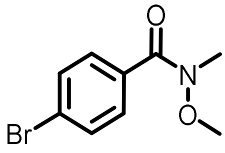

4-bromo-benzoic acid (10.0 g, 49.7 mmol) was dissolved in DMF (100 mL) and DIPEA (27.5 mL, 149.2 mmol), HATU (22.7 g, 59.7 mmol) and *N*,*O*-dimethyl hydroxylamine hydrochloride (4.8 g, 49.7 mmol) were added. The mixture was stirred at RT for 16 hours. It was quenched with water and extracted with EtOAc twice. The combined organics were washed with water and brine twice, dried over Na_2SO_4 and concentrated. The residue was purified by column chromatography on silica gel (0-50% EtOAc in petroleum ether) to yield **82** (10.0 g, 82% yield).

^{1}H NMR (400 MHz, $CDCl_3$-*d*) δ 7.63 – 7.51 (m, 4H), 3.54 (s, 3H), 3.36 (s, 3H)

MS (ESI) for $C_9H_{10}BrNO_2$ $[M+H]^+$ 244

***N*-methoxy-*N*-methyl-4-(4-methyl-1,3-thiazol-5-yl)benzamide 83**

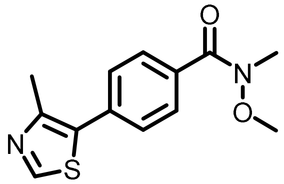

A suspension of **82** (10.0 g, 41.1 mol), 4-methylthiazole (8.1 g, 81.9 mmol), $Pd(OAc)_2$ (92 mg, 0.41 mmol) and KOAc (8.0 g, 81.9 mmol) in DMA (100 mL) was heated to 130 °C and stirred at this temperature for 16 hours. The mixture was cooled to RT and filtered through a Celite pad. It was quenched with ice-cold water and extracted with EtOAc twice. The combined organics were washed with water and brine, dried over Na_2SO_4 and concentrated. The residue was purified by column chromatography (0-50% EtOAc in petroleum ether) on silica gel to yield **83** (5.0 g, 47% yield).

^{1}H NMR (500 MHz, DMSO-d_6) δ 9.05 (s, 1H), 7.73 – 7.67 (m, 2H), 7.61 – 7.55 (m, 2H), 3.59 (s, 3H), 3.28 (s, 3H), 2.50 (s, 3H)

MS (ESI) for $C_{13}H_{14}N_2O_2S$ $[M+H]^+$ 263

1-(4-(4-methylthiazol-5-yl)phenyl)pent-4-en-1-one 84

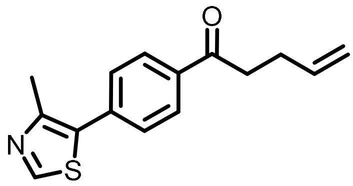

To a solution of **83** (10.0 g, 38.1 mmol) in THF (100 mL) at 0 °C was slowly added butenylmagnesium bromide (0.5 M in THF) (99.1 mL, 49.5 mmol) and the mixture was stirred at 0 °C for three hours. It was quenched with sat. aq. NH_4Cl (100 mL) and extracted twice with EtOAc. The combined organics were dried over Na_2SO_4 and concentrated. The residue was purified by column chromatography on silica gel (0-25% EtOAc in petroleum ether) to yield **84** (4.5 g, 46% yield).

^{1}H NMR (400 MHz, DMSO-d_6) δ 9.08 (s, 1H), 8.10 – 8.02 (m, 2H), 7.70 – 7.62 (m, 2H), 5.89 (ddt, J = 16.8, 10.2, 6.4 Hz, 1H), 5.09 (m, 1H), 4.99 (ddt, J = 10.2, 2.1, 1.3 Hz, 1H), 3.17 (t, J = 7.2 Hz, 2H), 2.51 (s, 3H), 2.45 – 2.33 (m, 2H)

MS (ESI) for $C_{15}H_{15}NOS$ $[M+H]^+$ 258

(*R*)-2-methyl-*N*-(1-(4-(4-methylthiazol-5-yl)phenyl)pent-4-en-1-ylidene)propane-2-sulfinamide 85

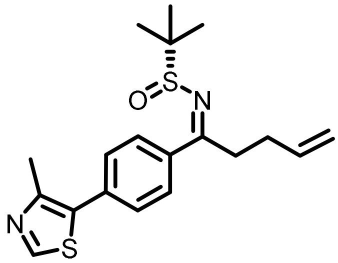

To a solution of **84** (10.0 g, 38.9 mmol) in THF (200 mL) was added (*R*)-2-methylpropane-2-sulfinamide (9.4 g, 77.8 mmol) and $Ti(O^iPr)_4$ (57.5 mL, 194.5 mmol) and the reaction mixture was refluxed for 16 hours. It was quenched with water and extracted with EtOAc twice. The combined organics were dried over Na_2SO_4 and concentrated. The residue was purified by column chromatography on silica gel (80-100% EtOAc in petroleum ether) to yield **85** (7.5 g, 54% yield).

^{1}H NMR (400 MHz, $CDCl_3$-*d*) δ 8.73 (s, 1H), 7.94 – 7.87 (m, 2H), 7.61 – 7.49 (m, 2H), 5.90 (ddt, J = 16.8, 10.1, 6.6 Hz, 1H), 5.16 – 5.06 (m, 1H), 5.06 – 5.02 (m, 1H), 3.46 – 3.34 (m, 1H), 3.31 – 3.27 (m, 1H), 2.59 (s, 3H), 2.56 – 2.37 (m, 2H), 1.34 (s, 9H)

MS (ESI) for $C_{19}H_{24}N_2OS_2$ $[M+H]^+$ 361

(*R*)-2-methyl-*N*-((*S*)-1-(4-(4-methylthiazol-5-yl)phenyl)pent-4-en-1-yl)propane-2-sulfinamide 86

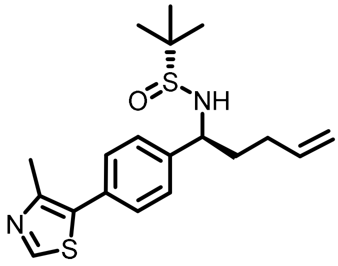

To a solution of **85** (9.0 g, 25.0 mmol) in dry THF (90 mL) was added *L*-Selectride (1.0 M in THF) (27.5 mL, 27.5 mmol) at -78 °C and the reaction mixture was stirred at the same temperature for 1 hour. It was quenched with sat. aq. NH_4Cl solution and extracted with EtOAc twice. The combined organics were dried over Na_2SO_4 and concentrated to yield crude **86** (5.0 g, 55% yield, dr > 9:1).

^{1}H NMR (400 MHz, $CDCl_3$-*d*) δ 8.68 (s, 1H), 7.48 – 7.40 (m, 2H), 7.40 – 7.32 (m, 2H), 5.79 (ddt, J = 16.8, 10.2, 6.4 Hz, 1H), 5.06 (m, 1H), 5.03 – 4.97 (m, 1H), 4.46 (ddd, J = 9.5, 6.2, 2.7 Hz, 1H), 3.49 (d, J = 2.7 Hz, 1H), 2.56 (s, 3H), 2.13 – 2.00 (m, 2H), 2.00 – 1.86 (m, 2H), 1.20 (s, 9H)

MS (ESI) for $C_{19}H_{26}N_2OS_2$ $[M+H]^+$ 363

(*R*)-*N*-((*S*)-5-hydroxy-1-(4-(4-methylthiazol-5-yl)phenyl)pentyl)-2-methylpropane-2-sulfinamide 87

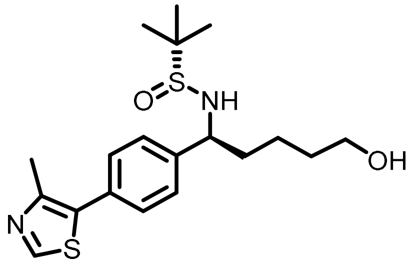

To a solution of **86** (7.0 g, 19.3 mmol) in THF (70 mL) was added 9-BBN (0.5 M in THF) (115.8 mL, 57.9 mmol) dropwise at 0 °C and the reaction mixture was stirred at RT for 3 hours. It was cooled to 0 °C again and 30% H_2O_2 in water (21.9 g, 193 mmol) and 4 M NaOH (48.3 mL, 193 mmol) were added. It was stirred at RT for 1 hour. The reaction was slowly quenched with 1 M HCl to pH 4 and extracted with DCM twice. The combined organics were dried over Na_2SO_4 and concentrated. The crude residue was purified by column chromatography on silica gel (5-8% MeOH in DCM) to yield **87** (4.0 g, 54% yield).

^{1}H NMR (400 MHz, $CDCl_3$-*d*) δ 8.69 (s, 1H), 7.46 – 7.39 (m, 2H), 7.39 – 7.31 (m, 2H), 4.44 (t, J = 7.0 Hz, 1H), 3.85 – 3.76 (m, 2H), 3.70 – 3.55 (m, 2H), 2.56 (s, 3H), 1.69 – 1.57 (m, 2H), 1.54 – 1.39 (m, 2H), 1.21 (s, 9H)

MS (ESI) for $C_{19}H_{28}N_2O_2S_2$ $[M+H]^+$ 381

***tert*-butyl (*S*)-(5-hydroxy-1-(4-(4-methylthiazol-5-yl)phenyl)pentyl)carbamate 88**

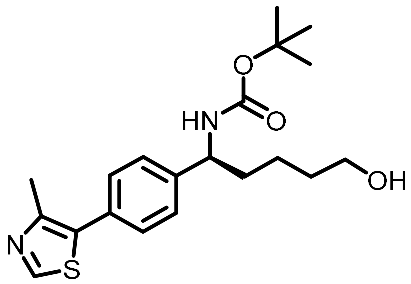

To a solution of **87** (5.0 g, 13.1 mmol) in DCM (50 mL) was added 4 M HCl in dioxane (10.0 mL, 40.0 mmol) dropwise at 0 °C and the reaction mixture was stirred at RT for 2 hours. It was concentrated under reduced pressure and the residue was triturated with diethyl ether to yield the crude amine as a hydrochloride salt. It was taken up in 1,4-dioxane (25 mL) and water (25 mL), Et_3N (5.5 mL, 39.3 mmol) and Boc_2O (4.3 g, 19.7 mmol) were added at 0 °C and the reaction mixture was stirred at RT for 2 hours. It was diluted with water and extracted with EtOAc twice. The combined organics were dried over Na_2SO_4 and concentrated. The residue was purified by column chromatography on silica gel (0-8% MeOH in DCM) to yield **88** (4.0 g, 79% yield)

^{1}H NMR (400 MHz, DMSO-d_6) δ 8.97 (s, 1H), 7.45 – 7.39 (m, 2H), 7.39 – 7.32 (m, 2H), 4.35 (t, J = 5.1 Hz, 1H), 3.37 – 3.31 (m, 2H), 2.44 (s, 3H), 1.67 – 1.53 (m, 2H), 1.39 (m, 2H), 1.35 (s, 9H), 1.26 – 1.12 (m, 2H)

MS (ESI) for $C_{20}H_{28}N_2O_3S$ $[M+H]^+$ 377

(2*S*,4*R*)-*N*-((*S*)-5-(4-(4-bromo-7-cyclopentyl-5-oxo-5,7-dihydrobenzo[4,5]imidazo[1,2-*a*]quinazolin-9-yl)piperidin-1-yl)-1-(4-(4-methylthiazol-5-yl)phenyl)pentyl)-1-((*S*)-2-(1-fluorocyclopropane-1-carboxamido)-3,3-dimethylbutanoyl)-4-hydroxypyrrolidine-2-carboxamide 25

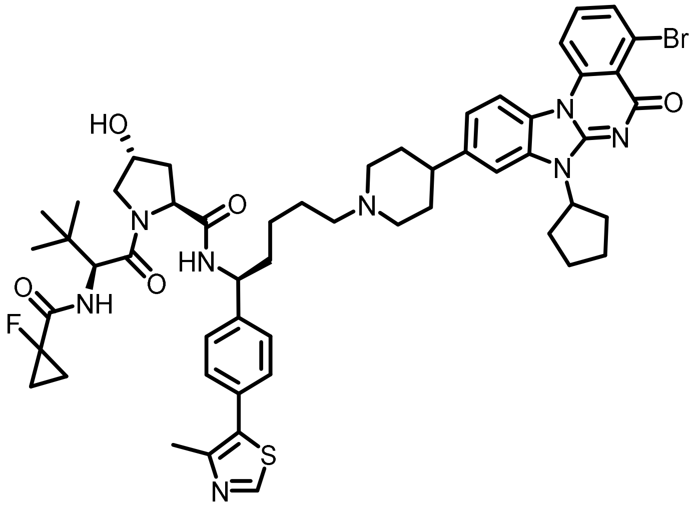

To a solution of **88** (56 mg, 0.12 mmol) in DCM (0.6 mL) was added Et_3N (52 µL, 0.38 mmol). The mixture was cooled to 0 °C and MsCl (20 µL, 0.25 mmol) was added dropwise. The mixture was stirred at RT for 10 minutes. The reaction was quenched with 10% aq. $NaHCO_3$ and stirred for 5 minutes. It was extracted twice with DCM and the combined organics were dried over Na_2SO_4 and concentrated. It was purified

over a short plug of silica, using 5% MeOH in DCM to elute the desired mesylate, which was dried *in vacuo* and used directly without further purification. The mesylate was taken up with **4** (69 mg, 0.13 mmol) in DMF (1 mL) and DIPEA (65 µL, 0.38 mmol) was added. The mixture was stirred at 80 °C overnight. The reaction was cooled and diluted with a 1:1 solution of MeCN/H_2O (1 mL). It was filtered through a syringe filter and injected directly for purification by acidic reverse phase HPLC. Fractions containing the desired intermediate were dried *in vacuo* and taken forward without further purification. To a solution of the intermediate in MeOH (1 mL) was added a 37% aq. solution of HCl (1 mL). The reaction was stirred for 1 hour at RT. It was concentrated under reduced pressure and the residue was triturated with diethyl ether to yield the crude amine as an HCl salt. In a separate flask, a solution of **52** (46 mg, 0.14 mmol) and Et_3N (70 µL, 0.47 mmol) in DMF (1 mL) was stirred at RT. To the solution was added HATU (107 mg, 0.28 mmol) and the mixture was stirred for 30 minutes. The crude amine was added and the mixture was stirred overnight at RT. The reaction was quenched with water and dried *in vacuo* and the residue was purified by basic reverse phase HPLC (Method 3b) to yield **25** (61 mg, 49% yield).

^{1}H NMR (600 MHz, DMSO-d_6) δ 8.98 (s, 1H), 8.46 (dd, J = 8.4, 2.6 Hz, 2H), 8.27 (d, J = 8.6 Hz, 1H), 7.79 (d, J = 7.9 Hz, 1H), 7.68 (t, J = 8.2 Hz, 1H), 7.54 (s, 1H), 7.42 - 7.47 (m, 2H), 7.37 - 7.42 (m, 2H), 7.22 - 7.31 (m, 2H), 5.25 (quin, J = 8.8 Hz, 1H), 5.14 (d, J = 3.5 Hz, 1H), 4.79 - 4.89 (m, 1H), 4.58 (d, J = 9.2 Hz, 1H), 4.51 (t, J = 8.3 Hz, 1H), 4.30 (br s, 1H), 3.54 - 3.69 (m, 2H), 2.89 - 3.07 (m, 2H), 2.66 - 2.78 (m, 1H), 2.46 (s, 3H), 2.21 - 2.34 (m, 4H), 1.93 - 2.09 (m, 8H), 1.66 - 1.86 (m, 10H), 1.42 - 1.59 (m, 3H), 1.28 - 1.42 (m, 1H), 1.22 (br dd, J = 8.3, 3.0 Hz, 2H), 0.98 (s, 9H)

^{13}C NMR (150 MHz, DMSO-d_6) δ 170.1, 168.1, 167.4 (d, CF = 20.4 Hz), 163.5, 150.9, 148.2, 147.1, 143.3, 143.0, 138.2, 132.7, 131.5, 130.5, 129.9, 129.1, 128.2, 126.2, 124.2, 122.2, 120.6, 116.0, 114.8, 112.9, 108.6, 77.6 (d, CF = 231.4 Hz), 68.2, 58.1, 57.6, 56.0, 55.9, 53.4, 53.2, 51.4, 41.3, 37.1, 35.7, 35.5, 32.7, 27.4, 25.7, 25.6, 23.9, 23.1, 15.4, 12.2 (m, CF)

HRMS (m/z) for $C_{54}H_{64}BrFN_8O_5S$ $[M+H]^+$ calculated 1035.39605; obtained 1035.39371

Supplementary Figure 25: synthesis of PROTAC 11

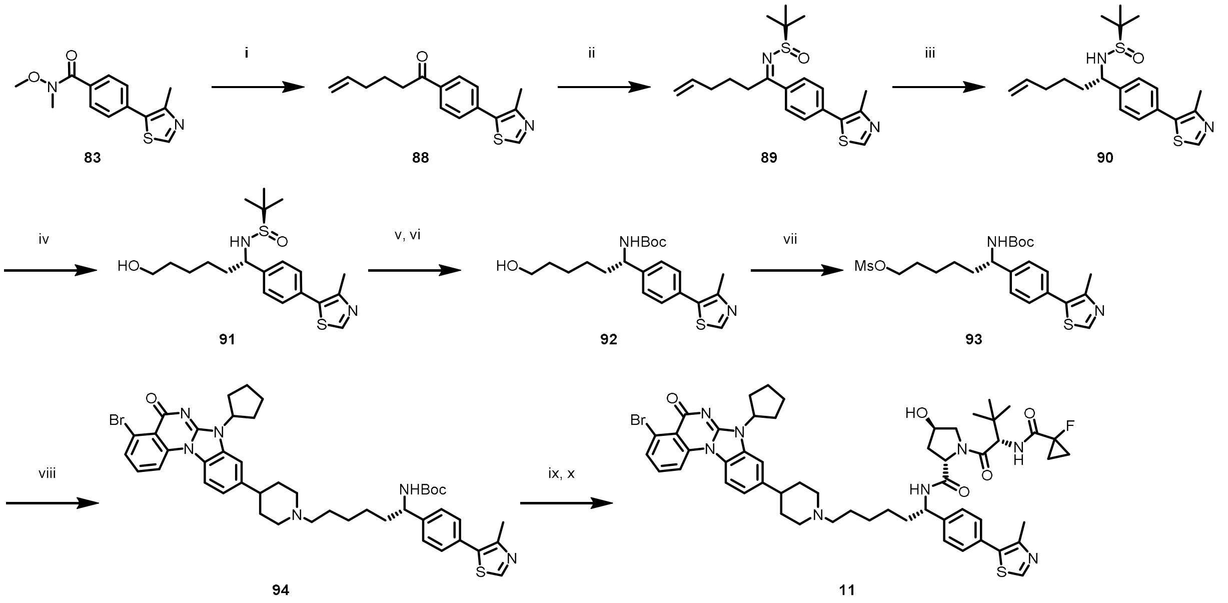

i) (pent-4-en-1-yl)magnesium bromide, THF, -10 °C ; ii) (*R*)-2-methylpropane-2-sulfinamide, $Ti(OEt)_4$, THF, 65 °C ; iii) *L*-selectride, THF, -78 °C ; iv) 9-BBN, THF, 0 °C – RT, then 30% aq.H_2O_2, 4 M NaOH, 0 °C – RT ; v) HCl, DCM, 0 °C ; vi) Boc_2O, Et_3N, 1,4-dioxane/H_2O, 0 °C - RT ; vii) MsCl, Et_3N, DCM, 0 °C ; viii) **4**, DIPEA, NMP, 80 °C ; ix) HCl, MeOH, RT ; x) **51**, HATU, Et_3N, DMF, RT

1-(4-(4-methylthiazol-5-yl)phenyl)hex-5-en-1-one 88

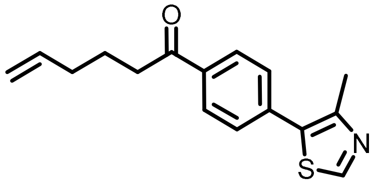

83 (3.00 g, 0.011 mol) was dissolved in THF (30.0 mL) and cooled to -10 °C. Then (pent-4-en-1-yl)magnesium bromide (22.9 mL, 0.023 mol) was added dropwise and the reaction mixture was stirred at RT for 2 hours. It was quenched with sat. aq. NH_4Cl solution and extracted with EtOAc twice. The combined organic layers were dried over Na_2SO_4 and concentrated *in vacuo*. The crude was purified by silica gel column chromatography (40-60% EtOAc in petroleum ether) to give the desired product **88** (1.60 g, 52% yield).

^{1}H NMR (500 MHz, $CDCl_3$-*d*) δ 8.74 (s, 1H), 7.99 - 8.04 (m, 2H), 7.52 - 7.58 (m, 2H), 5.84 (ddt, J = 17.0, 10.3, 6.7, 6.7 Hz, 1H), 4.97 - 5.10 (m, 2H), 3.00 (t, J = 7.4 Hz, 2H), 2.58 (s, 3H), 2.18 (q, J = 7.1 Hz, 2H), 1.88 (quin, J = 7.3 Hz, 2H)

MS (ESI) for $C_{16}H_{17}NOS$ $[M+H]^+$ 272

(*R*)-2-methyl-*N*-(1-(4-(4-methylthiazol-5-yl)phenyl)hex-5-en-1-ylidene)propane-2-sulfinamide 89

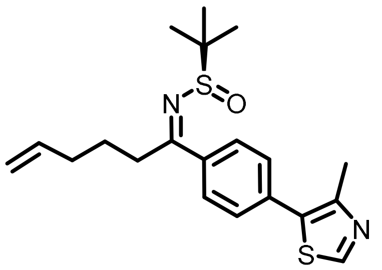

To a solution of **88** (3.00 g, 0.011 mol) in THF (30.0 mL) was added (*R*)-2-methylpropane-2-sulfinamide (2.01 g, 77.8 mmol) and $Ti(OEt)_4$ (7.57 g, 0.033 mmol) and the reaction mixture was refluxed for 16 hours. It was quenched with sat. aq. $NaHCO_3$ solution and extracted with EtOAc twice. The combined organics were washed with water, dried over Na_2SO_4 and concentrated *in vacuo*. The crude product **89** was taken to the next step without further purification (3.00 g, 73% yield).

^{1}H NMR (400 MHz, $CDCl_3$-*d*) δ 8.73 (s, 1H), 7.90 (br d, J = 7.6 Hz, 2H), 7.52 (d, J = 8.5 Hz, 2H), 5.84 (ddt, J = 17.0, 10.2, 6.7, 6.7 Hz, 1H), 4.96 - 5.13 (m, 2H), 3.12 - 3.35 (m, 2H), 2.59 (s, 3H), 2.22 (q, J = 7.3 Hz, 2H), 1.73 - 1.88 (m, 2H), 1.26 - 1.39 (m, 9H)

MS (ESI) for $C_{20}H_{26}N_2OS_2$ $[M+H]^+$ 375

(*R*)-2-methyl-*N*-((*S*)-1-(4-(4-methylthiazol-5-yl)phenyl)hex-5-en-1-yl)propane-2-sulfinamide 90

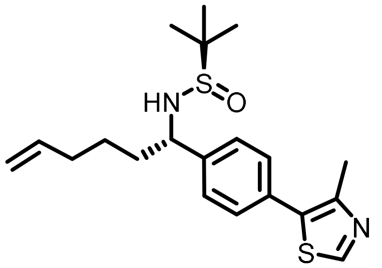

A stirred solution of **89** (2.0 g, 5.34 mmol) in THF (20.0 mL) was cooled to -78 °C and *L*-selectride (1.11 g, 5.87 mmol) was added. The mixture was stirred at the same temperature for 1 hour. The reaction was quenched with sat. aq. NH_4Cl and extracted with EtOAc twice. The combined organic layers were dried over Na_2SO_4 and concentrated *in vacuo*. The crude was purified by silica gel column chromatography (80-100% EtOAc in petroleum ether) to give the desired product **90** (1.0 g, 50% yield).

^{1}H NMR (400 MHz, $CDCl_3$-*d*) δ 8.68 (s, 1H), 7.33 - 7.45 (m, 4H), 5.64 - 5.86 (m, 1H), 4.88 - 5.07 (m, 2H), 4.36 - 4.46 (m, 1H), 3.42 (br s, 1H), 2.56 (s, 2H), 2.55 (s, 1H), 2.05 - 2.10 (m, 2H), 1.74 - 1.88 (m, 2H), 1.35 - 1.49 (m, 1H), 1.23 - 1.26 (m, 6H), 1.20 (s, 4H)

MS (ESI) for $C_{20}H_{28}N_2OS_2$ $[M+H]^+$ 377

(*R*)-*N*-((*S*)-6-hydroxy-1-(4-(4-methylthiazol-5-yl)phenyl)hexyl)-2-methylpropane-2-sulfinamide 91

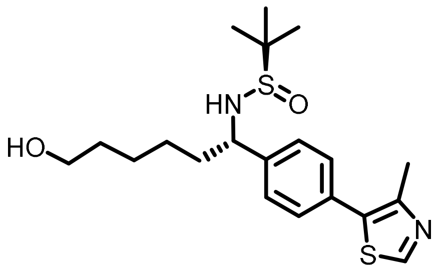

A stirred solution of **90** (5.00 g, 0.013 mol) in THF (50.0 mL) is cooled to 0 °C and 9-BBN (0.5 M in THF) (79.7 mL, 0.040 mol) was added. The reaction was stirred at RT for 2 hours. It was cooled to 0 °C again and 30% H_2O_2 (15.05 g, 0.133 mol) was added dropwise followed by addition of 4 M aq. NaOH (33.2 mL, 0.133 mol). The mixture was stirred at RT for 1 hour and slowly quenched with 1 M aq. HCl to pH 4 and extracted with DCM twice. The combined organic layers were dried over Na_2SO_4 and concentrated *in vacuo*. The crude product was purified by silica gel column chromatography (5-8% MeOH in DCM) to give the desired product **91** (3.50 g, 67% yield).

^{1}H NMR (400 MHz, DMSO-*d₆*) δ 8.97 (s, 1H), 7.41 - 7.45 (m, 3H), 7.37 - 7.40 (m, 2H), 5.36 (d, J = 5.4 Hz, 1H), 4.29 - 4.34 (m, 1H), 4.25 (d, J = 4.4 Hz, 1H), 4.16 - 4.23 (m, 1H), 3.49 - 3.64 (m, 1H), 2.44 (s, 3H), 1.74 - 1.95 (m, 1H), 1.56 - 1.73 (m, 2H), 1.19 - 1.50 (m, 4H), 1.10 (s, 2H), 1.06 (s, 7H)

MS (ESI) for $C_{20}H_{30}N_2O_2S_2$ $[M+H]^+$ 396

(*S*)-*tert*-butyl (6-hydroxy-1-(4-(4-methylthiazol-5-yl)phenyl)hexyl)carbamate 92

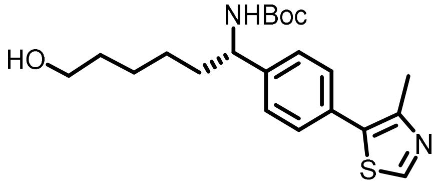

91 (3.50 g, 8.87 mmol) is dissolved in DCM (35.0 mL) and cooled to 0 °C. Then 4 M HCl in 1,4-dioxane (10.0 mL) was added dropwise and the reaction was stirred for 2 hours at the same temperature. The solvents were removed *in vacuo* and the residue was triturated with diethyl ether and taken to the next step without further purification. This crude (2.80 g, 8.57 mmol) was dissolved in 1,4-dioxane (15.0 mL) and water (15.0 mL). Then Et_3N (3.61 mL, 25.70 mmol) and Boc_2O (2.95 mL, 12.85 mmol) were added dropwise at 0 °C and the reaction was continued for 2 hours at RT. The mixture was diluted with water and extracted with EtOAc twice. The combined organic layers were dried over Na_2SO_4 and concentrated *in vacuo*. The crude was purified by silica gel column chromatography (50-80% EtOAc in petroleum ether) to give the desired product **92** (2.38 g, 71% yield)

^{1}H NMR (400 MHz, $CDCl_3$-*d*) δ 8.67 (s, 1H), 7.37 - 7.44 (m, 2H), 7.31 (br d, J = 7.8 Hz, 2H), 4.61 - 4.88 (m, 2H), 3.63 (t, J = 6.4 Hz, 2H), 2.53 (s, 3H), 1.76 (br d, J = 5.9 Hz, 2H), 1.51 - 1.59 (m, 3H), 1.34 - 1.46 (m, 13H)

MS (ESI) for $C_{21}H_{30}N_2O_3S$ $[M+H]^+$ 392

(*S*)-6-((*tert*-butoxycarbonyl)amino)-6-(4-(4-methylthiazol-5-yl)phenyl)hexyl methanesulfonate 93

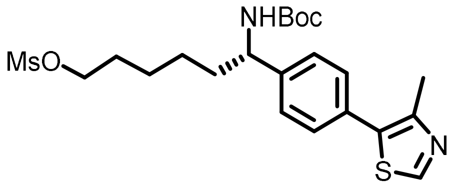

92 (75 mg, 0.19 mmol) and Et_3N (67 µL, 0.48 mmol) were dissolved in DCM (1.0 mL) and cooled to 0 °C. Then MsCl (30 µL, 0.38 mmol) was added slowly. The mixture was stirred at RT for 15 minutes. The reaction was quenched with sat. aq. $NaHCO_3$ solution, diluted with water and stirred at RT for 5 minutes. It was extracted with DCM twice. The combined organic layers were passed through a phase separator cartridge and concentrated *in vacuo*. The crude was purified by silica gel column chromatography (0-3% MeOH in DCM) to give the desired product **93** (59 mg, 90% yield).

^{1}H NMR (400 MHz, $CDCl_3$-*d*) δ 8.70 (s, 1H), 7.47 – 7.41 (m, 2H), 7.37 – 7.31 (m, 2H), 4.83 (s, 1H), 4.67 (s, 1H), 4.23 (t, J = 6.5 Hz, 2H), 3.01 (s, 3H), 2.57 (s, 3H), 1.82 – 1.73 (m, 4H), 1.46 (s, 9H), 1.37 – 1.34 (m, 4H).

MS (ESI) for $C_{22}H_{32}N_2O_5S_2$ $[M+H]^+$ 469

(*S*)-*tert*-butyl (6-(4-(4-bromo-7-cyclopentyl-5-oxo-5,7-dihydrobenzo[4,5]imidazo[1,2-*a*]quinazolin-9-yl)piperidin-1-yl)-1-(4-(4-methylthiazol-5-yl)phenyl)hexyl)carbamate 94

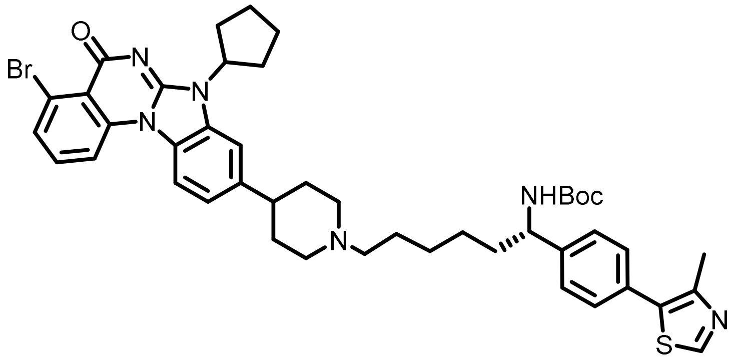

93 (59 mg, 0.13 mmol) and **4** (72 mg, 0.13 mmol) were dissolved in NMP (1.0 mL) and DIPEA (65 µL, 0.38 mmol) was added. The mixture was stirred at 80 °C overnight. The reaction mixture was diluted with MeCN/H_2O, filtered through a syringe filter and purified by basic HPLC (Method 3b) to give the desired product **94** (43 mg, 41% yield).

^{1}H NMR (400 MHz, DMSO-*d₆*) δ 8.98 (s, 1H), 8.47 (d, J = 8.4 Hz, 1H), 8.28 (d, J = 8.6 Hz, 1H), 7.79 (d, J = 7.9 Hz, 1H), 7.64 - 7.71 (m, 1H), 7.54 (s, 1H), 7.41 - 7.47 (m, 3H), 7.36 - 7.41 (m, 2H), 7.27 (d, J = 8.6 Hz, 1H), 5.25 (quin, J = 8.7 Hz, 1H), 4.41 - 4.59 (m, 1H), 2.98 (br d, J = 10.9 Hz, 2H), 2.46 (s, 3H), 2.22 - 2.36 (m, 5H), 1.95 - 2.08 (m, 7H), 1.57 - 1.84 (m, 8H), 1.22 - 1.46 (m, 14H)

MS (ESI) for $C_{45}H_{53}BrN_6O_3S$ $[M+H]^+$ 837

(2*S*,4*R*)-*N*-((*S*)-6-(4-(4-bromo-7-cyclopentyl-5-oxo-5,7-dihydrobenzo[4,5]imidazo[1,2-*a*]quinazolin-9-yl)piperidin-1-yl)-1-(4-(4-methylthiazol-5-yl)phenyl)hexyl)-1-((*S*)-2-(1-fluorocyclopropanecarboxamido)-3,3-dimethylbutanoyl)-4-hydroxypyrrolidine-2-carboxamide 11

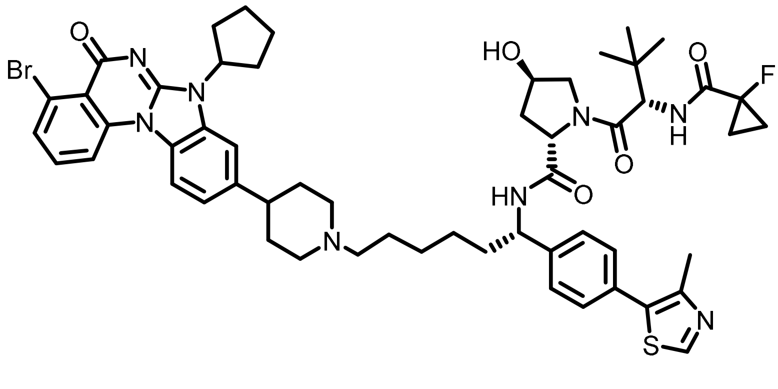

94 (31 mg, 0.037 mmol) was taken up in MeOH (0.75 mL) and conc. HCl (0.750 mL, 8.74 mmol) was added. The mixture was stirred at RT for 1 hour. The solvents were removed *in vacuo*. The residue was dissolved in water and basified with $NaHCO_3$ solution. It was extracted with DCM/MeOH 9:1 twice. The combined organic layers were passed through a phase separator cartridge and concentrated *in vacuo*. The crude amine was taken to the next step without further purification.

52 (18 mg, 0.048 mmol) was dissolved in DMF (1.0 mL) and Et_3N (21 µL, 0.15 mmol) and HATU (41 mg, 0.11 mmol) were added. The mixture was stirred at RT for 5 minutes and the crude amine (27 mg, 0.037 mmol) was added. The resulting mixture was stirred at RT for 1 hour. The reaction mixture was diluted with MeCN/H_2O, filtered through a syringe filter and purified by basic HPLC (Method 3b) to give the desired product **11** (26 mg, 68% yield).

^{1}H NMR (600 MHz, DMSO-d_6) δ 8.99 (s, 1H), 8.47 (d, J = 8.4 Hz, 2H), 8.35 (br d, J = 8.4 Hz, 1H), 7.81 (d, J = 7.7 Hz, 1H), 7.69 (t, J = 8.2 Hz, 1H), 7.48 (br s, 1H), 7.46 (d, J = 8.3 Hz, 2H), 7.39 (d, J = 8.3 Hz, 2H), 7.27 (br d, J = 8.1 Hz, 1H), 7.22 (br d, J = 7.5 Hz, 1H), 5.27 (quin, J = 8.7 Hz, 1H), 5.15 (d, J = 3.5 Hz, 1H), 4.80 - 4.88 (m, 1H), 4.59 (d, J = 9.2 Hz, 1H), 4.50 (t, J = 8.3 Hz, 1H), 4.30 (br s, 1H), 3.53 - 3.64 (m, 3H), 2.86 - 3.15 (m, 4H), 2.47 (s, 3H), 2.25 (br s, 2H), 1.98 - 2.15 (m, 7H), 1.86 - 1.98 (m, 2H), 1.58 - 1.83 (m, 8H), 1.48 (br d, J = 7.3 Hz, 1H), 1.29 - 1.44 (m, 6H), 1.18 - 1.29 (m, 2H), 0.99 (s, 9H)

^{13}C NMR (150 MHz, DMSO-d_6) δ 170.1, 168.1, 167.4 (d, CF = 20.4 Hz), 163.5, 148.3, 147.2, 143.2, 138.2, 132.7, 131.6, 130.5, 129.7, 129.2, 128.2, 126.2, 124.6, 122.3, 120.2, 116.0, 114.7, 113.2, 108.5, 77.6 (d, CF = 232.7 Hz), 68.2, 58.1, 56.1, 56.0, 53.3, 51.2, 37.2, 35.5, 35.4, 27.4, 25.8, 25.6, 24.6, 24.1, 15.4, 12.2 (m, CF)

HRMS (m/z) for $C_{55}H_{66}BrFN_8O_5S$ $[M+H]^+$ calculated 1049.41170; obtained 1049.41093

Supplementary Figure 26: synthesis of ACBI2 and PROTAC 28

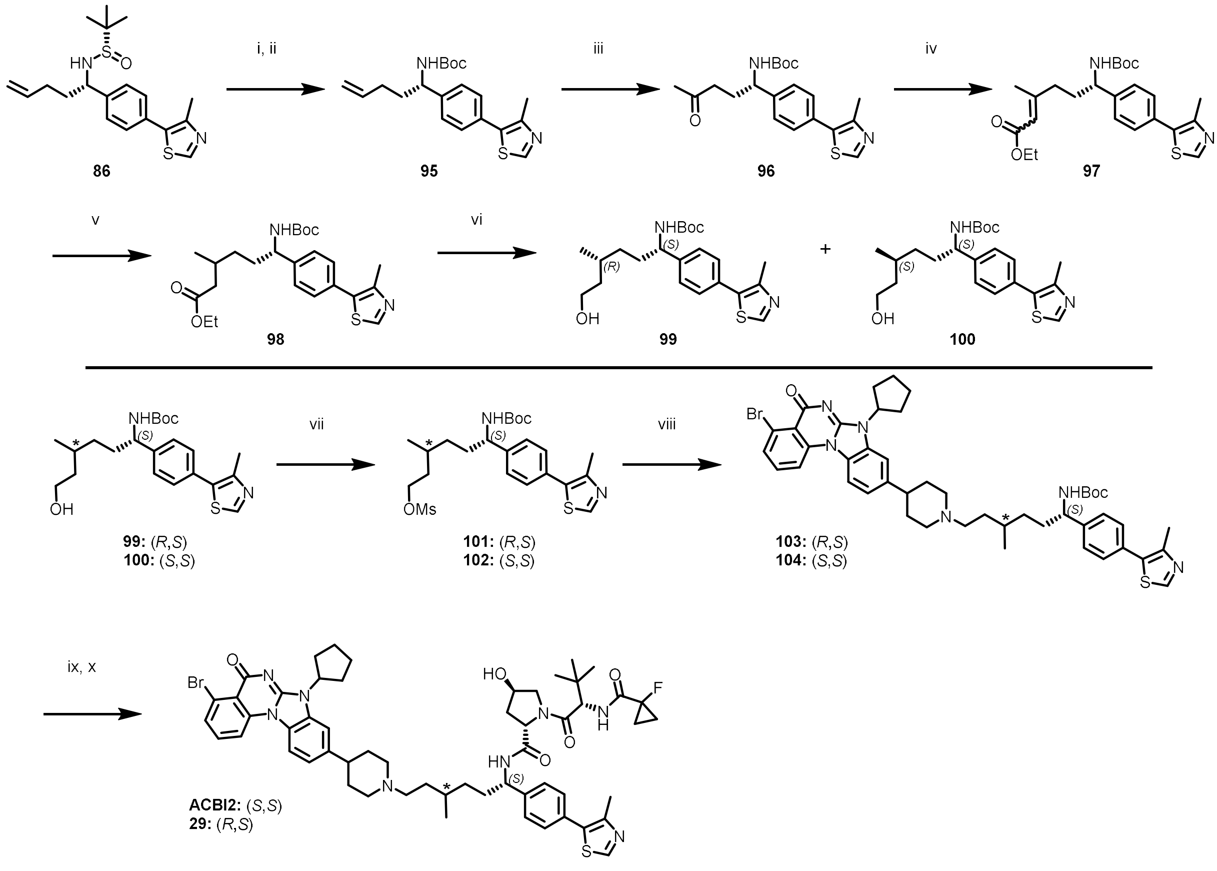

i) HCl, DCM, 0 °C ; ii) Boc_2O, Et_3N, 1,4-dioxane/H_2O, 0 °C – RT ; iii) $PdCl_2$, CrO3, MeCN/H_2O, 60 °C ; iv) NaH, triethylphosphonoacetate, THF, -10 °C ; v)H_2, Pd/C, MeOH, 50 °C ; vi) $LiAlH_4$, THF, 0 °C ; vii) MsCl, Et_3N, DCM, 0 °C ; viii) **4**, K_2CO_3, KI, NMP/MeCN, 80 °C ; ix) HCl, MeOH, RT ; x) **acid**, HATU, Et_3N, DMF, RT

(*S*)-*tert*-butyl (1-(4-(4-methylthiazol-5-yl)phenyl)pent-4-en-1-yl)carbamate 95

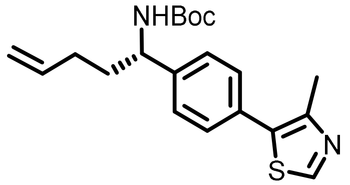

To a stirred solution of **86** (8.50 g, 0.023 mol) in DCM (40.0 mL), 4 M HCl in 1,4-dioxane (25.5 mL, 0.102 mol) was added dropwise at 0 °C and the reaction was stirred for 2 hours at 0 °C. The reaction mixture was concentrated *in vacuo* and crude (*S*)-1-(4-(4-methylthiazol-5-yl)phenyl)pent-4-en-1-amine hydrochloride was carried to the next step without further purification.

To a stirred solution of the crude (7.00 g, 0.024 mol) in 14-dioxane (35.0 mL) and water (35.0 mL), Et_3N (9.99 mL, 0.071 mol) and Boc_2O (8.17 ml, 0.036 mol) were added dropwise at 0 °C and the reaction was stirred for 2 hours at RT. The reaction mixture was diluted with water and extracted with EtOAc twice. The combined organic layers were dried over Na_2SO_4 and concentrated *in vacuo*. The obtained crude was

purified by silica gel column chromatography (50-80% EtOAc in petroleum ether) to give the desired product **95** (3.20 g, 38% yield).

^{1}H NMR (400 MHz, DMSO-*d$_6$*) δ 8.96 (s, 1H), 7.39 - 7.48 (m, 3H), 7.33 - 7.38 (m, 2H), 5.79 (ddt, J = 17.1, 10.3, 6.6, 6.6 Hz, 1H), 4.91 - 5.02 (m, 2H), 4.41 - 4.60 (m, 1H), 2.44 (s, 3H), 1.91 - 2.10 (m, 2H), 1.60 - 1.82 (m, 2H), 1.35 (s, 9H)

MS (ESI) for $C_{20}H_{26}N_2O_2S$ $[M+H]^+$ 359

(*S*)-*tert*-butyl (1-(4-(4-methylthiazol-5-yl)phenyl)-4-oxopentyl)carbamate 96

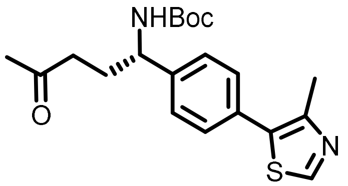

95 (8.50 g, 23.7 mmol) was dissolved in MeCN (200 mL) and water (30.0 mL). Then $PdCl_2$ (629 mg, 3.5 mmol) and CrO3 (4.70 g, 47.0 mmol) were added. The mixture was stirred at 60 °C for 6 hours. The reaction mixture was cooled to RT, diluted with EtOAc, filtered through a Celite pad and rinsed with EtOAc. The layers were separated. The aqueous layer was extracted with EtOAc. The organic layer was dried over Na_2SO_4 and concentrated *in vacuo*. The crude was purified by silica gel column chromatography (30-60% EtOAc in petroleum ether) to give the desired product **96** (4.00 g, 45% yield).

^{1}H NMR (400 MHz, DMSO-*d$_6$*) δ 8.97 (s, 1H), 7.40 - 7.46 (m, 3H), 7.32 - 7.38 (m, 2H), 4.40 - 4.49 (m, 1H), 2.41 - 2.46 (m, 5H), 2.04 (s, 3H), 1.74 - 1.83 (m, 2H), 1.35 (s, 9H)

MS (ESI) for $C_{20}H_{26}N_2O_3S$ $[M+H]^+$ 375

(*S*)-ethyl 6-((*tert*-butoxycarbonyl)amino)-3-methyl-6-(4-(4-methylthiazol-5-yl)phenyl)hex-2-enoate 97

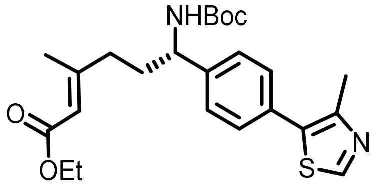

NaH (60% dispersion in mineral oil, 288 mg, 7.2 mmol) was dissolved in THF (10 mL) and cooled to 0 °C. Triethylphosphonoacetate (1.50 g, 6.7 mmol) was added dropwise. After complete addition, the mixture was stirred at 0 °C for 20 minutes and then cooled to -10 °C. **96** (1.00 g, 2.7 mmol), dissolved in a minimal amount of THF, was added dropwise. The mixture was allowed to reach RT and stirred for 16 hours. The reaction was quenched with ice-cold water and extracted with EtOAc. The combined organic layers were dried over Na_2SO_4 and concentrated under reduced pressure. The crude was purified by silica gel column chromatography (20-50% EtOAc in petroleum ether) to give the desired product **97** (0.55 g, 46% yield) as a mixture of E/Z-isomers.

^{1}H NMR (400 MHz, DMSO-*d$_6$*) δ 8.96 (s, 1H), 7.48 (br d, J = 8.8 Hz, 1H), 7.40 - 7.45 (m, 2H), 7.31 - 7.40 (m, 2H), 5.58 - 5.66 (m, 1H), 4.45 (br d, J = 5.4 Hz, 1H), 4.02 (quin, J = 7.1 Hz, 2H), 2.44 (s, 2H), 2.10 - 2.25 (m,

2H), 2.08 (s, 1H), 1.97 (s, 2H), 1.85 (s, 1H), 1.68 - 1.82 (m, 2H), 1.35 (s, 8H), 1.22 (br s, 1H), 1.12 - 1.20 (m, 3H)

MS (ESI) for $C_{24}H_{32}N_2O_4S$ $[M+H]^+$ 445

(6*S*)-ethyl 6-((*tert*-butoxycarbonyl)amino)-3-methyl-6-(4-(4-methylthiazol-5-yl)phenyl)hexanoate 98

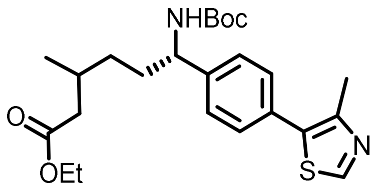

97 (2.0 g, 4.5 mmol) was dissolved in MeOH (60 mL) and 10% Pd/C (2.0 g) was added. The reaction mixture was stirred under aHydrogen pressure of 80 PSI at 50 °C for 40 hours. The reaction was filtered through a Celite pad, rinsed with 10% MeOH in DCM and concentrated under reduced pressure to give the desired product **98** (1.70 g, 85% yield), which was taken to the next step without further purification.

MS (ESI) for $C_{24}H_{34}N_2O_4S$ $[M+H]^+$ 447

***tert*-butyl ((1*S*,4*R*)-6-hydroxy-4-methyl-1-(4-(4-methylthiazol-5-yl)phenyl)hexyl)carbamate 99 and**

***tert*-butyl ((1*S*,4*S*)-6-hydroxy-4-methyl-1-(4-(4-methylthiazol-5-yl)phenyl)hexyl)carbamate 100**

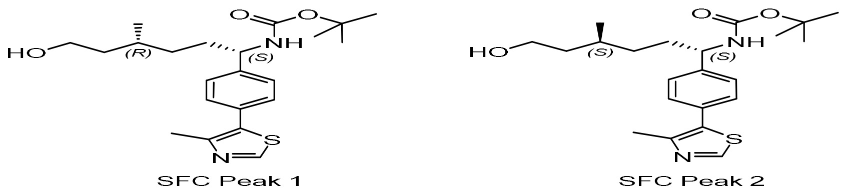

98 (1.70 g, 3.8 mmol) was dissolved in THF (17.0 mL) and cooled to 0 °C in an ice bath. Then $LiAlH_4$ (2 M in THF, 3.80 mL, 7.6 mmol) was added dropwise. The reaction mixture was stirred at 0 °C for 1 hour and cautiously quenched with sat. NH_4Cl solution at 0 °C. It was diluted with DCM and water. The salts were filtered off over a Celite pad. The layers were separated and the aqueous phase was extracted with DCM. The combined organic layers were dried over Na_2SO_4 and concentrated under reduced pressure. The crude was purified by silica gel column chromatography (50-70% EtOAc in petroleum ether) to give the product (1.10 g, 71% yield) as a mixture of diastereoisomers.

The diastereomeric mixture was further purified by SFC (Chiralpak AD-H, 25% MeOH) to obtain the desired products as pure diastereoisomers **99** (343 mg, 31% recovery) and **100** (359 mg, 33% recovery).

99: ^1H NMR (400 MHz, DMSO-d_6) δ 8.98 (s, 1H), 7.41 - 7.45 (m, 2H), 7.35 - 7.41 (m, 3H), 4.39 - 4.52 (m, 1H), 4.27 (br t, J = 4.9 Hz, 1H), 3.34 - 3.47 (m, 2H), 2.46 (s, 3H), 1.62 (q, J = 7.6 Hz, 2H), 1.40 - 1.56 (m, 2H), 1.37 (s, 9H), 1.21 (dt, J = 13.4, 6.6 Hz, 2H), 0.96 - 1.11 (m, 1H), 0.82 (d, J = 6.5 Hz, 3H)KK

MS (ESI) for $C_{22}H_{32}N_2O_3S$ $[M+H]^+$ 405

100: ^1H NMR (400 MHz, DMSO-d_6) δ 8.98 (s, 1H), 7.42 - 7.45 (m, 2H), 7.35 - 7.40 (m, 3H), 4.45 (br d, J = 5.6 Hz, 1H), 4.24 (t, J = 5.0 Hz, 1H), 3.34 - 3.46 (m, 2H), 1.68 (dt, J = 13.9, 8.9 Hz, 1H), 1.39 - 1.61 (m, 4H), 1.37 (s, 9H), 1.14 - 1.29 (m, 5H), 0.83 (d, J = 6.6 Hz, 3H)

MS (ESI) for $C_{22}H_{32}N_2O_3S$ $[M+H]^+$ 405

(3*S*,6*S*)-6-((*tert*-butoxycarbonyl)amino)-3-methyl-6-(4-(4-methylthiazol-5-yl)phenyl)hexyl methanesulfonate 102

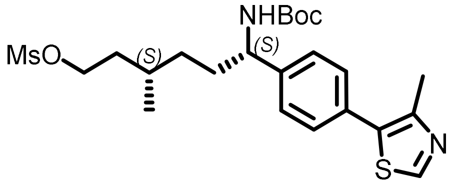

100 (2.59 g, 6.10 mmol) and Et_3N (2.13 mL, 15.26 mmol) were dissolved in DCM (25.0 mL) and cooled to 0 °C. Then MsCl (0.95 mL, 12.21 mmol) was added slowly. The mixture was stirred at 0 °C for 20 minutes, then quenched with sat. aq. $NaHCO_3$ solution, diluted with water and stirred at RT for 30 minutes. It was extracted with DCM twice. The combined organics were passed through a phase separator cartridge and concentrated under reduced pressure. The crude was purified by silica gel column chromatography (0-4% MeOH in DCM to give the desired product **102** (2.93 g, 99% yield).

^{1}H NMR (500 MHz, DMSO-d_6) δ 8.98 (s, 1H), 7.41 - 7.46 (m, 3H), 7.37 - 7.40 (m, 2H), 4.43 - 4.51 (m, 1H), 4.16 - 4.25 (m, 2H), 3.15 (s, 3H), 2.46 (s, 3H), 1.64 - 1.74 (m, 2H), 1.52 - 1.62 (m, 2H), 1.43 - 1.51 (m, 1H), 1.37 (s, 9H), 1.22 - 1.30 (m, 2H), 0.88 (d, J = 6.6 Hz, 3H)

MS (ESI) for $C_{23}H_{34}N_2O_5S_2$ $[M+H]^+$ 483

***tert*-butyl ((1*S*,4*S*)-6-(4-(4-bromo-7-cyclopentyl-5-oxo-5,7-dihydrobenzo[4,5]imidazo[1,2-*a*]quinazolin-9-yl)piperidin-1-yl)-4-methyl-1-(4-(4-methylthiazol-5-yl)phenyl)hexyl)carbamate 104**

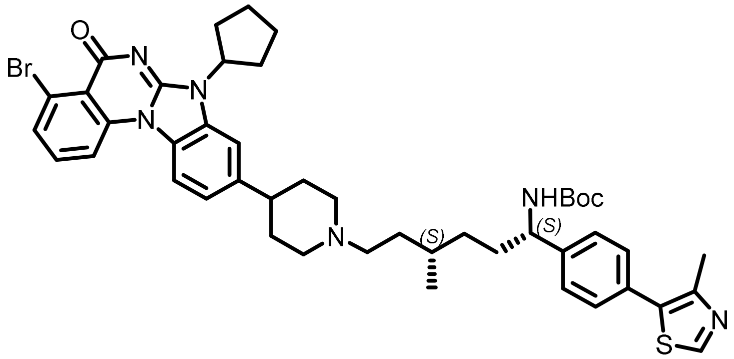

102 (475 mg, 0.98 mmol) and **4** (595 mg, 1.28 mmol) were taken up in NMP (5.0 mL) and MeCN (5.0 mL). Then K_2CO_3 (272 mg, 1.97 mmol) and KI (327 mg, 1.97 mmol) were added. The mixture was stirred at 80 °C for 2 hours. The mixture was diluted with DCM and water. The layers were separated. The aqueous layer was extracted with DCM. The combined organics were washed with sat. aq. NH_4Cl solution, passed through a phase separator cartridge and concentrated. The residue was dissolved in MeCN/H2O, filtered through a syringe filter and purified by basic prep HPLC (Method 3b) to give the desired product **104** (616 mg, 74% yield).

^{1}H NMR (500 MHz, DMSO-d_6) δ 8.98 (s, 1H), 8.47 (d, J = 8.5 Hz, 1H), 8.29 (d, J = 8.5 Hz, 1H), 7.80 (d, J = 7.9 Hz, 1H), 7.67 (t, J = 8.2 Hz, 1H), 7.54 (s, 1H), 7.41 - 7.46 (m, 3H), 7.37 - 7.41 (m, 2H), 7.25 - 7.29 (m, 1H),

5.25 (quin, J = 8.7 Hz, 1H), 4.39 - 4.60 (m, 1H), 2.97 (br d, J = 11.0 Hz, 2H), 2.65 - 2.79 (m, 1H), 2.46 (s, 3H), 2.24 - 2.32 (m, 4H), 1.94 - 2.07 (m, 5H), 1.67 - 1.83 (m, 7H), 1.55 - 1.64 (m, 1H), 1.42 - 1.52 (m, 2H), 1.37 (s, 9H), 1.26 (br dd, J = 13.4, 5.8 Hz, 4H), 0.87 (d, J = 6.3 Hz, 3H)

MS (ESI) for $C_{46}H_{55}BrN_6O_3S$ $[M+H]^+$ 851

(2*S*,4*R*)-*N*-((1*S*,4*S*)-6-(4-(4-bromo-7-cyclopentyl-5-oxo-5,7-dihydrobenzo[4,5]imidazo[1,2-*a*]quinazolin-9-yl)piperidin-1-yl)-4-methyl-1-(4-(4-methylthiazol-5-yl)phenyl)hexyl)-1-((*S*)-2-(1-fluorocyclopropanecarboxamido)-3,3-dimethylbutanoyl)-4-hydroxypyrrolidine-2-carboxamide, ACBI2

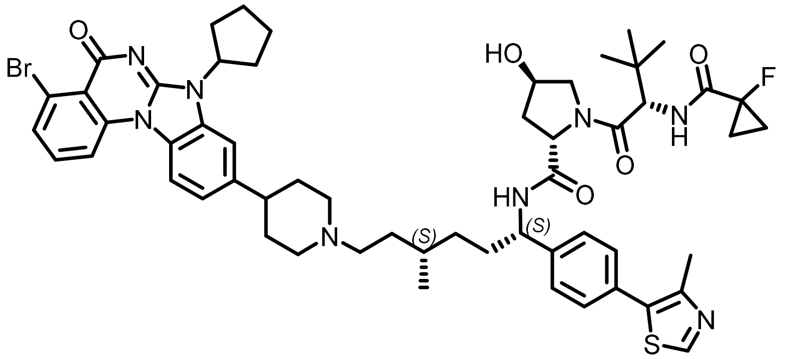

104 (615 mg, 0.72 mmol) was dissolved in MeOH (5.0 mL) and 4 M HCl in 1,4-dioxane (5.0 mL, 20.00 mmol) was added. The mixture was stirred at RT for 30 minutes. The solvents were removed *in vacuo* and the crude product was taken to the next step without further purification assuming quantitative yield.

52 (301 mg, 0.87 mmol), the crude amine (542 mg, 0.72 mmol) and HATU (420 mg, 1.08 mmol) were taken up in DMF (5.0 mL) and DIPEA (0.99 ml, 5.77 mmol) was added. The mixture was stirred at RT for 20 minutes. The reaction mixture was diluted with water and extracted with DCM twice. The combined organics were washed with water and sat. aq. NH_4Cl solution, passed through a phase separator cartridge and concentrated under reduced pressure. The residue was dissolved in MeCN/H_2O, filtered through a syringe filter and purified by basic prep HPLC (Method 3b) to give **ACBI2** (548 mg, 71% yield over two steps).

^{1}H NMR (600 MHz, DMSO-*d*$_6$) δ 8.98 (s, 1H), 8.47 (d, J = 8.4 Hz, 1H), 8.44 (d, J = 8.3 Hz, 1H), 8.28 (d, J = 8.6 Hz, 1H), 7.79 (d, J = 7.7 Hz, 1H), 7.67 (t, J = 8.1 Hz, 1H), 7.53 (s, 1H), 7.43 - 7.47 (m, 2H), 7.37 - 7.42 (m, 2H), 7.27 (br d, J = 8.3 Hz, 1H), 7.24 (dd, J = 9.2, 2.6 Hz, 1H), 5.25 (quin, J = 8.8 Hz, 1H), 5.13 (d, J = 3.7 Hz, 1H), 4.75 - 4.83 (m, 1H), 4.58 (d, J = 9.2 Hz, 1H), 4.51 (t, J = 8.3 Hz, 1H), 4.29 (br s, 1H), 3.53 - 3.65 (m, 2H), 3.00 (br d, J = 4.2 Hz, 2H), 2.69 (s, 1H), 2.46 (s, 3H), 2.22 - 2.35 (m, 4H), 1.95 - 2.12 (m, 8H), 1.69 - 1.91 (m, 7H), 1.61 - 1.69 (m, 1H), 1.44 - 1.56 (m, 2H), 1.19 - 1.42 (m, 7H), 0.98 (s, 9H), 0.88 (br d, J = 6.2 Hz, 3H)

^{13}C NMR (150 MHz, DMSO-*d*$_6$) δ 170.1, 168.1, 167.4 (d, CF = 20.4 Hz), 163.5, 150.9, 148.2, 147.1, 143.3, 138.2, 132.7, 131.5, 130.5, 129.9, 129.1, 128.2, 126.2, 124.2, 122.2, 120.6, 116.0, 114.8, 113.0, 108.5, 77.6 (d, CF = 231.4 Hz), 68.2, 66.3, 58.1, 56.0, 55.9, 55.5, 53.4, 53.4, 53.0, 51.8, 37.1, 35.5, 33.3, 32.8, 32.3, 29.8, 27.4, 25.7, 25.6, 23.9, 18.9, 15.4, 12.2 (m, CF)

MS (ESI) for $C_{56}H_{68}BrFN_8O_5S$ $[M+H]^+$ calculated 1063.4, obtained 1063.8 ; HRMS (m/z) for $C_{56}H_{68}BrFN_8O_5S$ $[M+H]^+$ calculated 1063.42735; obtained 1063.42481

(3*R*,6*S*)-6-((*tert*-butoxycarbonyl)amino)-3-methyl-6-(4-(4-methylthiazol-5-yl)phenyl)hexyl methanesulfonate 101

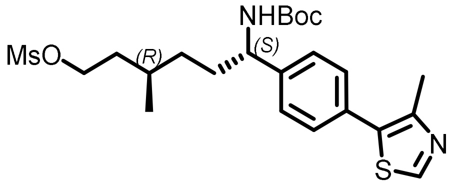

99 (360 mg, 0.89 mmol) and Et_3N (0.31 mL, 2.23 mmol) were dissolved in DCM (5.0 mL) and cooled to 0 °C. Then MsCl (0.14 mL, 1.78 mmol) was added slowly. The mixture was stirred at RT for 20 minutes, then quenched with sat. $NaHCO_3$ solution, diluted with water and stirred at RT for 15 minutes. It was extracted with DCM twice. The combined organics were passed through a phase separator cartridge and concentrated under reduced pressure. The crude was purified by silica gel column chromatography (0-5% MeOH in DCM) to give the desired product **101** (398 mg, 93% yield).

^{1}H NMR (500 MHz, DMSO-d_6) δ 8.98 (s, 1H), 7.42 - 7.46 (m, 3H), 7.37 - 7.40 (m, 2H), 4.40 - 4.51 (m, 1H), 4.16 - 4.25 (m, 2H), 3.16 (s, 3H), 2.46 (s, 3H), 1.52 - 1.73 (m, 4H), 1.42 - 1.50 (m, 1H), 1.37 (s, 9H), 1.24 (br s, 1H), 1.06 - 1.14 (m, 1H), 0.87 (d, *J* = 6.3 Hz, 3H)

MS (ESI) for $C_{23}H_{34}N_2O_5S_2$ $[M+H]^+$ 483

***tert*-butyl ((1*S*,4*R*)-6-(4-(4-bromo-7-cyclopentyl-5-oxo-5,7-dihydrobenzo[4,5]imidazo[1,2-*a*]quinazolin-9-yl)piperidin-1-yl)-4-methyl-1-(4-(4-methylthiazol-5-yl)phenyl)hexyl)carbamate 103**

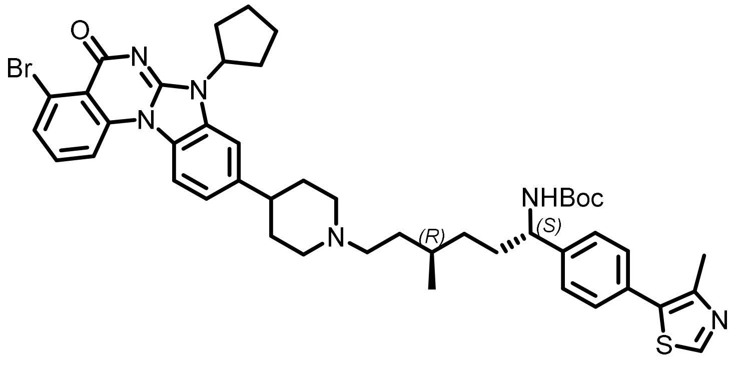

101 (398 mg, 0.83 mmol) and **4** (499 mg, 1.07 mmol) were taken up in NMP (4.0 mL) and MeCN (4.0 mL). Then K_2CO_3 (228 mg, 1.65 mmol) and KI (274 mg, 1.65 mmol) were added. The mixture was stirred at 80 °C for 2 hours and was then diluted with DCM and water. The layers were separated. The aqueous layer was extracted with DCM. The combined organics were washed with sat. aq. NH_4Cl solution, passed through a phase separator cartridge and concentrated *in vacuo*. The crude was dissolved in MeCN/H2O, filtered through a syringe filter and purified by basic prep HPLC (Method 3b) to give the desired product **103** (504 mg, 72% yield).

^{1}H NMR (500 MHz, DMSO-d_6) δ 8.97 (s, 1H), 8.47 (d, J = 8.5 Hz, 1H), 8.28 (d, J = 8.5 Hz, 1H), 7.80 (d, J = 7.6 Hz, 1H), 7.67 (t, J = 8.2 Hz, 1H), 7.54 (s, 1H), 7.41 - 7.46 (m, 3H), 7.38 - 7.41 (m, 2H), 7.27 (d, J = 8.5 Hz, 1H), 5.25 (quin, J = 8.7 Hz, 1H), 4.47 (q, J = 7.5 Hz, 1H), 2.97 (br d, J = 11.0 Hz, 2H), 2.65 - 2.75 (m, 1H), 2.45 (s,

3H), 2.27 (br t, J = 6.8 Hz, 4H), 1.92 - 2.05 (m, 6H), 1.70 - 1.86 (m, 6H), 1.65 (q, J = 7.7 Hz, 2H), 1.43 - 1.51 (m, 2H), 1.37 (s, 9H), 1.20 - 1.28 (m, 2H), 1.08 (dq, J = 14.2, 7.1 Hz, 1H), 0.86 (d, J = 6.3 Hz, 3H)

MS (ESI) for $C_{46}H_{55}BrN_6O_3S$ $[M+H]^+$ 851

(2*S*,4*R*)-*N*-((1*S*,4*R*)-6-(4-(4-bromo-7-cyclopentyl-5-oxo-5,7-dihydrobenzo[4,5]imidazo[1,2-*a*]quinazolin-9-yl)piperidin-1-yl)-4-methyl-1-(4-(4-methylthiazol-5-yl)phenyl)hexyl)-1-((*S*)-2-(1-fluorocyclopropanecarboxamido)-3,3-dimethylbutanoyl)-4-hydroxypyrrolidine-2-carboxamide 29

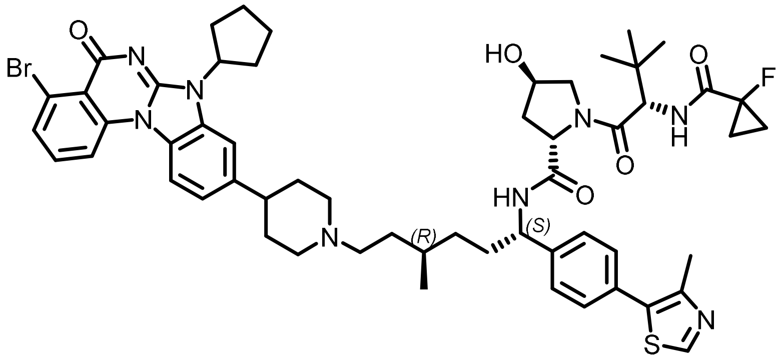

103 (503 mg, 0.59 mmol) was dissolved in MeOH (5.0 mL) and 4 M HCl in 1,4-dioxane (5.0 mL, 20.00 mmol) was added. The mixture was stirred at RT for 30 minutes. The solvents were removed *in vacuo* and the crude product was taken to the next step without further purification assuming quantitative yield.

52 (246 mg, 0.71 mmol), the crude amine (443 mg, 0.59 mmol) and HATU (343 mg, 0.88 mmol) were taken up in DMF (4.0 mL) and DIPEA (0.81 mL, 4.71 mmol) was added. The mixture was stirred at RT for 20 minutes. The reaction mixture was diluted with water and extracted with DCM twice. The combined organics were washed with water and sat. aq. NH_4Cl solution, passed through a phase separator cartridge and concentrated *in vacuo*. The residue was diluted with MeCN/H_2O, filtered through a syringe filter and purified by basic prep.HPLC (Method 3b) to give the desired product **29** (462 mg, 74% yield over two steps).

^1H NMR (600 MHz, DMSO-*d₆*) δ 8.97 (s, 1H), 8.46 (br d, J = 8.4 Hz, 2H), 8.28 (d, J = 8.6 Hz, 1H), 7.79 (d, J = 7.9 Hz, 1H), 7.67 (t, J = 8.2 Hz, 1H), 7.53 (s, 1H), 7.42 - 7.47 (m, 2H), 7.36 - 7.41 (m, 2H), 7.26 (br d, J = 8.4 Hz, 1H), 7.23 (br dd, J = 9.2, 2.2 Hz, 1H), 5.25 (quin, J = 8.7 Hz, 1H), 5.13 (d, J = 3.5 Hz, 1H), 4.75 - 4.88 (m, 1H), 4.58 (br d, J = 9.2 Hz, 1H), 4.50 (t, J = 8.2 Hz, 1H), 4.29 (br s, 1H), 3.51 - 3.66 (m, 2H), 2.98 (br d, J = 10.6 Hz, 2H), 2.64 - 2.76 (m, 1H), 2.45 (s, 3H), 2.23 - 2.34 (m, 4H), 1.92 - 2.08 (m, 7H), 1.64 - 1.85 (m, 9H), 1.51 (br s, 3H), 1.30 - 1.44 (m, 2H), 1.19 - 1.29 (m, 3H), 1.08 - 1.17 (m, 1H), 0.94 - 1.00 (m, 9H), 0.87 (br d, J = 5.7 Hz, 3H)

^{13}C NMR (150 MHz, DMSO-*d₆*) δ 170.1, 168.1, 167.4 (d, CF = 20.3 Hz), 163.5, 150.9, 148.2, 147.1, 143.2, 143.0, 138.2, 132.7, 131.5, 130.5, 129.9, 129.2, 128.2, 126.3, 124.2, 122.2, 120.6, 116.0, 114.8, 113.0, 108.5, 77.6 (d, CF = 231.4 Hz), 68.2, 58.1, 56.0, 55.9, 55.5, 53.5, 53.4, 53.1, 51.9, 41.4, 37.1, 35.6, 33.3, 32.9, 32.8, 32.8, 32.3, 29.8, 27.4, 25.7, 25.6, 23.9, 19.2, 15.4, 12.2 (m, CF)

HRMS (m/z) for $C_{56}H_{68}BrFN_8O_5S$ $[M+H]^+$ calculated 1063.42735; obtained 1063.42528

Supplementary Figure 27: synthesis of PROTAC 12 (*cis*-ACBI2)

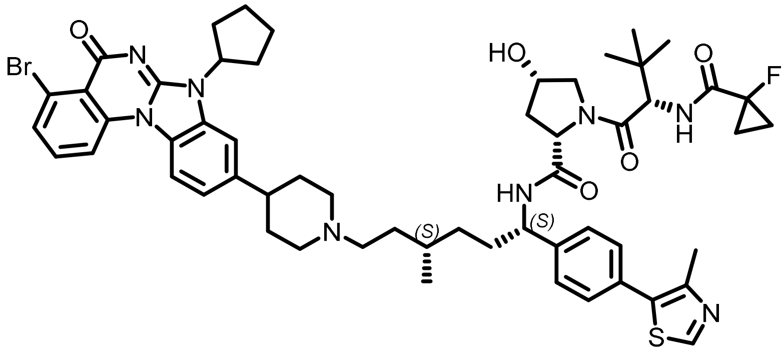

12 was prepared from ***cis*-51** according to the same procedures as **ACBI2.**

^{1}H NMR (600 MHz, DMSO-d_6) δ 8.98 (s, 1 H), 8.49 (d, J = 8.4 Hz, 1 H), 8.47 (d, J = 8.6 Hz, 1 H), 8.28 (d, J = 8.6 Hz, 1 H), 7.79 (d, J = 7.7 Hz, 1 H), 7.67 (t, J = 8.2 Hz, 1 H), 7.53 (s, 1 H), 7.43 - 7.48 (m, 2 H), 7.39 - 7.43 (m, 2 H), 7.23 - 7.28 (m, 2 H), 5.42 (d, J = 7.2 Hz, 1 H), 5.25 (quin, J = 8.8 Hz, 1 H), 4.75 - 4.85 (m, 1 H), 4.54 (d, J = 8.8 Hz, 1 H), 4.42 (dd, J = 8.7, 6.1 Hz, 1 H), 4.21 (dq, J = 11.9, 5.7 Hz, 1 H), 3.83 (dd, J = 10.3, 5.5 Hz, 1 H), 3.43 (dd, J = 10.2, 5.2 Hz, 1 H), 2.91 - 3.03 (m, 2 H), 2.68 (tt, J = 11.7, 3.7 Hz, 1 H), 2.46 (s, 3 H), 2.25 - 2.35 (m, 5 H), 1.96 - 2.07 (m, 6 H), 1.69 - 1.85 (m, 8 H), 1.60 (dt, J = 12.4, 6.0 Hz, 1 H), 1.45 - 1.53 (m, 2 H), 1.16 - 1.37 (m, 7 H), 1.00 (s, 9 H), 0.88 (d, J = 6.4 Hz, 3 H)

^{13}C NMR (150 MHz, DMSO-d_6) δ 170.7, 168.4, 167.7 (d, CF = 20.3 Hz), 163.5, 150.9, 148.2, 147.2, 143.0, 142.9, 138.2, 132.7, 131.5, 130.5, 129.9, 129.2, 128.2, 126.3, 124.2, 122.2, 120.6, 116.0, 114.8, 113.0, 108.5, 77.5 (d, CF = 232.7 Hz), 68.4, 58.0, 56.1, 55.5, 55.1, 53.5, 53.4, 53.1, 52.0, 41.4, 36.1, 34.9, 33.2, 33.0, 32.8, 32.8, 32.3, 29.8, 27.4, 25.6, 18.9, 15.4, 12.3 (m, CF)

HRMS (m/z) for $C_{56}H_{68}BrFN_8O_5S$ $[M+H]^+$ calculated 1063.42735; obtained 1063.42388

Supplementary Figure 28: synthesis of PROTAC 27

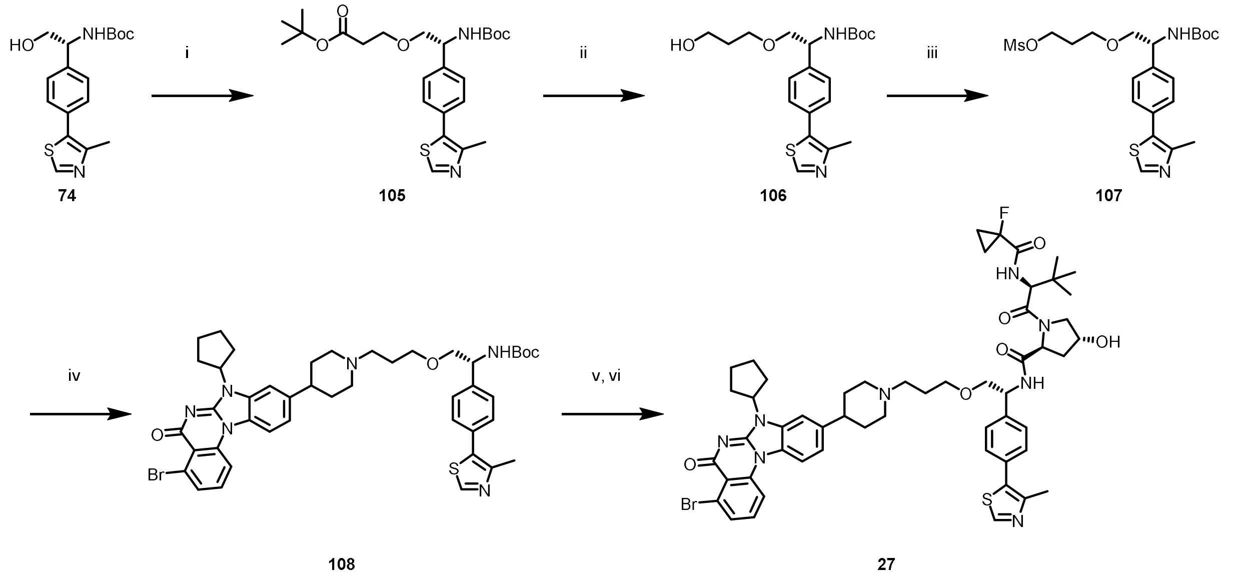

i) *tert*-butyl 3-bromopropanoate, tetrabutylammonium hydrogen sulfate, DCM, NaOH, RT ; ii) $LiAlH_4$, THF, 0 °C ; iii) MsCl, Et_3N, DCM, 0 °C ; iv) **4**, DIPEA, DMF, 80 °C ; v) HCl, MeOH, RT ; vi) **52**, HATU, Et_3N, DMF, RT.

(*R*)-*tert*-butyl 3-(2-((*tert*-butoxycarbonyl)amino)-2-(4-(4-methylthiazol-5-yl)phenyl)ethoxy)propanoate 105

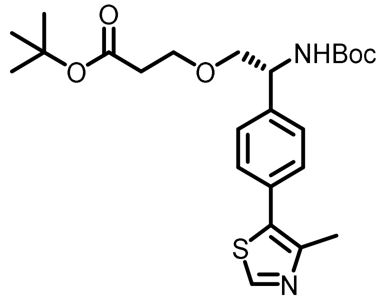

74 (5.00 g, 13.70 mmol) and tetrabutylammonium hydrogen sulfate (1.860 g, 5.48 mmol) were suspended in DCM (100.0 mL) and 4 M NaOH (80.0 mL) was added. Then *tert*-butyl 3-bromopropanoate (5.89 mL, 34.24 mmol), dissolved in 30 mL DCM, was added dropwise. The reaction mixture was stirred at RT vigorously for 2 days. Then additional bromide (2.90 mL, 16.86 mmol) was added and stirring was continued for 24 hours. The reaction mixture was diluted with water and extracted with DCM three times. The combined organic layers were passed through a phase separator cartridge and concentrated under reduced pressure. The crude was purified by silica gel column chromatography (0-5% MeOH in DCM) to give the desired product **105** (4.41 g, 70% yield).

^{1}H NMR (500 MHz, $CDCl_3$-*d*) δ 8.68 (s, 1H), 7.44 – 7.36 (m, 4H), 5.50 (s, 1H), 4.84 (s, 1H), 3.78 – 3.65 (m, 4H), 2.55 (s, 3H), 2.50 (t, J = 6.1 Hz, 2H), 1.47 (s, 9H)

MS (ESI) for $C_{24}H_{34}N_2O_5S$ [M +H$^+$] 463

(*R*)-*tert*-butyl (2-(3-hydroxypropoxy)-1-(4-(4-methylthiazol-5-yl)phenyl)ethyl)carbamate 106

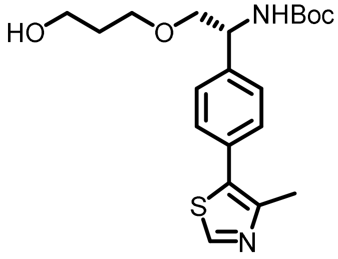

105 (1.66 g, 3.22 mmol) was dissolved in dry THF (17.0 mL) and cooled to 0 °C. $LiAlH_4$ (2 M in THF, 2.74 mL, 5.48 mmol) was added dropwise at 0 °C, keeping the temperature below 10 °C. The reaction mixture was stirred at 0 °C for 1 hour and carefully quenched at 0 °C with water. The mixture was diluted with DCM and water, filtered over a Celite pad and rinsed with DCM. The layers were separated via a phase separating cartridge and the organic solvent was removed *in vacuo* to give the desired product **106** (1.93 g, 94% yield).

^{1}H NMR (400 MHz, $CDCl_3$-*d*) δ 8.59 (s, 1H), 7.38 – 7.26 (m, 4H), 5.25 (d, J = 7.6 Hz, 1H), 4.81 (s, 1H), 3.72 – 3.45 (m, 6H), 2.45 (s, 3H), 1.83 – 1.68 (m, 2H), 1.42 – 1.33 (m, 9H)

MS (ESI) for $C_{20}H_{28}N_2O_4S$ [M +H$^+$] 393

(*R*)-3-(2-((*tert*-butoxycarbonyl)amino)-2-(4-(4-methylthiazol-5-yl)phenyl)ethoxy)propyl methanesulfonate 107

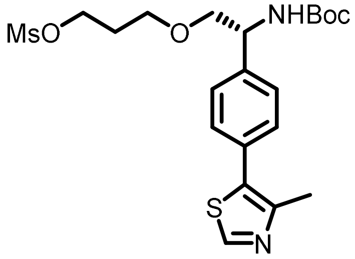

106 (1.19 g, 3.04 mmol) was dissolved in DCM (12.0 mL) and Et_3N (1.27 mL, 9.12 mmol) was added. The mixture was cooled to 0 °C and MsCl (0.47 mL, 6.08 mmol) was added slowly. The reaction mixture was stirred at 0 °C for 15 minutes and quenched with sat. $NaHCO_3$ solution. The mixture was stirred for an additional 10 minutes at RT. The layers were separated and the aqueous layer was extracted with DCM twice. The combined organic layers were dried and concentrated *in vacuo*. The crude was purified by silica gel column chromatography (0-4% MeOH in DCM) to give the desired product **107** (1.22 g, 85% yield).

^{1}H NMR (500 MHz, DMSO-d_6) δ 8.99 (s, 1H), 7.39 - 7.48 (m, 5H), 4.71 - 4.85 (m, 1H), 4.22 (t, J = 6.3 Hz, 2H), 3.47 - 3.58 (m, 4H), 3.14 (s, 3H), 2.46 (s, 3H), 1.89 (quin, J = 6.2 Hz, 2H), 1.39 (s, 9H)

MS (ESI) for $C_{21}H_{30}N_2O_6S_2$ [M+H]$^+$ 471

(*R*)-*tert*-butyl (2-(3-(4-(4-bromo-7-cyclopentyl-5-oxo-5,7-dihydrobenzo[4,5]imidazo[1,2-*a*]quinazolin-9-yl)piperidin-1-yl)propoxy)-1-(4-(4-methylthiazol-5-yl)phenyl)ethyl)carbamate 108

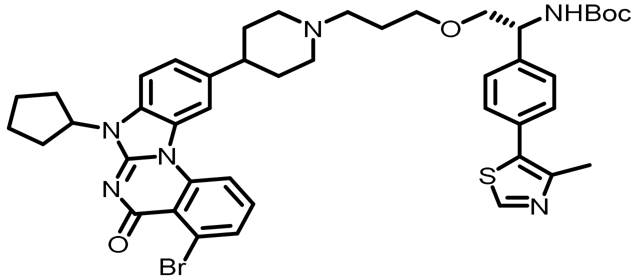

107 (175 mg, 0.37 mmol) and **4** (173 mg, 0.37 mmol) were dissolved in DMF (2.0 mL) and DIPEA (192 µL, 1.12 mmol) was added. The mixture was stirred at 80 °C for 2 hours. It was diluted with MeCN/H2O, filtered through a syringe filter and purified by basic prep.HPLC (Method 3b) to give the desired product **108** (165 mg, 53% yield).

MS (ESI) for $C_{44}H_{51}BrN_6O_4S$ $[M+H]^+$ 839

(2*S*,4*R*)-*N*-((*R*)-2-(3-(4-(4-bromo-7-cyclopentyl-5-oxo-5,7-dihydrobenzo[4,5]imidazo[1,2-*a*]quinazolin-9-yl)piperidin-1-yl)propoxy)-1-(4-(4-methylthiazol-5-yl)phenyl)ethyl)-1-((*S*)-2-(1-fluorocyclopropanecarboxamido)-3,3-dimethylbutanoyl)-4-hydroxypyrrolidine-2-carboxamide 27

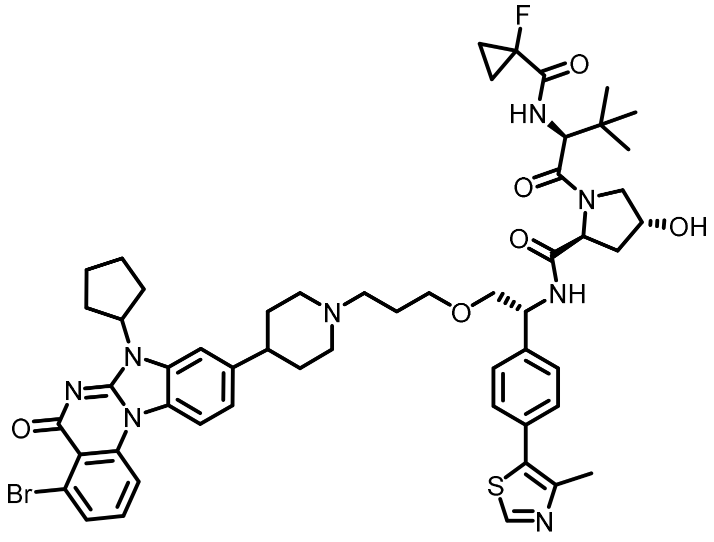

108 (165 mg, 0.20 mmol) was taken up in MeOH (2.0 mL) and conc. HCl (1.0 mL, 11.65 mmol) was added. The mixture was stirred at RT for 2 hours. The solvents were removed *in vacuo*. The crude amine was taken to the next step without further purification. **52** (33 mg, 0.10 mmol) was dissolved in DMF (1.0 mL) and Et_3N (39 µL, 0.27 mmol) and HATU (77 mg, 0.20 mmol) were added. The mixture was stirred at RT for 15 minutes. Then the crude amine (50 mg, 0.068 mmol) was added and the mixture was stirred at RT for 5 hours. The reaction mixture was diluted with MeCN/H_2O, filtered through a syringe filter and purified by basic prep HPLC (Method 3b) to give the desired product, **27** (39 mg, 55% yield).

^1H NMR (400 MHz, DMSO-*d*$_6$) δ 8.99 (s, 1H), 8.53 (d, J = 8.1 Hz, 1H), 8.48 (d, J = 8.9 Hz, 1H), 8.29 (d, J = 8.6 Hz, 1H), 7.80 (d, J = 7.4 Hz, 1H), 7.68 (t, J = 8.1 Hz, 1H), 7.53 (s, 1H), 7.40 - 7.49 (m, 4H), 7.27 (br d, J = 8.6

Hz, 2H), 5.25 (quin, J = 8.8 Hz, 1H), 5.17 (br s, 1H), 4.97 - 5.07 (m, 1H), 4.59 (d, J = 8.9 Hz, 1H), 4.55 (t, J = 8.2 Hz, 1H), 4.29 (br s, 1H), 3.53 - 3.73 (m, 5H), 3.48 (br t, J = 6.3 Hz, 3H), 2.94 (br d, J = 11.2 Hz, 2H), 2.47 (s, 3H), 2.22 - 2.32 (m, 4H), 1.90 - 2.14 (m, 7H), 1.62 - 1.87 (m, 8H), 1.29 - 1.48 (m, 2H), 1.22 (dd, J = 8.1, 2.8 Hz, 2H), 0.99 (s, 9H)

^{13}C NMR (150 MHz, DMSO-*d*$_6$) δ 171.4, 169.4, 168.6 (d, CF=19.1 Hz), 164.8, 152.1, 149.4, 148.3, 141.0, 139.2, 133.9, 132.8, 131.5, 130.8, 130.6, 129.2, 127.8, 125.7, 123.3, 121.4, 117.0, 115.8, 114.3, 109.7, 78.7 (d, CF=232.7 Hz), 73.0, 69.2, 68.0, 59.1, 57.1, 57.1, 54.4, 52.6, 38.2, 36.5, 28.5, 26.7, 26.6, 25.1, 16.4, 13.3 (m, CF)

HRMS (m/z) for $C_{54}H_{64}BrFN_8O_6S$ $[M+H]^+$ calculated 1051.39097; obtained 1051.38941

Supplementary Figure 29: synthesis of PROTAC 28

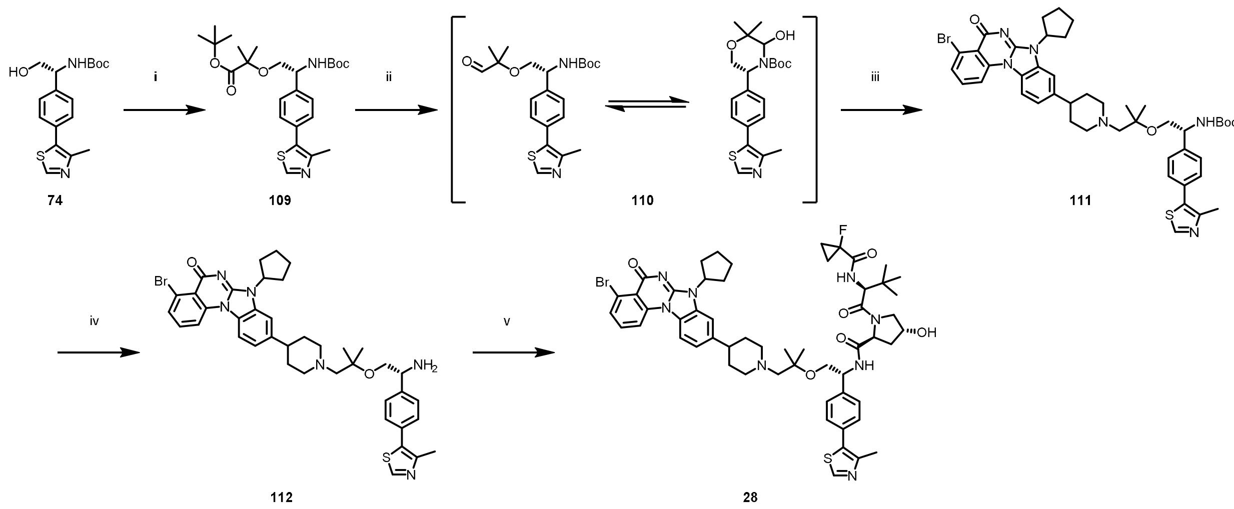

i) *tert*-butyl 2-bromo-2-methylpropanoate, tetrabutylammonium hydrogen sulfate, DCM, NaOH, 50 °C ; ii) $LiAlH_4$, THF, -20 °C ; iii) **4**, $NaBH(OAc)_3$, DCE, 60 °C ; iv) HCl, MeOH, 55 °C ; v) **52**, HATU, Et_3N, DMF, RT.

(*R*)-*tert*-butyl 2-(2-((*tert*-butoxycarbonyl)amino)-2-(4-(4-methylthiazol-5-yl)phenyl)ethoxy)-2-methylpropanoate 109

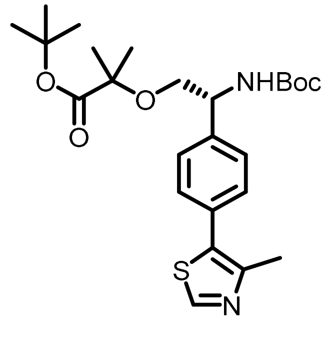

74 (10.00 g, 29.90 mmol) and tetrabutylammonium hydrogen sulfate (5.08 g, 14.95 mmol) were dissolved in DCM (190.0 mL) and 4 M aq. NaOH (180.0 mL). *tert*-Butyl 2-bromo-2-methylpropanoate (55.8 mL; 29.90 mmol) was added dropwise. The reaction mixture was stirred for 12 hours at 50 °C. It was diluted with DCM and water, the layers were separated and the organic solvent was removed *in vacuo*. The crude

was purified by silica gel column chromatography (0-70% EtOAc in cyclohexane) to give the desired product **109** (1.80 g, 13% yield).

^{1}H NMR (500 MHz, DMSO-*d$_6$*) δ 8.99 (s, 1H), 7.43 - 7.47 (m, 2H), 7.38 - 7.42 (m, 2H), 7.20 (br d, J = 8.2 Hz, 1H), 4.59 - 4.72 (m, 1H), 3.42 - 3.55 (m, 2H), 2.46 (s, 3H), 1.41 (s, 9H), 1.34 - 1.40 (m, 9H), 1.26 (d, J = 10.1 Hz, 6H)

MS (ESI) for $C_{25}H_{36}N_2O_5S$ $[M+H]^+$ 335

(*R*)-*tert*-butyl (2-((2-methyl-1-oxopropan-2-yl)oxy)-1-(4-(4-methylthiazol-5-yl)phenyl)ethyl)carbamate 110

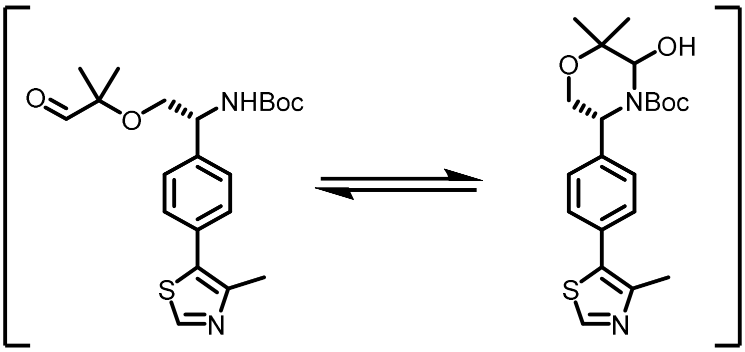

109 (3.00 g, 6.29 mmol) was dissolved in dry THF (60.0 mL) and cooled to -20 °C. Then $LiAlH_4$ (2 M in THF, 3.15 mL, 6.29 mmol) was added dropwise and the reaction mixture was stirred at -20 °C for 1 hour. It was quenched cautiously with water and extracted with DCM. The layers were separated and the organic solvent was removed *in vacuo*. The crude was purified by silica gel column chromatography (0-2.5% MeOH in DCM) to give the desired product **110** (1.94 g, 76% yield).

^{1}H NMR (500 MHz, DMSO-*d$_6$*) δ 8.99 (s, 1H), 7.56 (d, J = 8.2 Hz, 2H), 7.43 (d, J = 8.2 Hz, 2H), 6.61 (br d, J = 4.4 Hz, 1H), 5.35 (d, J = 5.0 Hz, 1H), 4.80 (dd, J = 10.6, 5.8 Hz, 1H), 3.90 (t, J = 11.0 Hz, 1H), 3.66 (dd, J = 11.5, 5.8 Hz, 1H), 2.45 (s, 3H), 1.21 (br s, 3H), 1.20 (br s, 9H), 1.14 (s, 3H)

MS (ESI) for $C_{21}H_{28}N_2O_4S$ $[M+H]^+$ 405

(*R*)-*tert*-butyl (2-((1-(4-(4-bromo-7-cyclopentyl-5-oxo-5,7-dihydrobenzo[4,5]imidazo[1,2-*a*]quinazolin-9-yl)piperidin-1-yl)-2-methylpropan-2-yl)oxy)-1-(4-(4-methylthiazol-5-yl)phenyl)ethyl)carbamate 111

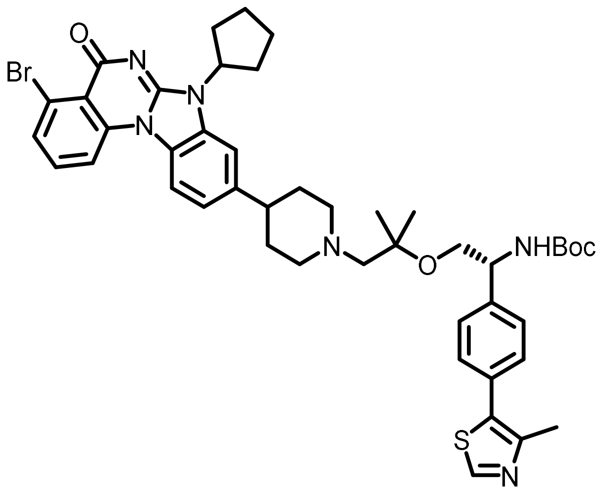

4 (1.13 g, 2.00 mmol) was dissolved in DCE (5.0 mL). The reaction mixture was heated to 60 °C and $NaBH(OAc)_3$ (1.41 g, 6.65 mmol) and **110** (0.54 g, 1.33 mmol) (dissolved in DCE) were added. The reaction mixture was stirred at 60 °C for 30 min. Another 1 equivalent of $NaBH(OAc)_3$ was added and the reaction mixture stirred for another 1 hour at 60 °C. It was cooled to RT and DCM and water was added. The layers were separated and the organic solvent was removed *in vacuo*. The crude was purified by basic HPLC (Method 3b) to give the desired product **111** (694 mg, 61% yield).

^{1}H NMR (500 MHz, DMSO-*d*$_6$) δ 8.94 (s, 1H), 8.47 (d, J = 8.2 Hz, 1H), 8.27 (d, J = 8.5 Hz, 1H), 7.80 (d, J = 7.6 Hz, 1H), 7.69 (t, J = 8.2 Hz, 1H), 7.50 (s, 1H), 7.39 - 7.45 (m, 4H), 7.29 (br d, J = 8.5 Hz, 1H), 7.23 (br d, J = 8.5 Hz, 1H), 5.24 (quin, J = 8.7 Hz, 1H), 4.62 (br d, J = 7.6 Hz, 1H), 3.45 - 3.53 (m, 2H), 2.97 (br dd, J = 27.4, 10.4 Hz, 2H), 2.58 - 2.62 (m, 1H), 2.42 (s, 3H), 2.23 - 2.32 (m, 4H), 2.08 - 2.16 (m, 2H), 2.01 (br d, J = 2.8 Hz, 4H), 1.65 - 1.77 (m, 6H), 1.37 (s, 9H), 1.06 - 1.17 (m, 6H)

MS (ESI) for $C_{45}H_{53}BrN_6O_4S$ $[M+H]^+$ 853

(*R*)-9-(1-(2-(2-amino-2-(4-(4-methylthiazol-5-yl)phenyl)ethoxy)-2-methylpropyl)piperidin-4-yl)-4-bromo-7-cyclopentylbenzo[4,5]imidazo[1,2-*a*]quinazolin-5(7*H*)-one 112

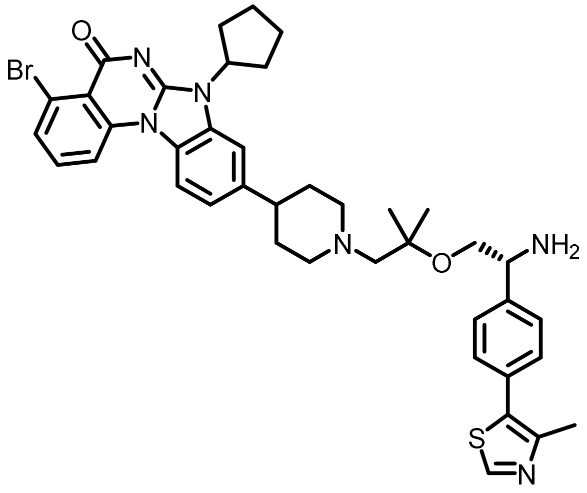

111 (163 mg, 0.19 mmol) was dissolved in MeOH (3.0 mL) and 4 M HCl in 1,4-dioxane (1.0 mL, 4.00 mmol) was added. The mixture was stirred at 55 °C for 1 hour. The reaction mixture was concentrated to dryness, dissolved in MeCN/H_2O, basified with aq. NH_3 and purified by basic HPLC (Method 3b) to give the desired product **112** (117 mg, 81% yield).

^{1}H NMR (400 MHz, DMSO-*d$_6$*) δ 8.94 (s, 1H), 8.47 (d, J = 8.4 Hz, 1H), 8.27 (d, J = 8.4 Hz, 1H), 7.81 (d, J = 7.9 Hz, 1H), 7.64 - 7.75 (m, 1H), 7.51 (s, 1H), 7.46 - 7.50 (m, 2H), 7.39 - 7.44 (m, 2H), 7.25 (d, J = 8.9 Hz, 1H), 5.25 (quin, J = 8.7 Hz, 1H), 3.96 (br t, J = 6.0 Hz, 1H), 2.99 (br t, J = 10.1 Hz, 2H), 2.59 - 2.66 (m, 1H), 2.42 (s, 3H), 2.23 - 2.32 (m, 4H), 2.15 (br s, 2H), 2.02 (br s, 4H), 1.72 (br d, J = 7.1 Hz, 6H), 1.13 (d, J = 7.1 Hz, 6H)

MS (ESI) for $C_{40}H_{45}BrN_6O_2S$ $[M+H]^+$ 753

(2S,4R)-N-[(1R)-2-{[1-(4-{6-bromo-11-cyclopentyl-8-oxo-1,9,11-triazatetracyclo[8.7.0.0^{2},^{7}.0^{12},^{17}]heptadeca-2,4,6,9,12,14,16-heptaen-14-yl}piperidin-1-yl)-2-methylpropan-2-yl]oxy}-1-[4-(4-methyl-1,3-thiazol-5-yl)phenyl]ethyl]-1-[(2S)-2-[(1-fluorocyclopropyl)formamido]-3,3-dimethylbutanoyl]-4-hydroxypyrrolidine-2-carboxamide 28

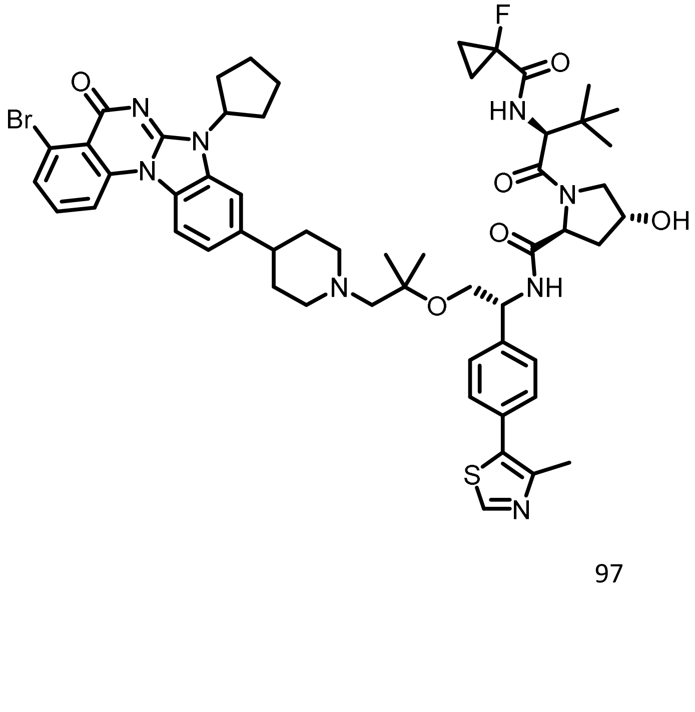

52 (13 mg, 0.038 mmol) was dissolved in DMF (0.50 mL) and Et_3N (17 µl, 0.12 mmol) and HATU (17 mg, 0.044 mmol) was added. The mixture was stirred at RT for 5 minutes and then **112** (22 mg, 0.029 mmol) was added. The reaction mixture was stirred at RT for 1 hour. It was diluted with MeCN/H_2O, filtered through a syringe filter and purified by basic HPLC (Method 3b) to give the desired product **28** (25 mg, 80% yield).

^{1}H NMR (600 MHz, DMSO-d_6) δ 8.93 (s, 1H), 8.46 (d, J = 8.4 Hz, 1H), 8.36 (d, J = 8.3 Hz, 1H), 8.25 (d, J = 8.6 Hz, 1H), 7.80 (d, J = 7.7 Hz, 1H), 7.68 (t, J = 8.1 Hz, 1H), 7.48 (s, 1H), 7.42 (s, 4H), 7.25 (dd, J = 9.2, 2.4 Hz, 1H), 7.21 (d, J = 8.8 Hz, 1H), 5.24 (quin, J = 8.7 Hz, 1H), 5.14 (br s, 1H), 4.85 - 4.92 (m, 1H), 4.58 (d, J = 9.2 Hz, 1H), 4.53 (t, J = 8.3 Hz, 1H), 4.29 (br s, 1H), 3.52 - 3.67 (m, 3H), 2.92 (br t, J = 11.1 Hz, 2H), 2.55 - 2.61 (m, 1H), 2.41 (s, 3H), 2.23 - 2.32 (m, 5H), 1.96 - 2.17 (m, 7H), 1.55 - 1.87 (m, 7H), 1.27 - 1.47 (m, 2H), 1.21 (br dd, J = 8.1, 3.1 Hz, 2H), 1.12 (d, J = 12.1 Hz, 6H), 0.95 - 1.00 (m, 9H)

^{13}C NMR (150 MHz, DMSO-d_6) δ 170.2, 168.2, 167.4 (d, CF = 20.4 Hz), 163.5, 150.8, 148.2, 147.1, 143.0, 140.4, 138.2, 132.7, 131.5, 130.5, 129.9, 129.4, 127.9, 127.1, 124.1, 122.2, 120.5, 116.0, 114.7, 112.9, 108.5, 77.5 (d, CF = 232.7 Hz), 75.6, 68.2, 65.4, 63.7, 58.1, 56.0, 55.9, 54.9, 54.9, 53.4, 52.5, 41.0, 37.1, 35.5, 33.0, 33.0, 27.4, 25.7, 25.6, 24.0, 23.0, 22.8, 15.3, 12.2 (m, CF)

HRMS (m/z) for $C_{55}H_{66}BrFN_8O_6S$ $[M+H]^+$ calculated 1065.40662; obtained 1065.40369

Supplementary Figure 30: synthesis of PROTAC 30

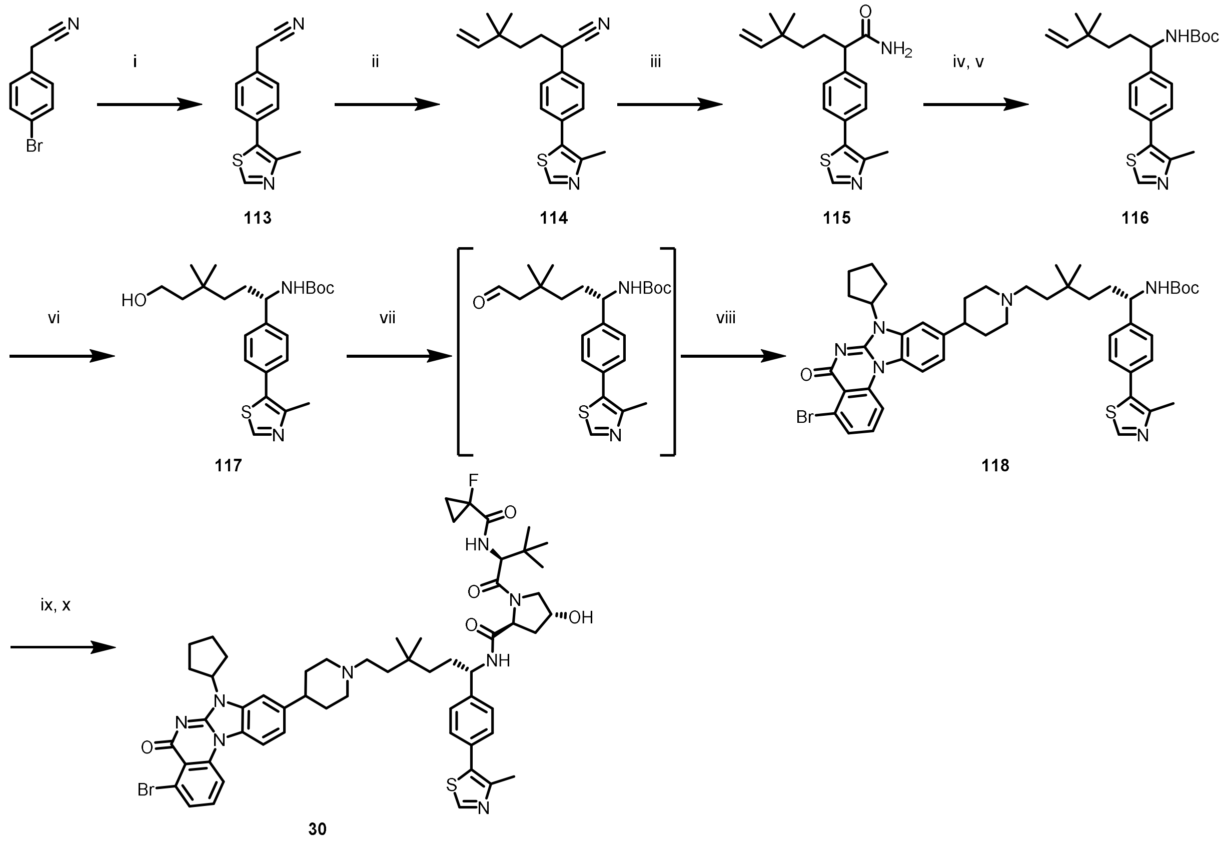

i) 4-methylthiazole, KOAc, Pd(OAc)$_2$, DMA, 120 °C ; ii) 5-iodo-3,3-dimethylpent-1-ene, LiHMDS, THF, -78 °C to -20 °C ; iii) NaOH, MeOH/H_2O, 100 °C ; iv) (*bis*(trifluoroacetoxy)iodo)benzene, MeCN/H_2O, RT ; v) Boc_2O, Et_3N, 0 °C ; vi) 9-

BBN, THF, 0 °C to RT, then 30% aq.H_2O_2, 4 M NaOH, 0 °C to RT ; vii) (diacetoxyiodo)benzene, TEMPO, DCM, RT ; viii) **4**, DCE, 50 °C, then $NaBH(OAc)_3$; ix) HCl, MeOH, RT ; x) **52**, HATU, DIPEA, DMF, RT

2-(4-(4-methylthiazol-5-yl)phenyl)acetonitrile 113

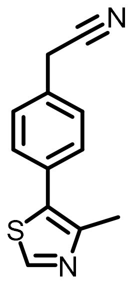

4-Bromophenylacetonitrile (25.0 g, 0.13 mol), 4-methylthiazole (25.3 g, 0.26 mol) and KOAc (25.0 g, 0.26 mol) were taken up in DMA (200.0 mL) and purged with argon for 15 minutes. Then $Pd(OAc)_2$ (0.69 g, 1.28 mmol) was added and the mixture was stirred at 120 °C for 16 hours. The reaction mixture was allowed to cool to room temperature and quenched with ice cold water. The resulting precipitate was collected by filtration and dried under vacuum. It was purified by column chromatography (20-50% EtOAc in petroleum ether) to give the desired product **113** (13.0 g, 48% yield).

^{1}H NMR (400 MHz, $CDCl_3$-*d*) δ 8.71 (s, 1H), 7.45 - 7.50 (m, 2H), 7.38 - 7.43 (m, 2H), 3.80 (s, 2H), 2.54 (s, 3H)

MS (ESI) for $C_{12}H_{10}N_2S$ $[M+H]^+$ 215

5,5-dimethyl-2-(4-(4-methylthiazol-5-yl)phenyl)hept-6-enenitrile 114

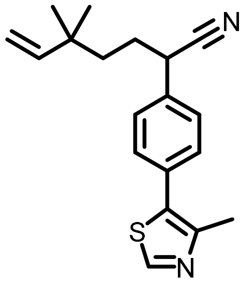

113 (11.0 g, 51.3 mmol) was dissolved in THF (110.0 mL) and cooled to -78 °C. LiHMDS (1 M in THF, 154.0 mL, 154.0 mmol) was added dropwise and it was stirred at the same temperature for 20 minutes. Then 5-iodo-3,3-dimethylpent-1-ene (15.0 g, 66.9 mmol) was added dropwise and the reaction mixture was slowly warmed to -20°C and stirred for 30 min. It was cooled to -78 °C again and slowly quenched with sat. aq. NH_4Cl solution and extracted with EtOAc twice. The combined organic layers were dried over Na_2SO_4 and concentrated *in vacuo*. The obtained crude was purified by silica gel column chromatography (20-40% EtOAc in petroleum ether) to give the desired product **114** (8.0 g, 47% yield).

^{1}H NMR (500 MHz, DMSO-*d$_6$*) δ 9.02 (s, 1H), 7.52 - 7.58 (m, 2H), 7.45 - 7.50 (m, 2H), 5.74 (dd, J = 17.5, 10.8 Hz, 1H), 4.87 - 4.96 (m, 2H), 4.26 (dd, J = 8.4, 6.4 Hz, 1H), 2.47 (s, 3H), 1.70 - 1.83 (m, 2H), 1.34 - 1.50 (m, 2H), 0.96 (s, 6H)

MS (ESI) for $C_{19}H_{22}N_2S$ $[M+H]^+$ 311

5,5-dimethyl-2-(4-(4-methylthiazol-5-yl)phenyl)hept-6-enamide 115

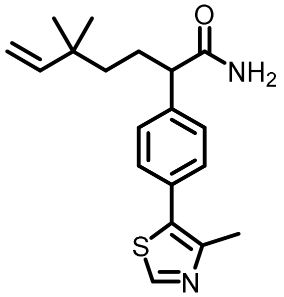

A mixture of **114** (6.00 g, 19.3 mmol) and sodium hydroxide (7.73 g, 193 mmol) in MeOH (90.0 mL) and water (30.0 mL) was refluxed at 100 °C for 4 hours. The reaction mixture was concentrated *in vacuo*. The obtained residue was dissolved in water and extracted with EtOAc twice. The combined organic layers were dried over Na_2SO_4 and concentrated *in vacuo*. The obtained crude is washed with a mixture of n-pentane and diethyl ether to give the desired product **115** (4.00 g, 63% yield).

^{1}H NMR (400 MHz, DMSO-*d$_6$*) δ 8.96 (d, J = 1.0 Hz, 1H), 7.48 (br s, 1H), 7.33 - 7.45 (m, 4H), 6.74 - 6.97 (m, 1H), 5.63 - 5.79 (m, 1H), 4.78 - 4.99 (m, 2H), 2.43 (d, J = 1.0 Hz, 3H), 1.77 - 1.90 (m, 1H), 1.41 - 1.55 (m, 1H), 1.24 (td, J = 12.6, 3.7 Hz, 1H), 1.05 - 1.16 (m, 1H), 0.92 (s, 6H)

MS (ESI) for $C_{19}H_{24}N_2OS$ $[M+H]^+$ 329

***tert*-butyl (4,4-dimethyl-1-(4-(4-methylthiazol-5-yl)phenyl)hex-5-en-1-yl)carbamate 116**

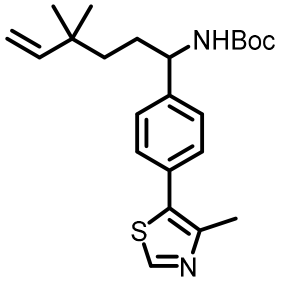

115 (2.20 g, 6.69 mmol) was dissolved in MeCN (50.0 mL) and water (17.0 mL). (*Bis*(trifluoroacetoxy)iodo)benzene (3.46 g, 8.05 mmol) was added and the reaction mixture was stirred at RT for 16 hours. It was cooled to 0 °C and Et_3N (1.88 mL, 13.3 mmol) and Boc_2O (2.31 mL, 10.0 mmol) were added. Stirring was continued at RT for 4 hours. The reaction mixture was diluted with water and extracted with EtOAc twice. The combined organic layers were dried over Na_2SO_4 and concentrated *in vacuo*. The obtained crude was purified by silica gel column chromatography (20-40% EtOAc in petroleum ether) to give the desired product **116** (2.20 g, 82% yield).

^{1}H NMR (500 MHz, DMSO-*d$_6$*) δ 8.98 (s, 1H), 7.42 - 7.45 (m, 2H), 7.40 (br d, J = 8.5 Hz, 1H), 7.36 (br d, J = 8.1 Hz, 2H), 5.73 (dd, J = 17.5, 10.8 Hz, 1H), 4.81 - 4.92 (m, 2H), 4.38 - 4.45 (m, 1H), 2.46 (s, 3H), 1.44 - 1.63 (m, 2H), 1.36 (s, 9H), 1.20 - 1.29 (m, 1H), 1.13 - 1.19 (m, 1H), 0.93 (s, 6H)

MS (ESI) for $C_{23}H_{32}N_2O_2S$ $[M+H]^+$ 401

(*S*)-*tert*-butyl (6-hydroxy-4,4-dimethyl-1-(4-(4-methylthiazol-5-yl)phenyl)hexyl)carbamate 117

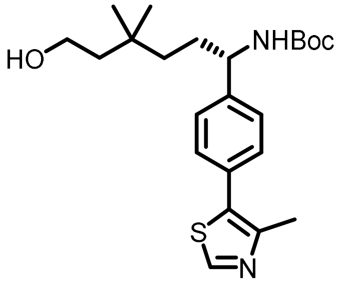

116 (3.00 g, 7.49 mmol) was dissolved in THF (30.0 mL) and cooled to 0 °C. Then 9-BBN (0.5 M in THF, 44.9 mL, 22.4 mmol) was added slowly at 0 °C. After complete addition, the cooling was removed and the mixture was stirred at RT for 2 hours until complete conversion of the alkene. The reaction mixture was cooled to 0 °C again and 30% aq.H_2O_2 solution (2.55 g, 74.9 mmol) was added dropwise, followed by 4 M aq. NaOH solution (18.7 mL, 74.9 mmol). The cooling was removed and the mixture was stirred at RT for 1 hour. The reaction was slowly quenched and acidified to pH 4 by carefully adding 1 M aq. HCl. It was extracted with DCM twice. The combined organic layers were dried over Na_2SO_4 and concentrated *in vacuo*. The obtained crude product was purified by silica gel column chromatography (0-10% MeOH in DCM) to give the desired product (1.40 g, 45% yield) as a racemic mixture, which was further purified by SFC ((*R*,*R*)Whelk-O1, 25% MeOH) to obtain the desired product as a single enantiomer **117** (0.39 g of Peak 1, 29% recovery).

^{1}H NMR (400 MHz, DMSO-d_6) δ 8.95 (s, 1H), 7.36 - 7.42 (m, 3H), 7.32 - 7.36 (m, 2H), 4.32 - 4.44 (m, 1H), 4.19 (br s, 1H), 4.07 (br s, 1H), 3.35 (br s, 2H), 3.13 (s, 1H), 2.42 (s, 3H), 1.45 - 1.61 (m, 2H), 1.17 - 1.37 (m, 10H), 1.02 (td, J = 12.6, 4.2 Hz, 1H), 0.77 (s, 6H)

MS (ESI) for $C_{23}H_{34}N_2O_3S$ $[M+H]^+$ 419

(*S*)-*tert*-butyl (6-(4-(4-bromo-7-cyclopentyl-5-oxo-5,7-dihydrobenzo[4,5]imidazo[1,2-*a*]quinazolin-9-yl)piperidin-1-yl)-4,4-dimethyl-1-(4-(4-methylthiazol-5-yl)phenyl)hexyl)carbamate 118

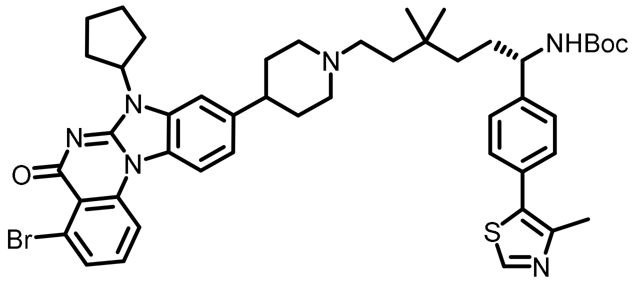

117 (50 mg, 0.12 mmol) was dissolved in DCM (1.5 mL) and (diacetoxyiodo)benzene (50 mg, 0.15 mmol) and TEMPO (5 mg, 0.03 mmol) were added. The mixture was stirred at RT overnight. The reaction mixture was diluted with DCM and purified by silica gel column chromatography (0-2% MeOH in DCM) to give the desired aldehyde in sufficient purity for the next step (38 mg, 77% yield).

4 (28 mg, 0.06 mmol) was suspended in DCE (600 µL) and the aldehyde (17 mg, 0.04 mmol) was added. The mixture was heated to 50 °C, $NaBH(OAc)_3$ (17 mg, 0.08 mmol) was added at this temperature and the

mixture was stirred at 50 °C for 30 min. The reaction was cooled to RT and water was added. The solvents were removed completely *in vacuo*. The residue was dissolved in water/methanol and basified. It was purified by basic HPLC (Method 3b) to give the desired product, **118** (33 mg, 93% yield).

MS (ESI) for $C_{47}H_{57}BrN_6O_3S$ $[M+H]^+$ 866

(2*S*,4*R*)-*N*-((1*S*,4*S*)-6-(4-(4-bromo-7-cyclopentyl-5-oxo-5,7-dihydrobenzo[4,5]imidazo[1,2-*a*]quinazolin-9-yl)piperidin-1-yl)-4-methyl-1-(4-(4-methylthiazol-5-yl)phenyl)hexyl)-1-((*S*)-2-(1-fluorocyclopropanecarboxamido)-3,3-dimethylbutanoyl)-4-hydroxypyrrolidine-2-carboxamide 30

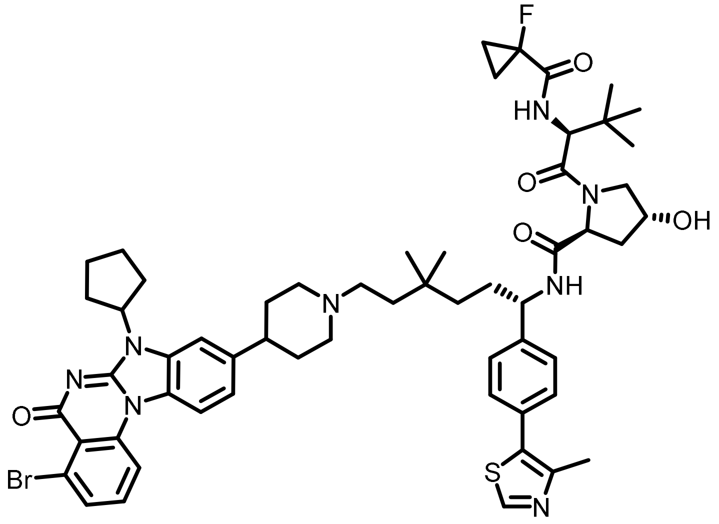

118 (33 mg, 0.04 mmol) was dissolved in MeOH (1.0 mL) and conc. HCl (1.0 mL) was added. The reaction mixture was stirred for 30 min at RT and concentrated *in vacuo* to give the crude amine (*S*)-9-(1-(6-amino-3,3-dimethyl-6-(4-(4-methylthiazol-5-yl)phenyl)hexyl)piperidin-4-yl)-4-bromo-7-cyclopentylbenzo[4,5]imidazo[1,2-*a*]quinazolin-5(7*H*)-one hydrochloride which was used for the next step without further purification.

52 (18 mg, 0.05 mmol), the crude amine (31 mg, 0.04 mmol) and HATU (22 mg, 0.06 mmol) were dissolved in DMF (1.0 mL) and DIPEA (39 µL, 0.23 mmol) was added. The solution was stirred for 30 minutes at RT. The mixture was diluted with MeCN/water, filtered and purified by basic HPLC to give the desired product **30** (25 mg, 61% yield).

^1H NMR (600 MHz, DMSO-d_6) δ 8.98 (s, 1H), 8.47 (d, J = 8.4 Hz, 1H), 8.44 (d, J = 8.4 Hz, 1H), 8.29 (d, J = 8.6 Hz, 1H), 7.80 (d, J = 7.7 Hz, 1H), 7.68 (t, J = 8.2 Hz, 1H), 7.52 (s, 1H), 7.44 - 7.47 (m, 2H), 7.38 - 7.42 (m, 2H), 7.22 - 7.29 (m, 2H), 5.20 - 5.35 (m, 1H), 5.14 (d, J = 3.5 Hz, 1H), 4.72 - 4.81 (m, 1H), 4.58 (d, J = 9.2 Hz, 1H), 4.51 (t, J = 8.3 Hz, 1H), 4.29 (br s, 1H), 3.54 - 3.63 (m, 2H), 2.92 - 3.06 (m, 2H), 2.65 - 2.75 (m, 1H), 2.46 (s, 3H), 2.19 - 2.31 (m, 4H), 1.92 - 2.07 (m, 7H), 1.58 - 1.86 (m, 9H), 1.29 - 1.51 (m, 5H), 1.22 (br dd, J = 8.4, 2.8 Hz, 2H), 1.07 - 1.17 (m, 1H), 0.98 (s, 9H), 0.86 (s, 6H)

^{13}C NMR (150 MHz, DMSO-d_6) δ 170.0, 168.1, 167.4 (d, CF = 20.4 Hz), 163.5, 150.9, 148.2, 147.2, 143.2, 143.0, 138.2, 132.7, 131.5, 130.5, 129.8, 129.2, 128.2, 126.4, 124.2, 122.2, 120.6, 116.0, 114.8, 113.0, 108.5, 77.6 (d, CF = 232.7 Hz), 68.2, 58.1, 56.0, 55.9, 53.4, 53.3, 53.3, 53.1, 52.4, 37.1, 37.0, 35.6, 32.8, 31.1, 30.6, 27.4, 26.7, 26.7, 25.7, 25.6, 23.9, 15.4, 12.2 (m, CF)
HRMS (m/z) for $C_{57}H_{70}BrFN_8O_5S$ $[M+H]^+$ calculated 1077.44300; obtained 1077.44185

Supplementary Figure 31: synthesis of PROTAC 31

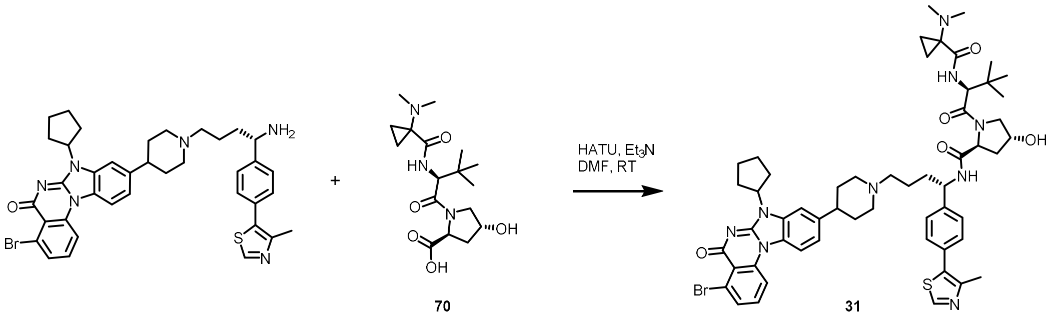

(2*S*,4*R*)-*N*-((*S*)-4-(4-(4-bromo-7-cyclopentyl-5-oxo-5,7-dihydrobenzo[4,5]imidazo[1,2-*a*]quinazolin-9-yl)piperidin-1-yl)-1-(4-(4-methylthiazol-5-yl)phenyl)butyl)-1-((*S*)-2-(1-(dimethylamino)cyclopropanecarboxamido)-3,3-dimethylbutanoyl)-4-hydroxypyrrolidine-2-carboxamide 31

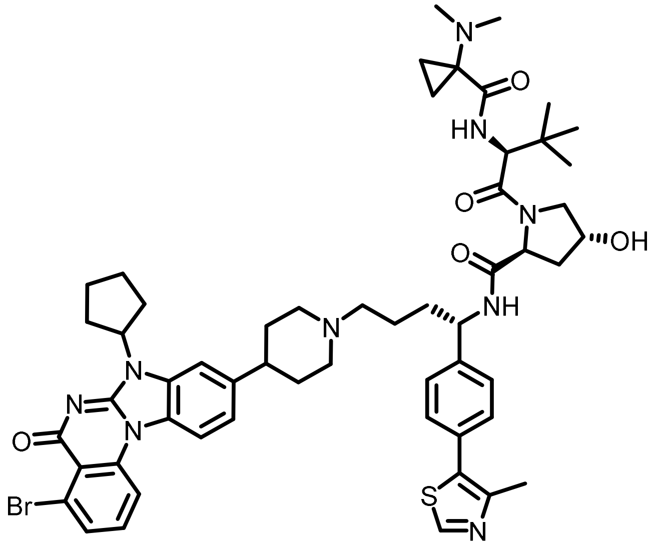

31 was prepared using acid **70** according to the same procedures as for **6**.

^{1}H NMR (600 MHz, DMSO-d_6) δ 8.97 - 9.01 (m, 1H), 8.49 (br d, J = 8.4 Hz, 1H), 8.47 (br d, J = 8.4 Hz, 1H), 8.29 (br d, J = 8.6 Hz, 1H), 8.10 (br d, J = 9.7 Hz, 1H), 7.79 (d, J = 7.7 Hz, 1H), 7.67 (t, J = 8.1 Hz, 1H), 7.54 (s, 1H), 7.44 - 7.50 (m, 2H), 7.40 (br d, J = 8.1 Hz, 2H), 7.28 (br d, J = 8.4 Hz, 1H), 5.20 - 5.32 (m, 1H), 5.12 (br d, J = 2.4 Hz, 1H), 4.83 - 4.92 (m, 1H), 4.45 - 4.55 (m, 2H), 4.30 (br s, 1H), 3.51 - 3.64 (m, 2H), 3.06 (br dd, J = 31.7, 10.1 Hz, 2H), 2.70 (br t, J = 11.3 Hz, 1H), 2.47 (s, 4H), 2.21 - 2.38 (m, 5H), 2.13 (s, 6H), 1.95 - 2.05 (m, 8H), 1.66 - 1.89 (m, 11H), 1.43 - 1.52 (m, 1H), 0.96 (s, 9H)

^{13}C NMR (150 MHz, DMSO-d_6) δ 171.4, 170.2, 168.6, 163.5, 150.9, 148.2, 147.2, 143.4, 143.0, 138.2, 132.7, 131.5, 130.5, 129.8, 129.1, 128.2, 126.2, 124.2, 122.2, 120.6, 116.0, 114.8, 113.0, 108.5, 68.2, 58.0, 57.4, 56.0, 55.7, 53.3, 53.3, 53.1, 51.4, 46.8, 41.5, 41.1, 37.2, 35.6, 33.9, 32.9, 27.3, 25.8, 25.6, 24.0, 22.7, 15.4, 11.6, 9.8

HRMS (m/z) for $C_{55}H_{68}BrN_9O_5S$ $[M+H]^+$ calculated 1046.43202; obtained 1046.42942

^{1}H and ^{13}C 1D NMR spectra for compounds 1-31

Supplementary Figure 32: ^{1}H and ^{13}C spectra of **1**

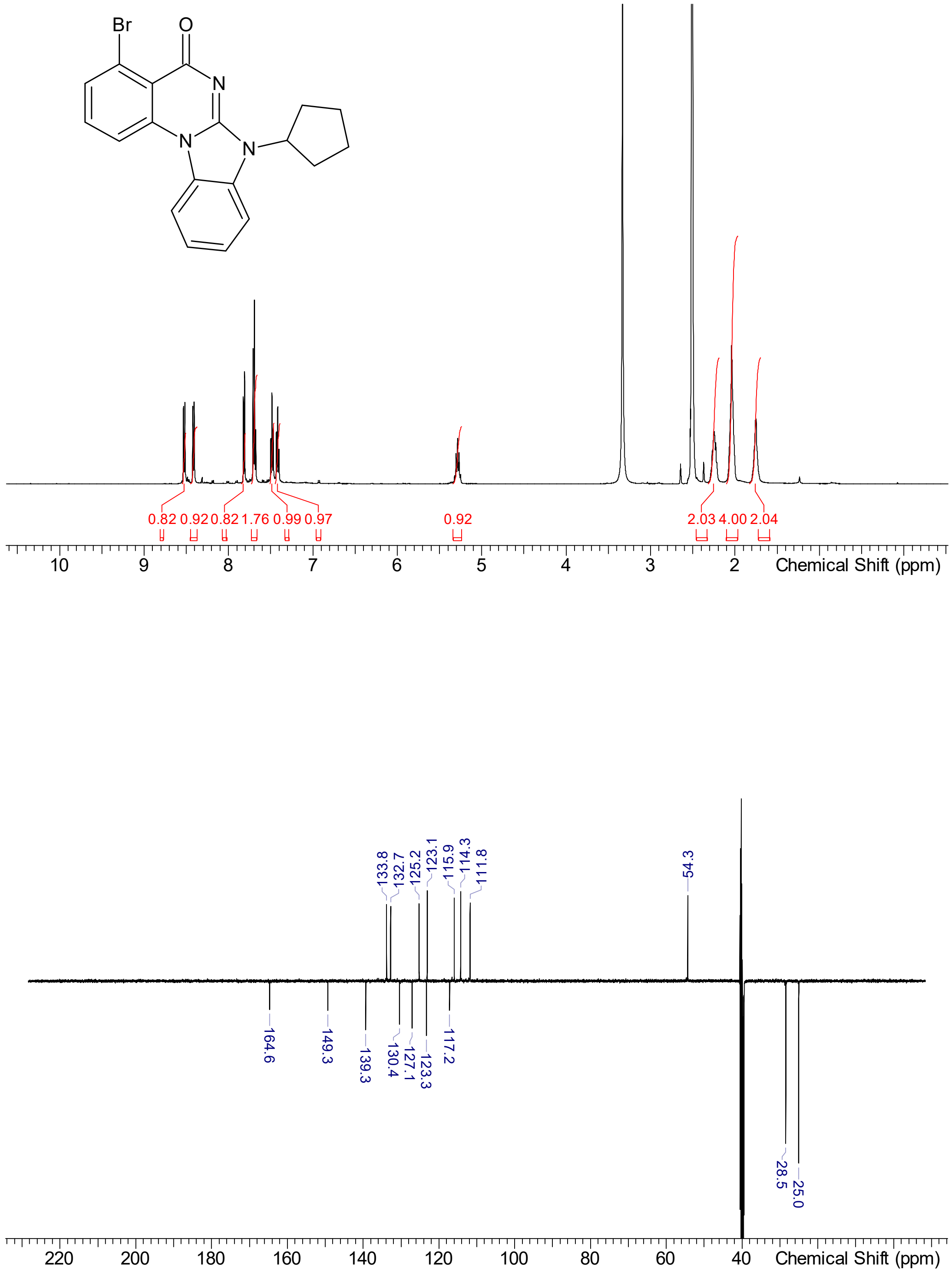

Supplementary Figure 33: ^{1}H and ^{13}C spectra of **2**

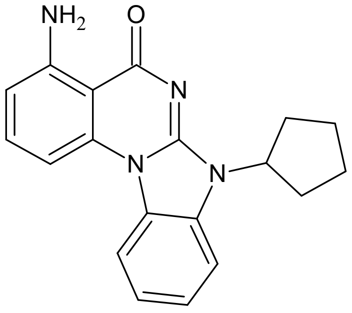

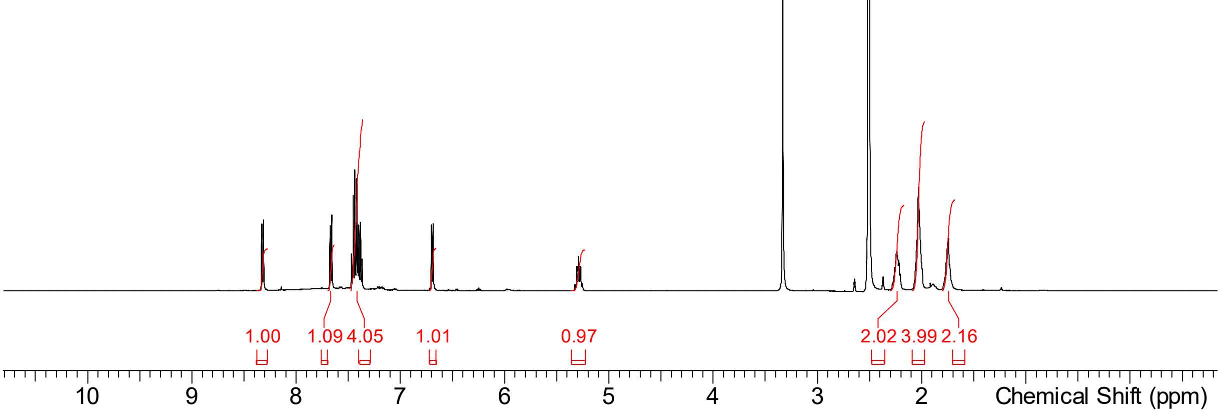

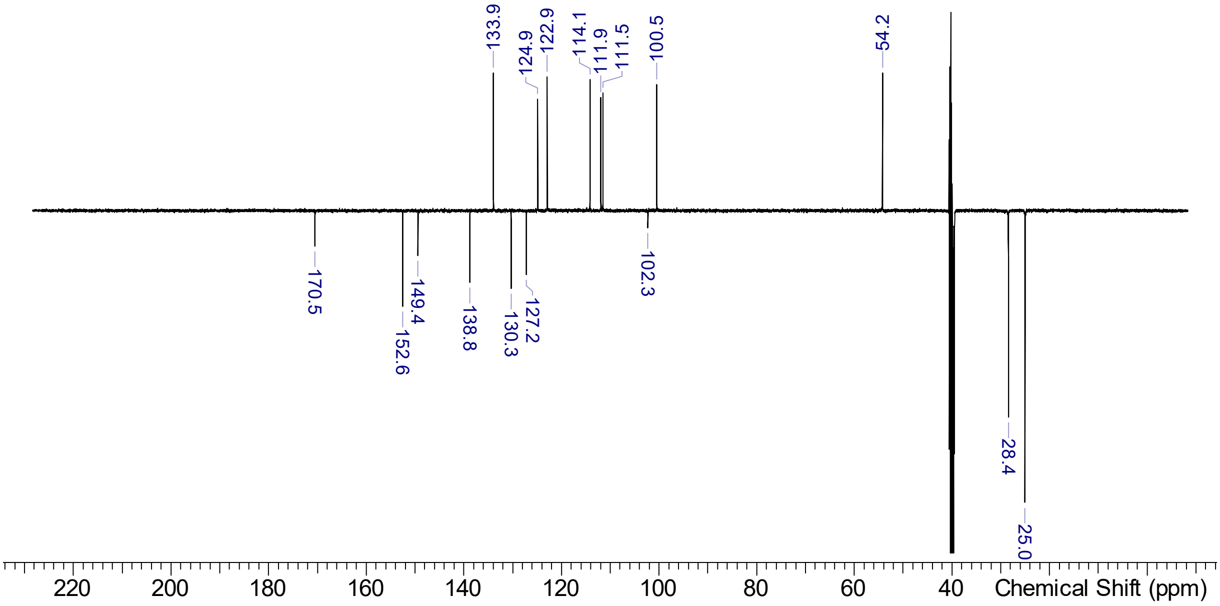

Supplementary Figure 34: ^{1}H and ^{13}C spectra of **3**

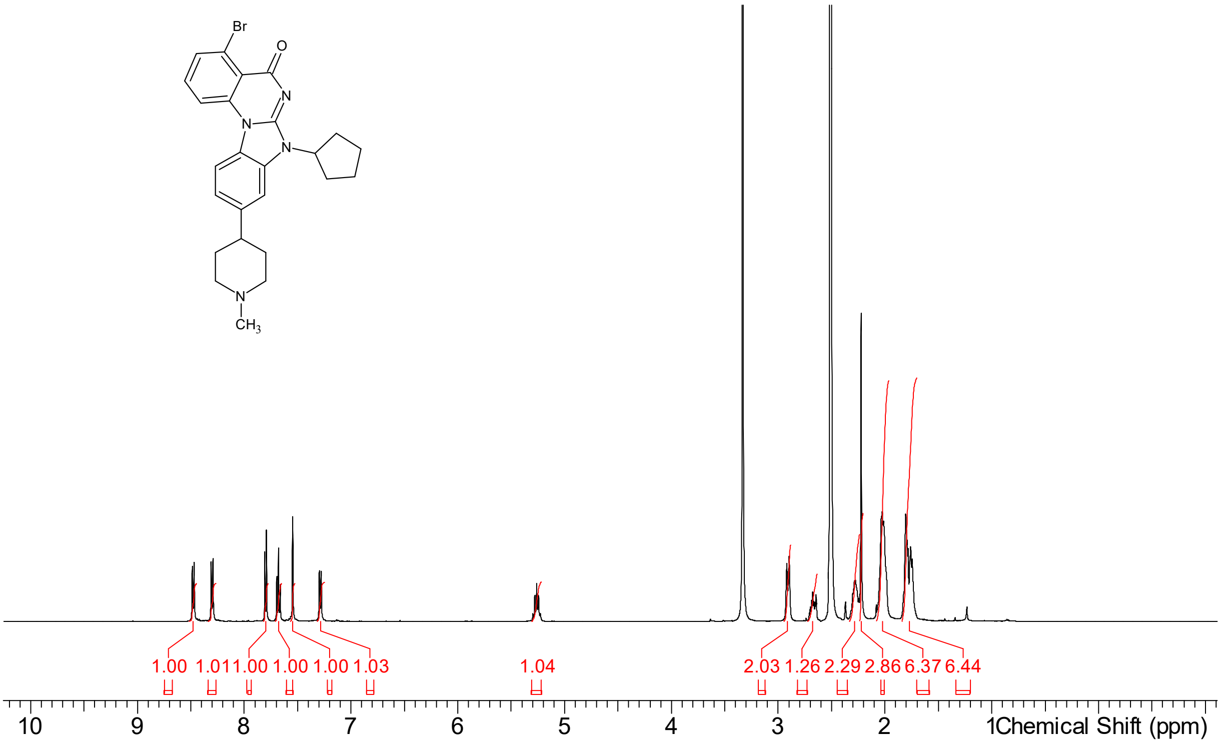

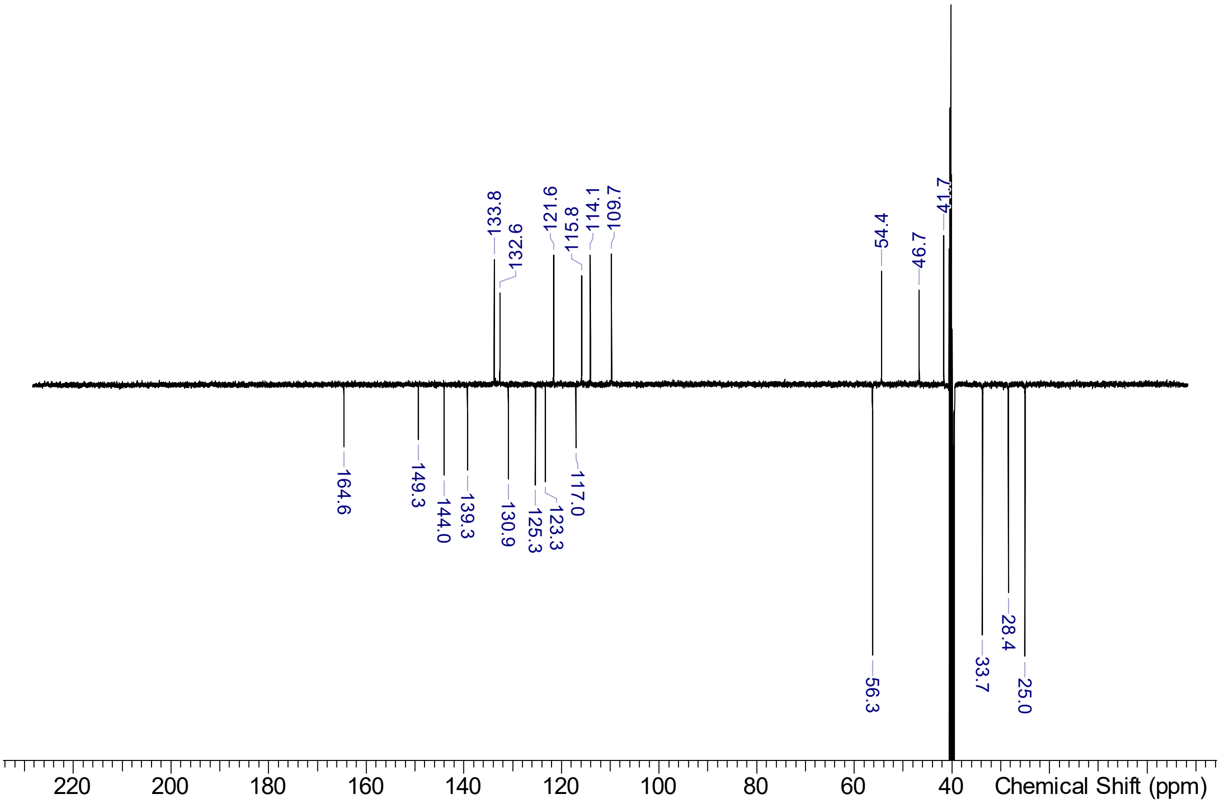

Supplementary Figure 35: ^{1}H and ^{13}C spectra of **4**

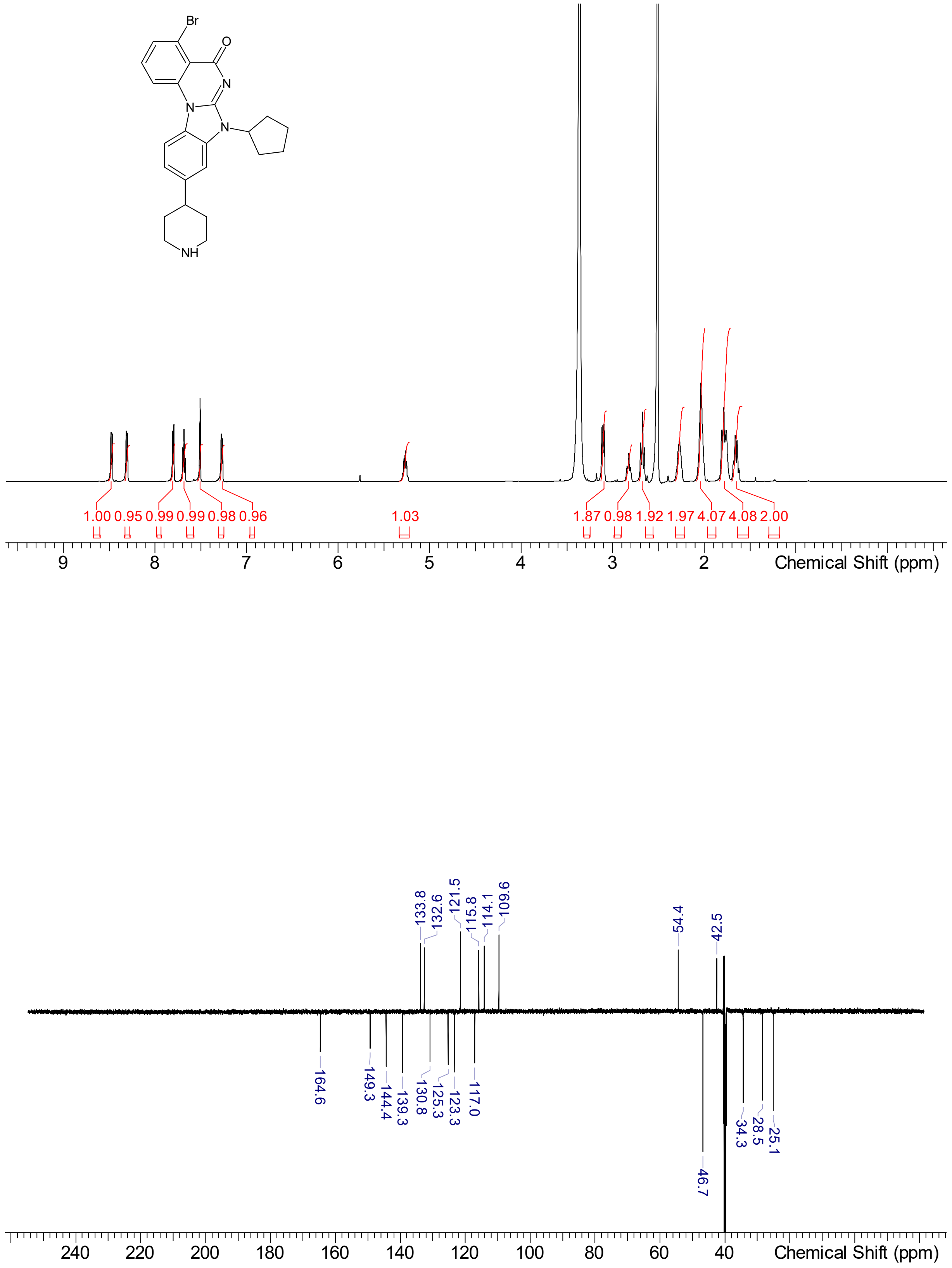

Supplementary Figure 36: ^{1}H and ^{13}C spectra of **5**

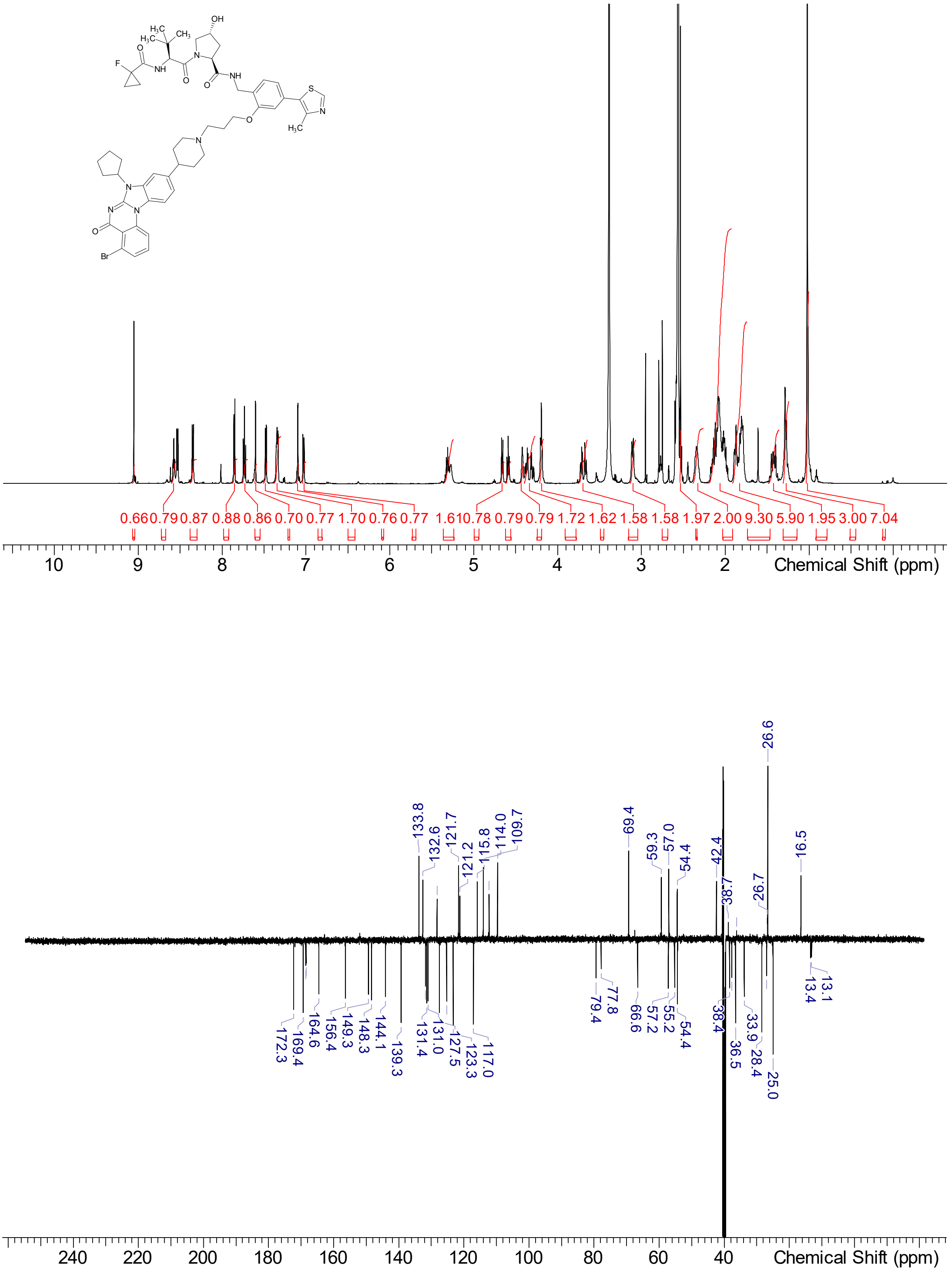

Supplementary Figure 37: ^{1}H and ^{13}C spectra of **6**

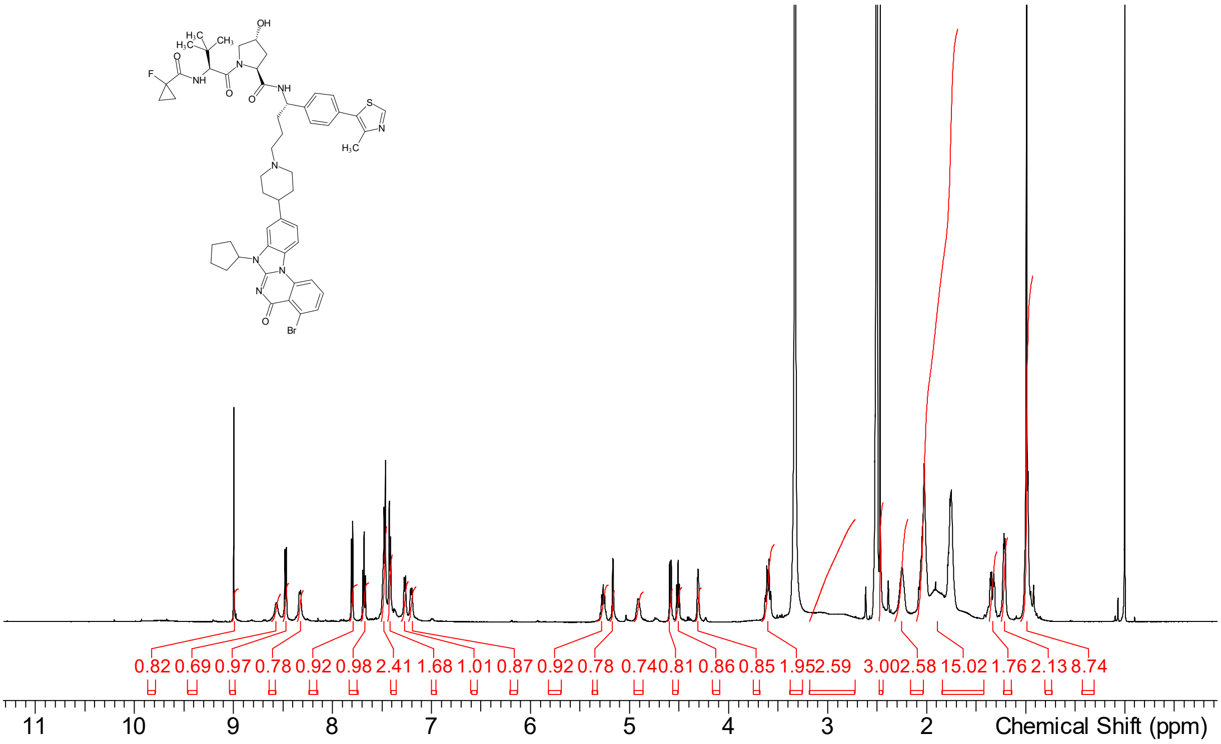

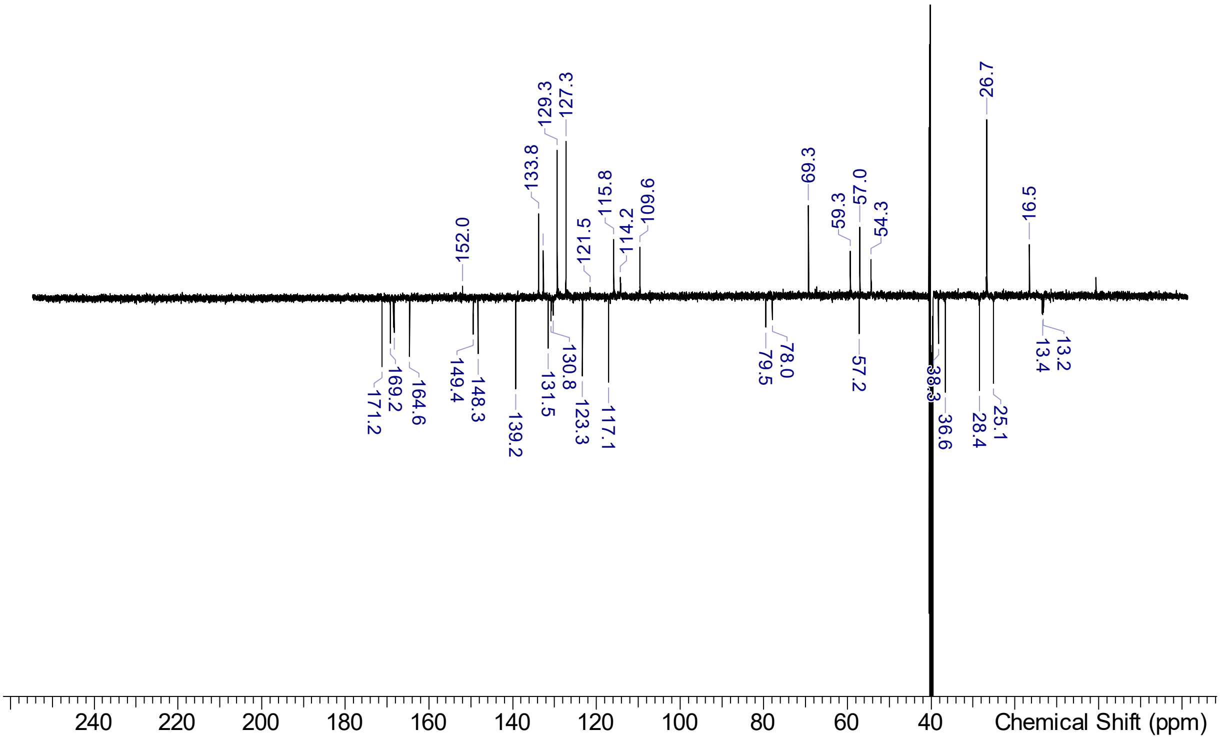

Supplementary Figure 38: ^{1}H and ^{13}C spectra of **7**

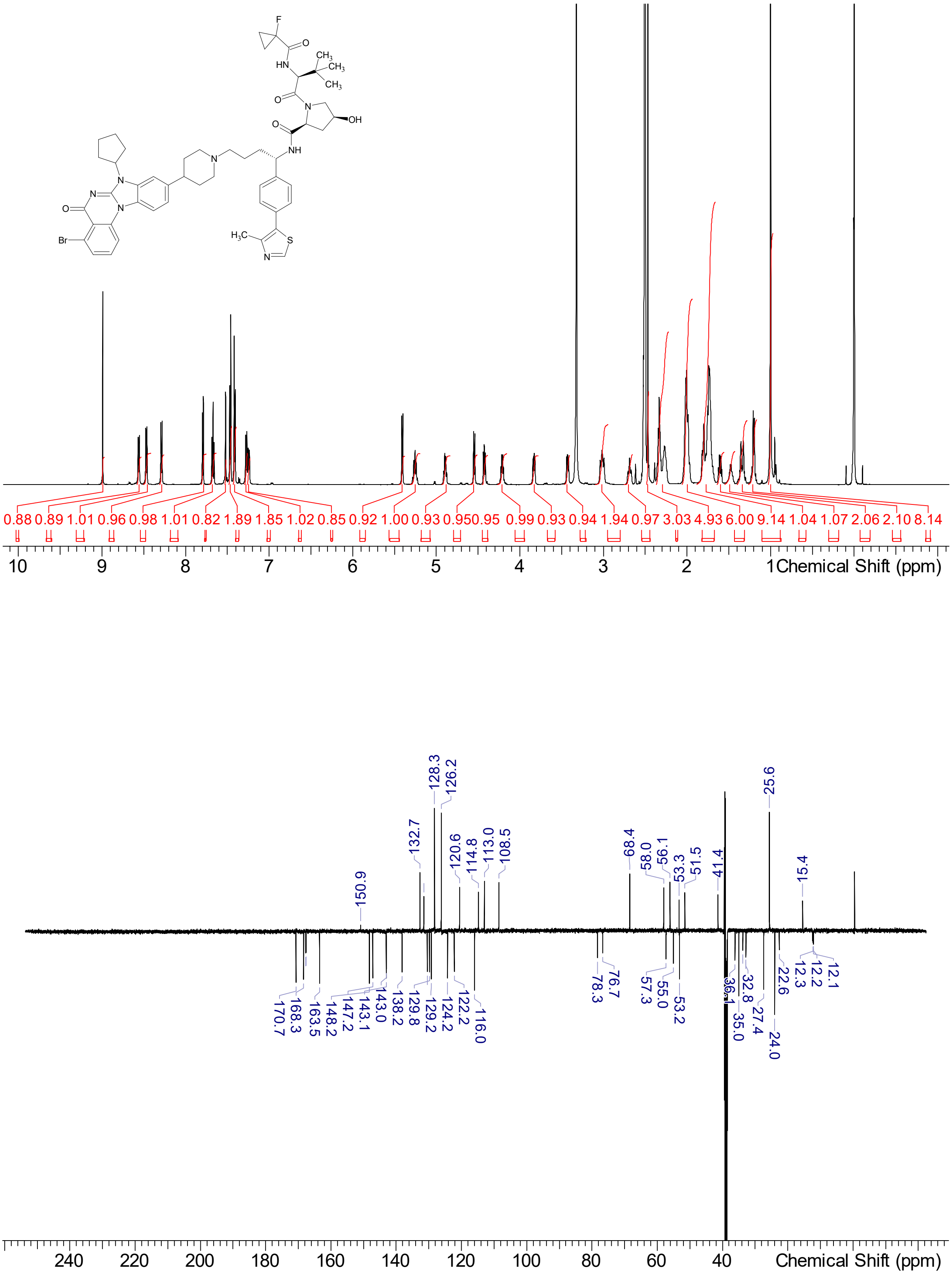

Supplementary Figure 39: ^{1}H and ^{13}C spectra of **8**

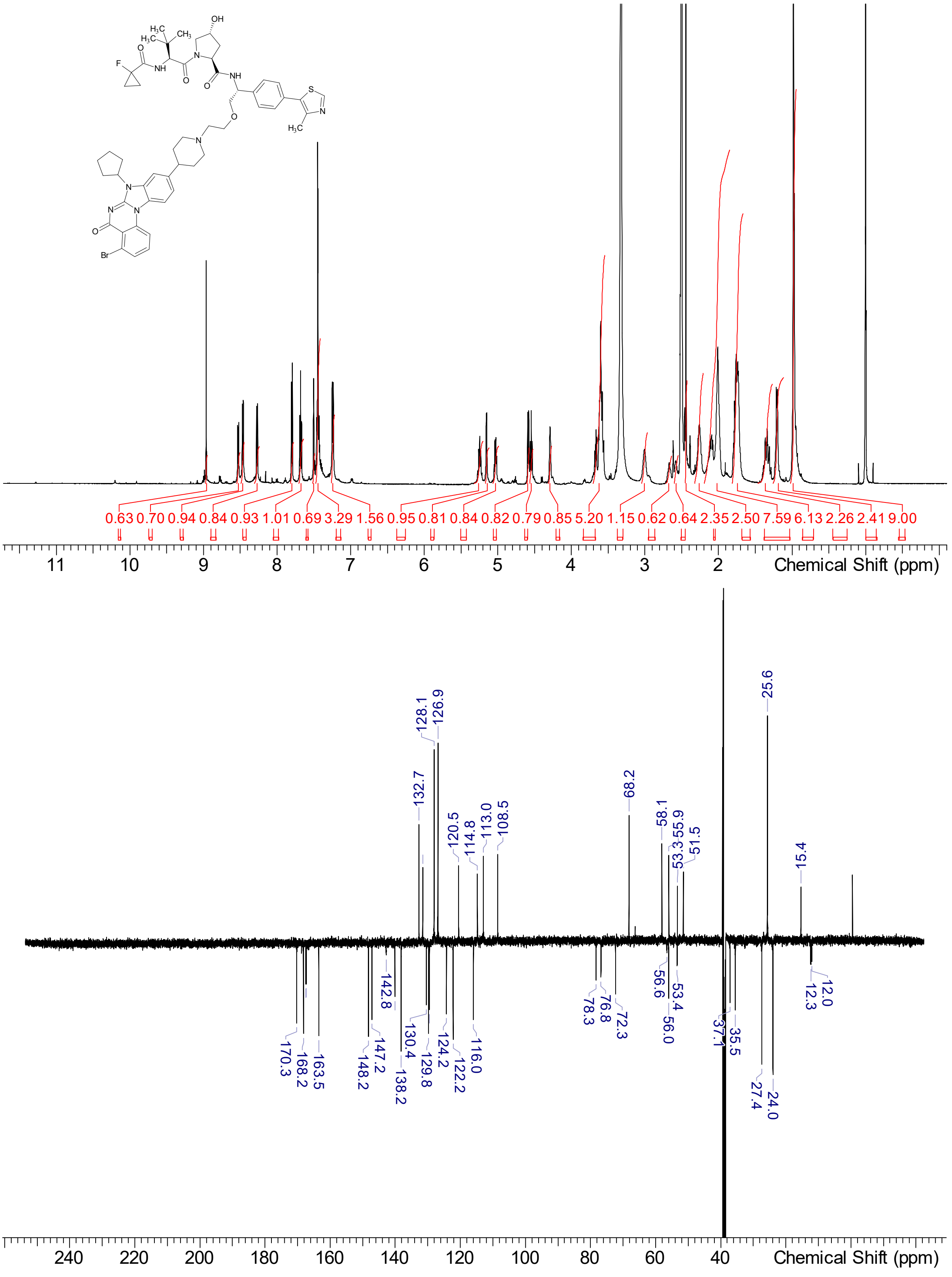

Supplementary Figure 40: ^{1}H and ^{13}C spectra of **9**

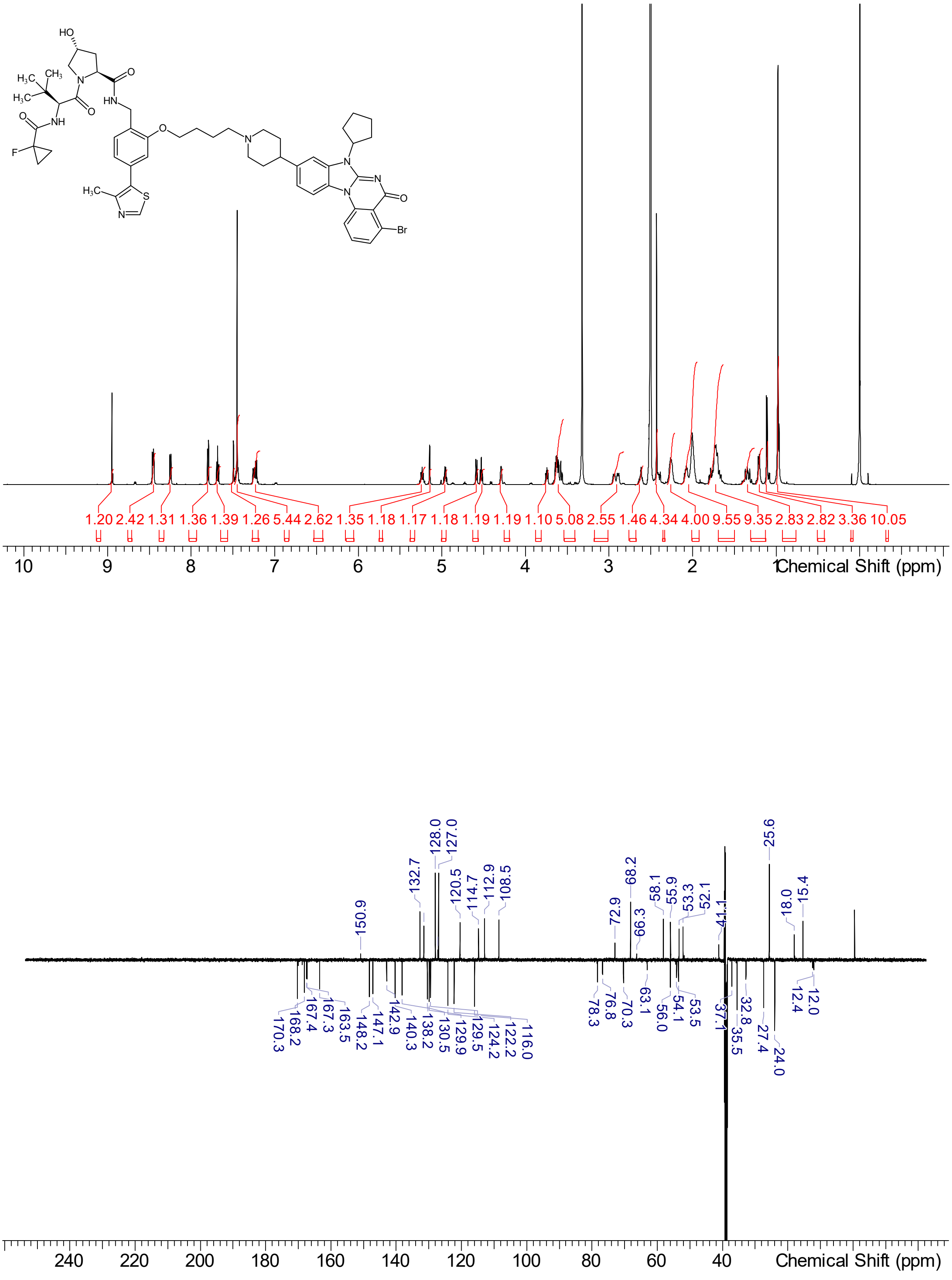

Supplementary Figure 41: ^{1}H and ^{13}C spectra of **10**

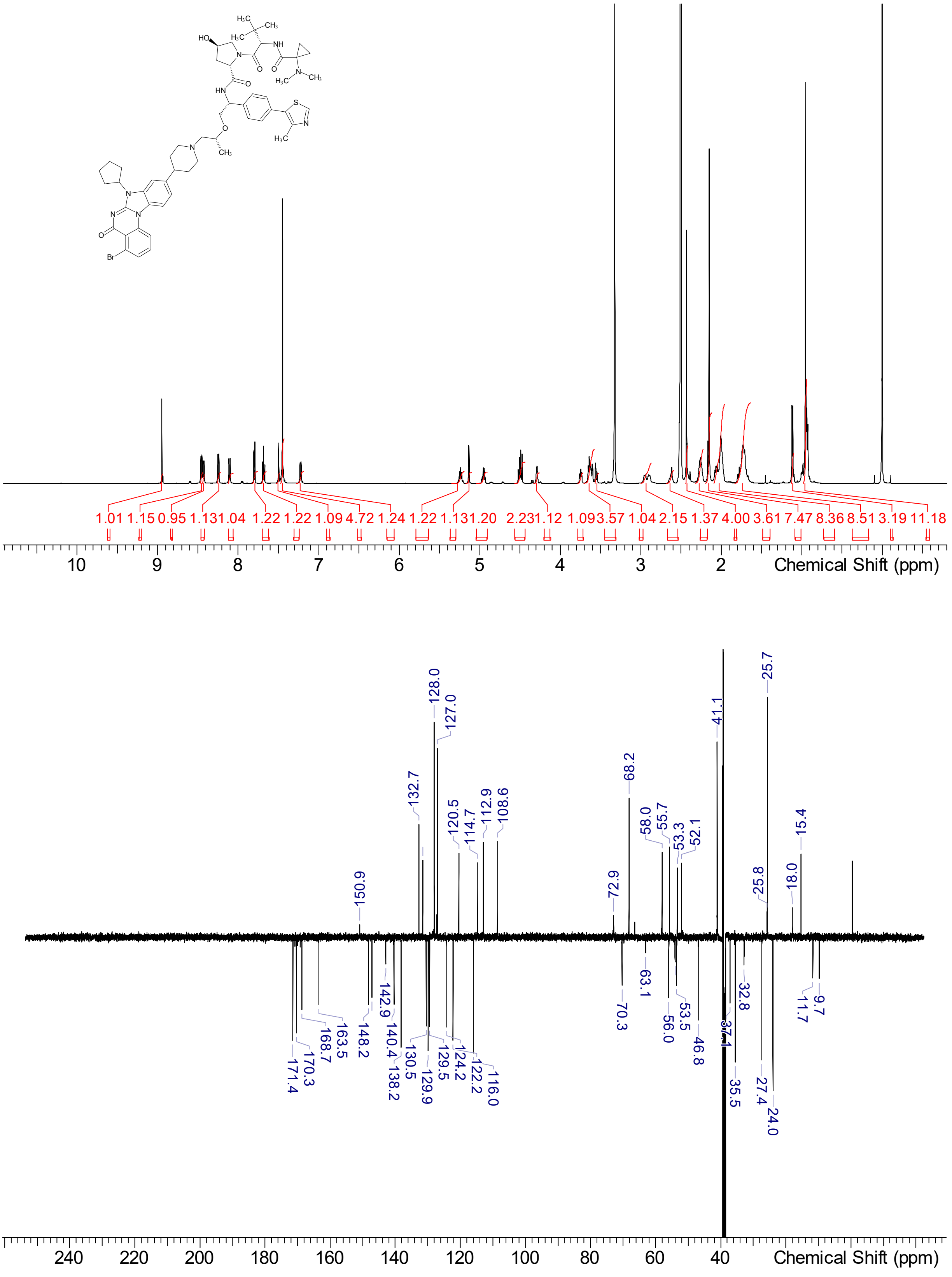

Supplementary Figure 42: ^{1}H and ^{13}C spectra of **11**

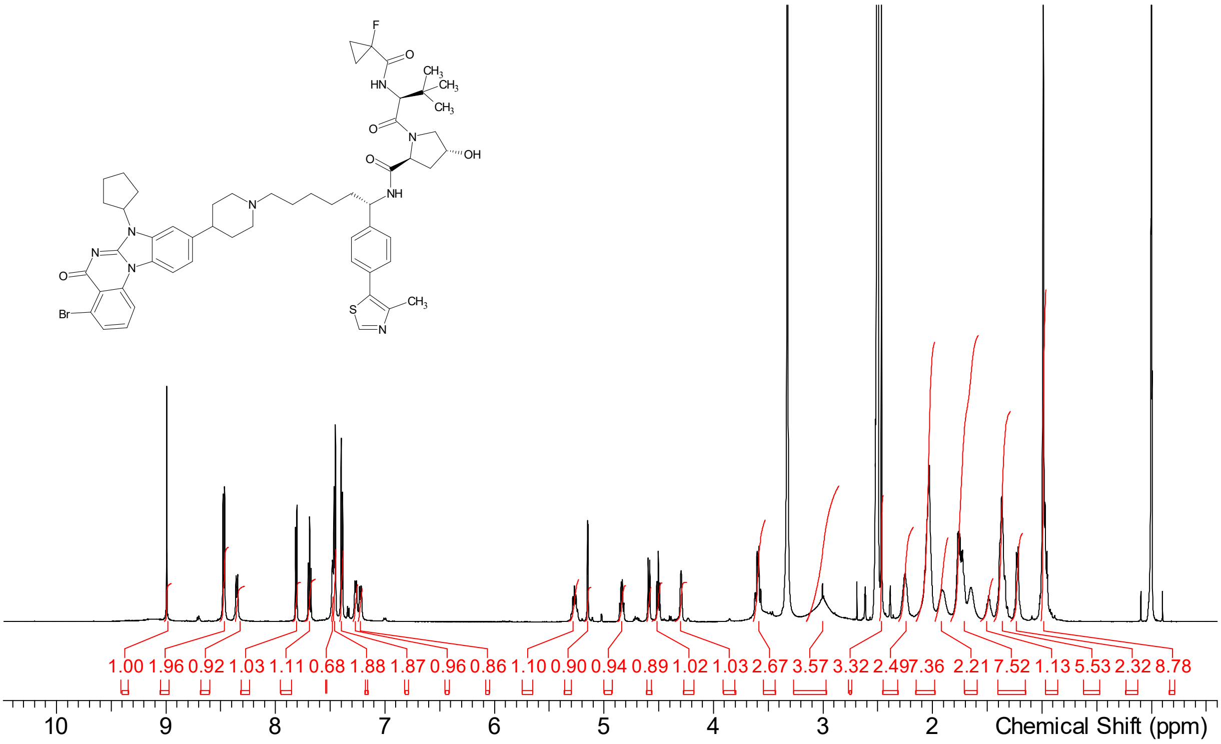

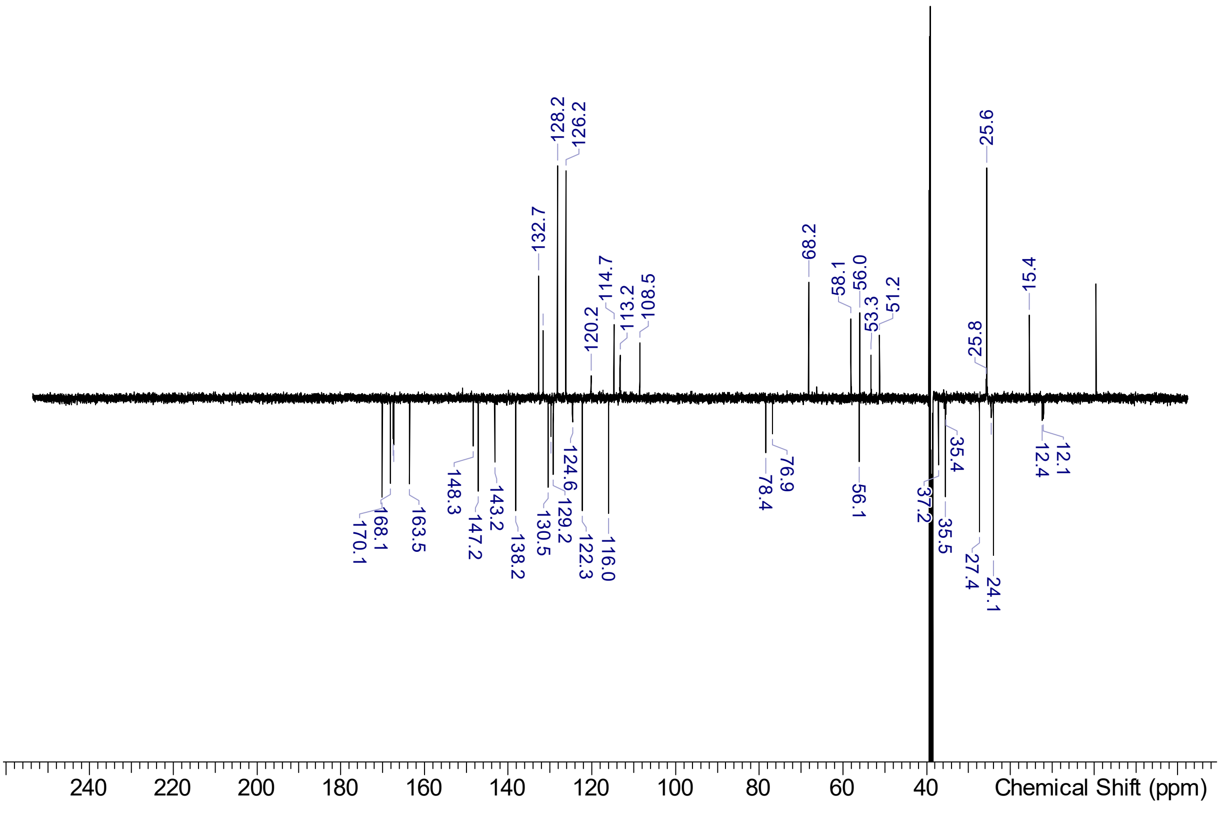

Supplementary Figure 43: ^{1}H and ^{13}C spectra of **ACBI2**

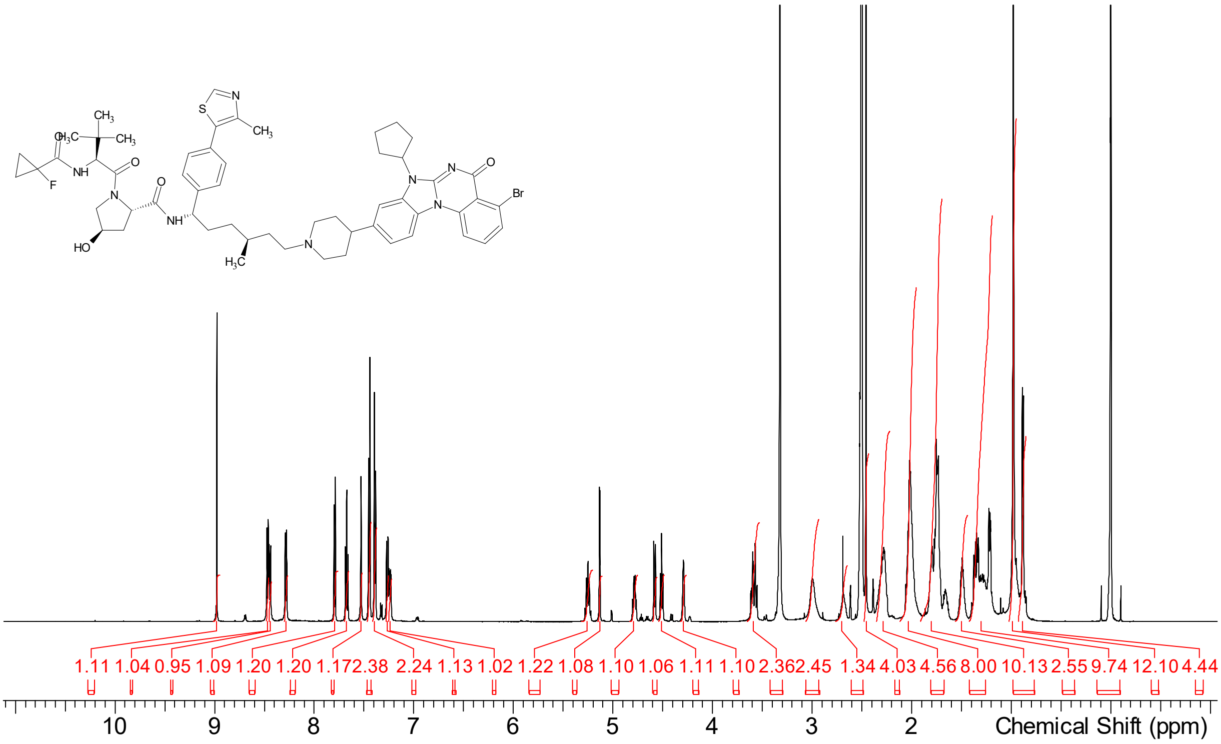

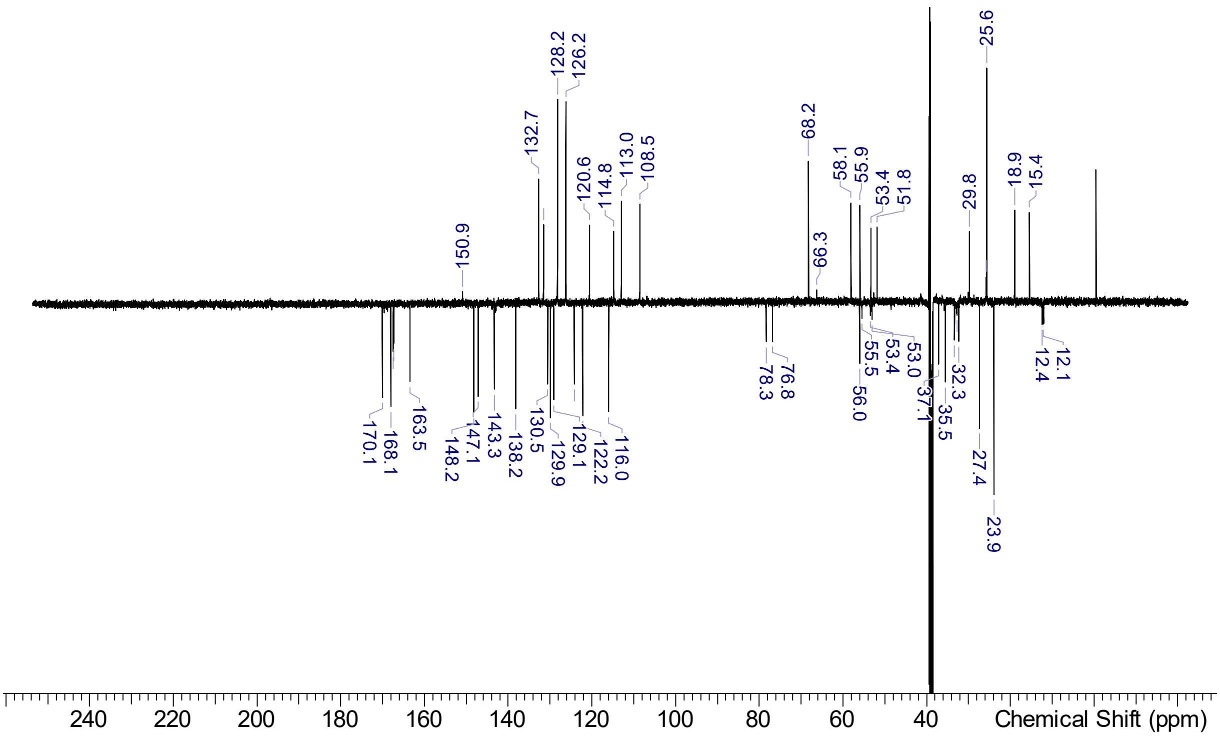

Supplementary Figure 44: ^{1}H and ^{13}C spectra of **12**

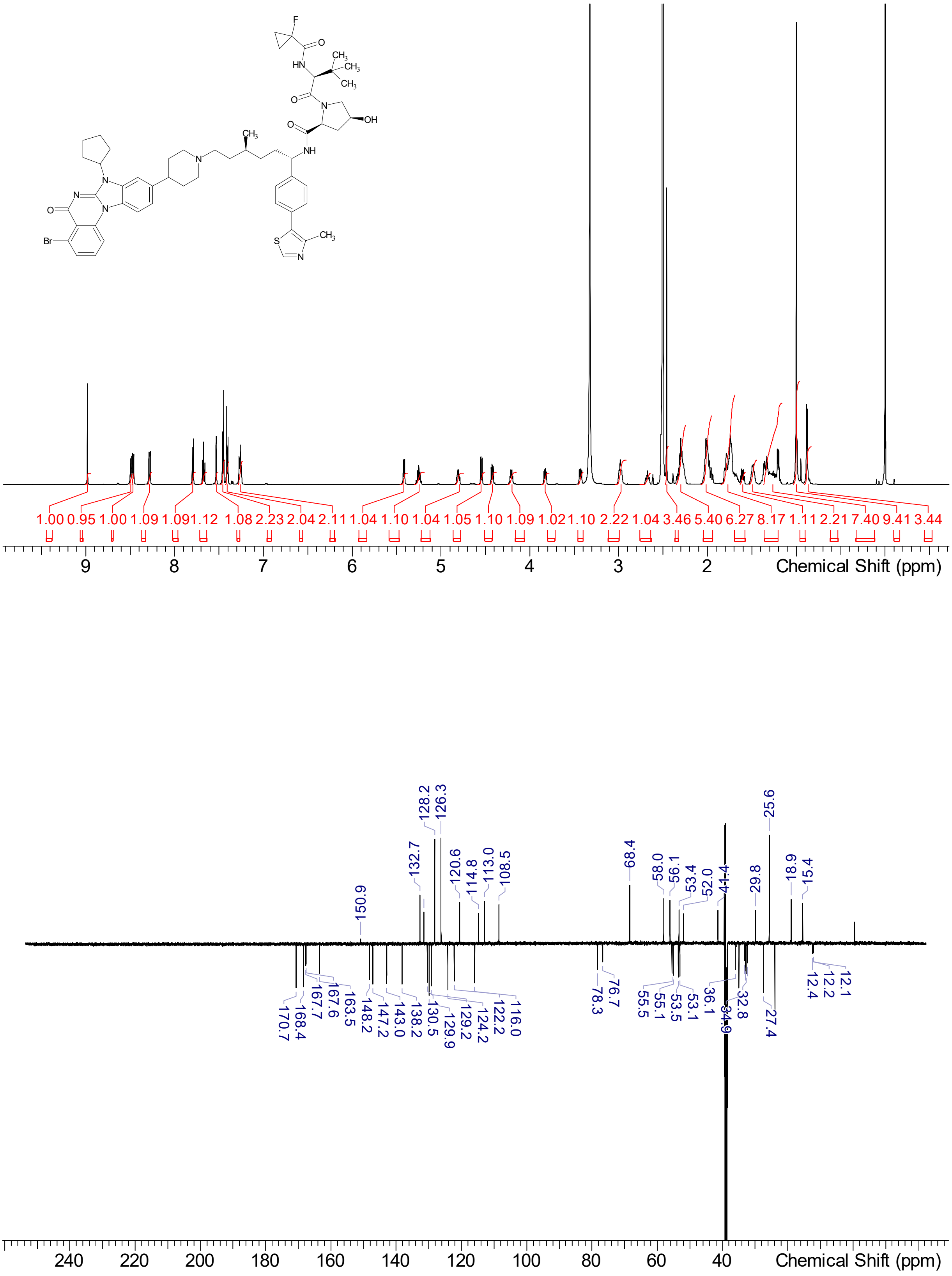

Supplementary Figure 45: ^{1}H and ^{13}C spectra of **13**

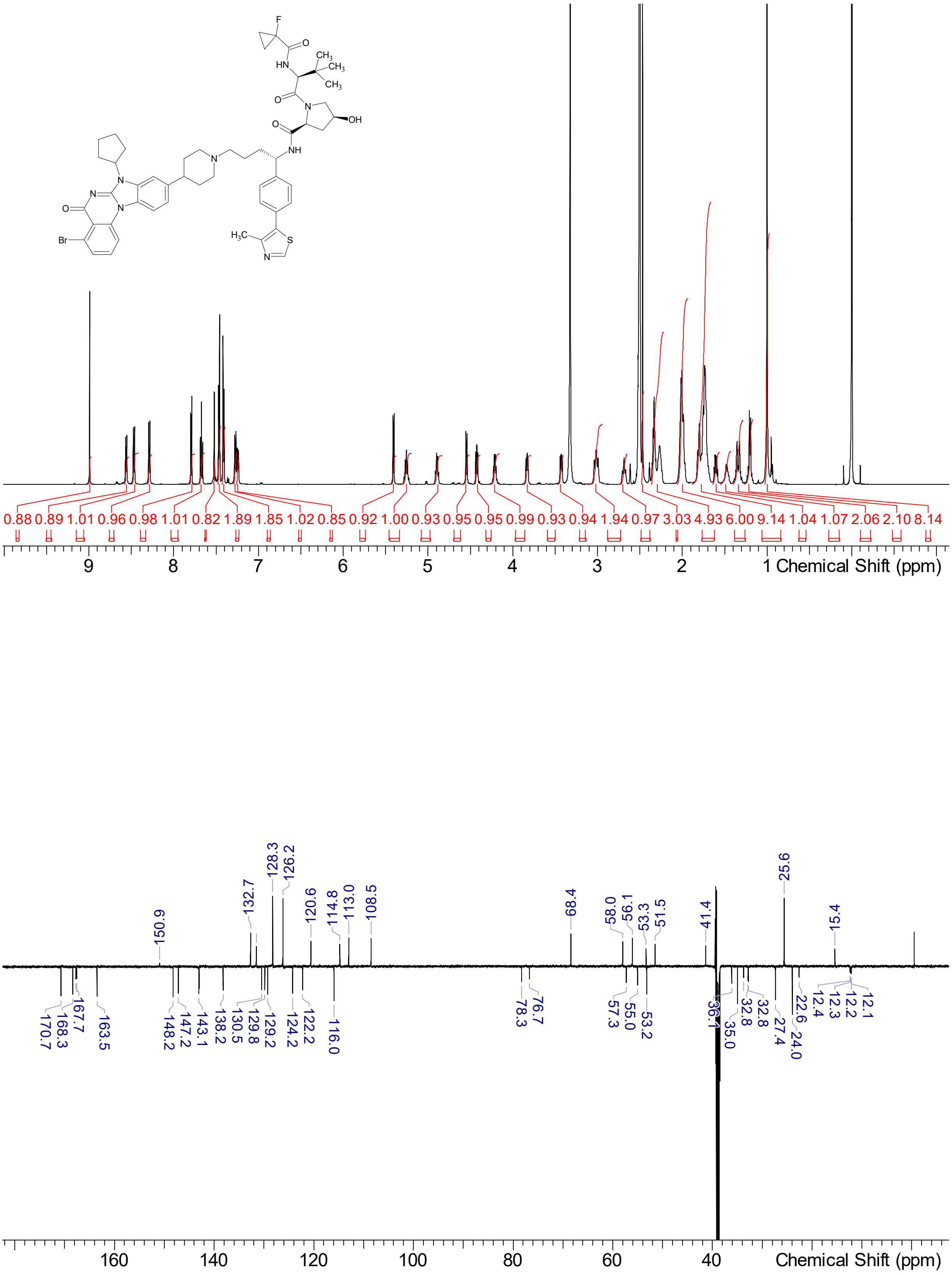

Supplementary Figure 46: ^{1}H and ^{13}C spectra of **14**

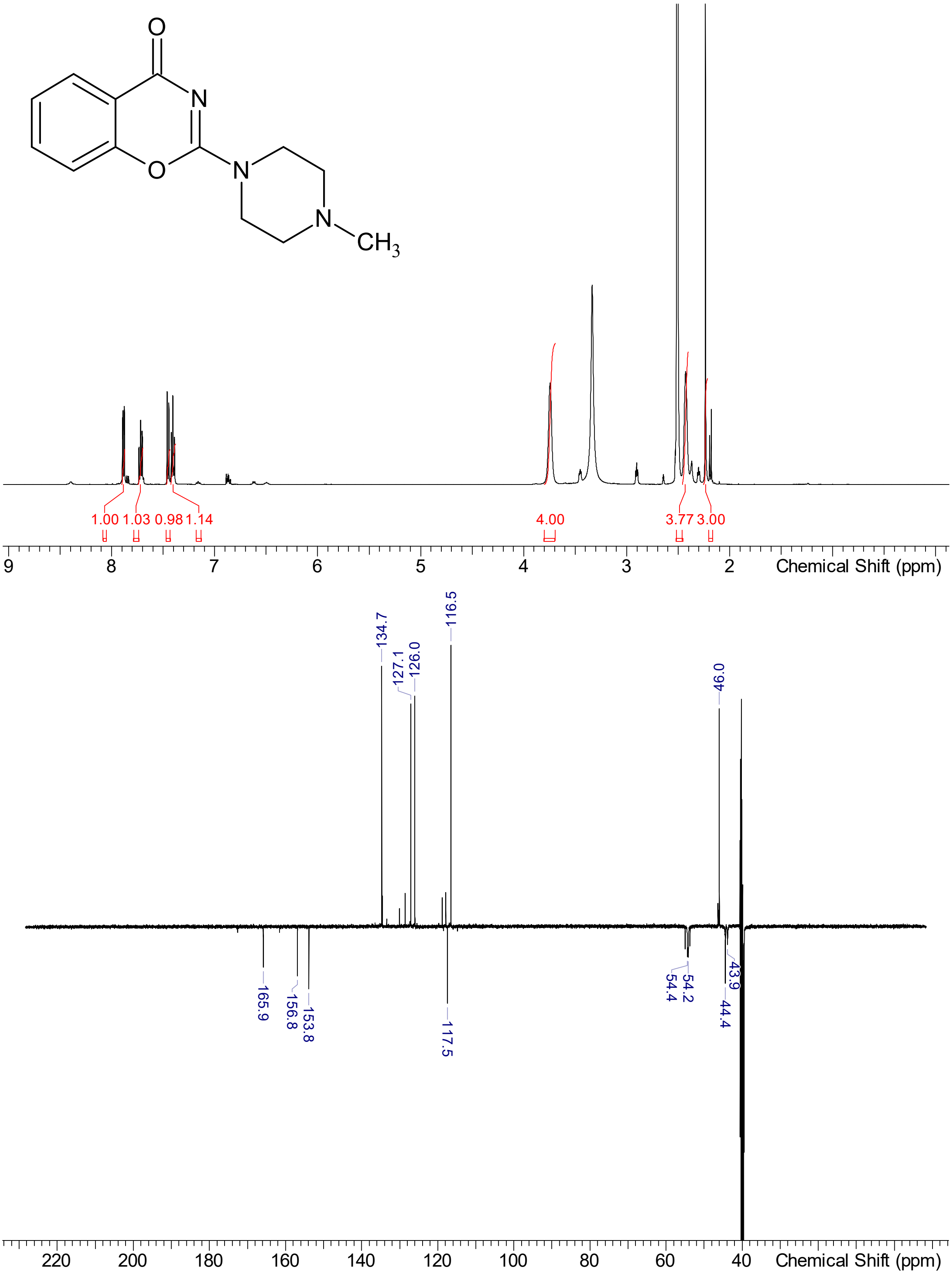

Supplementary Figure 47: ^{1}H and ^{13}C spectra of **15**

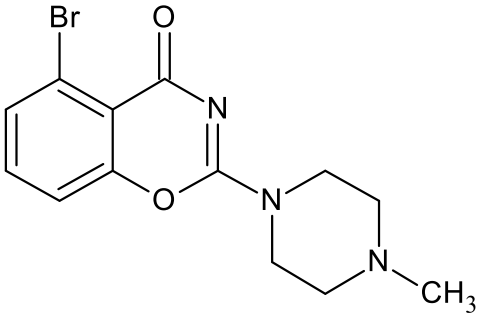

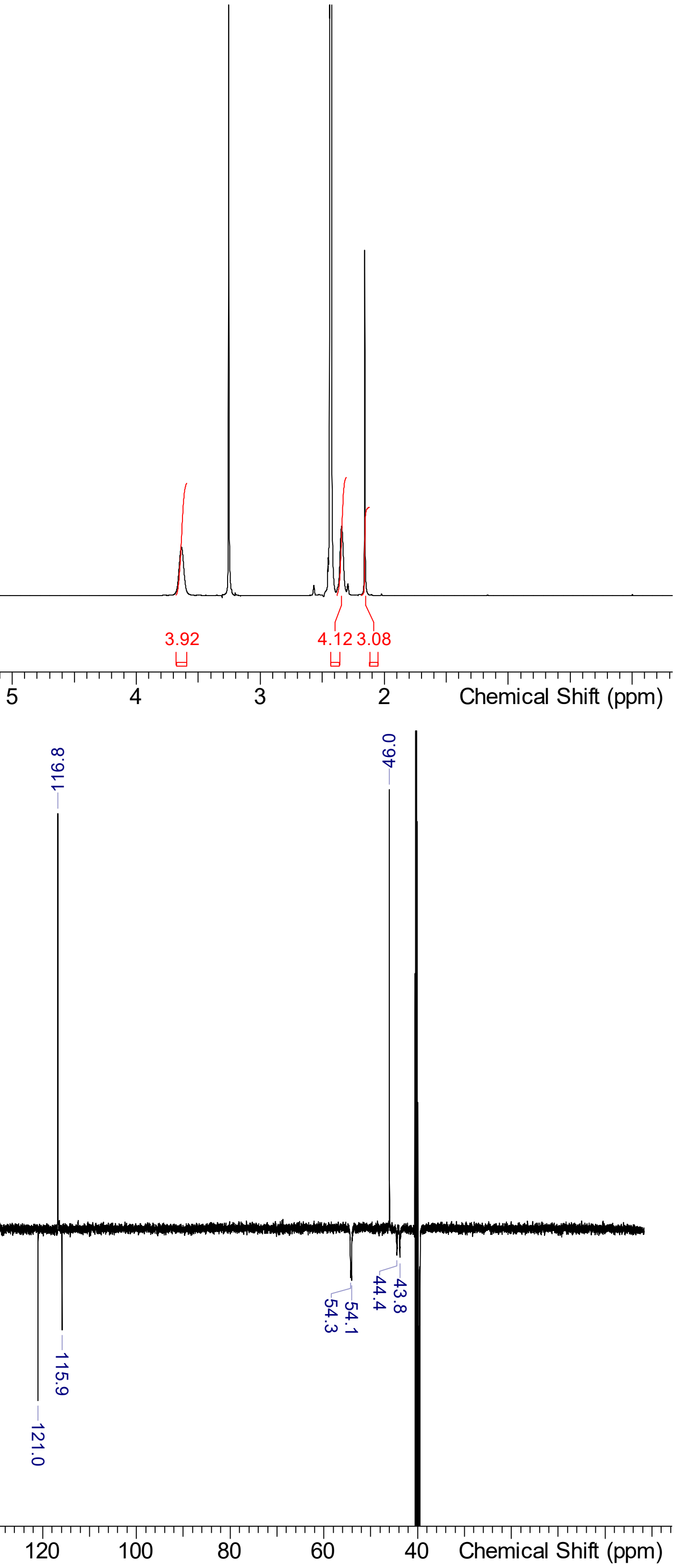

Supplementary Figure 48: ^{1}H and ^{13}C spectra of **16**

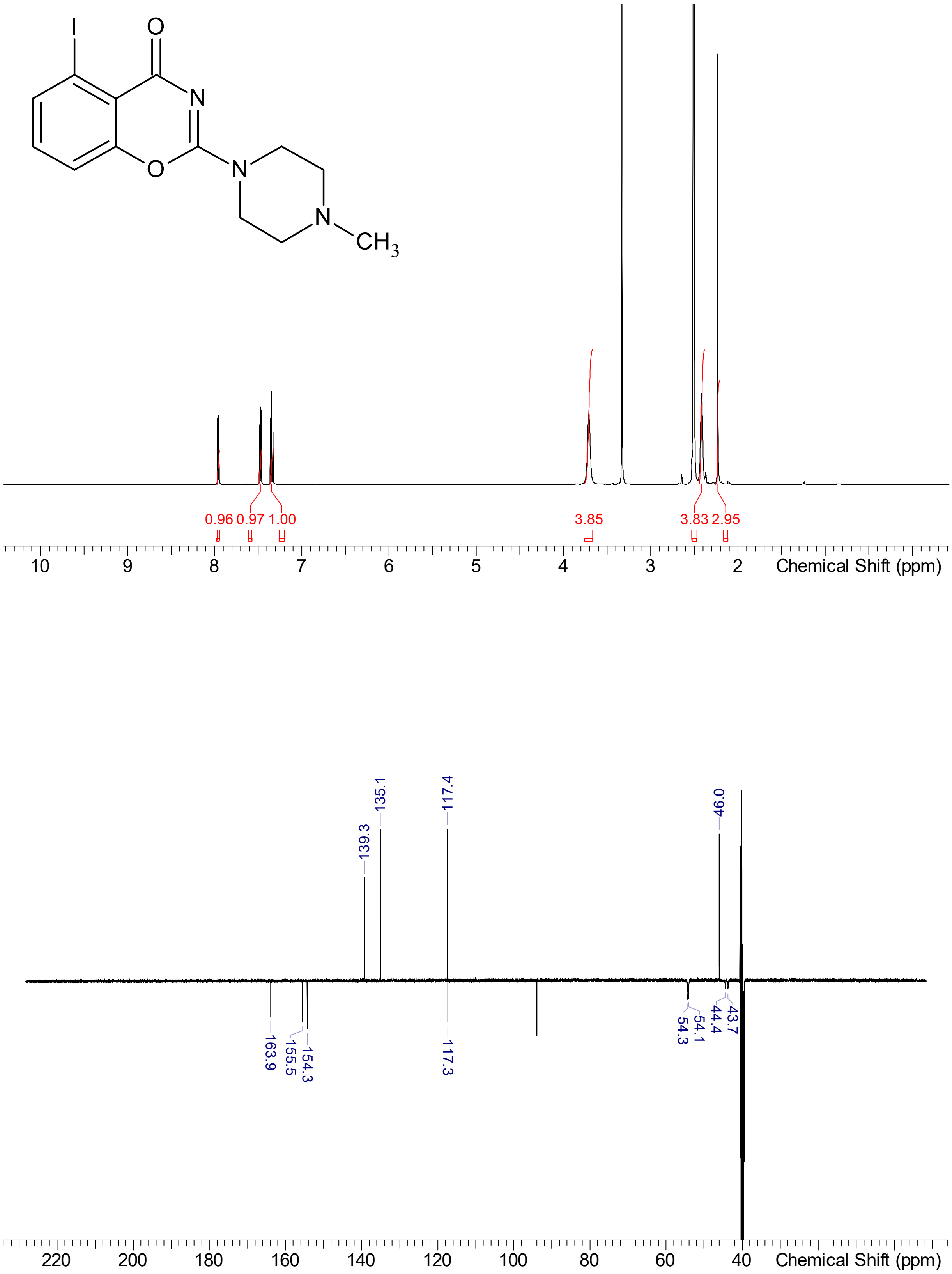

Supplementary Figure 49: ^{1}H and ^{13}C spectra of **17**

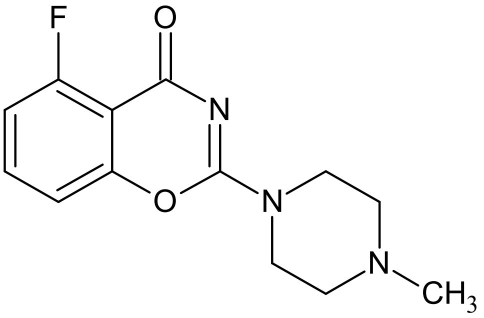

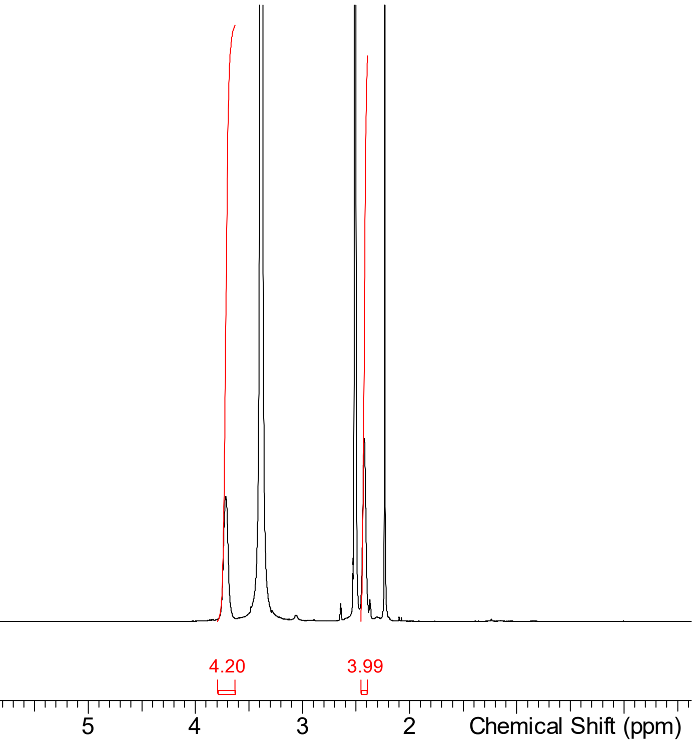

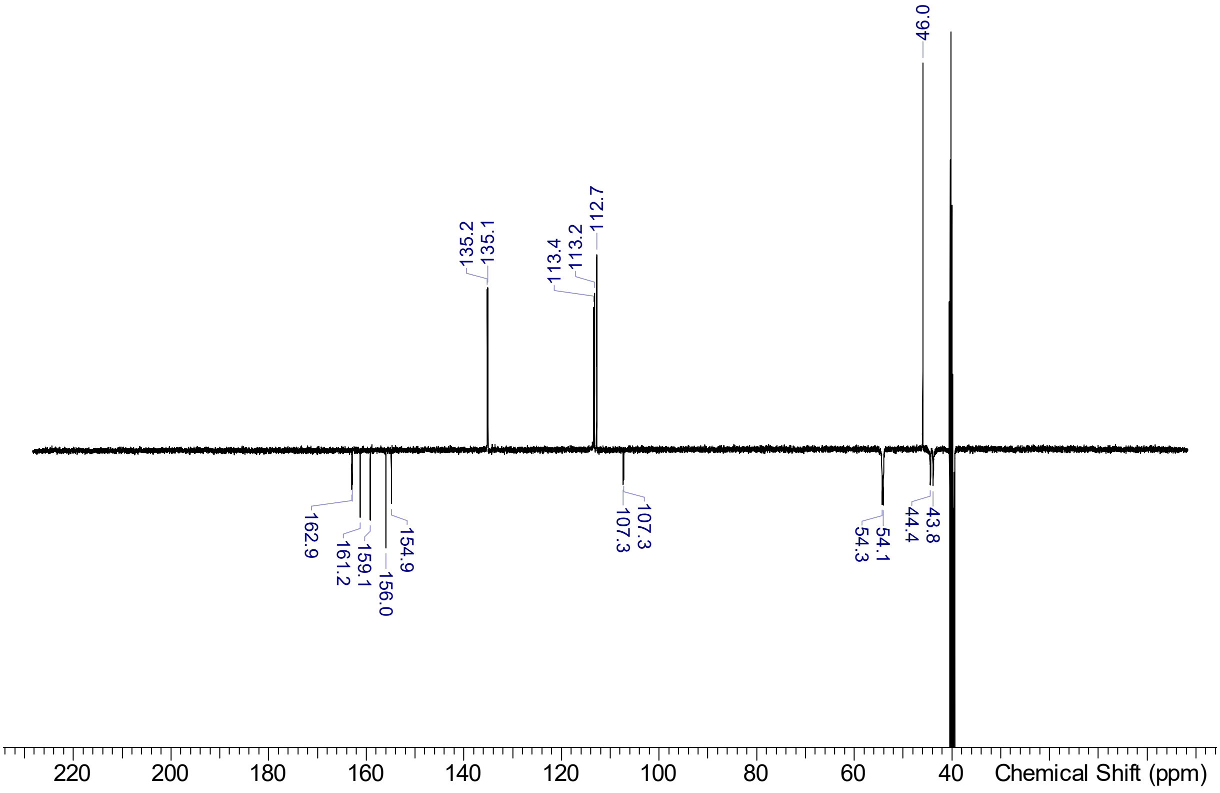

Supplementary Figure 50: ^{1}H and ^{13}C spectra of **18**

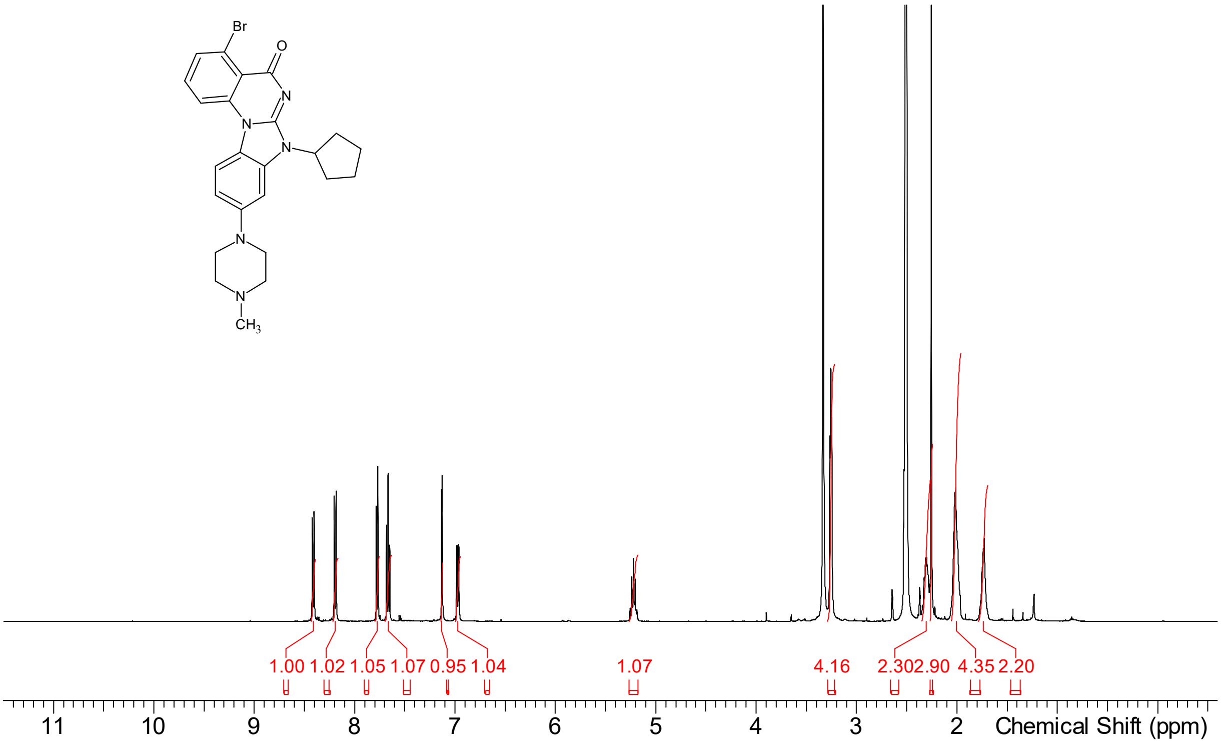

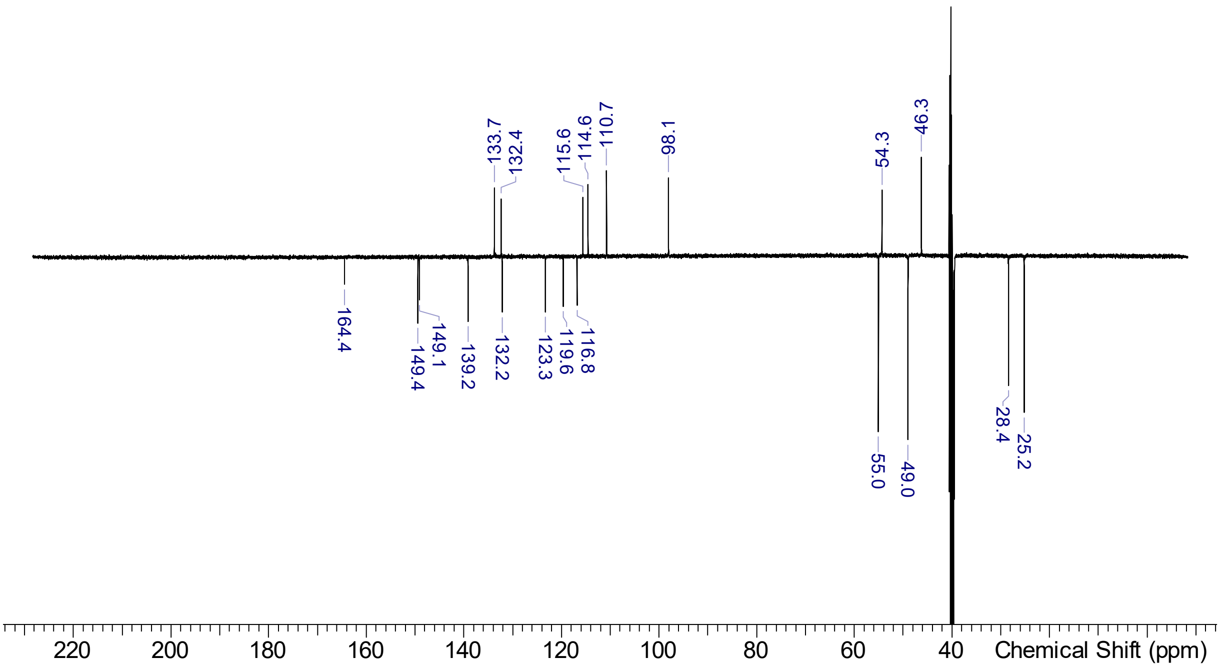

Supplementary Figure 51: ^{1}H and ^{13}C spectra of **19**

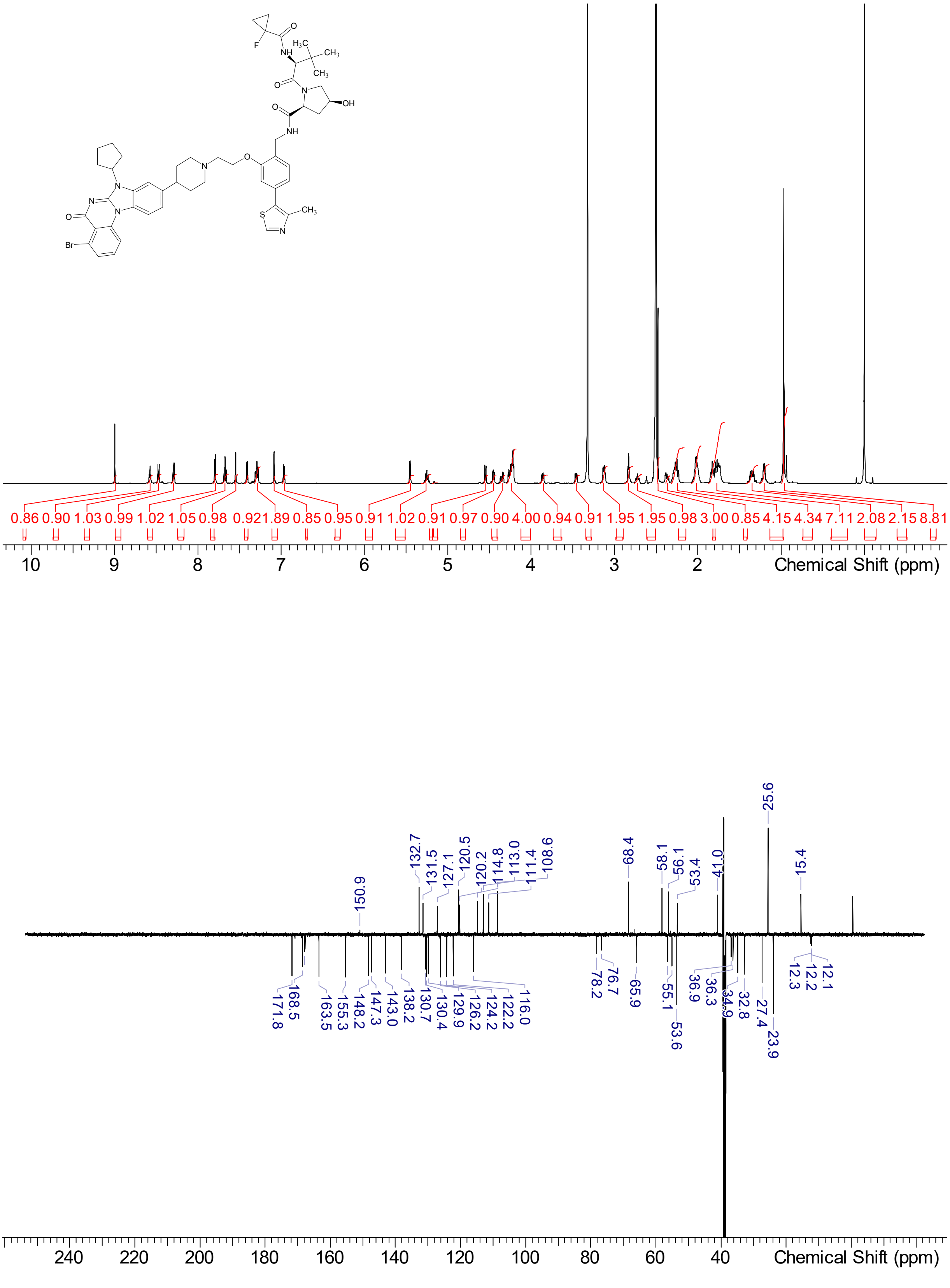

Supplementary Figure 52: ^{1}H and ^{13}C spectra of **20**

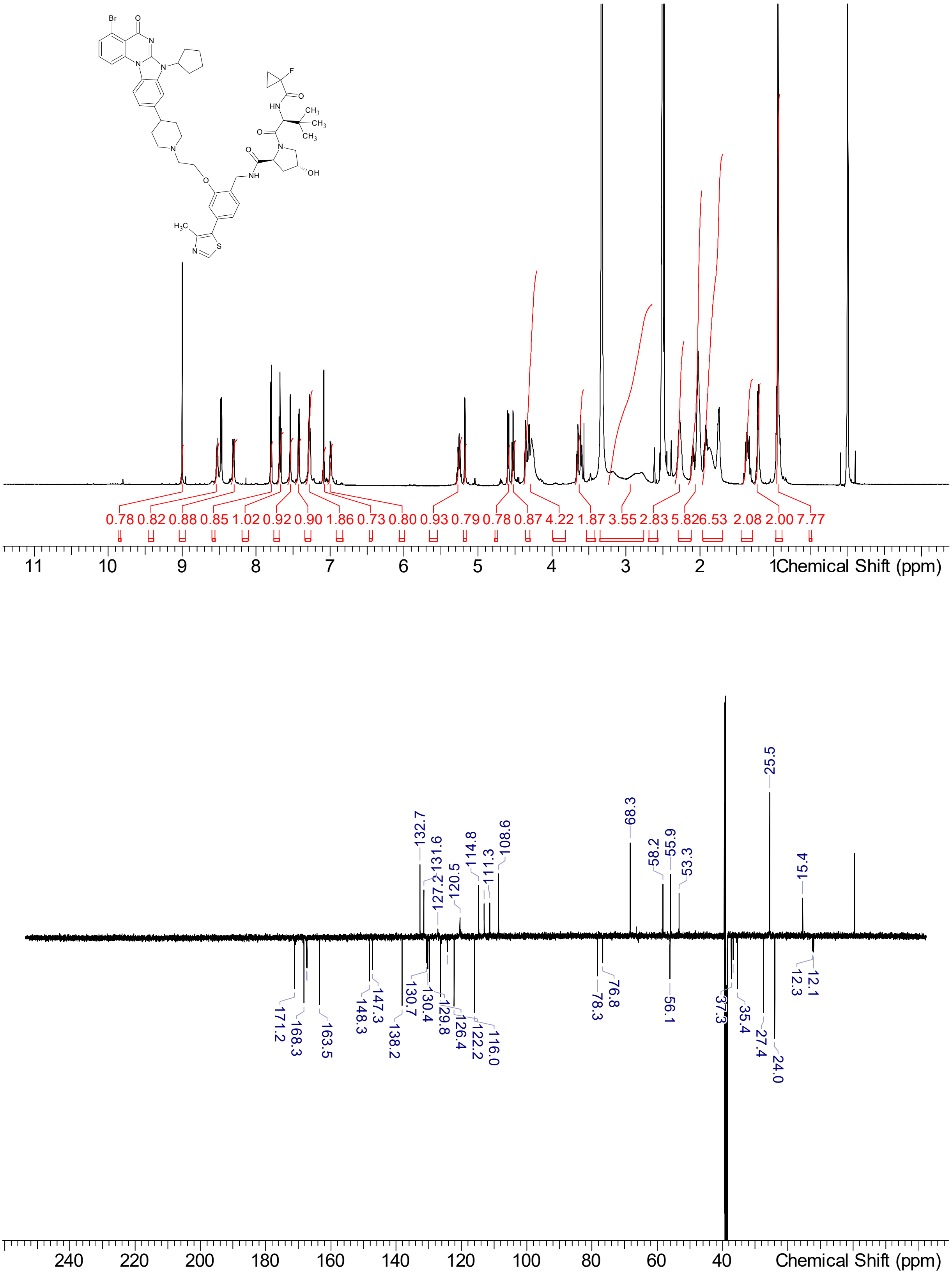

Supplementary Figure 53: ^{1}H spectrum of **21**

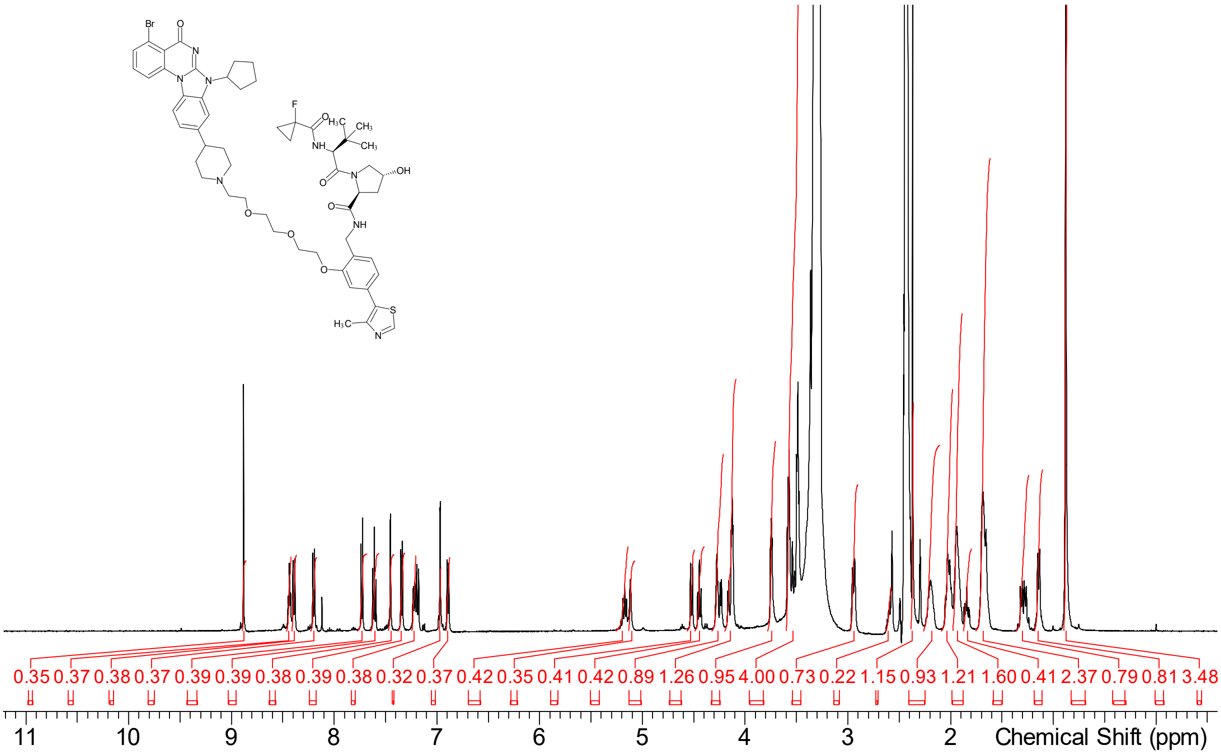

Supplementary Figure 54: ^{1}H and ^{13}C spectra of **22**

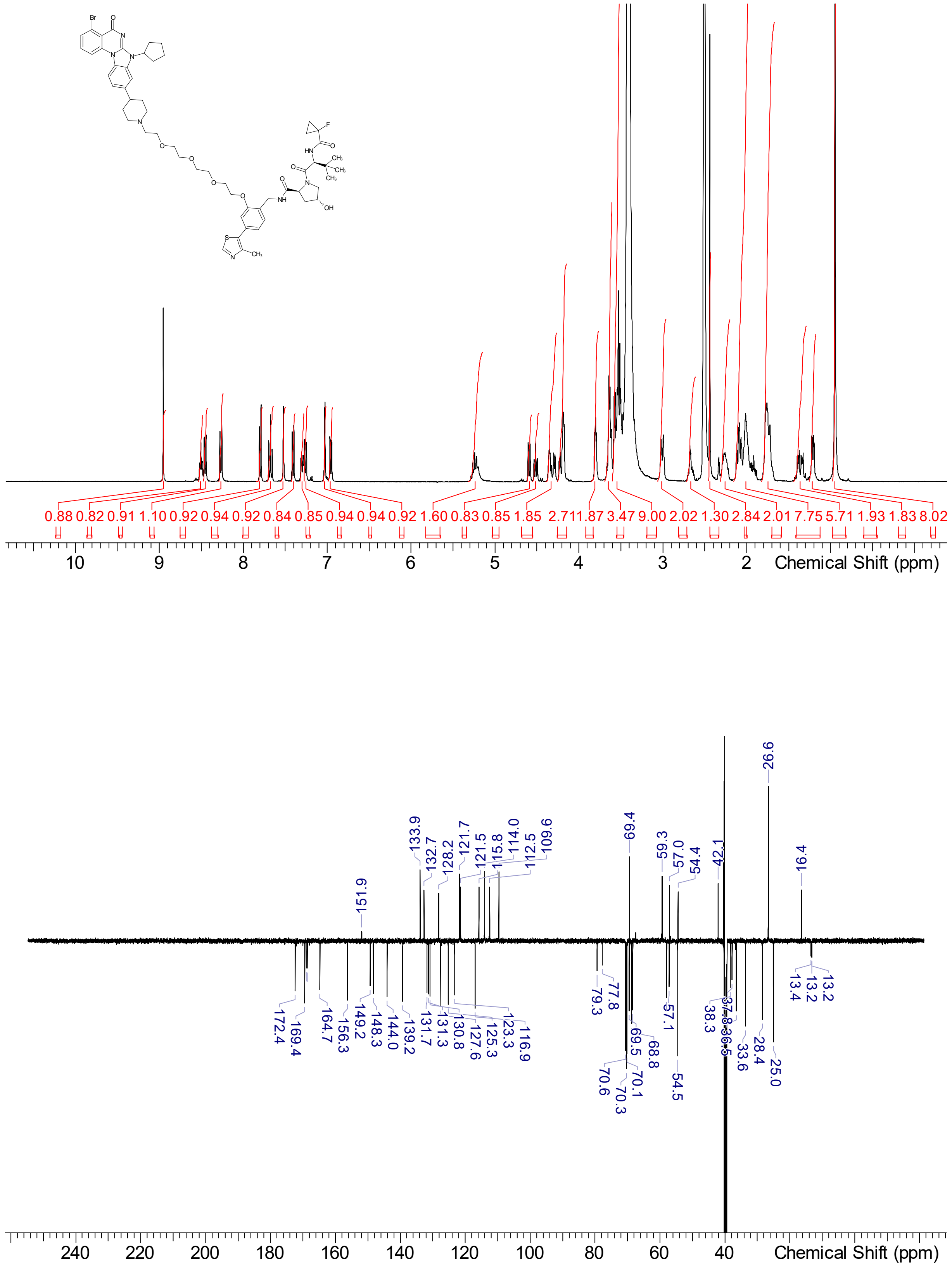

Supplementary Figure 55: ^{1}H and ^{13}C spectra of **23**

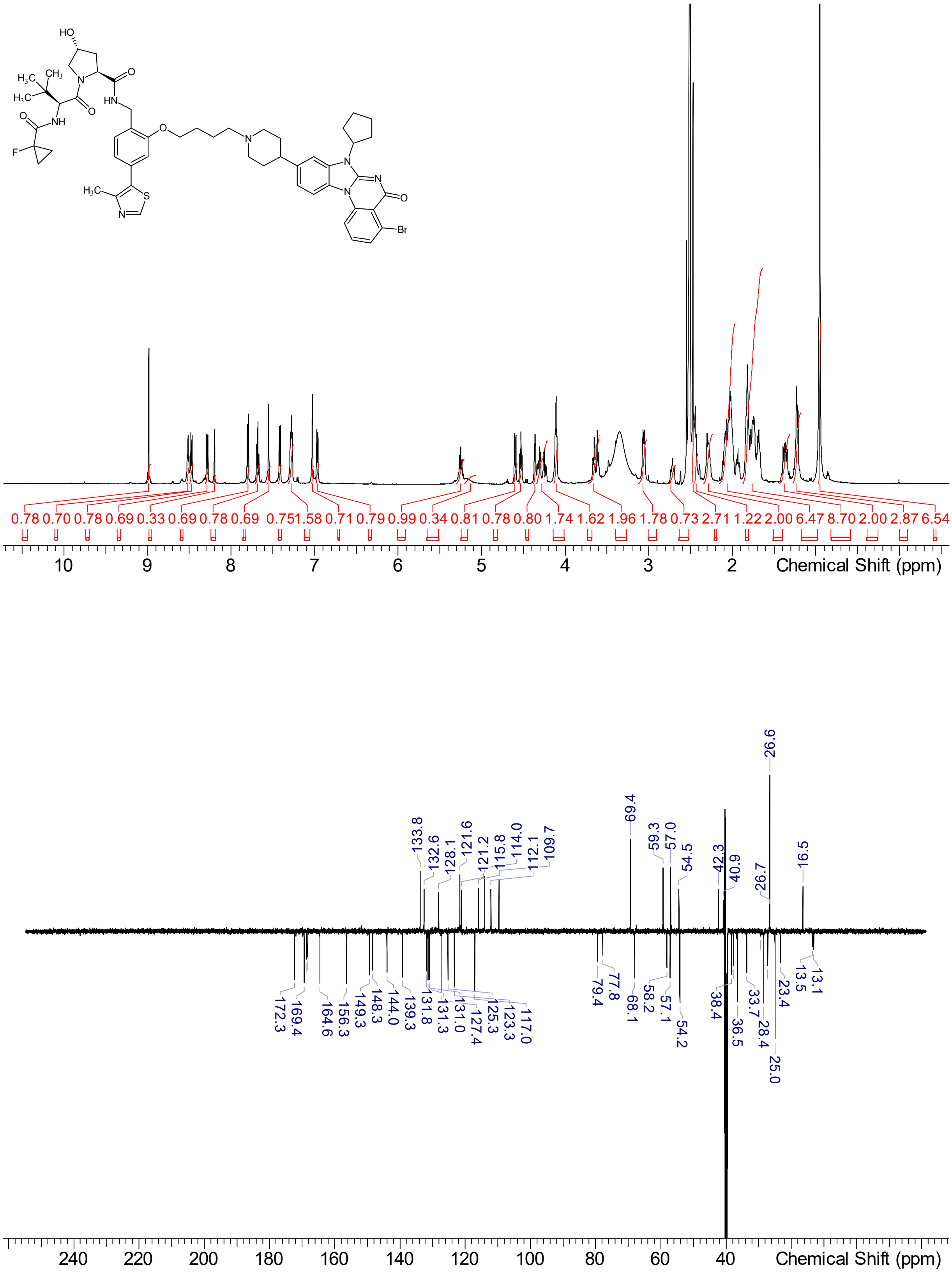

Supplementary Figure 56: ^{1}H spectrum of **24**

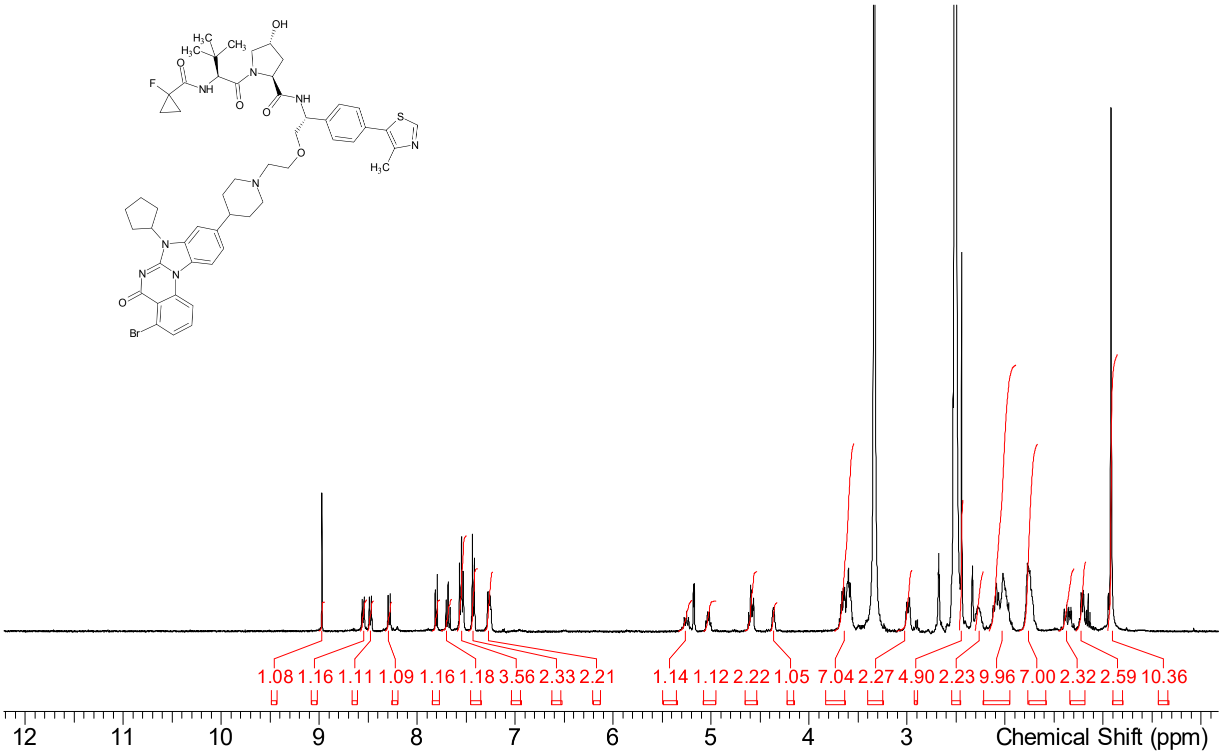

Supplementary Figure 57: ^{1}H and ^{13}C spectra of **25**

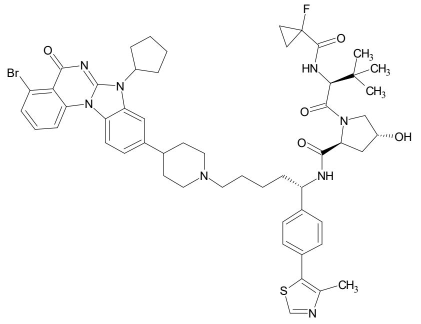

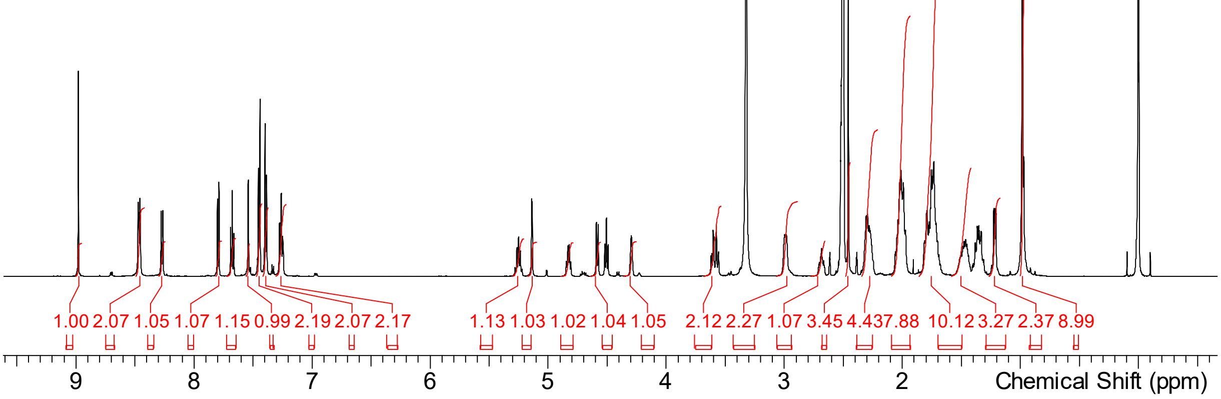

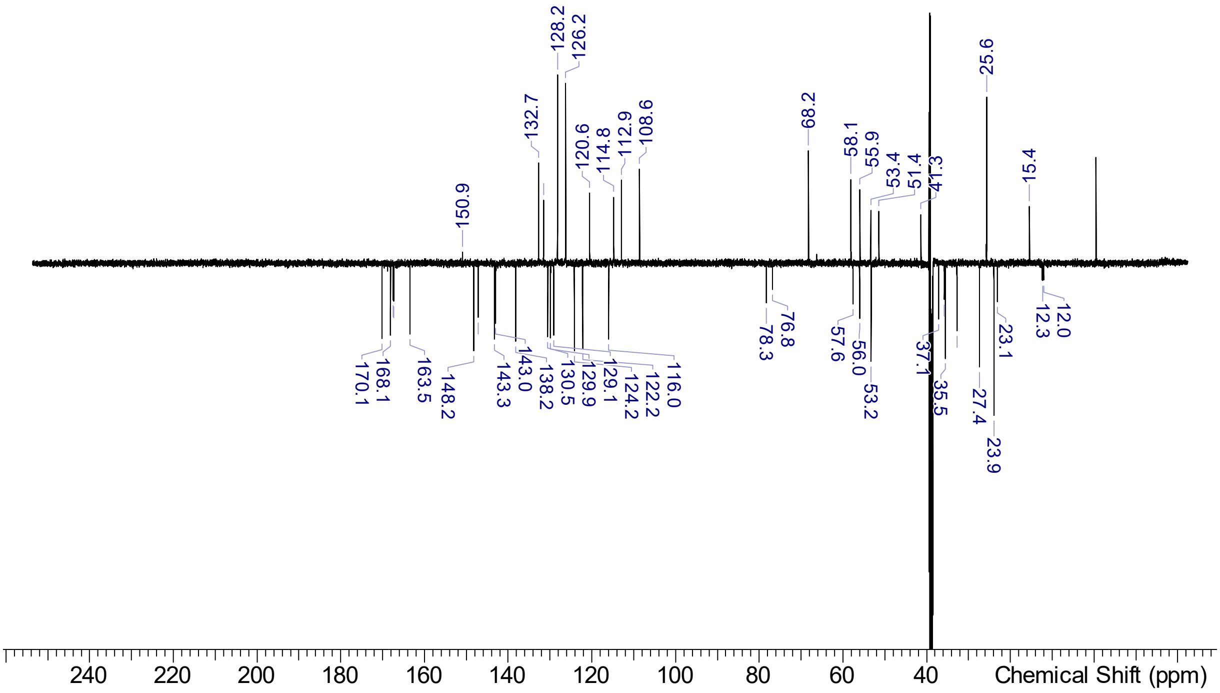

Supplementary Figure 58: ^{1}H and ^{13}C spectra of **26**

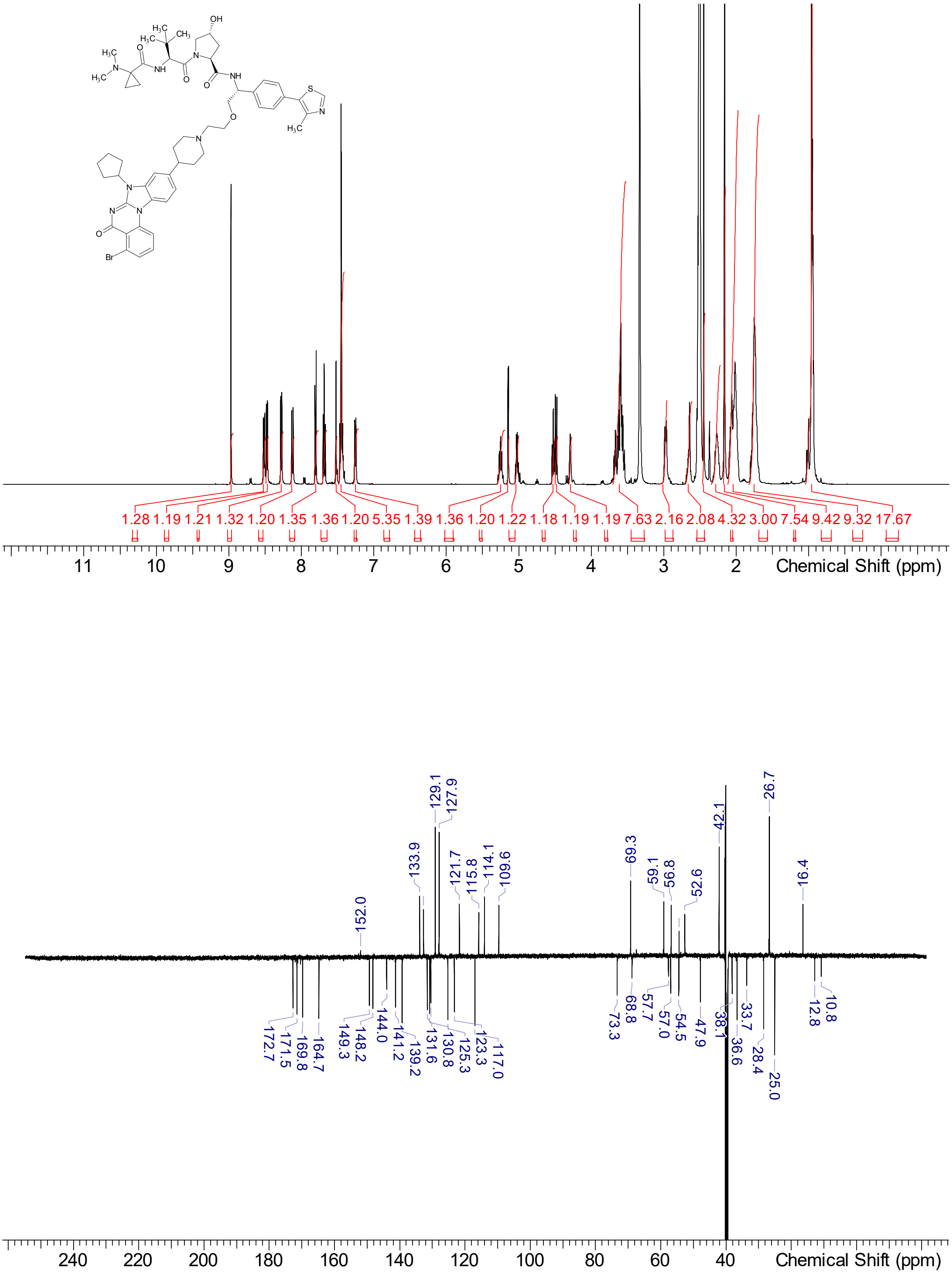

Supplementary Figure 59: ^{1}H and ^{13}C spectra of **27**

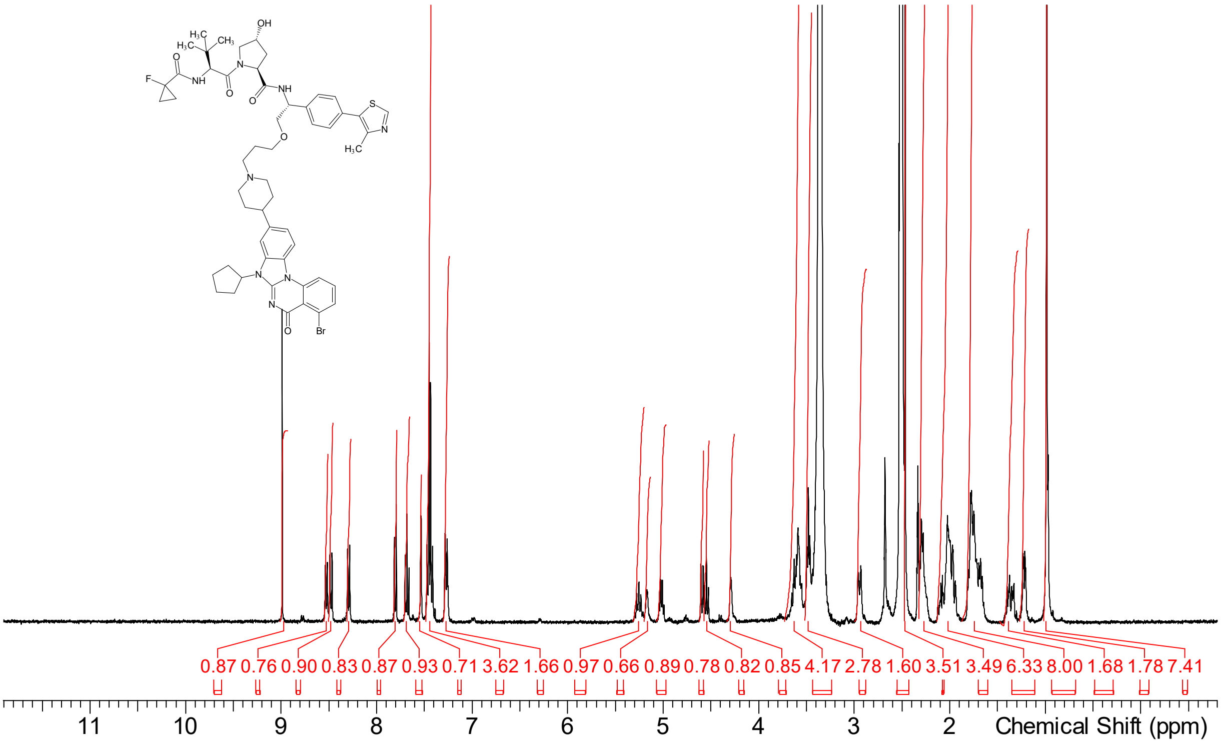

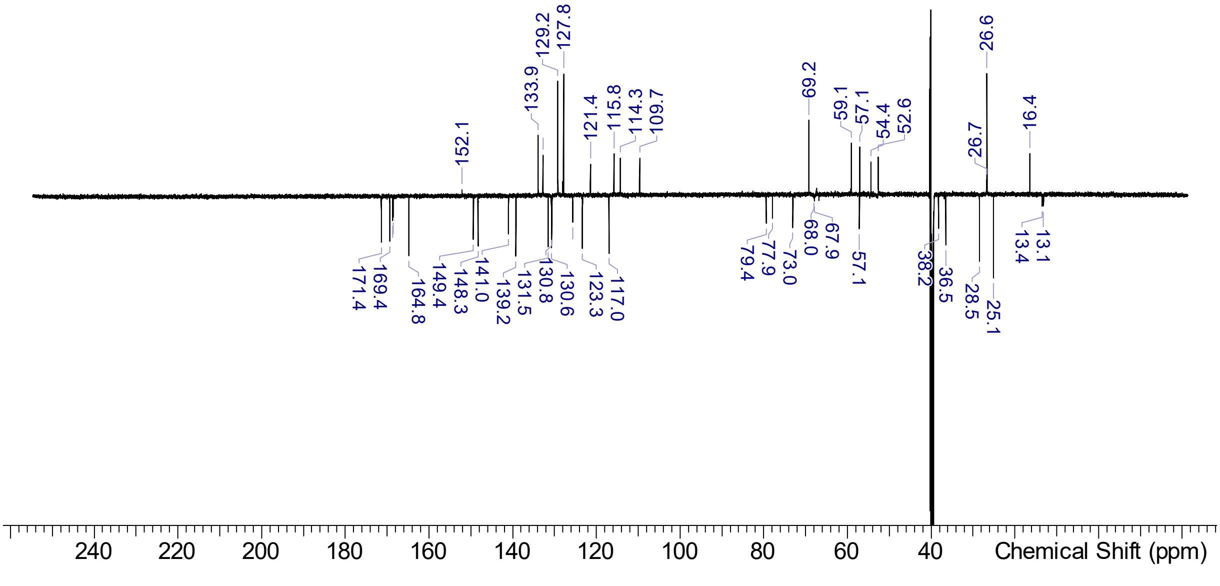

Supplementary Figure 60: ^{1}H and ^{13}C spectra of **28**

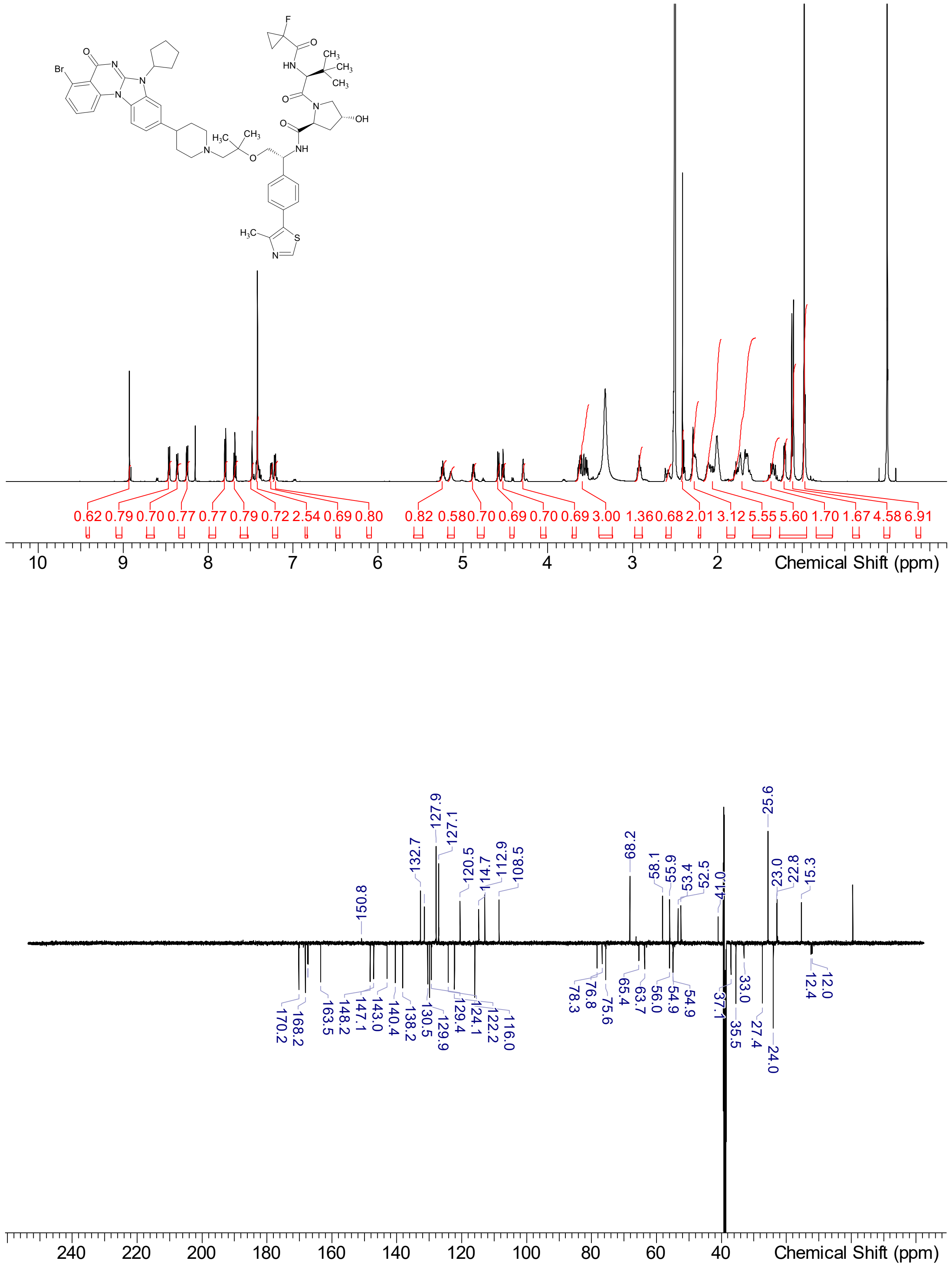

Supplementary Figure 61: ^{1}H and ^{13}C spectra of **29**

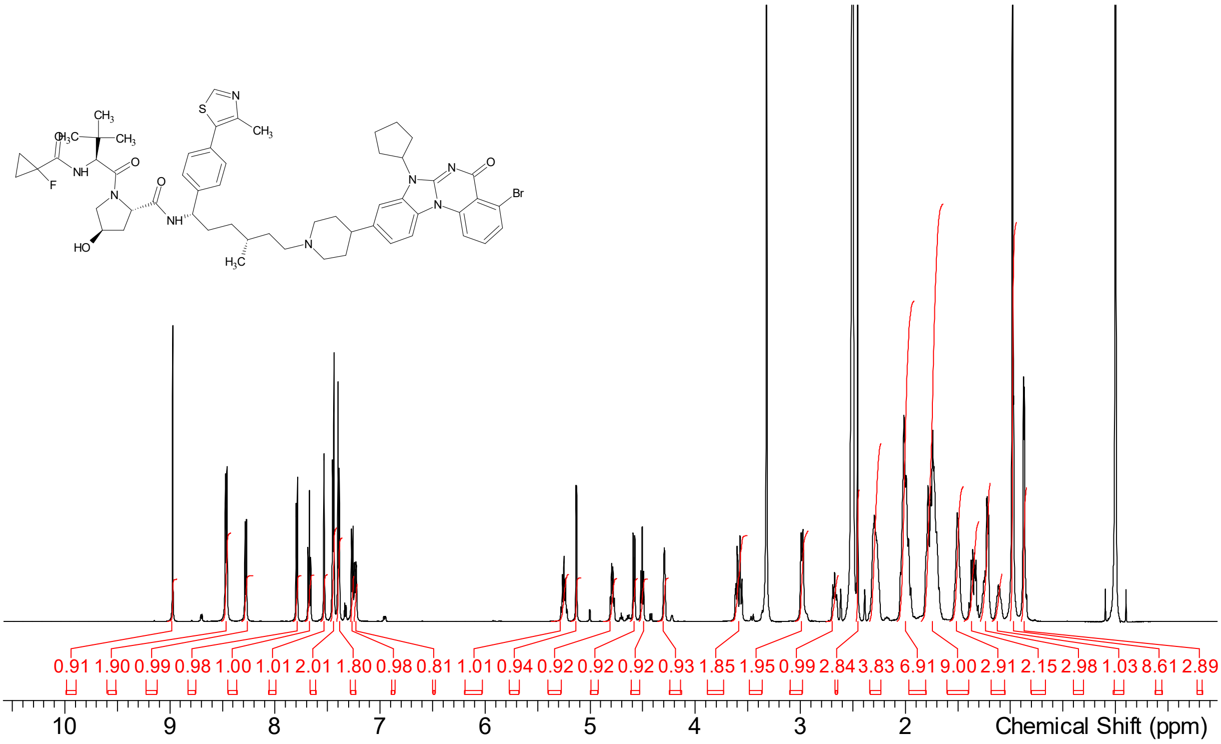

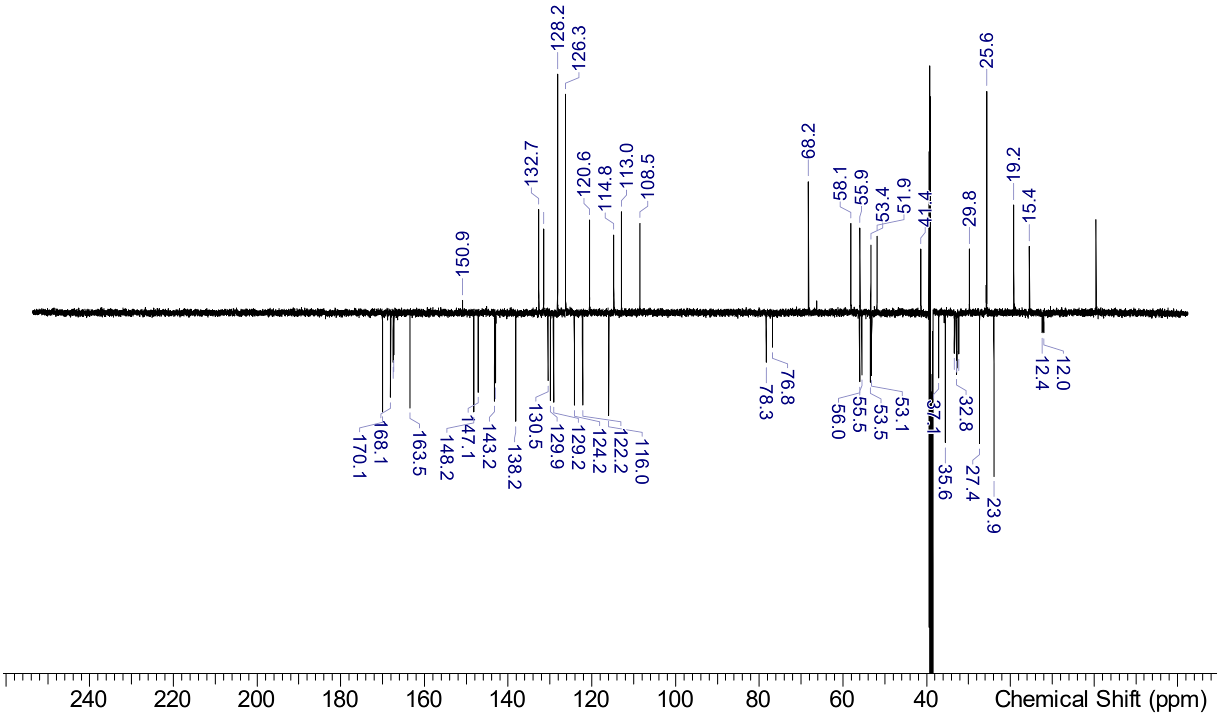

Supplementary Figure 62: ^{1}H and ^{13}C spectra of **30**

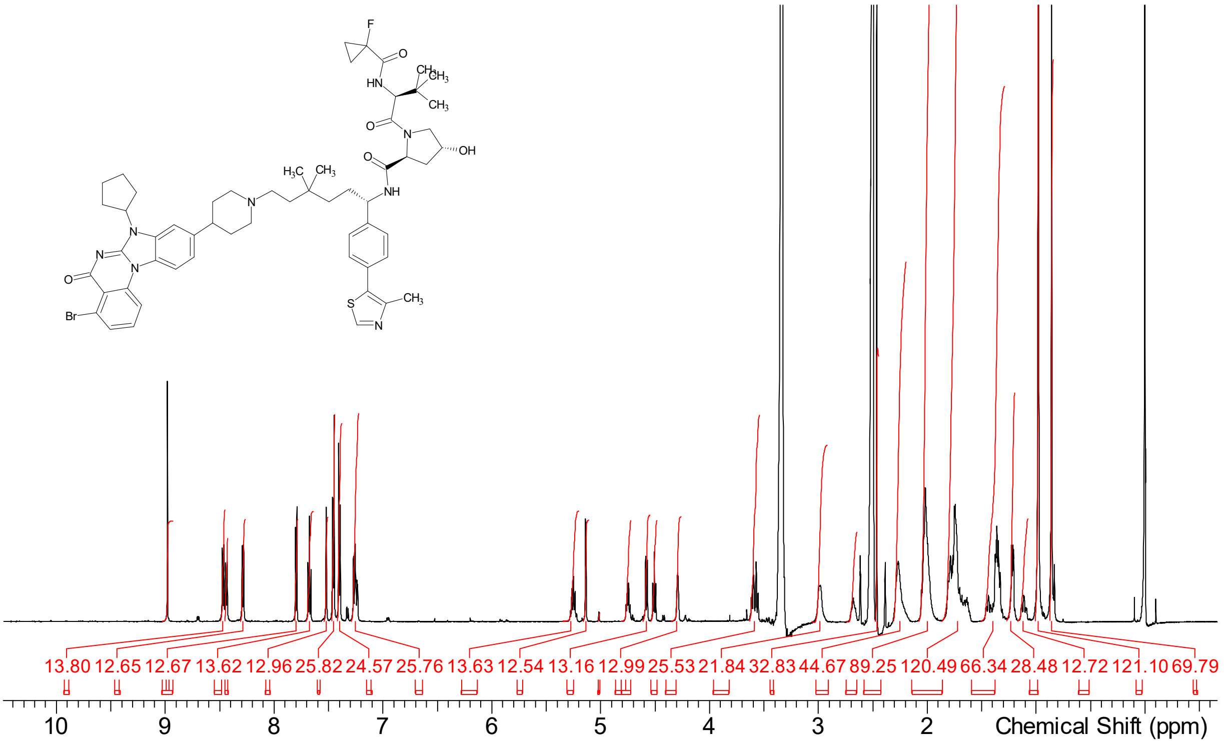

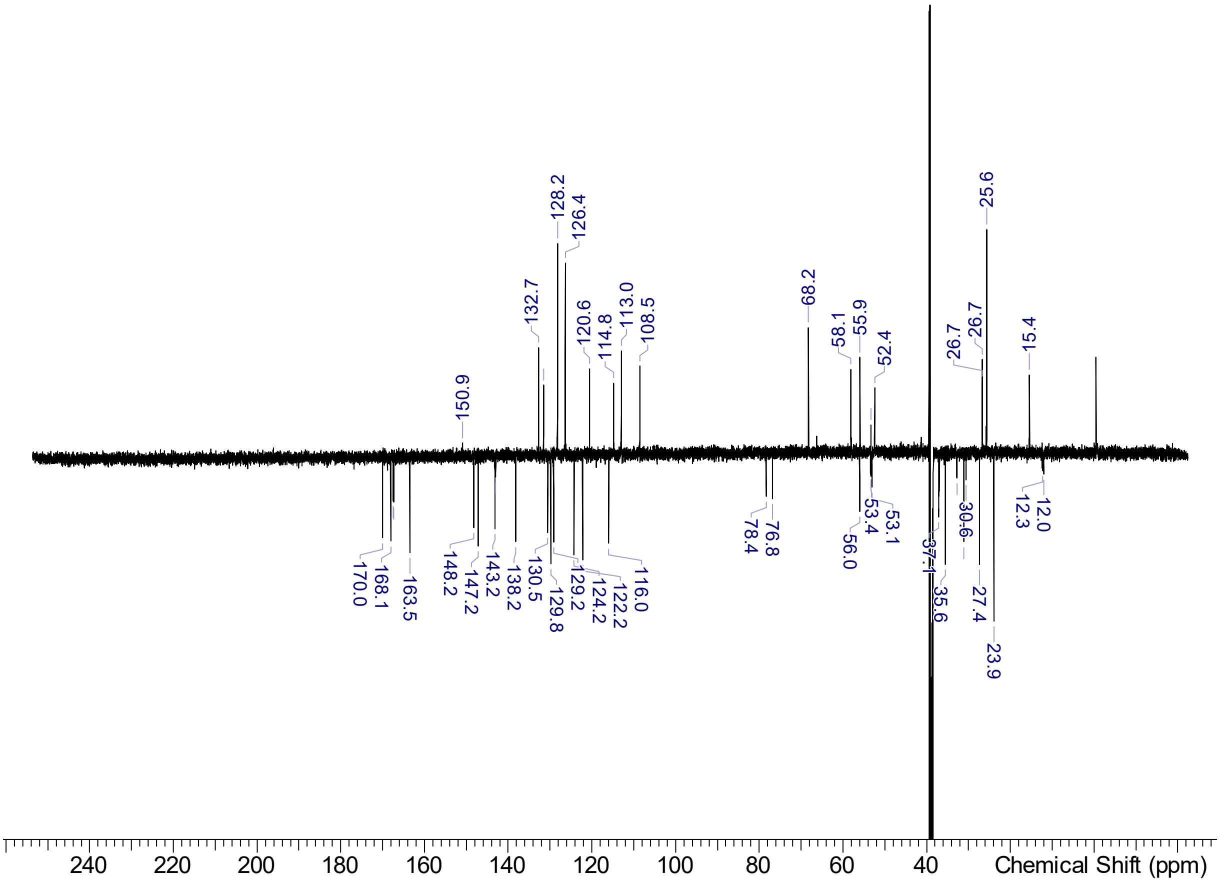

Supplementary Figure 63: ^{1}H and ^{13}C spectra of **31**

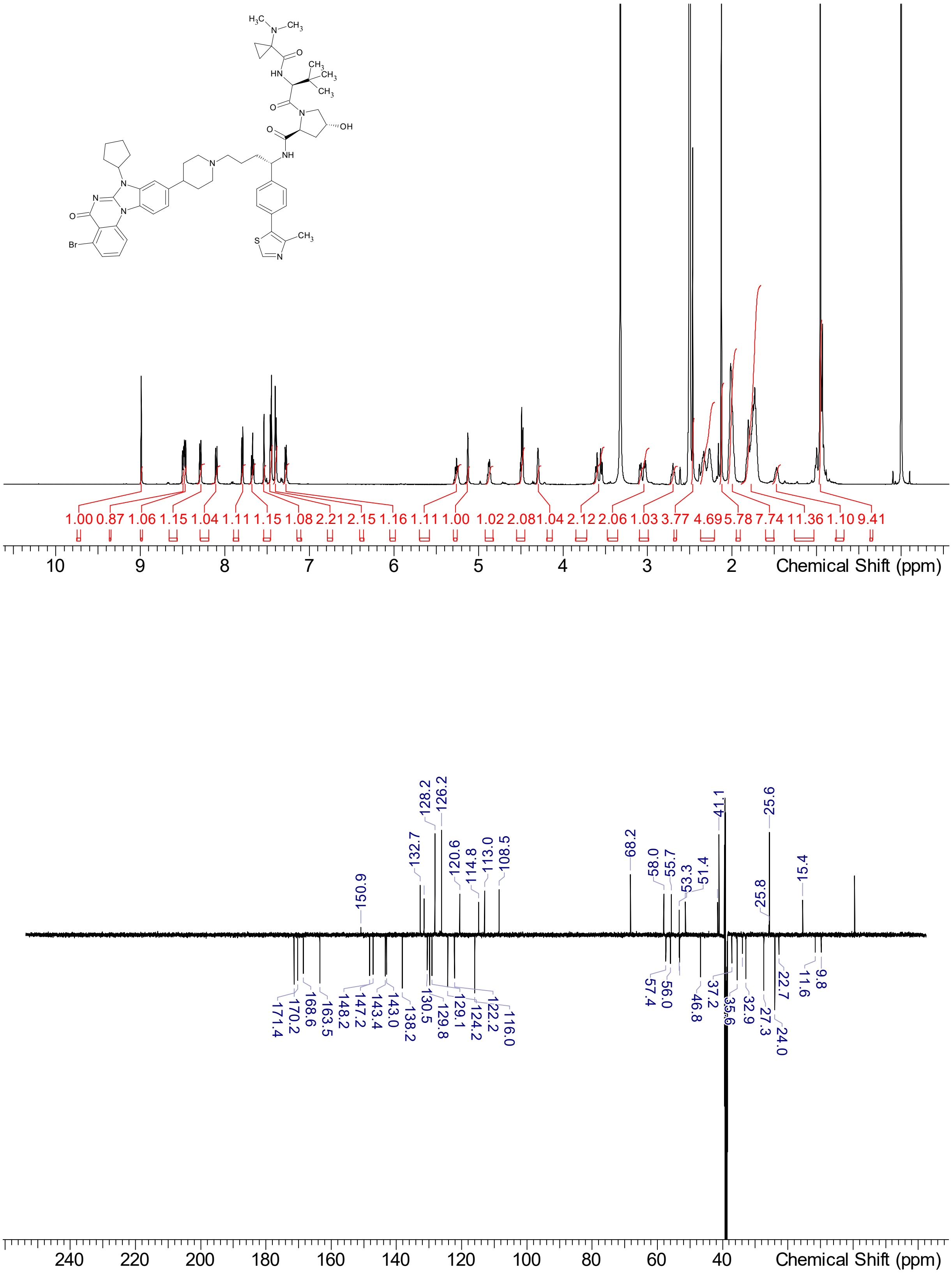

Supplementary References

1 D. Neuhaus, M.P. Williamson, The Nuclear Overhauser Effect in Structural and Conformational Analysis 2nd edition, pp 30ff, Wiley-VCH.

2 Zoppi, V. *et al.* Iterative Design and Optimization of Initially Inactive Proteolysis Targeting Chimeras (PROTACs) Identify VZ185 as a Potent, Fast, and Selective von Hippel–Lindau (VHL) Based Dual Degrader Probe of BRD9 and BRD7. *Journal of Medicinal Chemistry* **62**, 699-726, doi:10.1021/acs.jmedchem.8b01413 (2019).

3 Farnaby, W. *et al.* BAF complex vulnerabilities in cancer demonstrated via structure-based PROTAC design. *Nat Chem Biol* **15**, 672-680, doi:10.1038/s41589-019-0294-6 (2019).

4 Wang, S. *et al.* Androgen receptor protein degraders. WIPO patent WO2020142228 (2020).
