# Supplementary material for: A selective and orally bioavailable VHL-recruiting PROTAC achieves SMARCA2 degradation in vivo
Source: Nat Commun. 2022 Oct 10;13:5969. doi: 10.1038/s41467-022-33430-6 (PMC9551036; doi:10.1038/s41467-022-33430-6)
Supplement: Supplementary file 9 — Reporting Summary [file 41467_2022_33430_MOESM9_ESM.pdf]

nature portfolio

Corresponding author(s): Harald Weinstabl, William Farnaby

Last updated by author(s): Sep 2, 2022

Reporting Summary

Nature Portfolio wishes to improve the reproducibility of the work that we publish. This form provides structure for consistency and transparency in reporting. For further information on Nature Portfolio policies, see our Editorial Policies and the Editorial Policy Checklist.

Statistics

For all statistical analyses, confirm that the following items are present in the figure legend, table legend, main text, or Methods section.

n/a	Confirmed	
☐	☒	The exact sample size (n) for each experimental group/condition, given as a discrete number and unit of measurement
☐	☒	A statement on whether measurements were taken from distinct samples or whether the same sample was measured repeatedly
☐	☒	The statistical test(s) used AND whether they are one- or two-sided<br>*Only common tests should be described solely by name; describe more complex techniques in the Methods section.*
☒	☐	A description of all covariates tested
☐	☒	A description of any assumptions or corrections, such as tests of normality and adjustment for multiple comparisons
☐	☒	A full description of the statistical parameters including central tendency (e.g. means) or other basic estimates (e.g. regression coefficient) AND variation (e.g. standard deviation) or associated estimates of uncertainty (e.g. confidence intervals)
☐	☒	For null hypothesis testing, the test statistic (e.g. F, t, r) with confidence intervals, effect sizes, degrees of freedom and P value noted<br>*Give P values as exact values whenever suitable.*
☒	☐	For Bayesian analysis, information on the choice of priors and Markov chain Monte Carlo settings
☒	☐	For hierarchical and complex designs, identification of the appropriate level for tests and full reporting of outcomes
☒	☐	Estimates of effect sizes (e.g. Cohen's d, Pearson's r), indicating how they were calculated

Our web collection on statistics for biologists contains articles on many of the points above.

Software and code

Policy information about availability of computer code

Data collection	Software used for data collection is mentioned in respective Methods sections:<br>Bruker Topspin version 3.6<br>ProteinSimple Compass multiple versions<br>Maxquant version 2.0.3.0<br>AMBER multiple versions
Data analysis	No custom algorithms or software were generated/used. Software used for analysis is mentioned in respective Methods sections.<br>Bruker Topspin version 3.6<br>MOLPROBITY multiple versions<br>XDS multiple versions<br>PHASER multiple versions<br>CCP4 multiple versions<br>COOT multiple versions<br>autoBUSTER multiple versions<br>PISA multiple versions<br>autoPROC multiple versions<br>STARANISO multiple versions<br>Biacore Insight multiple versions<br>ProteinSimple Compass multiple versions<br>R multiple versions<br>GraphPad Prism multiple versions<br>Microsoft Excel multiple versions

SCIEX Analyst multiple versions
Indica Labs HALO multiple versions
Maxquant version 2.0.3.0
Licor Image Studio Lite version 5.2

For manuscripts utilizing custom algorithms or software that are central to the research but not yet described in published literature, software must be made available to editors and reviewers. We strongly encourage code deposition in a community repository (e.g. GitHub). See the Nature Portfolio guidelines for submitting code & software for further information.

Data

Policy information about availability of data

All manuscripts must include a data availability statement. This statement should provide the following information, where applicable:

- Accession codes, unique identifiers, or web links for publicly available datasets
- A description of any restrictions on data availability
- For clinical datasets or third party data, please ensure that the statement adheres to our policy

Proteomics data generated during the study are available via ProteomeXchange Consortium via the PRIDE44 partner repository, under the dataset identifier PXD032239 [https://www.ebi.ac.uk/pride/archive/projects/pxd032239] (Effects on the proteome of NCI-H1568 cells for compound 6 and ACBI2). X-ray co-crystal data have been deposited to the PDB under accession codes 7Z78 [https://doi.org/10.2210/pdb7Z78/pdb] (compound 4 in complex with SMARCA2BD), 7Z6L [https://doi.org/10.2210/pdb7Z6L/pdb] (VCB : compound 5 : SMARCA2BD complex), 7Z77 [https://doi.org/10.2210/pdb7Z76/pdb] (VCB : compound 6 : SMARCA2BD complex), and 7Z76 [https://doi.org/10.2210/pdb7Z77/pdb] (VCB : compound 10 SMARCA2BD complex). 1 H and 13 C NMR spectra for PROTACs are provided in the Supplementary Information. All other data generated for all Tables, Figures and Supplementary Figures are available in the Source and Supplementary Data files. Plasmids generated in this study are available from the corresponding author upon reasonable request due to restrictions in plasmid repositories for non-academic researchers.

Field-specific reporting

Please select the one below that is the best fit for your research. If you are not sure, read the appropriate sections before making your selection.

[x] Life sciences [ ] Behavioural & social sciences [ ] Ecological, evolutionary & environmental sciences

For a reference copy of the document with all sections, see nature.com/documents/nr-reporting-summary-flat.pdf

Life sciences study design

All studies must disclose on these points even when the disclosure is negative.

Sample size	For in vitro experiments, no dedicated sample size calculations were performed; typically, experiments were repeated at least 3 times independently (sufficient for calculation of mean and SD). In cases where n=3 or larger was not possible for technical reasons, assays were highly optimized with least 2 technical replicates (which were not used to calculate mean or SD). In vivo sample/group sizes were calculated individually for each tumour model based on tumour growth during model establishment experiments. A power analysis was performed using a sample size calculator (https://www.stat.ubc.ca/~rollin/stats/ssize/n2.html). For both models used in the study, 10 mice per group were used for each experiment, in accordance with statistical tests to be performed (see methods and figure legends for details).
Data exclusions	No data exclusions were performed unless indicated and explained otherwise (figure legends).
Replication	All experiments were replicated independently at least twice, each typically with technical replicates. Details are indicated in figure legends.
Randomization	No defined randomization was performed for in vitro experiments but well allocation in cell-based and biochemical assays was arbitrary and varied over independent experimental replicates. Edge wells were left unused to avoid plate effect. Randomizing in in vivo experiments was performed such that average tumour sizes were similar across treatment groups.
Blinding	Blinding was neither possible nor relevant for in vitro or in vivo experiments due to experimental setup to ensure correct sample labeling. No clinical experiments were performed.

Reporting for specific materials, systems and methods

We require information from authors about some types of materials, experimental systems and methods used in many studies. Here, indicate whether each material, system or method listed is relevant to your study. If you are not sure if a list item applies to your research, read the appropriate section before selecting a response.

Materials & experimental systems

n/a	Involved in the study
☐	☒ Antibodies
☐	☒ Eukaryotic cell lines
☒	☐ Palaeontology and archaeology
☐	☒ Animals and other organisms
☐	☒ Human research participants
☒	☐ Clinical data
☒	☐ Dual use research of concern

Methods

n/a	Involved in the study
☒	☐ ChIP-seq
☒	☐ Flow cytometry
☒	☐ MRI-based neuroimaging

Antibodies

Antibodies used

primary ABs:
- SMARCA2 Sigma #HPA029981 RRID:AB_10602406 polyclonal multiple lots, validated for
- SMARCA2 Cell Signaling #11966 RRID:AB_2797783 clone D9E8B multiple lots
- SMARCA4 Cell Signaling #52251 RRID:AB_2799410 clone E9O6 multiple lots
- SMARCA4 Cell Signaling #49360 RRID:AB_2728743 clone D1Q7F multiple lots
- SMARCA4 Abcam #ab110641 RRID:AB_10861578 clone EPNCIR111A multiple lots
- SMARCA4 Abcam #ab108318 RRID:AB_10889900 clone EPR3912 multiple lots
- PBRM1 Bethyl Laboratories #A301-591A RRID:AB_1078808 polyclonal multiple lots
- β-actin Cell Signaling #4970 RRID:AB_2223172 clone 13E5 multiple lots
- GAPDH Abcam #ab9485 RRID:AB_307275 polyclonal multiple lots

secondary ABs:
- Alexa Fluor 647 goat anti mouse IgG Invitrogen #A32728 RRID:AB_2633277 polyclonal multiple lots
- Alexa Fluor 488 goat anti rabbit IgG Invitrogen #A11034 RRID:AB_2576217 polyclonal multiple lots
- IRDye 800CW donkey anti-rabbit IgG LI-COR #926-32213, RRID:AB_621848

Validation

All antibodies were obtained from reputable vendors. Refer to their websites for validation data and relevant citations for the species and application used in this study. Further, all antibodies are profiled in public repositories (RRID). Data provided in this manuscript (pharmacological manipulation of antibody targets, unbiased proteomics) further validates the antibodies used (SMARCA2/4, PBRM1).

Eukaryotic cell lines

Policy information about cell lines

Cell line source(s)

Cell lines were obtained from The University of Texas Southwestern Medical Center (HCC-364, HCC-515), DSMZ (KYSE-30, Caco-2), JCRB (LU99), Charles River (LXFA 629L, LXFA 923L, LXFL 529L , LXFL 1121L) and ATCC (HCC4006, HCT-15, NCI-H1568, NCI-H23, RKO, HCT116, A549, NCI-H1355, SK-MEL-5, OV-90, TOV-112D, SK-N-AS, MP38)

Authentication

Cell lines were authenticated by STR profiling (Eurofins Genomics).

Mycoplasma contamination

All cell lines used in the study were free of mycoplasma contamination in regular checks.

Commonly misidentified lines
(See ICLAC register)

None.

Animals and other organisms

Policy information about studies involving animals; ARRIVE guidelines recommended for reporting animal research

Laboratory animals

Female BomTac:NMRI-Foxn1nu mice were obtained from Taconic Denmark at an age of 6-8 weeks. After arrival at the local AAALAC-accredited animal facility at Boehringer Ingelheim RCV GmbH & Co KG mice were allowed to adjust to housing conditions for at least 5 days before the start of the experiment, i.e. mice in all experiments were 7-9 weeks old. Mice were group-housed under pathogen-free and controlled environmental conditions (21 ± 1.5 °C temperature, 55 ± 10% humidity) and handled according to the institutional, governmental and European Union guidelines (Austrian Animal Protection Laws, GV-SOLAS and FELASA). Animal studies were approved as described in the Ethics section. Food and water were provided ad libitum.

Wild animals

No wild animals were used in the study.

Field-collected samples

No field-collected samples were used in the study.

Ethics oversight

All animal studies were approved by the internal ethics committee (called "ethics committee") of Boehringer Ingelheim RCV GmbH & Co KG in the department of Cancer Pharmacology and Disease Positioning. Furthermore, all protocols were approved by the Austrian governmental committee (MA 60 Veterinary office; approval numbers: GZ: 154399/2018/16 and GZ: MA 58-670393-2019-18).

Note that full information on the approval of the study protocol must also be provided in the manuscript.

Human research participants

Policy information about studies involving human research participants

Population characteristics	No personal data on the included donors was received.
Recruitment	Human whole blood was purchased from the Austrian Red Cross. No recruitment criteria were applied.
Ethics oversight	Human whole blood was purchased from the Austrian Red Cross, who always obtain samples under informed consent in accordance with relevant guidelines, regulations and internal approvals to ensure ethics and IC of donors.

Note that full information on the approval of the study protocol must also be provided in the manuscript.
